# Reductive Hydroxymethylation of 4‐Heteroarylpyridines

**DOI:** 10.1002/chem.202000060

**Published:** 2020-01-30

**Authors:** Hamish B. Hepburn, Timothy J. Donohoe

**Affiliations:** ^1^ Department of Chemistry University of Oxford Chemistry Research Laboratory Mansfield Road Oxford OX1 3TA UK

**Keywords:** aromatic heterocycles, hydroxymethylation, iridium, organic synthesis, reductive functionalization

## Abstract

The activation of pyridinium salts with electron‐withdrawing heterocycles enables an iridium‐catalyzed reductive hydroxymethylation reaction to proceed smoothly, facilitating the preparation of useful 3D heteroaryl‐substituted functionalized piperidines. The methodology is used to prepare 3‐hydroxymethylated analogues of pharmaceutical agents. Mechanistically, formaldehyde acts as both a hydride donor and the electrophile, leading to the formation of two new carbon–hydrogen bonds and one new carbon–carbon bond under relatively mild conditions.

## Graphical abstract

**Reductive functionalization**: The activation of pyridinium salts with a 4‐heteroaryl group allows catalytic reductive hydroxymethylation, leading to the preparation of heterocyclic substituted 3‐hydroxymethylated piperidines (see scheme) with many uses in medicinal chemistry.

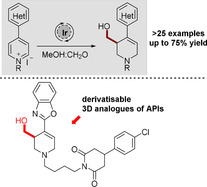

Heterocyclic compounds are highly privileged structural motifs present in a large variety of important molecules, including but not limited to, pharmaceuticals, agrochemicals, natural products, and functional materials.^[1]^ Heterocycles can be found in their fully saturated, partially saturated or unsaturated forms, all of which are highly sought after compounds of great synthetic utility.

Nitrogen‐containing heterocycles are of particular importance due to their chemical and physical properties. A 2014 survey discovered that 59 % of all FDA approved unique small molecule pharmaceuticals contained a nitrogen heterocycle, highlighting their unique importance among medicinally relevant structural motifs.^[2]^ A wide variety of key nitrogen‐containing heterocyclic compounds feature both the saturated and unsaturated form in their molecular skeleton, and, in some cases, heterocycles of differing oxidation levels are adjacent to each other in the molecule. For example, the antipsychotic pharmaceutical Risperidone features an unsaturated benzisoxazole adjacent to a saturated piperidine and a top selling fungicide Zorvec contains an aromatic thiazole adjacent to a piperidine (Scheme 1 a).

**Scheme 1:**
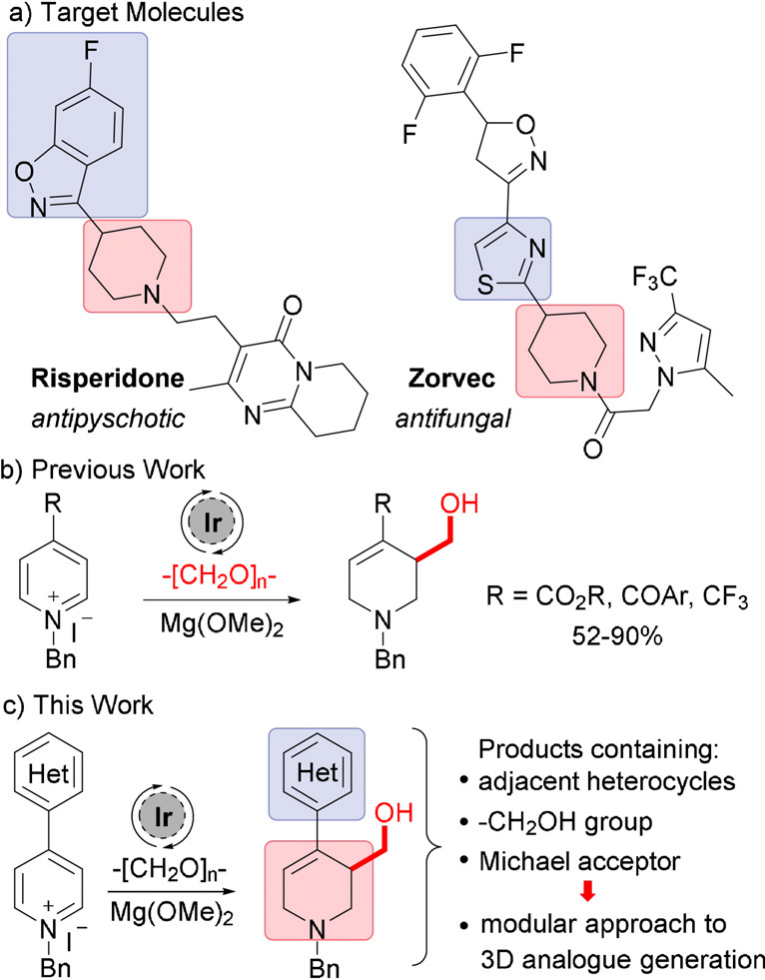
Heterocyclic compounds.

Recently, we have reported an iridium‐catalyzed interrupted transfer hydrogenation process that leads to the reductive hydroxymethylation of electron‐deficient pyridinium salts using cheap and readily available paraformaldehyde as both the hydride source and the electrophile (Scheme 1 b).[^3^, ^4^]

During our investigations, we determined pyridines bearing 4‐carbonyl substituents were excellent substrates for the transformation, but 4‐arylpyridines were insufficiently electron poor to undergo the reductive hydroxymethylation. Analysis showed that this was due to the resulting pyridinium salt being unactivated towards to the initial dearomative addition of iridium‐hydride. Given that is it well precedented in the literature that certain heterocycles are electron‐withdrawing in nature and can activate adjacent olefins towards nucleophilic addition,[^5^, ^6^] combined with the continuing need for the synthetic methodologies that both access saturated heterocycles and install small functional groups, such as CH_2_OH,[^7^, ^8^] we decided to investigate whether 4‐heteroaryl pyridines were sufficiently activated to be utilized in the reductive hydroxymethylation reaction (Scheme 1 c). Not only would this approach provide rapid access to 4‐heteroaryl‐tetrahydropiperidines bearing multiple functionality but it would also allow the modular preparation of 3D analogues of potential agrochemicals and pharmaceuticals from readily available (flat) pyridine starting materials.

To investigate the potential of heteroarylpyridines as suitable substrates, 4‐benzoxazolepyridinium iodide **1 a** was chosen as a model substrate and was subjected to conditions for the iridium‐catalyzed hydroxymethylation, [IrCp*Cl_2_]_2_ (1 mol %), Mg(OMe)_2_ (0.75 equiv), paraformaldehyde (20 equiv), and potassium iodide (4 equiv)^[9]^ in MeOH at 65 °C for 16 hours (Table 1, entry 1).^[3]^ Pleasingly, 65 % of the hydroxymethylated tetrahydropiperidine **2 a** was isolated, indicating that benzoxazoles were sufficiently electron‐withdrawing to facilitate initial reduction of the pyridinium salt. With this result in hand, an optimization was initiated, aiming at increasing the isolated yield of the product **2 a** (Table 1). Reducing the temperature to 45 °C was found to have minimal effect (entry 2) as did decreasing the equivalents of KI from four to two (entry 3). Switching base to NaOMe resulted in complete consumption of **1 a** but led to no formation of **2 a** (entry 4). However, it was found that increasing the loading of paraformaldehyde to 25 (71 %, entry 5) and 30 equivalents (73 %, entry 6) had a positive effect on the yield, but 40 equivalents led to a decrease in yield (63 %, entry 7). Additionally, doubling the equivalents of base, led to no improvement (entry 8) but reducing the temperature to room temperature led to a small yet appreciable increase to 76 % yield (entry 9). Finally, a control experiment determined that no product was formed in the absence of iridium (entry 10).

**Table 1:** Iridium‐catalyzed hydroxymethylation of **1 a**.

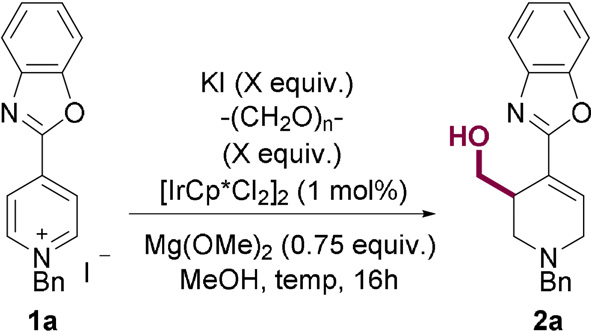				
Entry	Paraformaldehyde (equiv)	KI (equiv)	*T* [°C]	Yield [%]
1	20	4	65	65
2	20	4	45	66
3	20	2	45	66
4^[a]^	20	2	45	0
5	25	2	45	71
6	30	2	45	73
7	40	2	45	63
8^[b]^	30	2	45	73
9	30	2	25	76
10^[c]^	30	2	45	0

[a] Using NaOMe (1.5 equiv) as base. [b] Using 1.5 equiv of Mg(OMe)_2_. [c] With no iridium.

Having developed optimal conditions for the transformation, the scope of the 4‐benzoxazolepyridine substrates was explored. However, an issue of solubility was rapidly encountered; many of the pyridinium salts exhibited very limited solubility in methanol at room temperature, leading to low conversions. This issue was overcome by increasing the temperature of the reaction to 45 °C which lead to a fully homogeneous reaction mixture, despite the slight reduction in isolated yield for some more soluble substrates. The simple reduced product **3** was observed in almost all cases in around 10 % yield.^[10]^ However, these compounds were easily removed by column chromatography (Scheme 2).

**Scheme 2:**
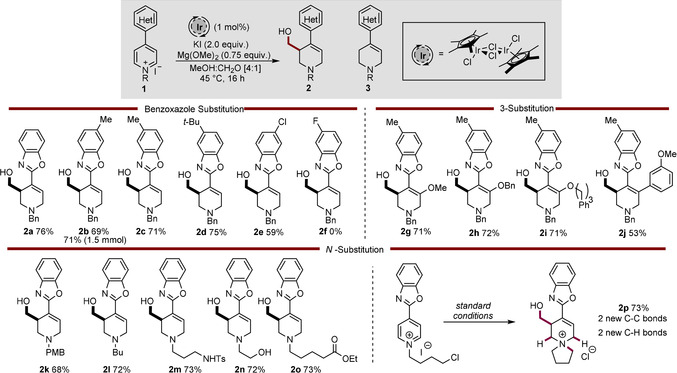
Scope of the reductive hydroxymethylation of 4‐benzoxazole pyridines.

A variety of substituents are tolerated on the carbocyclic ring of the benzoxazole, including electron‐rich (**2 b**–**2 d**) and electron‐withdrawing functional groups (**2 e**), in both the 5‐, and 6‐ positions with good yields (62 to 75 %). Undertaking the reaction at a scale of 1.5 mmol proceeded well delivering **2 b** in 71 % yield. Unfortunately, the presence of a fluoride group (**2 f**) on the benzoxazole lead to degradation of the starting material and no product was observed in the ^1^H NMR spectrum of the reaction mixture. The 3‐position of the pyridine ring could be substituted with a range of different groups, giving good yields throughout, including simple (**2 g**–**h**) and more complex ethers (**2 i**) along with aryl rings (**2 j**). The substituent on the pyridine nitrogen atom can also be varied greatly, alternative protecting groups, such *para*‐methoxybenzyl (PMB) are tolerated (**2 k**), as are other functionalized alkyl chains (**2 l**–**o**) opening the possibility to install functionality before the reductive hydroxymethylation, removing the necessity for a protecting group. Furthermore, these examples show that a variety of potentially sensitive functional groups are well tolerated under the conditions, including −NHTs (**2 m**, 73 %), −OH (**2 n**, 72 %) and −CO_2_Et (**2 o**, 73 %). Entry **2 o** is of particular interest as it demonstrates that a) despite the basic conditions, the presence of enolizable protons did not hinder the reaction progress and b) despite the presence of methoxide and an excess of methanol, the ethyl ester was conserved. Interestingly, when substrate **1 p**, bearing an *N*‐4‐chlorobutyl group was subjected to the standard reaction conditions, the expected product was not obtained, but rather spirocyclic ammonium species **2 p** was isolated in 73 % yield. This can be rationalized due to the expected tetrahydropyridine product featuring a nucleophilic nitrogen atom which can undergo a spontaneous intramolecular attack onto the alkyl chloride, forming **2 p** and forging two new carbon–carbon and two new carbon–hydrogen bonds. This example indicates that with judicious substrate design, this methodology presents the opportunity to develop cascade processes to prepare more complex molecules in one synthetic step.

Pleasingly, the structure of the activating aromatic heterocycle is not limited to the use of benzoxazole and a variety of other electron‐withdrawing heterocyclic groups were successfully used as activating substituents (Scheme 3). Heterocycles that can be utilized include benzothiazole (**2 q**, 66 %), oxadiazoles (**2 r**–**s**, 52–57 %), thiazole (**2 t**, 54 %), and oxazoles (**2 u**–**w**, up to 65 %). Additionally, benzisoxazole, a motif featured in pharmaceuticals such a Risperidone in Scheme 1, was found to give the expected product **2 x** in moderate yield. This flexibility in activating the heterocycle, allows for the synthesis of wide variety of useful 3‐hydroxymethylated‐4‐heteroaryl piperidines with relative ease, allowing access to a range of useful products.

**Scheme 3:**
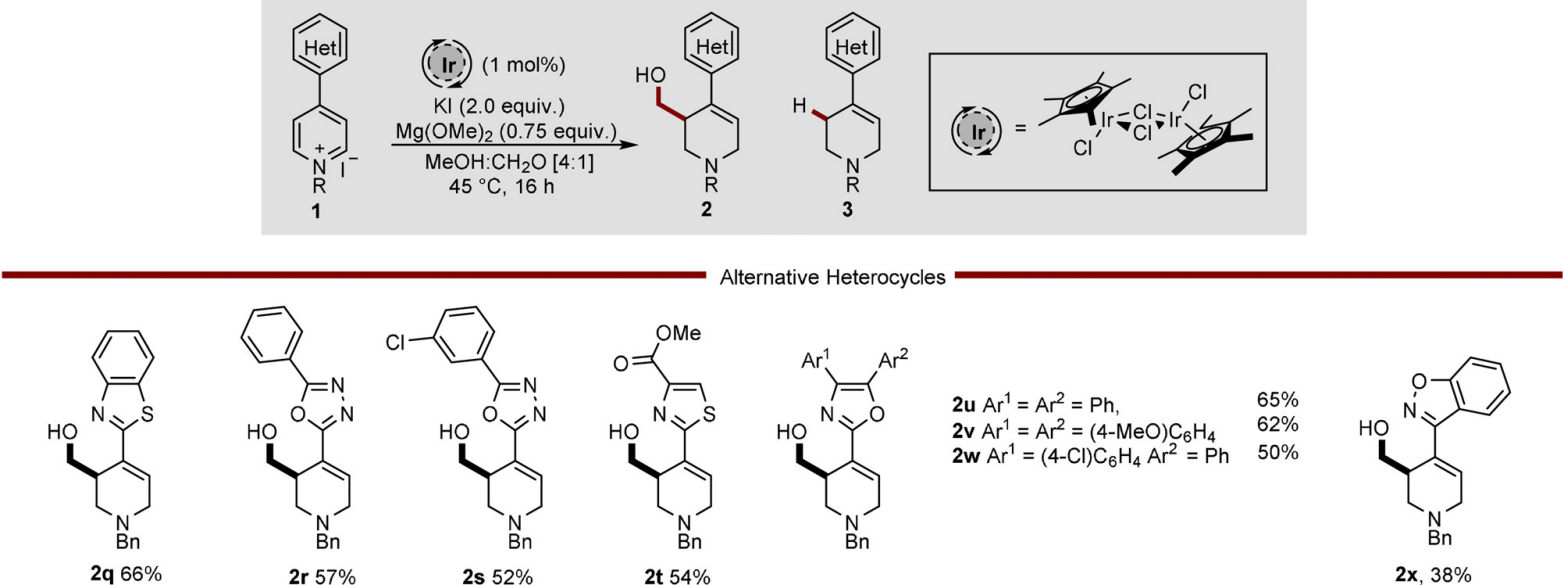
Scope of the reductive hydroxymethylation of heterocycle‐activated pyridines.

To showcase the versatility of this method and its relevance to the drug discovery process, we utilized it in the late‐stage preparation of analogues of medicinally relevant 4‐heteroaryl piperidines, such as the anti‐psychotic pharmaceutical Iloperidone (Scheme 4). This approach is highly modular and convergent: in step I aromatic and aliphatic components are joined together by alkylation, thus activating the pyridine; next, the dearomative functionalization reaction (step II) was performed which led to structures with ample functionality for further derivatisation and exploration of 3D chemical space (step III). Thus, a set of medicinal lead inspired 4‐heteroarylpyridines and functionalized alkyl iodides were heated together to furnish highly decorated pyridinium salts (**1 aa–ad**) which were then subjected to the iridium‐catalyzed process, delivering the 3‐hydroxymethylated analogues **2 aa–ad** in good yields.[^11^, ^12^, ^13^, ^14^]

**Scheme 4:**
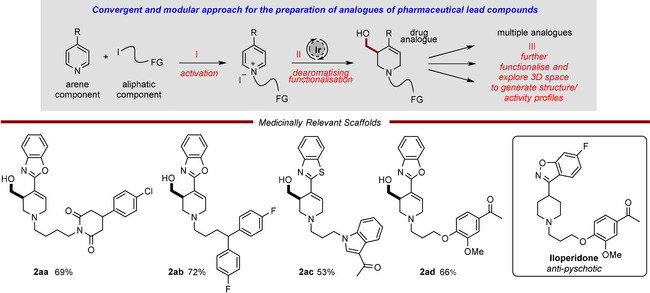
Convergent preparation of medicinally relevant piperidine scaffolds.

This synthetic sequence is operationally simple and delivers products bearing multiple new functional groups, such as an activated electron‐deficient alkene and a primary alcohol, allowing many possibilities for the additional functionalization reactions which are essential for structure–activity relationship studies. Furthermore, in part due to the relatively mild conditions for transition‐metal catalysis (45 °C and a mild base), a wide range of potentially sensitive functional groups, such as cyclic imides, aromatic halides, enolizable ketones, electron‐rich heterocycles, and aromatic ethers, were all tolerated under reaction conditions and do not hinder the catalytic reaction.

Mechanistically, we believe the reaction proceeds under a pathway that is depicted in Scheme 5.^[3a]^ Addition of methanol to formaldehyde generates hemiacetal **5**, which is then oxidized by iridium to generate methyl formate and an iridium‐hydride species. Addition of iridium‐hydride occurs at the C^2^‐position of **1 a** due to the presence of the electron‐withdrawing heterocycle in the C^4^‐position, resulting in the formation of enamine **6**. The trapping of formaldehyde by **6**, leads to the formation of a new carbon–carbon bond and the iminium **7**, which is readily reduced by a second equivalent of iridium‐hydride to furnish product **2 a**. Supporting this pathway, ^1^H NMR analysis of the crude reaction indicated the presence of formate byproducts, consistent with the oxidation of formaldehyde.

**Scheme 5:**
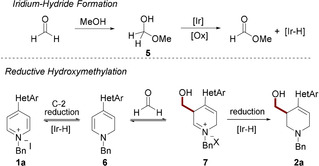
Proposed mechanistic pathway.

In conclusion, we have developed the iridium‐catalyzed reductive hydroxymethylation of activated 4‐heteroaryl pyridines, giving rise to highly useful decorated 4‐heteroarylpiperidines using methanol and formaldehyde as reagents. The methodology has been used in the late‐stage preparation of medicinally relevant scaffolds demonstrating its importance for medicinal chemistry and its tolerance of a wide range of functional groups. In our laboratory we are continuing to investigate other activation strategies and substrates which have the potential to be proficient in this methodology.

## Conflict of interest

The authors declare no conflict of interest.

## Supporting information

As a service to our authors and readers, this journal provides supporting information supplied by the authors. Such materials are peer reviewed and may be re‐organized for online delivery, but are not copy‐edited or typeset. Technical support issues arising from supporting information (other than missing files) should be addressed to the authors.

Supplementary
