# Supplementary material for: Reductive Hydroxymethylation of 4‐Heteroarylpyridines
Source: Chemistry. 2020 Jan 30;26(9):1963–7. doi: 10.1002/chem.202000060 (PMC7754387; doi:10.1002/chem.202000060)
Supplement: Supplementary file 1 — Supplementary [file CHEM-26-1963-s001.pdf]

CHEMISTRY
A European Journal

Supporting Information

Reductive Hydroxymethylation of 4-Heteroarylpyridines

Hamish B. Hepburn and Timothy J. Donohoe*[a]

chem_202000060_sm_miscellaneous_information.pdf

Contents

General Experimental Techniques ... 2

Preparation of Starting Materials ... 3

Preparation of Pyridinium Salts ... 13

Reductive Hydroxymethylation ... 27

Spectra ... 40

General Experimental Techniques

Chemicals and solvents

Unless stated otherwise, all chemicals were purchased from commercial suppliers (Sigma-Aldrich, Fluorochem, Alfa Aesar) and used without further purification. The magnesium methoxide was purchased from Sigma-Aldrich as a 6-10% w/w solution in methanol and was titrated using EDTA in the presence of Eriochrome Black T as an indicator. Benzyl iodide was prepared according to literature procedure.[1]

Glassware and reaction conditions

Reactions were carried out in oven-dried microwave vials under an atmosphere of air unless otherwise stated.

Analytical techniques

^{1}H and ^{13}C NMR spectra were recorded on a Bruker AVIII400 Spectrometer (400 MHz and 100 MHz respectively) or a Bruker AVII500 (^{1}H: 500 MHz and ^{13}C: 126 MHz) in $CDCl_3$ or DMSO-d_6, and referenced to residual solvent peaks. Chemical shifts δ are quoted in parts per million (ppm) to the nearest 0.01 for ^{1}H and 0.1 for ^{13}C, coupling constants *J* are quoted in Hz to the nearest 0.1 and splitting are recorded as singlet (s), doublet (d), triplet (t), quartet (q), pentet (p), doublet of a doublet (dd), doublet of a doublet of a doublet (ddd), and multiplet (m). Assignments were based upon COSY, HSQC and HMBC experiments. Infrared spectra were recorded on a Bruker Tensor 27 FT-IR spectrometer fitted with an Attenuated Total Reflectance (ATR) sampling accessory. Absorption maxima are quoted in wavenumbers (cm^{-1}). Mass spectra were recorded on a Fisons Platform II spectrometer under electrospray ionisation (ESI). High resolution mass spectra are given to four decimal places and were recorded on a Bruker MicroTof (resolution = 10000 FWHM). Melting points (m.p.) were obtained using a Lecia VMGT heated-stage microscope and are uncorrected.

Chromatography

Analytical thin layer chromatography was performed on pre-coated silica gel aluminium sheets from Merck (TLC Silica Gel 60 F_{254s}). Spots were visualized either by the quenching of UV fluorescence or by staining with phosphomolybdic acid solution. Preparative flash column chromatography was carried out using Geduran Silica Gel 60 (40 µm – 63 µm) from Merck.

---

[1] Hoang, C. T.; Alezra, V.; Guilot, R.; Kouklovsky, C. *Org. Lett.* **2007**, *9*, 2521-2524.

Preparation of Starting Materials

General Procedure A: Preparation of 4-(Benzoxazole)pyridines

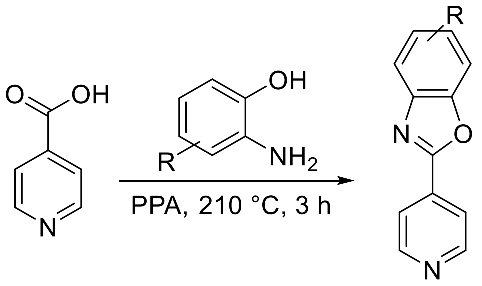

2-Aminophenol (1.0 equiv.) and isonicotinic acid (1.0 equiv.) were added to polyphosphoric acid (2.5 g per mmol) and the resulting mixture was heated under argon at 210 °C for 3 hours. The solution was cooled to 160 °C and poured into 100 mL of ice water and allowed to cool. The acidic solution was neutralised with NaOH solution and filtered, resulting in the formation of a white paste on the filter. The paste was dissolved with a mixture of EtOAc and acetone and then concentrated to remove the acetone. The EtOAc solution was dried ($MgSO_4$), filtered, and concentrated *in vacuo* to yield *pyridine* as a powder.

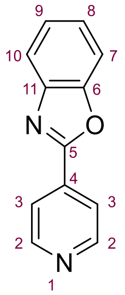

4-(Benzoxazole)pyridine (S1)

The title compound was prepared according to General Procedure A using 2-aminophenol (3.27 g, 30 mmol) to give *pyridine* **S1** (4.50 g, 77%) as a cream solid. The spectroscopic data matched the previous literature reports.[2]

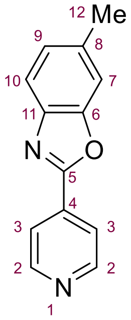

4-(5-Methylbenzoxazole)pyridine (S2)

The titled compound was by prepared according to General Procedure A using 2-amino-5-methylphenol (1.23 g, 10 mmol) to give *pyridine* **S2** (1.14 g, 54%) as a beige solid. The spectroscopic data matched the previous literature reports.[3]

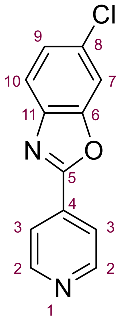

4-(5-Chlorobenzoxazole)pyridine (S3)

The title compound was prepared according to General Procedure A using 2-amino-5-chlorophenol (1.43 g, 10 mmol) to give an impure powder. The crude material was purified by flash column chromatography (50% EtOAc in pentane) to give *pyridine* **S3**

[2] Mao, S.; Zhang, H.; Shen, K.; Xu, Y.; Shi, X.; Wu, H. *Polyhedron*, **2017**, *134*, 336-344.

[3] Sumitomo Chemical Company, Limited - European Patent EP2274983, 2011, A1

(650 mg, 28%) as a pink solid. The spectroscopic data matched the previous literature reports.[4]

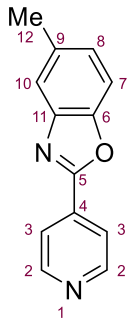

4-(4-Methylbenzoxazole)pyridine (S4)

The title compound was prepared according to General Procedure A using 2-amino-4-methylphenol (1.23 g, 10 mmol) to give *pyridine* **S4** (1.06 g, 50%) as a beige solid. The spectroscopic data matched the previous literature reports.[5]

General Procedure A2: Preparation of 4-(Benzoxazole)pyridines

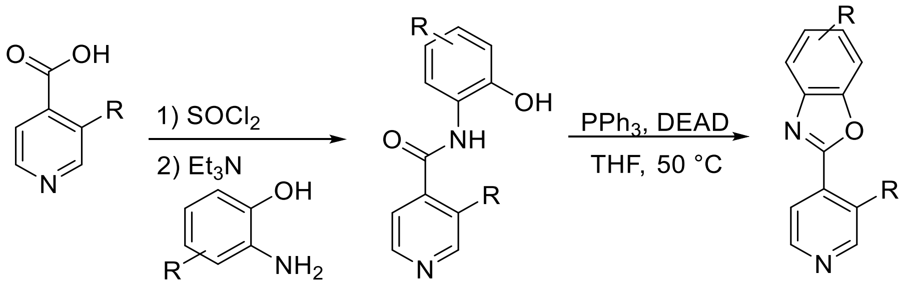

Step 1: Isonicotinic acid (1 equiv.) was dissolved in 5 mL of $SOCl_2$ and heated to reflux for 1 hour. The excess $SOCl_2$ was removed by rotary evaporation to furnish the acid chloride as a white solid. 2-Aminophenol (1.0 equiv.) and triethylamine (1.1 equiv.) were dissolved in toluene and the acid chloride was added careful to the solution, portionwise. The solution as heated at reflux for 6 hours, cooled to room temperature and filtered. The solid (which consists of amide and triethylamine hydrochloride) was suspended in water and sonicated for 10 minutes. The resulting suspension is filtered and washed with diethyl ether to furnish amide.

Step 2: The amide (1.0 equiv.) and triphenylphosphine (1.2 equiv.) were dissolved in THF and DEAD (1.2 equiv.) was added slowly to the solution. The solution was heated at 50 °C and monitored by TLC. Once the starting material had fully converted to product (indicated by a new less polar spot, blue under 254 nm UV, on the TLC) the reaction was cooled to room temperature (usually 1.5-5 hours). The solution was diluted with EtOAc and water and extracted three times with EtOAC. The organic layers were combined, dried, filtered and concentrated *in vacuo*. The crude material was purified by FCC to furnish 4-benzoxazolepyridine.

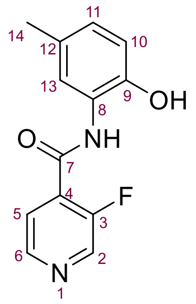

3-Fluoro-*N*-(2-hydroxy-5-methylphenyl)isonicotinamide (S5).

The title compound was prepared according to step 1 of General Procedure A2 using 3-fluoroisonicotinic acid (2.82 g, 20 mmol) and 2-amino-4-methylphenol (2.46 g, 20 mmol) to give *amide* **S5** (2.82 g, 11.4 mmol 57%) as a beige solid.

m.p. (acetone): 224-226 °C;

HRMS (ESI): Exact mass calculated for $C_{13}H_{12}O_2N_2F$ $[M+H]^+$: 247.08773, found: 247.08783;

[4] VASTOX PLC - Patent WO2007/91106, **2007**, A2

[5] Zhou, Q.; Liu, S.; Ma, M.; Cui, H.-Z.; Hong, X.; Huang, S.; Zhang, J.-F.; Hou, X.-F. *Synthesis*, **2018**, *50*, 1315-1322.

^{1}H NMR ($(CD_3)_2SO$) δ 9.76 (1H, br s, O**H**), 9.70 (1H, br d, *J* = 4.7 Hz, N**H**), 8.75 (1H, d, *J* = 2.0 Hz, C^{2}**H**), 8.59 (1H, dd, *J* = 4.9, 1.3 Hz, C^{6}**H**), 7.78 (1H, d, *J* = 1.9 Hz, C^{5}**H**), 7.76 (1H, dd, *J* = 6.3, 4.9 Hz, C^{11}**H**), 6.90-6.74 (2H, m, C^{10}**H** + C^{13}**H**), 2.23 (3H, s, C^{14}**H**$_3$);
^{13}C NMR ($(CD_3)_2SO$) δ 160.1 (C^{7}), 155.4 (d, *J* = 256.8 Hz, C^{3}), 146.4 (d, *J* = 4.8 Hz, C^{6}), 145.9 (C), 139.0 (d, *J* = 24.8 Hz, C^{2}), 130.4 (d, *J* = 12.2 Hz, C^{4}), 127.6 (C), 126.0 (C^{10}), 125.1 (C), 123.6 (C^{11}), 123.0 (C^{5}), 115.2 (C^{13}), 20.5 (C^{14});
^{19}F NMR ($(CD_3)_2SO$) δ –129.1;
IR (neat) (cm^{-1}): 3398, 2361, 2161, 1722, 1650, 1558, 1491, 1458, 1352, 1282, 1199, 1130, 1096.

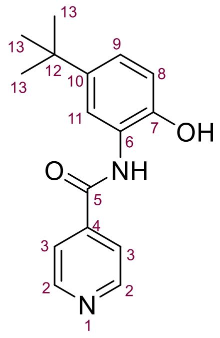

***N*-(2-Hydroxy-5-*tert*-butylphenyl)isonicotinamide (S6).**
The title compound was prepared according to step 1 of General Procedure A2 using isonicotinic acid (1.23 g, 10 mmol) and 2-amino-4-*tert*-butylphenol (1.65 g, 10 mmol) to give *amide* **S6** (1.67 g, 62%) as a yellow solid.
m.p. (acetone): 195-197 °C;
HRMS (ESI): Exact mass calculated for $C_{16}H_{19}O_2N_2$ $[M+H]^+$: 271.14410, found: 271.14398;
^{1}H NMR ($(CD_3)_2SO$) δ 9.85 (1H, s, O**H**), 9.48 (1H, s, N**H**), 8.78 (2H, d, *J* = 6.2 Hz, 2 x C^{2}**H**), 7.89 (2H, d, *J* = 6.1 Hz, 2 x C^{3}**H**), 7.59 (1H, d, *J* = 2.4 Hz, C^{11}**H**), 7.10 (1H, dd, *J* = 8.5, 5.2 Hz, C^{9}**H**), 6.86 (1H, d, *J* = 8.5 Hz), 1.25 (9H, s, 3 x C^{13}**H**$_3$);
^{13}C NMR ($(CD_3)_2SO$) δ 163.7 (C^{5}), 150.2 (2 x C^{2}), 147.9 (C), 141.7 (C), 141.3 (C), 124.4 (C), 123.3 (C^{9}), 122.1 (C^{11}), 121.6 (2 x C^{3}), 115.7 (C^{8}), 33.8 (C^{12}), 31.4 (3 x C^{13});
IR (neat) (cm^{-1}):3379, 2970, 2899, 2161, 1723, 1650, 1557, 1472, 1392, 1227, 1201, 1092, 1069.

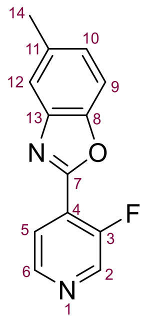

3-Fluoro-4-(4-methylbenzoxazole)pyridine (S7)
The title compound was prepared according to step 2 of General Procedure A2 using 3-fluoro-*N*-(2-hydroxy-5-methylphenyl)isonicotinamide (2.33 g, 9.5 mmol) and was purified by FCC (10-30% EtOAc in pentane) to give *pyridine* **S7** (2.14 g, 99%) as a cream solid.
m.p. (acetone): 90-92 °C;
HRMS (ESI): Exact mass calculated for $C_{13}H_{10}ON_2F$ $[M+H]^+$: 229.07717, found: 229.07722;
^{1}H NMR ($CDCl_3$) δ 8.72 (1H, d, *J* = 2.6 Hz, C^{2}**H**), 8.61 (1H, dd, *J* = 5.0, 0.9 Hz, C^{6}**H**), 8.11 (1H, dd, *J* = 6.1, 5.1 Hz, C^{5}**H**), 7.66 (1H, dt, *J* = 1.6, 0.8 Hz, C^{12}**H**), 7.54 (1H, d, *J* = 8.4 Hz, C^{9}**H**), 7.31-7.25 (1H, m, C^{10}**H**), 2.52 (3H, s, C^{14}**H**);
^{13}C NMR ($CDCl_3$) δ 157.1 (C), 156.1 (d, *J* = 268.6 Hz, C^{3}), 148.9 (C), 146.1 (d, *J* = 5.6 Hz, C^{6}), 141.7 (C), 140.3 (d, *J* = 24.5 Hz, C^{2}), 135.2 (C), 127.9 (C^{10}), 122.7 (C^{5}), 122.3 (C), 120.7 (C^{12}), 110.4 (C^{9}), 21.6 (C^{14});
^{19}F NMR ($CDCl_3$) δ –125.5;
IR (neat) (cm^{-1}): 2923, 2161, 1622, 1574, 1481, 1385, 1312, 1274, 1221, 1205, 1130, 1053, 871.

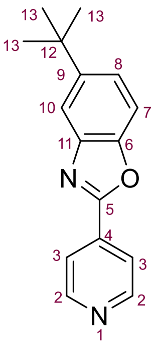

4-(4-*tert*-Butylbenzoxazole)pyridine (S8)
The title compound was prepared according to step 2 of General Procedure A2 using *N*-(2-hydroxy-5-*tert*-butylphenyl)isonicotinamide 1.35 g, 5 mmol) and was purified by FCC (10-30% EtOAc in pentane) to give *pyridine* **S8** (990 mg, 79%) as a white solid.
m.p. (acetone): 115-117 °C;
HRMS (ESI): Exact mass calculated for $C_{16}H_{17}ON_2$ $[M+H]^+$: 253.13354, found: 253.13350;
^{1}H NMR ($CDCl_3$) δ 8.82 (2H, d, *J* = 4.5 Hz, 2 x C^{2}**H**), 8.08 (2H, d, *J* = 4.5 Hz, 2 x C^{3}**H**), 7.84 (1H, dd, *J* = 1.9, 0.7 Hz, C^{10}**H**), 7.54 (1H, dd, *J* = 8.7, 0.7 Hz, C^{7}**H**), 7.50 (1H, dd, *J* = 8.7, 1.9 Hz, C^{8}**H**), 1.41 (9H, s, 3 x C^{13}**H**$_3$);
^{13}C NMR ($CDCl_3$) δ 160.7 (C), 150.7 (2 x C^{2}), 148.8 (C), 148.7 (C), 141.6 (C), 134.3 (C), 124.2 (C^{8}), 120.9 (2 x C^{3}), 117.0 (C^{10}), 110.0 (C^{7}), 35.0 (C^{9}), 31.7 (3 x C^{13});

IR (neat) (cm^{-1}): 3240, 2868, 2161, 1748, 1617, 1602, 1543, 1481, 1391, 1340, 1296, 1219, 1139, 1085, 1068, 1058.

3-Methoxy-4-(4-methylbenzoxazole)pyridine (S9)

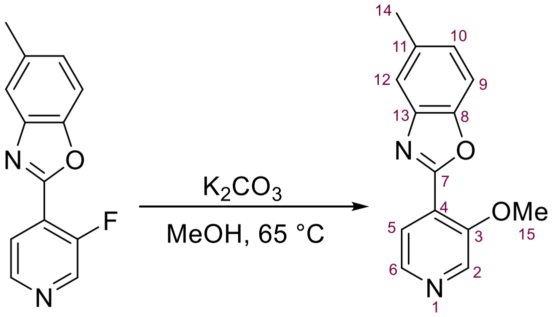

3-Fluoro-4-(4-methylbenzoxazole)pyridine (342 mg, 1.5 mmol) and potassium carbonate (414 mg, 3 mmol) were heated in MeOH (10 mL) for 2 hours at 65 °C. The solution was cooled and concentrated *in vacuo*. The residue was dissolved in EtOAc and water and extracted three times with EtOAc. The organic layers were combined, dried, filtered, and concentrated *in vacuo* to yield *pyridine* **S9** (285 mg, 79%) as a cream solid.

m.p. (acetone): 138-140 °C;

HRMS (ESI): Exact mass calculated for $C_{14}H_{13}O_2N_2$ $[M+H]^+$: 241.09715, found: 241.09715;

^1H NMR ($CDCl_3$) δ 8.57 (1H, s, C^2**H**), 8.43 (1H, d, *J* = 4.9 Hz, C^6**H**), 8.01 (1H, dd, *J* = 4.9, 0.6 Hz, C^5**H**), 7.65 (1H, dt, *J* = 1.7, 0.8 Hz, C^{12}**H**), 7.51 (1H, d, *J* = 8.3 Hz, C^9**H**), 7.23 (1H, ddd, *J* = 8.4, 1.7, 0.7 Hz, C^{10}**H**), 4.16 (3H, s, C^{15}**H**$_3$), 2.51 (3H, d, *J* = 0.6 Hz, C^{14}**H**$_3$);

^{13}C NMR ($CDCl_3$) δ 159.3 (C), 153.0 (C), 150.4 (C), 148.5 (C), 142.7 (C^6), 135.8 (C^2), 134.7 (C), 127.3 (C^9), 123.4 (C^5), 122.8 (C), 120.6 (C^{12}), 110.2 (C^{10}), 57.0 (C^{15}), 21.6 (C^{14});

IR (neat) (cm^{-1}): 2919, 2520, 2013, 1613, 1521, 1466, 1344, 1286, 1267, 1234, 1168, 1122, 1010, 933.

3-Benzoxy-4-(4-methylbenzoxazole)pyridine (S10)

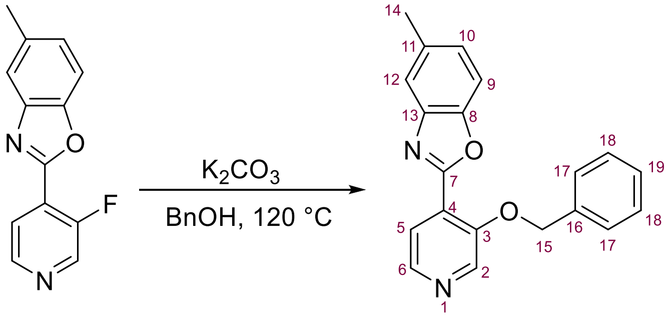

3-Fluoro-4-(4-methylbenzoxazole)pyridine (456 mg, 2 mmol) and potassium carbonate (1.0 g, 7.2 mmol) were heated in BnOH (2 mL) for 16 hours at 120 °C. The solution was cooled and concentrated *in vacuo*. The residue was dissolved in EtOAc and water and extracted three times with EtOAc. The organic layers were combined, dried, filtered, and concentrated *in vacuo*. The crude material was purified by FCC (30-60% EtOAc:pentane) to yield *pyridine* **S10** (427 mg, 68%) as a peach solid.

m.p. (acetone): 118-120 °C;

HRMS (ESI): Exact mass calculated for $C_{20}H_{17}O_2N_2$ $[M+H]^+$: 317.1285, found: 317.1284;

^1H NMR ($CDCl_3$) δ 8.58 (1H, s, C^2**H**), 8.42 (1H, d, *J* = 5.0 Hz, C^6**H**), 8.05 (1H, d, *J* = 5.0 Hz, C^5**H**), 7.64 (1H, dd, *J* = 1.7, 0.7 Hz, C^{12}**H**), 7.59 (2H, dq, *J* = 7.3, 0.7 Hz, 2 x C^{17}**H**), 7.49 (1H, d, *J* = 8.4 Hz, C^9**H**), 7.44-7.39 (2H, m, 2 x C^{18}**H**), 7.36-7.33 (1H, m, C^{19}**H**), 7.24 (1H, dd, *J* = 8.5, 1.7 Hz, C^{10}**H**), 5.42 (2H, s, C^{15}**H**$_2$), 2.52 (3H, s, C^{14}**H**$_3$);

^{13}C NMR ($CDCl_3$) δ 159.4 (C), 152.1 (C), 149.0 (C), 142.9 (C^6), 141.9 (C), 137.8 (C^2), 136.0 (C), 134.7 (C), 128.6 (2 x C^{18}), 128.1 (C^{19}), 127.2 (C^9), 127.0 (2 x C^{17}), 123.8 (C), 123.6 (C^5), 120.4 (C^{12}), 110.1 (C^{10}), 71.6 (C^{15}), 21.5 (C^{14});
IR (neat) (cm^{-1}) : 2923, 2161, 1524, 1497, 1414, 1384, 1313, 1281, 1225, 1173, 1061, 1026, 873, 841.

3-(3-Phenylpropyl)-4-(4-methylbenzoxazole)pyridine (S11)

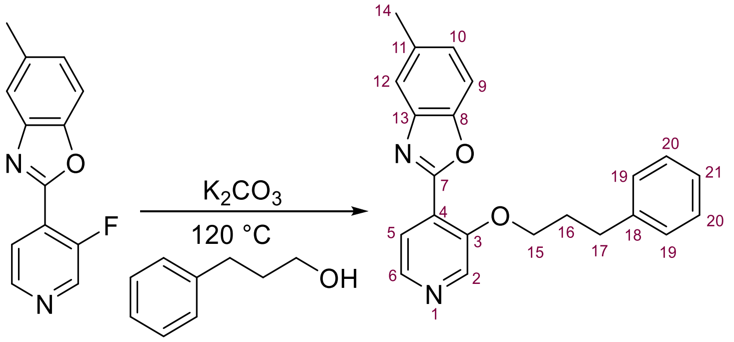

3-Fluoro-4-(4-methylbenzoxazole)pyridine (456 mg, 2 mmol) and potassium carbonate (1.0 g, 7.2 mmol) were heated in 3-phenylpropyano-1-ol (2 mL) for 16 hours at 120 °C. The solution was cooled and concentrated *in vacuo*. The residue was dissolved in EtOAc and water and extracted three times with EtOAc. The organic layers were combined, dried, filtered, and concentrated *in vacuo*. The crude material was purified by FCC (30-60% EtOAc:pentane) to yield *pyridine* **S11** (462 mg, 67%) as a cream solid.
m.p. (acetone): 93-95 °C;
HRMS (ESI): Exact mass calculated for $C_{22}H_{21}O_2N_2$ $[M+H]^+$: 345.1598, found: 345.1597;
^1H NMR ($CDCl_3$) δ 8.51 (1H, s, C^2**H**), 8.42 (1H, d, *J* = 5.0 Hz, C^6**H**), 8.04 (1H, d, *J* = C^5**H**), 7.65 (1H, dt, *J* = 1.7, 0.8 Hz, C^{12}**H**), 7.51 (1H, d, *J* = 8.3 Hz, C^9**H**), 7.34-7.20 (6H, m, C^{10}**H** + 2 x C^{19}**H** + 2 x C^{20}**H** + C^{21}**H**), 4.29 (2H, t, *J* = 6.1 Hz, C^{15}**H**$_2$), 2.99 (2H, dd, *J* = 8.4, 6.8 Hz, C^{17}**H**$_2$), 2.53 (3H, s, C^{14}**H**$_3$), 2.30-2.25 (2H, m, C^{16}**H**$_2$);
^{13}C NMR ($CDCl_3$) δ 159.7 (C), 152.6 (C), 149.0 (C), 142.4 (C^6), 141.9 (C), 141.3 (C), 136.9 (C^2), 134.7 (C), 128.53 (2 x $C^{19/20}$), 128.46 (2 x $C^{19/20}$), 127.1 ($C^{10/21}$), 126.0 ($C^{10/21}$), 123.6 (C^5), 123.3 (C), 120.4 (C^{12}), 110.0 (C^9), 68.6 (C^{15}), 31.9 (C^{17}), 31.0 (C^{16}), 21.5 (C^{14});
IR (neat) (cm^{-1}): 2953, 1563, 1522, 1481, 1456, 1385, 1312, 1198, 1062, 1029, 938, 928, 902, 874, 802.

3-(3-Methoxyphenyl)-4-(4-methylbenzoxazole)pyridine (S12)

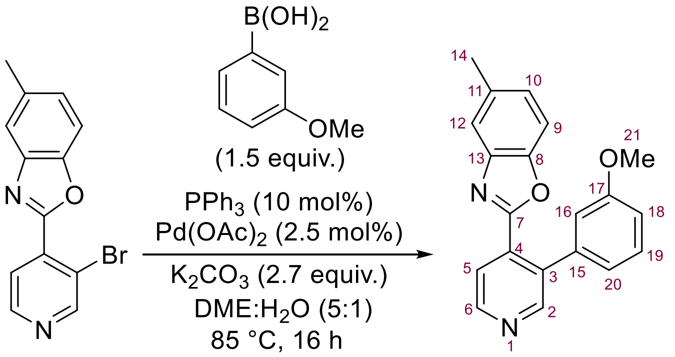

3-Bromo-4-(4-methylbenzoxazole)pyridine (636 mg, 2.2 mmol), potassium carbonate (819 mg, 75.94 mmol), triphenylphosphine (58 mg, 0.22 mmol), and 3-methoxybenzene boronic acid (502 mg, 3.3 mmol) were added to dimethoxyethane (10 mL) and water (2 mL) and the solution was purged with argon for 10 minutes. Palladium acetate (12.3 mg, 2.5 mol%) was added and the solution as heated at 85 °C for 16 hours. The solution was cooled and diluted with EtOAc (20 mL) and water (20 mL). The organic layer was separated, and the aqueous layer was washed with EtOAc (2 x 20 mL). The organic layers were combined, washed with brine (20 mL), dried ($MgSO_4$), filtered, and concentrated *in vacuo*. The residue was purified by FCC (10-40% EtOAc:pentane) to give *pyridine* **S12** (620 mg, 89%) as a highly viscous yellow oil.

HRMS (ESI): Exact mass calculated for $C_{20}H_{17}O_2N_2$ $[M+H]^+$: 317.12845, found: 317.12851;
^1H NMR ($CDCl_3$) δ 8.80-8.78 (2H, m, C^2**H** + C^6**H**), 8.04 (1H, dd, *J* = 5.1, 0.7 Hz, C^5**H**), 7.55 (1H, dt, *J* = 1.8, 0.8 Hz, C^{12}**H**), 7.34-7.28 (1H, m, C^{19}**H**), 7.25 (1H, d, *J* = 8.3 Hz, C^9**H**), 7.16 (1H, dd, *J* = 8.0, 1.5 Hz, C^{10}**H**), 6.99-6.94 (1H, d, *J* = 8.0 Hz, $C^{18/20}$**H**), 6.91-8.87 (2H, m, C^{16}**H** + $C^{18/20}$**H**), 3.76 (3H, s, C^{21}**H**$_3$), 2.48 (3H, s, C^{14}**H**$_3$);
^{13}C NMR ($CDCl_3$) δ 161.1 (C),159.5 (C), 151.7 (C^2), 149.0 (C), 148.7 (C^6), 141.6 (C), 138.5 (C), 136.2 (C), 134.7 (C), 133.4 (C), 129.3 (C^{19}), 127.1 (C^{10}), 123.6 (C^5), 121.6 (C^{16}), 120.4 (C^{12}), 114.5 ($C^{18/20}$), 113.9 ($C^{18/20}$), 110.2 (C^9), 55.2 (C^{21}), 21.4 (C^{14});
IR (neat) (cm^{-1}): 2939, 2833, 1466, 1428, 1322, 1258, 1176, 1019, 870, 844, 826, 796.

4-Benzothiazolepyridine (S13)

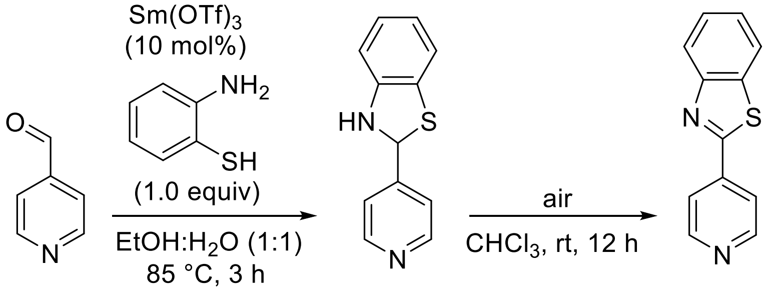

A mixture of 2-aminothiophenol (1.07 mL, 10 mmol), samarium(III) triflate (0.6 g, 1 mmol) and 4-pyridinecarboxaldehyde (0.94 mL, 10 mmol) in EtOH (20 mL) and water (20 mL) was heated at 85 °C for 3 hours. The mixture was cooled, extracted with Et_2O (3 x 50 mL), dried ($MgSO_4$), filtered, and concentrated *in vacuo*. NMR analysis of the crude material showed it was a 9:1 mixture of non-oxidised:oxidised compound. The crude material was dissolved in chloroform (50 mL) and stirred in an open flask for 12 hours. The resulting solid was purified by FCC (20-50% EtOAc:pentane) to give 4-benzothiazolepyridine **S13** (1.34 g, 63%) as a cream solid. The spectroscopic data matched previous literature reports.[6]

Isonicotinic Esters

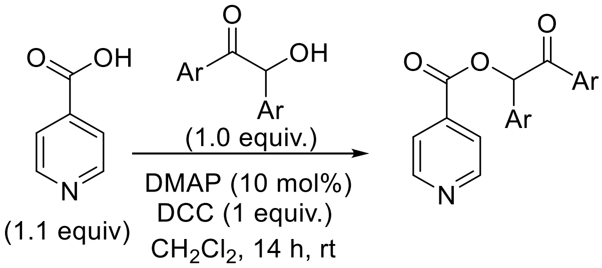

Appropriate Benzoin derivative (1.0 equiv), DMAP (10 mol%), and isonicotinic acid (1.1 equiv.) were suspended in CH_2Cl_2 (5 mL per 1 mmol). After 5 minutes, DCC (1.0 equiv.) was added and the suspension was stirred at room temperature overnight. The solution was diluted with CH_2Cl_2 (50 mL) and water (100 mL) and separated. The aqueous layer was extracted twice further (CH_2Cl_2 2 x 50 mL) and the organic layers were combined, dried ($MgSO_4$), filtered and concentrated *in vacuo*. The crude material was purified by FCC to give the corresponding isonicotinic ester.

[6] Huang, Y.; Zhou, P.; Wu, W.; Jiang, H. *J. Org. Chem.* **2018**, *83*, 2460-2466.

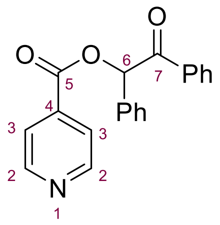

(2-Oxo-1,2-diphenylethyl) pyridine-4-carboxylate (S14)
The title compound was prepared from benzoin (2.12 g, 10 mmol) and was purified by FCC (10% to 50% EtOAc/pentane) to give *ester* **S14** (2.01 g, 63%) as a white solid.
m.p. (acetone): 154-156 °C;
HRMS (ESI): Exact mass calculated for $C_{20}H_{16}O_3N$ $[M+H]^+$: 318.11247, found: 318.11243;
^{1}H NMR ($CDCl_3$) δ 8.80 (2H, d, *J* = 6.1 Hz, 2 x C^{2}**H**), 8.01-7.97 (2H, m, 2 x Ar**H**), 7.93 (2H, d, *J* = 6.0 Hz, 2 x C^{3}**H**), 7.59-7.53 (3H, m, 3 x Ar**H**), 7.45-7.38 (5H, m, 5 x Ar**H**), 7.11 (1H, s, C^{6}**H**);
^{13}C NMR ($CDCl_3$) δ 192.9 (C^{7}), 164.6 (C^{5}), 150.7 (2 x C^{2}), 136.7 (C), 134.4 (C), 133.7 (C^{Ar}), 133.2 (C), 129.7 (C^{Ar}), 129.3 (2 x C^{Ar}), 128.83 (2 x C^{Ar}), 128.80 (2 x C^{Ar}), 128.75 (2 x C^{Ar}), 123.1 (2 x C^{3}), 78.7 (C^{6});
IR (neat) (cm^{-1}): 2163, 2033, 1727, 1697, 1597, 1495, 1410, 1359, 1282, 1244, 1230, 1178, 1091, 1063, 1002, 956, 933.

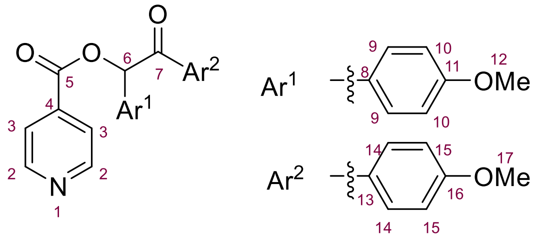

(2-Oxo-1,2-di(4-methoxyphenyl)ethyl) pyridine-4-carboxylate (S15)
The title compound was prepared from 4-methoxybenzoin (2.72 g, 10 mmol) and was purified by FCC (30% to 80% EtOAc/pentane) to give *ester* **S15** (2.06 g, 54%) as an orange gum.
HRMS (ESI): Exact mass calculated for $C_{22}H_{20}O_5N$ $[M+H]^+$: 378.1336, found: 378.1335;
^{1}H NMR ($CDCl_3$) δ 8.78 (2H, d, *J* = 6.1 Hz, 2 x C^{2}**H**), 7.96 (2H, d, *J* = 9.0 Hz, 2 x C^{14}**H**), 7.91 (2H, d, *J* = 6.1 Hz, 2 x C^{3}**H**), 7.47 (2H, d, *J* = 8.8 Hz, 2 x C^{9}**H**), 7.04 (1H, s, C^{6}**H**), 6.93 (2H, d, *J* = 8.8 Hz, 2 x C^{10}**H**), 6.89 (2H, d, *J* = 9.0 Hz, 2 x C^{15}**H**), 3.83 (3H, s, C^{17}**H**$_3$), 3.79 (3H, s, C^{12}**H**$_3$);
^{13}C NMR ($CDCl_3$) δ 191.2 (C^{7}), 164.7 (C^{5}), 163.8 (C^{11}), 160.5 (C^{16}), 150.5 (2 x C^{1}), 136.9 (C), 131.1 (2 x C^{14}), 130.2 (2 x C^{9}), 127.2 (C), 125.5 (C), 123.1 (2 x C^{3}), 114.7 (2 x C^{15}), 113.9 (2 x C^{10}), 78.1 (C^{6}), 55.4 (C^{17}), 55.3 (C^{12});
IR (neat) (cm^{-1}): 2935, 2161, 1727, 1685, 1598, 1574, 1461, 1408, 1326, 1247, 1170, 1064, 1029.

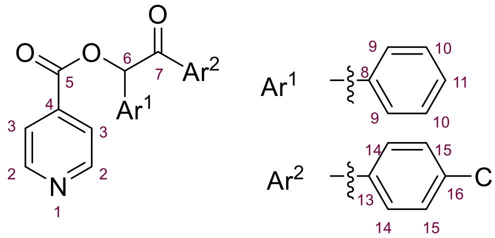

(2-Oxo-1-phenyl-2-(4-chlorophenyl)ethyl) pyridine-4-carboxylate (S16)
The title compound was prepared from 4-methoxybenzoin (2.47 g, 10 mmol) and was purified by FCC (20% to 50% EtOAc/pentane) to give *ester* **S16** (2.30 g, 66%) as a white solid.
m.p. (acetone): 138-140 °C;
HRMS (ESI): Exact mass calculated for $C_{20}H_{15}O_3NCl$ $[M+H]^+$: 352.0735, found: 352.0735;
^{1}H NMR ($CDCl_3$) δ 8.80 (2H, d, *J* = 6.1 Hz, 2 x C^{2}**H**), 7.94-7.90 (4H, m, 2 x C^{14}**H** + 2 x C^{3}**H**), 7.56-7.52 (2H, m, 2 x C^{10}**H**), 7.46-7.38 (5H, m, 2 x C^{9}**H** + C^{11}**H** + 2 x C^{15}**H**), 7.04 (1H, s, C^{6}**H**);
^{13}C NMR ($CDCl_3$) δ 191.7 (C^{7}), 164.6 (C^{5}), 150.6 (2 x C^{2}), 140.3 (C)¸136.5 (C), 132,8 (C), 132.6 (C), 130.2 (2 x C^{14}), 129.8 (C^{11}), 129.4 (2 x C$^{9/15}$), 129.1 (2 x C$^{9/15}$), 128.7 (2 x C^{10}), 123.1 (2 x C^{3}), 78.6 (C^{6});
IR (neat) (cm^{-1}): 2981, 2161, 1978, 1716, 1692, 1588, 1562, 1496, 1461, 1407, 1385, 1327, 1291, 1262, 1225, 1192.

4-Oxazolepyridine

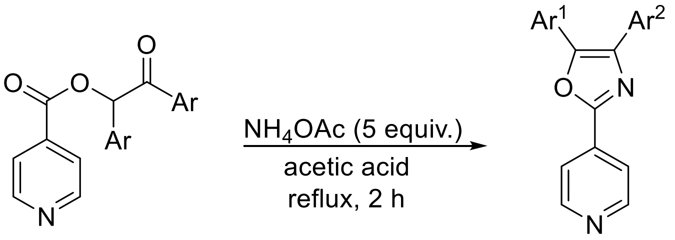

Isonicotinic ester (1 equiv.) and ammonium acetate (5 equiv.) were dissolved in glacial acetic acid (0.7 M) and heated at reflux for 2 hours. The reaction was cooled and diluted with H_2O (50 mL), basified with $NaOH_{(aq)}$ and then extracted with EtOAc (3 x 50 mL). The organic layers were combined, dried ($MgSO_4$), filtered, and concentrated *in vacuo*. The crude material was purified by FCC to give *pyridine*.

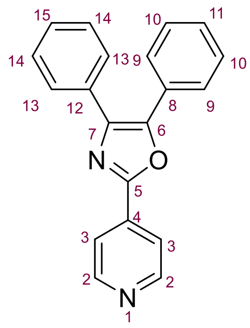

4-(4,5-Diphenyloxazole)pyridine (S17)

The title compound was prepared from **S14** (1.59 g, 5 mmol) and was purified by FCC (20-80% EtOAc/pentane) to give *pyridine* **S17** (330 mg, 22%) as a white solid which was 95% pure by ^{1}H NMR and was taken on without further purification.

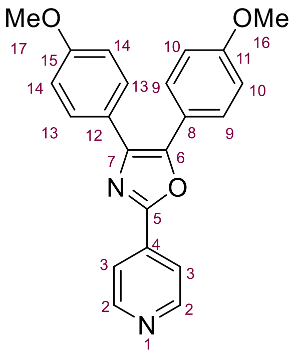

4-(4,5-Di(4-methoxyphenyl)oxazole)pyridine (S18)

The title compound was prepared from **S15** (1.89 g, 5 mmol) and was purified by FCC (50-70% EtOAc/pentane) to give *pyridine* **S18** (560 mg, 31%) as a peach solid which was 95% pure by ^{1}H NMR and taken onto the next step without further purification.

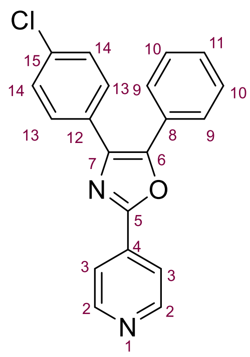

4-(4,(4-Chlorophenyl,-5-phenyl)oxazole)pyridine (S19)

The title compound was prepared from **S16** (1.75 g, 5 mmol) and was purified by FCC (20-50% EtOAc/pentane) to give *pyridine* **S19** (190 mg, 11%) as a cream solid which was 95% pure by ^{1}H NMR and was taken on without further purification.

(2-Methoxyphenyl)(pyridine-4-yl)methanone (S20)

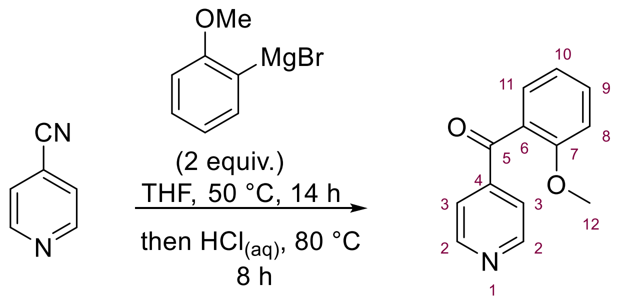

4-Cyanopyridine (2.08 g, 20 mmol) was dissolved in THF (10 mL) and cooled to 0 °C. 2-Methoxyphenylmagnesium bromide (40 mL, 1.0 M, 40 mmol) was slowly added and the solution was warmed to room temperature and then heated to 50 °C for 14 hours. The solution was cooled to 0 °C and H_2O (10 mL) was slowly added. The solution was evaporated *in vacuo* to remove the THF and 50 mL of 3M HCl was added, and the solution was heated at 80 °C for 8 hours. The solution was cooled to room temperature and the solution was washed with EtOAc. The aqueous phase was basified (NaOH) and extracted with CH_2Cl_2 (3 x 50 mL). The crude material was purified by FCC (0-50% EtOAc:pentane) to give *pyridine* **S20** (2.96 g, 69%) as a yellow oil.

HRMS (ESI): Exact mass calculated for $C_{13}H_{12}O_2N$ $[M+H]^+$: 214.08626, found: 214.08628;

^{1}H NMR ($CDCl_3$) δ 8.76 (2H, d, *J* = 6.0 Hz, 2 x C^2**H**), 7.57-7.51 (3H, m, 2 x C^3**H** + C$^{9/10}$**H**), 7.46 (1H, dd, *J* = 7.5, 1.6 Hz, C$^{8/11}$**H**), 7.08 (1H, td, *J* = 7.5, 1.0 Hz, C$^{9/10}$**H**), 7.00 (1H, d, *J* = 8.4 Hz, C$^{8/11}$**H**), 3.68 (3H, s, C^{12}**H**$_3$);

^{13}C NMR ($CDCl_3$) δ 195.4 (C^5), 157.9 (C)¸150.3 (2 x C^2), 144.7 (C)¸133.3 (C$^{9/10}$), 130.3 (C$^{8/11}$), 127.1 (C), 122.2 (2 x C^3), 120.8 (C$^{9/10}$), 111.5 (C$^{8/11}$), 55.4 (C^{12});

IR (neat) (cm^{-1}): 1666, 1598, 1556, 1486, 1462, 1436, 1407, 1326, 1297, 1244, 1159, 1111, 1048, 1020, 935, 841, 754, 694.

(2-Hydroxyphenyl)(pyridine-4-yl)methanone (S21)

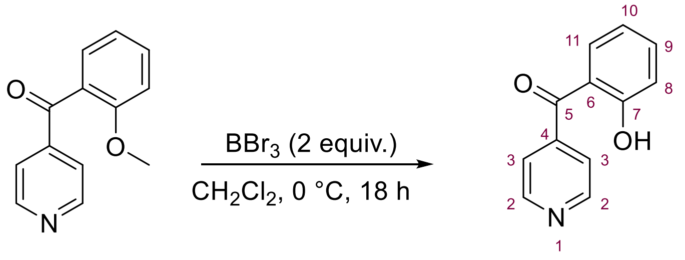

S20 (2.13 g, 10 mmol) was dissolved in CH_2Cl_2 (100 mL) and cooled to 0 °C, boron tribromide (20 mL, 20 mmol) was added dropwise and the reaction was stirred for 18 hours. The solution was added to ice and the pH was adjusted to 7 using sodium bicarbonate and stirred for 1 hour. The aqueous solution was extracted with CH_2Cl_2 (2 x 100 mL) and the organic layers were combined, dried ($MgSO_4$), filtered, and concentrated *in vacuo*. The crude material was purified by FCC (10-40% EtOAc:pentane) to give *pyridine* **S21** (1.59 g, 80%) as a yellow solid.

m.p. (acetone): 70-72 °C;

HRMS (ESI): Exact mass calculated for $C_{12}H_{10}O_2N$ $[M+H]^+$: 200.07061, found: 200.07067;

^{1}H NMR ($CDCl_3$) δ 11.8 (1H, s, O**H**), 8.83 (2H, d, *J* = 6.0 Hz, 2 x C^2**H**), 7.57 (1H, ddd, *J* = 8.7, 7.2, 1.7 Hz, C$^{9/10}$**H**), 7.50 (2H, d, *J* = 6.0 Hz, 2 x C^3**H**), 7.47 (1H, dd, *J* = 8.0, 1.7 Hz, C$^{8/11}$**H**), 7.10 (1H, dd, *J* = 8.4, 1.1 Hz, C$^{8/11}$**H**), 6.90 (1H, ddd, *J* = 8.2, 7.2, 1.1 Hz, C$^{9/10}$**H**);

^{13}C NMR ($CDCl_3$) δ 199.8 (C^5), 163.5 (C), 150.2 (2 x C^2), 144.7 (C), 137.6 (C$^{9/10}$), 133.1 (C$^{8/11}$), 122.2 (2 x C^3), 119.1 (C$^{9/10}$), 118.8 (C$^{8/11}$), 118.4 (C);

IR (neat) (cm^{-1}): 3040, 1634, 1614, 1574, 1543, 1485, 1455, 1405, 1318, 1253, 1196, 1149, 1066, 1035, 991, 941, 839, 815.

(2-Hydroxyphenyl)(pyridine-4-yl)methylamine (S22)

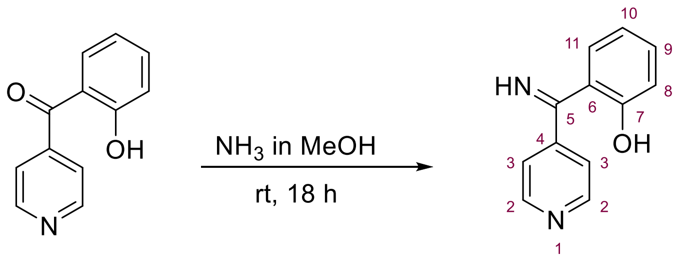

S21 (1.54 g, 7.54 mmol) was dissolved in 7N ammonia in methanol (10 mL) and stirred at room temperature for 2 hours. The solution was concentrated *in vacuo* to yield *imine* **S22** (1.41 g, 94%) as an orange solid.
m.p. (acetone): 128-130 °C;
HRMS (ESI): Exact mass calculated for $C_{12}H_{11}ON_2$ $[M+H]^+$: 199.08659, found: 199.08671;
^1H NMR ($CDCl_3$) δ 14.09 (1H, br s, O**H**), 9.50 (1H, br s, N**H**), 8.78 (2H, d, *J* = 6.0 Hz, 2 x C^2**H**), 7.39 (1H, ddd, *J* = 8.7, 7.2, 1.7 Hz, $C^{9/10}$**H**), 7.31 (2H, d, *J* = 6.0 Hz, 2 x C^3**H**), 7.08-7.02 (2H, m, 2 x $C^{8/11}$**H**), 6.77 (1H, ddd, *J* = 8.2, 7.2, 1.2 Hz, $C^{9/10}$**H**);
^{13}C NMR ($CDCl_3$) δ 179.0 (C^5), 163.0 (C), 150.5 (2 x C^2), 146.2 (C), 133.9 ($C^{9/10}$), 131.6 ($C^{8/11}$), 121.4 (2 x C^3), 118.4 ($C^{8/11}$), 118.1 ($C^{9/10}$), 117.5 (C);
IR (neat) (cm^{-1}): 3139, 1591, 1544, 1499, 1446, 1408, 1273, 1215, 1149, 1103, 1065, 921, 836, 816, 753, 634.

4-(Benzisoxazole)pyridine (S23)

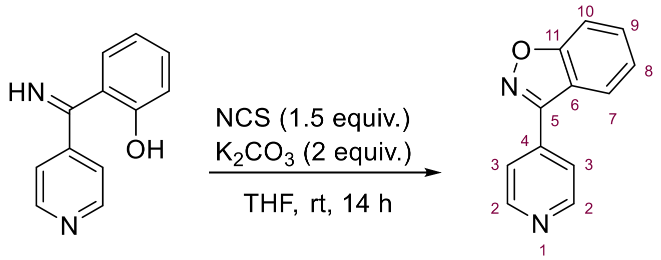

S22 (1.20 g, 6 mmol), potassium carbonate (1.66 g, 12 mmol), and *N*-chlorosuccinimide (1.2 g, 9 mmol) were suspended in THF (20 mL) and stirred at room temperature for 14 hours. The solution was quenched with H_2O (50 mL) and diethyl ether (50 mL). The solution was separated, and the aqueous layer was extracted with diethyl ether (2 x 50 mL). The organic layers were combined, dried ($MgSO_4$), filtered and concentrated *in vacuo*. The crude material was purified by FCC (10-30% EtOAc in pentane) to give *pyridine* **S23** (720 mg, 61%) as a yellow solid.
m.p. (acetone): 72-74 °C;
HRMS (ESI): Exact mass calculated for $C_{12}H_9ON_2$ $[M+H]^+$: 197.07094, found: 197.07100;
^1H NMR ($CDCl_3$) δ 8.85 (2H, d, *J* = 6.1 Hz, 2 x C^2**H**), 7.96 (1H, dt, *J* = 8.0 1.0 Hz, $C^{7/10}$**H**), 7.91 (2H, d, *J* = 6.1 Hz, 2 x C^3**H**), 7.72 (1H, dt, *J* = 8.5, 1.0 Hz, $C^{7/10}$**H**), 7.66 (1H, ddd, *J* = 8.4, 6.9, 1.2 Hz, $C^{8/9}$**H**), 7.45 (1H, ddd, *J* = 8.0, 6.9, 1.1 Hz, $C^{8/9}$**H**);
^{13}C NMR ($CDCl_3$) δ 164.2 (C), 155.2 (C), 150.6 (2 x C^2), 136.8 (C), 130.3 ($C^{8/9}$), 124.5 ($C^{8/9}$), 122.2 (2 x C^3), 121.6 ($C^{7/10}$), 119.7 (C), 110.5 ($C^{7/10}$);
IR (neat) (cm^{-1}): 3138, 1711, 1606, 1489, 1411, 1377, 1274, 1192, 905, 880, 826, 752, 705, 670, 654, 632.

Preparation of Pyridinium Salts

General Procedure B: Preparation of benzyl pyridinium iodide salts

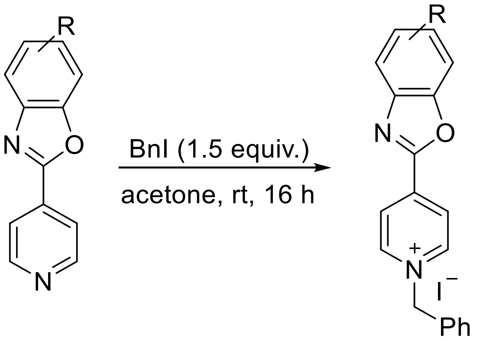

A mixture of the corresponding pyridine (1.00 equiv.) and benzyl iodide (1.50 equiv.) in acetone (10.0 mL) was stirred in the dark at room temperature for 16 hours. The solvent was removed under reduced pressure, followed by addition of acetone (5 mL) and diethyl ether (50 mL). The resulting suspension was sonicated (5 min) then filtered. The resultant solid was washed with diethyl ether and dried under vacuum for one hour to give the benzyl pyridinium iodide salts as crystalline solids.

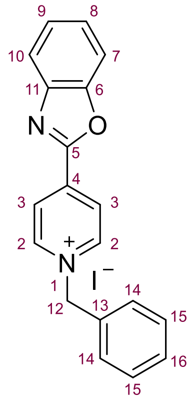

***N*-(Benzyl)-4-(2-benzoxazole)pyridinium Iodide (1a)** The title compound was prepared according to General Procedure B using 4-(benzoxazole)pyridine (655 mg, 3.3 mmol) to give salt **1a** (1.30 g, 95%) as a yellow solid.
m.p. (acetone): 268-270 °C;
HRMS (ESI): Exact mass calculated for $C_{19}H_{15}ON_2$ $[M]^+$: 287.11789, found: 287.11787;
^{1}H NMR ($(CD_3)_2SO$) δ 9.38 (2H, d, *J* = 6.9 Hz, 2 x C^2**H**), 8.81 (2H, d, *J* = 6.9 Hz, 2 x C^3**H**), 8.01 (1H, ddd, *J* = 8.0, 1.3, 0.7 Hz, C^7**H**), 7.95 (1H, dt, *J* = 8.3, 1.0 Hz, C^{10}**H**), 7.69-7.52 (4H, m, C^8**H** + C^9**H** + 2 x C^{15}**H**), 7.41-7.53 (3H, m, 2 x C^{14}**H** + C^{16}**H**), 5.96 (2H, s, 2 x C^{12}**H**$_2$);
^{13}C NMR ($(CD_3)_2SO$) δ 157.7 (C^4), 150.9 (C^5), 146.0 (2 x C^2), 141.2 (C), 140.9 (C), 134.1 (C), 129.6 (C^{16}), 129.4 (2 x C^{14}), 129.1 (2 x C^{15}), 128.4 (C^8), 126.2 (C^9), 125.4 (2 x C^3), 121.3 (C^7), 111.9 (C^{10}), 63.4 (C^{12});
IR (neat) (cm^{-1}): 3012, 1639, 1555, 1498, 1454, 1346, 1268, 1153, 1113, 1070, 1038, 933, 890, 851.

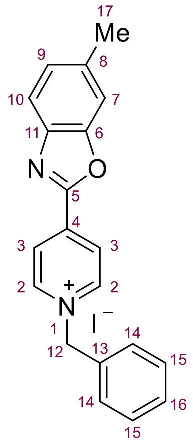

***N*-(Benzyl)-4-(5-methylbenzoxazole)pyridinium Iodide (1b)** The title compound was prepared according to General Procedure B using 4-(5-methylbenzoxazole)pyridine (420 mg, 2.0 mmol) to give salt **1b** (0.582 g, 68%) as an orange solid.
m.p. (acetone): 234-236 °C;
HRMS (ESI): Exact mass calculated for $C_{20}H_{17}ON_2$ $[M]^+$: 301.13354, found: 301.13345;
^{1}H NMR ($(CD_3)_2SO$) δ 9.36 (2H, d, *J* = 7.0 Hz, 2 x C^2**H**), 8.77 (2H, d, *J* = 6.9 Hz, 2 x C^3**H**), 7.88 (1H, d, *J* = 8.2 Hz, C^{10}**H**), 7.76 (1H, s, C^7**H**), 7.61-7.57 (2H, m, 2 x C^{15}**H**), 7.51-7.44 (3H, m, 2 x C^{14}**H** + C^{16}**H**), 7.39 (1H, dd, *J* = 8.3, 1.6 Hz, C^9**H**), 5.94 (2H, s, C^{12}**H**$_2$), 2.52 (3H, s, C^{17}**H**$_3$);
^{13}C NMR ($(CD_3)_2SO$) δ 157.1 (C^4), 151.3 (C^5), 145.9 (2 x C^2), 140.9 (C^6), 139.2 (C^{11}), 139.1 (C^{13}), 134.2 (C^8), 129.6 (C^{16}), 129.4 (2 x C^{14}), 129.0 (2 x C^{15}), 127.6 (C^9), 125.2 (2 x C^3), 120.7 (C^{10}), 111.6 (C^7), 63.3 (C^{12}), 21.6 (C^{17});
IR (neat) (cm^{-1}): 3033, 2162, 2034, 1637, 1480, 1456, 1391, 1346, 1320, 1208, 1180, 1113, 1093, 1010, 983.

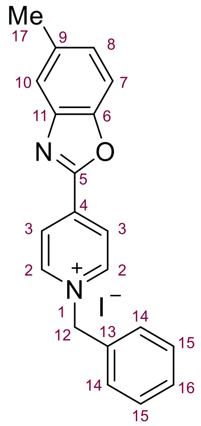

***N*-(Benzyl)-4-(4-methylbenzoxazole)pyridinium Iodide (1c)** The title compound was prepared according to General Procedure B using 4-(4-methylbenzoxazole)pyridine (420 mg, 2.0 mmol) to give salt **1c** (812 mg, 95%) as a red solid.

m.p. (acetone): 251-253 °C;

HRMS (ESI): Exact mass calculated for $C_{20}H_{17}ON_2$ $[M]^+$: 301.13354, found: 301.13353;

^{1}H NMR ($(CD_3)_2SO$) δ 9.36 (2H, d, J = 7.0 Hz, 2 x C^2**H**), 8.79 (2H, d, J = 7.0 Hz, 2 x C^3**H**), 7.83 (1H, d, J = 8.5 Hz, C^7**H**), 7.81-7.79 (1H, m, C^{10}**H**), 7.68-7.56 (2H, m, 2 x C^{15}**H**), 7.52-7.39 (4H, m, C^8**H** + 2 x C^{14}**H** + C^{16}**H**), 5.94 (2H, s, C^{12}**H**$_2$), 2.49 (3H, s, C^{17}**H**$_3$);

^{13}C NMR ($(CD_3)_2SO$) δ 157.7 (C), 149.2 (C), 145.9 (2 x C^2), 141.5 (C), 140.9 (C), 135.9 (C), 134.1 (C), 129.6 (C^7), 129.5 (C^{16}), 129.3 (2 x C^{14}), 129.0 (2 x C^{15}), 125.3 (2 x C^3), 120.8 (C^{10}), 111.3 (C^8), 63.3 (C^{12}), 21.0 (C^{17});

IR (neat) (cm^{-1}): 2997, 1635, 1547, 1494, 1343, 1289, 1215, 1149, 1067, 935, 859, 818, 798, 752, 737.

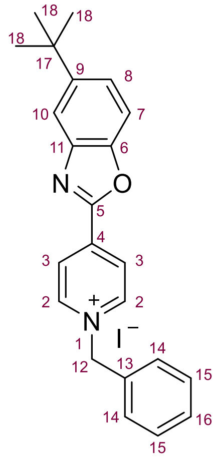

***N*-(Benzyl)-4-(4-*tert*-butylbenzoxazole)pyridinium Iodide (1d)** The title compound was prepared according to General Procedure B using 4-(4-*tert*-butylbenzoxazole)pyridine (504 mg, 2.0 mmol) to give salt **1d** (756 mg, 80%) as a red solid.

m.p. (acetone): 130-132 °C;

HRMS (ESI): Exact mass calculated for $C_{23}H_{23}ON_2$ $[M]^+$: 343.18049, found: 343.18048;

^{1}H NMR ($(CD_3)_2SO$) δ 9.37 (2H, d, J = 7.0 Hz, 2 x C^2**H**), 8.78 (2H, d, J = 7.0 Hz, 2 x C^3**H**), 7.94 (1H, dd, J = 1.9, 0.6 Hz, C^{10}**H**), 7.85 (1H, dd, J = 8.8, 0.6 Hz, C^7**H**), 7.71 (1H, dd, J = 8.8, 1.9 Hz, C^8**H**), 7.60 (2H, dt, J = 5.8, 1.7 Hz, 2 x C^{15}**H**), 7.51-7.44 (3H, m, C^{16}**H** + 2 x C^{14}**H**), 5.96 (2H, s, C^{12}**H**$_2$), 1.37 (9H, s, 3 x C^{18}**H**$_3$);

^{13}C NMR ($(CD_3)_2SO$) δ 157.7 (C), 149.3 (C), 149.1 (C), 146.0 (2 x C^2), 141.3 (C), 140.9 (C), 134.1 (C), 129.6 (C^{16}), 129.3 (2 x C^{14}), 129.1 (2 x C^{15}), 126.3 (C^8), 125.2 (2 x C^3), 117.3 (C^{10}), 111.1 (C^7), 63.3 (C^{12}), 34.9 (C^{17}), 31.4 (3 x C^{18});

IR (neat) (cm^{-1}): 3427, 2960, 2526, 2161, 2028, 1638, 1553, 1454, 1346, 1271, 1205, 1155, 1082, 1068, 934, 839, 818.

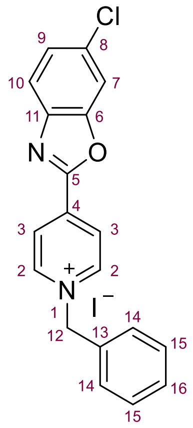

***N*-(Benzyl)-4-(5-chlorobenzoxazole)pyridinium Iodide (1e)** The title compound was prepared according to General Procedure B using 4-(5-chlorobenzoxazole)pyridine (460 mg, 2.0 mmol) to give salt **1e** (0.539 g, 60%) as a red solid.

m.p. (acetone): 201-203 °C;

HRMS (ESI): Exact mass calculated for $C_{19}H_{14}ON_2Cl$ $[M]^+$: 321.07892, found: 321.07904;

^{1}H NMR ($(CD_3)_2SO$) δ 9.38 (2H, d, J = 7.0 Hz, 2 x C^2**H**), 8.78 (2H, d, J = 6.9 Hz, 2 x C^3**H**), 8.21 (1H, dd, J = 2.0, 0.5 Hz, C^7**H**), 8.04 (1H, dd, J = 8.7, 0.5 Hz, C^9**H**), 7.67-7.55 (3H, m, C^{10}**H** + 2 x C^{15}**H**), 7.55-7.36 (3H, m, 2 x C^{14}**H** + C^{16}**H**), 5.95 (2H, s, C^{12}**H**$_2$);

^{13}C NMR ($(CD_3)_2SO$) δ 158.45 (C^4), 151.2 (C^5), 146.0 (2 x C^2), 140.6 (C^6), 140.2 (C^8), 134.0 (C^7), 132.5 (C^{11}), 129.5 (C^{13}), 129.3 (2 x C^{15}), 129.0 (2 x C^{14}), 126.7 (C^{16}), 125.4 (2 x C^3), 122.4 (C^9), 112.3 (C^{10}), 63.4 (C^{12});

IR (neat) (cm^{-1}): 3035, 2161, 1641, 1560, 1496, 1454, 1436, 1333, 1259, 1210, 1144, 1066, 1054, 931, 839, 824, 750.

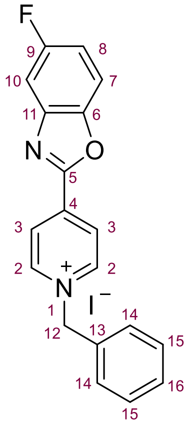

N-(Benzyl)-4-(6-fluorobenzoxazole)pyridinium Iodide (1f) The title compound was prepared according to General Procedure B using 4-(6-fluorobenzoxazole)pyridine (428 mg, 2.0 mmol) to give salt **1f** (0.680 g, 79%) as a red solid.
m.p. (acetone): 225-227 °C;
HRMS (ESI): Exact mass calculated for $C_{19}H_{14}ON_2F$ $[M]^+$: 305.10847, found: 305.10840;
^{1}H NMR ($(CD_3)_2SO$) δ 9.38 (2H, d, J = 7.0 Hz, 2 x C^2**H**), 8.77 (2H, d, J = 7.0 Hz, 2 x C^3**H**), 8.06 (1H, dd, J = 8.9, 5.0 Hz, C^7**H**), 7.99 (1H, dd, J = 8.4, 2.4 Hz, C^{10}**H**), 7.62-7.58 (2H, m, 2 x C^{15}**H**), 7.52-7.42 (4H, m, C^8**H** + 2 x C^{14}**H** + C^{16}**H**), 5.96 (2H, s, C^{12}**H**$_2$);
^{13}C NMR ($(CD_3)_2SO$) δ 161.6 (d, J = 245.7 Hz, C^9), 158.5 (d, J = 4.0 Hz, C^6), 151.1 (d, J = 15.4 Hz, C^{11}), 146.0 (2 x C^1), 140.6 (C), 137.9 (C), 134.1 (C), 129.6 (C^{16}), 129.3 (2 x C^{14}), 129.1 (2 x C^{15}), 125.3 (2 x C^3), 122.4 (d, J = 10.5 Hz, C^7), 114.5 (d, J = 25.3 Hz, C^8), 99.9 (d, J = 28.7 Hz, C^{10}), 63.4 (C^{12});
^{19}F NMR ($CDCl_3$) δ –110.5;
IR (neat) (cm^{-1}): 2981, 2161, 1637, 1483, 1455, 1347, 1287, 1152, 1110, 1064, 957, 844, 754, 737, 700, 616.

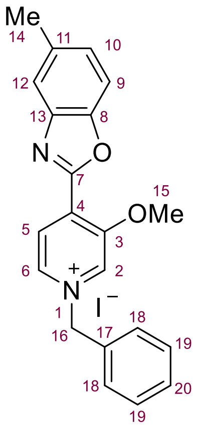

N-(Benzyl)-3-methoxy-4-(4-methylbenzoxazole)pyridinium Iodide (1g) The title compound was prepared according to General Procedure B using 3-methoxy-4-(4-methylbenzoxazole)pyridine (264 mg, 1.1 mmol) to give salt **1g** (453 mg, 90%) as an orange solid.
m.p. (acetone): 193-195 °C;
HRMS (ESI): Exact mass calculated for $C_{21}H_{19}O_2N_2$ $[M]^+$: 331.14410, found: 331.14398;
^{1}H NMR ($(CD_3)_2SO$) δ 9.44 (1H, d, J = 1.3 Hz, C^2**H**), 8.90 (1H, dd, J = 6.4, 1.3 Hz, C^6**H**), 8,70 (1H, d, J = 6.3 Hz, C^5**H**), 7.89-7.71 (2H, m, C^9**H** + C^{12}**H**), 7.68-7.58 (2H, m, 2 x C^{19}**H**), 7.53-7.44 (3H, m, C^{20}**H** + 2 x C^{18}**H**), 7.41 (1H, ddd, J = 8.4, 1.8, 0.7 Hz, C^{10}**H**), 5.94 (2H, s, C^{16}**H**$_2$), 4.24 (3H, s, C^{15}**H**$_3$), 2.47 (3H, s, C^{14}**H**$_3$);
^{13}C NMR ($(CD_3)_2SO$) δ 156.4 (C), 155.5 (C), 148.7 (C), 141.2 (C), 137.1 (C^6), 135.5 (C), 134.1 (C), 132.7 (C^2), 129.4 (C^{10}), 129.2 (2 x C^{18}), 129.1 (C^{20}), 128.8 (2 x C^{19}), 128.6 (C), 127.7 (C^5), 120.6 (C^{12}), 111.0 (C^9), 63.7 (C^{16}), 58.8 (C^{15}), 21.0 (C^{14});
IR (neat) (cm^{-1}): 2980, 2161, 1628, 1571, 1515, 1496, 1457, 1353, 1314, 1281, 1195, 1059, 1016, 947, 892, 799.

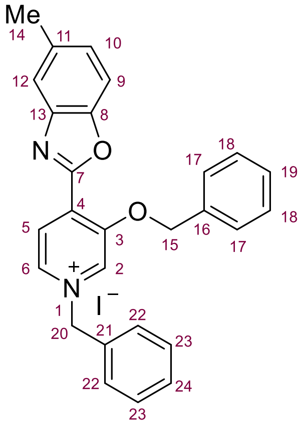

N-(Benzyl)-3-benzoxy-4-(4-methylbenzoxazole)pyridinium Iodide (1h) The title compound was prepared according to General Procedure B using 3-benzoxy-4-(4-methylbenzoxazole)pyridine (316 mg, 1.0 mmol) to give salt **1h** (463 mg, 87%) as an orange solid.
m.p. (acetone): 195-197 °C;
HRMS (ESI): Exact mass calculated for $C_{27}H_{23}O_2N_2$ $[M]^+$: 407.17540, found: 407.17514;
^{1}H NMR ($(CD_3)_2SO$) δ 9.53 (1H, s, C^2**H**), 8.95 (1H, d, J = 6.3 Hz, C^6**H**), 8.74 (1H, d, J = 6.3 Hz, C^5**H**), 7.78 (1H, s, C^{12}**H**), 7.74 (1H, d, J = 8.4 Hz, C^9**H**), 7.68-7.56 (4H, m, 2 x C^{18}**H** + 2 x C^{23}**H**), 7.52-7.38 (7H, m, C^{10}**H** + 2 x C^{17}**H** + C^{19}**H** + 2 x C^{22}**H** + C^{24}**H**), 5.93 (2H, s, C^{20}**H**$_2$), 5.65 (2H, s, C^{15}**H**$_2$), 2.48 (3H, s, C^{14}**H**$_3$);
^{13}C NMR ($(CD_3)_2SO$) δ 156.5 (C), 154.3 (C), 148.7 (C), 141.2 (C), 137.5 (C^6), 135.6 (C), 134.9 (C), 134.1 (C), 133.6 (C^2), 129.5 (C$^{19/24}$), 129.3 (C), 129.21 (2 x C$^{17/22}$), 129.16 (C$^{19/24}$), 128.8 (2 x C$^{17/22}$), 128.7 (2 x C$^{18/23}$), 128.4 (C), 127.8 (C^5), 127.2 (2 x C$^{18/23}$), 120.6 (C^{12}), 110.9 (C^9), 72.3 (C^{15}), 63.7 (C^{20}), 21.0 (C^{14});
IR (neat) (cm^{-1}): 2981, 2161, 2026, 1638, 1513, 1455, 1350, 1315, 1272, 1205, 1153, 1073, 1002, 850, 820, 806.

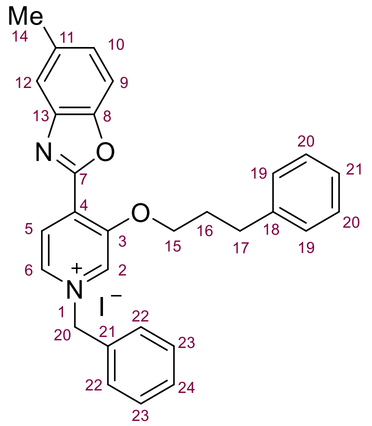

***N*-(Benzyl)-3-(3-phenylpropoxy)-4-(4-methylbenzoxazole)pyridinium Iodide (1i)** The title compound was prepared according to General Procedure B using 3-(3-phenylpropoxy)-4-(4-methylbenzoxazole)pyridine (344 mg, 1.0 mmol) to give salt **1i** (505 mg, 90%) as an orange solid.
m.p. (acetone): 210-212 °C;
HRMS (ESI): Exact mass calculated for $C_{29}H_{27}O_2N_2$ [M]$^+$: 435.20670 found: 435.20636;
^{1}H NMR ((CD$_3$)$_2$SO) δ 9.40 (1H, d, *J* = 1.3 Hz, C^{2}**H**), 8.90 (1H, dd, *J* = 6.4, 1.2 Hz, C^{6}**H**), 8.71 (1H, d, *J* = 6.3 Hz, C^{5}**H**), 7.78-7.73 (2H, m, C^{9}**H** + C^{12}**H**), 7.64-7.60 (2H, m, 2 x C^{23}**H**), 7.50-7.44 (3H, m, 2 x C^{22}**H** + C^{24}**H**), 7.43 (1H, dd, *J* = 8.4, 1.5 Hz, C^{10}**H**), 7.32-7.26 (4H, m, 2 x C^{19}**H** + 2 x C^{20}**H**), 7.19 (1H, ddd, *J* = 6.3, 4.7, 2.9 Hz, C^{21}**H**), 5.91 (2H, s, C^{20}**H**$_2$), 4.46 (2H, t, *J* = 6.0 Hz, C^{15}**H**$_2$), 2.93 (2H, dd, *J* = 8.6, 6.6 Hz, C^{17}**H**$_2$), 2.48 (3H, s, C^{14}**H**$_3$), 2.25-2.16 (2H, m, C^{16}**H**$_2$);
^{13}C NMR ((CD$_3$)$_2$SO) δ 156.6 (C), 154.7 (C), 148.8 (C), 141.14 (C), 141.10 (C), 137.1 (C^{6}), 135.6 (C), 134.1 (C), 133.0 (C^{2}), 129.4 (C^{10}), 129.2 (2 x C^{22}) 129.1 (C^{24}), 128.9 (C^{11}), 128.7 (2 x C^{23}), 128.4 (2 x C^{19} + 2 x C^{20}), 127.7 (C^{5}), 125.9 (C^{21}), 120.5 (C^{12}), 110.8 (C^{9}), 70.4 (C^{15}), 63.7 (C^{20}), 31.1 (C^{17}), 30.1 (C^{16}), 21.0 (C^{14});
IR (neat) (cm^{-1}): 2972, 1573, 1495, 1470, 1454, 1344, 1318, 1287, 1197, 1058, 1018, 928, 818, 791, 729, 701.

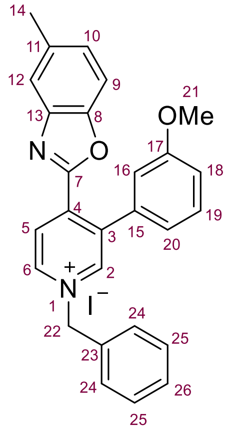

***N*-(Benzyl)-3-(3-methoxyphenyl)-4-(4-methylbenzoxazole)pyridinium Iodide (1j)** The title compound was prepared according to General Procedure B using 3-(3-methoxyphenyl)-4-(4-methylbenzoxazole)pyridine (590 mg, 1.86 mmol) to give salt **1j** (700 mg, 70%) as a yellow solid.
m.p. (acetone): 115-117 °C;
HRMS (ESI): Exact mass calculated for $C_{27}H_{23}O_2N_2$ [M]$^+$: 407.17540, found: 407.17523;
^{1}H NMR ((CD$_3$)$_2$SO) δ 9.59 (1H, d, *J* = 1.5 Hz, C^{2}**H**), 9.29 (1H, dd, *J* = 6.5, 1.4 Hz, C^{6}**H**), 8.81 (1H, d, *J* = 6.5 Hz, C^{5}**H**), 7.69-7.65 (3H, m, C^{9}**H** + C^{12}**H** + C^{26}**H**), 7.55-7.40 (5H, m, C^{19}**H** + 2 x C^{24}**H** + 2 x C^{25}**H**), 7.34 (1H, dd, *J* = 8.5, 1.7 Hz, C^{10}**H**), 7.20-7.13 (2H, m, C^{16}**H**+ C$^{18/20}$**H**), 7.05 (1H, dt, *J* = 7.8, 1.3 Hz, C$^{18/20}$**H**), 5.97 (2H, s, C^{22}**H**$_2$), 3.76 (3H, s, C^{21}**H**$_3$), 2.44 (3H, s, C^{14}**H**$_2$);
^{13}C NMR ((CD$_3$)$_2$SO) δ 159.7 (C), 158.2 (C), 149.1 (C), 147.6 (C^{2}), 143.7 (C^{6}), 141.4 (C), 139.9 (C), 139.8 (C), 136.0 (C), 135.4 (C), 134.4 (C), 130.2 (C$^{10/19/26}$), 130.0 (C$^{10/19/26}$), 129.7 (2 x C$^{24/25}$), 129.6 (2 x C$^{24/25}$), 129.5 (C$^{10/19/26}$), 128.8 (C^{5}), 122.2 (C$^{18/20}$), 121.1 (C^{12}), 115.62 (C^{16}), 115.58 (C$^{18/20}$), 111.3 (C^{9}), 63.8 (C^{22}), 55.8 (C^{21}), 21.4 (C^{14});
IR (neat) (cm^{-1}): 1706, 1633, 1610, 1581, 1484, 1422, 1360, 1287, 1223, 1146, 1045, 911, 808, 785, 752, 714.

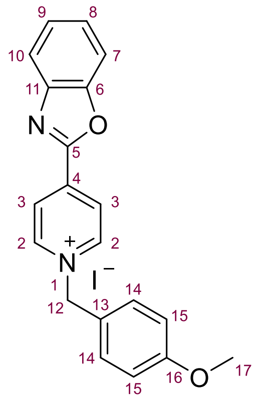

***N*-(*para*-Methoxybenzyl)-4-(benzoxazole)pyridinium Iodide (1k)** The title compound was prepared according to General Procedure B using 4-(benzoxazole)pyridine (392 mg, 2.0 mmol) and *para*-methoxybenzyl iodide (600 mg, 2.4 equiv.) to give salt **1k** (0.891 g, 99%) as an orange solid.
m.p. (acetone): 220-222 °C;
HRMS (ESI): Exact mass calculated for $C_{20}H_{17}O_2N_2$ [M]$^+$: 317.12845, found: 317.12836;
^{1}H NMR ((CD$_3$)$_2$SO) δ 9.35 (2H, d, *J* = 7.0 Hz, 2 x C^{2}**H**), 8.78 (2H, d, *J* = 6.9 Hz, 2 x C^{3}**H**), 8.00 (1H, ddd, *J* = 8.0, 1.3, 0.7 Hz, C$^{7/10}$**H**), 7.94 (1H, dt, *J* = 8.3, 1.0 Hz, C$^{7/10}$**H**), 7.72-7.42 (4H, m, C^{8}**H** + C^{9}**H** + 2 x C^{14}**H**), 7.04 (2H, d, *J* = 8.8 Hz, 2 x C^{15}**H**), 5.88 (2H, s, C^{12}**H**$_2$), 3.77 (3H, s, C^{17}**H**$_3$);

^{13}C NMR (($(CD_3)_2SO$) δ 160.2 (C), 157.7 (C), 150.9 (C), 145.7 (2 x C^{2}), 141.2 (C), 140.7 (C), 131.0 (2 x C^{14}), 128.3 ($C^{8/9}$), 126.2 ($C^{8/9}$), 125.8 (C), 125.3 (2 x C^{3}), 121.3 ($C^{7/10}$), 114.7 (2 x C^{15}), 111.9 ($C^{7/10}$), 63.1 (C^{12}), 55.4 (C^{17});
IR (neat) (cm^{-1}): 2980, 1709, 1638, 1606, 1514, 1462, 1353, 1249, 1183, 1147, 1066, 1022, 847, 817, 790, 756.

***N*-(*n*-Butyl)-4-(benzoxazole)pyridinium Iodide (1l)**

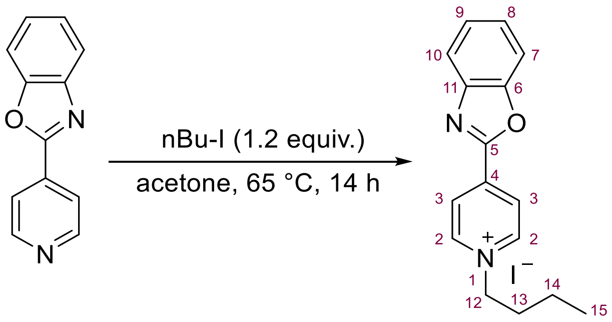

4-(Benzoxazole)pyridine (392 mg, 2.0 mmol) and *n*-butyl iodide (442 mg, 2.4 mmol) were dissolved in acetone (5 mL) and heated to 65 °C for 14 hours. The solution was cooled, added to diethyl ether (100 mL) and sonicated for 10 minutes, before being filtered. The solids were washed with diethyl ether to give *salt* **1l** (0.571 g, 75%) as an orange solid
m.p. (acetone): 240-242 °C;
HRMS (ESI): Exact mass calculated for $C_{16}H_{17}ON_2$ $[M]^+$: 253.13354, found: 253.13356;
^{1}H NMR (($(CD_3)_2SO$) δ 9.30 (2H, d, *J* = 6.9 Hz, 2 x C^{2}**H**), 8.80 (2H, d, *J* = 6.9 Hz, 2 x C^{3}**H**), 8.02 (1H, d, *J* = 7.4 Hz, $C^{7/10}$**H**), 7.96 (1H, d, *J* = 8.1 Hz, $C^{7/10}$**H**), 7.65 (1H, ddd, *J* = 8.4, 7.4, 1.3 Hz, $C^{8/9}$**H**), 7.57 (1H, td, *J* = 7.7, 1.1 Hz, $C^{8/9}$**H**), 4.70 (2H, t, *J* = 7.4 Hz, C^{12}**H**$_2$), 1.95 (2H, dd, *J* = 8.6, 6.3 Hz, C^{13}**H**$_2$), 1.34 (2H, app h, *J* = 7.4 Hz, C^{14}**H**$_2$), 0.94 (3H, t, *J* = 7.4 Hz, C^{15}**H**$_2$);
^{13}C NMR (($(CD_3)_2SO$) δ 157.7 (C), 150.9 (C), 146.0 (2 x C^{2}), 141.2 (C), 140.4 (C), 128.3 ($C^{8/9}$), 126.2 ($C^{8/9}$), 125.0 (2 x C^{3}), 121.3 ($C^{7/10}$), 111.8 ($C^{7/10}$), 60.7 (C^{12}). 32.7 (C^{13}), 18.8 (C^{14}), 13.4 (C^{15});
IR (neat) (cm^{-1}): 2927, 1644, 1552, 1505, 1458, 1350, 1294, 1214, 1167, 1113, 1068, 934, 891, 858, 820, 773.

***N*-(3-Tosylpropyl)-4-(benzoxazole)pyridinium Tosylate (1m)**

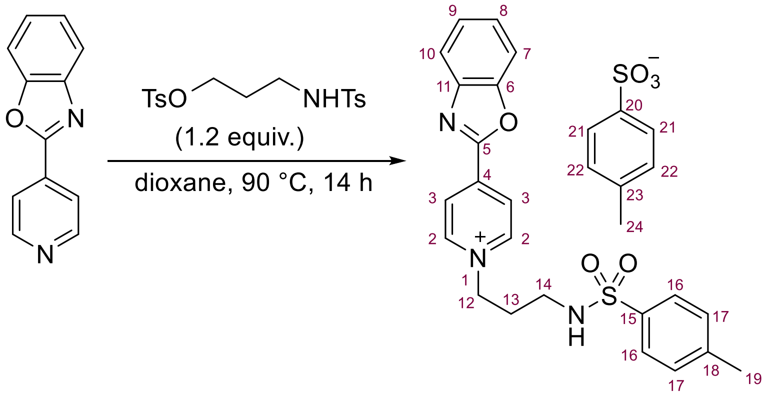

4-(Benzoxazole)pyridine (392 mg, 2.0 mmol) and *N*-tosyl-3-tosylate-1-propylamine (919 mg, 2.4 mmol) were dissolved in dioxane (5 mL) and heated to 90 °C for 14 hours. The solution was cooled, added to diethyl ether (100 mL) and sonicated for 10 minutes, before being filtered. The solids were washed with diethyl ether to give *salt* **1m** (868 mg, 75%) as a beige solid.
m.p. (acetone): 138-140 °C;
HRMS (ESI): Exact mass calculated for $C_{22}H_{22}O_3N_3S$ $[M]^+$: 408.1376, found: 408.1375;
^{1}H NMR (($(CD_3)_2SO$) δ 9.22 (2H, d, *J* = 7.0 Hz, 2 x C^{2}**H**), 8.77 (2H, d, *J* = 6.9 Hz, 2 x C^{3}**H**), 8.02 (1H, ddd, *J* = 7.9, 1.3, 0.7 Hz, $C^{7/10}$**H**), 7.96 (1H, dt, *J* = 8.2, 0.9 Hz, $C^{7/10}$**H**), 7.71-7.63 (4H, m, N**H** + $C^{8/9}$**H** + 2 x C^{16}**H**),

7.57 (1H, td, *J* = 7.8, 1.1 Hz, $C^{8/9}$**H**), 7.47 (2H, d, *J* = 8.0 Hz, 2 x C^{21}**H**), 7.40 (2H, d, *J* = 7.8 Hz, 2 x C^{17}**H**), 7.10 (2H, d, *J* = 7.8 Hz, 2 x C^{22}**H**), 4.71 (2H, t, *J* = 7.2 Hz, C^{12}**H**$_2$), 2.81 (2H, q, *J* = 6.5 Hz, C^{14}**H**$_2$), 2.38 (3H, s, C^{19}**H**$_3$), 2.27 (3H, s, C^{24}**H**$_3$), 2.12 (2H, p, *J* = 7.0 Hz, C^{13}**H**$_2$);
^{13}C NMR ($(CD_3)_2SO$) δ 158.1 (C), 151.4 (C), 146.6 (2 x C^2), 146.3 (C), 143.3 (C), 141.7 (C), 140.9 (C), 138.0 (C), 137.5 (C), 130.2 (2 x C^{17}), 128.8 ($C^{8/9}$), 128.5 (2 x C^{22}), 127.0 (2 x C^{16}), 126.7 ($C^{8/9}$), 126.0 (2 x C^{21}), 125.5 (2 x C^3), 121.8 ($C^{7/10}$), 112.3 ($C^{7/10}$), 59.1 (C^{12}), 31.0 (C^{13}), 21.4 (C^{19}), 21.2 (C^{24}), *C^{14} is approximately 40 ppm but its exact position is masked by the DMSO peak as shown below in the HSQC spectra.*

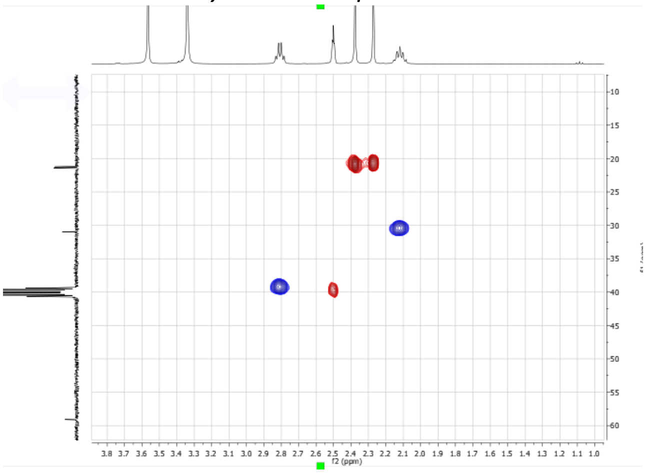

IR (neat) (cm^{-1}): 3400, 3079, 2934, 2162, 1645, 1540, 1405, 1351, 1323, 1221, 1173, 1162, 1117, 1067, 1030, 1008.

***N*-(2-Ethan-1-ol)-4-(benzoxazole)pyridinium Iodide (1n)**

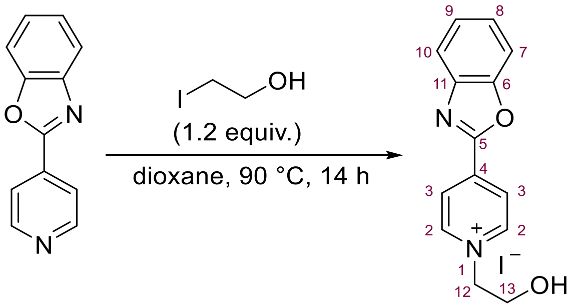

4-(Benzoxazole)pyridine (392 mg, 2.0 mmol) and 2-iodothanol (413 mL, 2.4 mmol) were dissolved in dioxane (5 mL) and heated to 90 °C for 14 hours. The solution was cooled, added to diethyl ether (100 mL) and sonicated for 10 minutes, before being filtered. The solids were washed with diethyl ether to give *salt* **1n** (614 mg, 83%) as a yellow solid.
m.p. (acetone): 256-258 °C;
HRMS (ESI): Exact mass calculated for $C_{14}H_{13}O_2N_2$ $[M]^+$: 241.09715, found: 241.09722;
^1H NMR ($(CD_3)_2SO$) δ 9.21 (2H, d, *J* = 7.0 Hz, 2 x C^2**H**), 8.80 (2H, d, *J* = 6.9 Hz, 2 x C^3**H**), 8.01 (1H, ddd, *J* = 7.9, 1.2, 0.8 Hz, $C^{7/10}$**H**), 7.96 (1H, dt, *J* = 8.3, 0.9 Hz, $C^{7/10}$**H**), 7.67-7.62 (1H, m, $C^{8/9}$**H**), 7.59-7.54 (1H, m, $C^{8/9}$**H**), 5.31 (1H, t, *J* = 5.2 Hz, O**H**), 4.77 (2H, t, *J* = 5.0 Hz, C^{12}**H**$_2$), 3.92 (2H, q, *J* = 4.9 Hz, C^{13}**H**$_2$);
^{13}C NMR ($(CD_3)_2SO$) δ 157.7 (C), 150.9 (C), 146.4 (2 x C^2), 141.2 (C), 140.5 (C)¸128.2 ($C^{8/9}$), 126.1 ($C^{8/9}$), 124.6 (2 x C^3), 121.3 ($C^{7/10}$), 11.8 ($C^{7/10}$), 63.3 (C^{12}), 60.0 (C^{13});
IR (neat) (cm^{-1}): 3249, 2161, 1641, 1554, 1502, 1462, 1437, 1372, 1350, 1333, 1249, 1195, 1171, 1112, 1070, 951.

N-(Ethyl pentanoate)-4-(benzoxazole)pyridinium Iodide (1o)

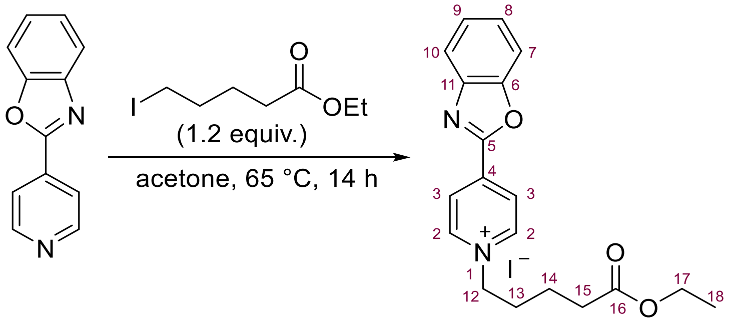

4-(Benzoxazole)pyridine (392 mg, 2.0 mmol) and ethyl 5-iodopentanoate (614 mg, 2.4 mmol) were dissolved in acetone (5 mL) and heated to 65 °C for 14 hours. The solution was cooled, added to diethyl ether (100 mL) and sonicated for 10 minutes, before being filtered. The solids were washed with diethyl ether to give *salt* **1o** (338 mg, 37%) as a hydroscopic yellow solid.
m.p. (acetone): 180-182 °C;
HRMS (ESI): Exact mass calculated for $C_{19}H_{21}O_3N_2$ $[M]^+$: 325.1547, found: 325.1545;
^{1}H NMR ($(CD_3)_2SO$) δ 9.29 (2H, d, *J* = 7.0 Hz, 2 x C^2**H**), 8.81 (2H, d, *J* = 6.9 Hz, 2 x C^3**H**), 8.02 (1H, dt, *J* = 7.9, 1.1 Hz, C$^{7/10}$**H**), 7.96 (1H, dt, *J* = 8.3, 0.9 Hz, C$^{7/10}$**H**), 7.65 (1H, ddd, *J* = 8.3, 7.3, 1.3 Hz, C$^{8/9}$**H**), 7.57 (1H, ddd, *J* = 8.4, 7.4, 1.1 Hz, C$^{8/9}$**H**), 4,71 (2H, t, *J* = 7.3 Hz, C^{12}**H**$_2$), 4.05 (2H, q, *J* = 7.1 Hz, C^{17}**H**$_2$), 2.39 (2H, t, *J* = 7.4 Hz, C^{15}**H**$_2$), 1.99 (2H, dq, *J* = 9.7, 7.3 Hz, C^{13}**H**$_2$), 1.57 (2H, dq, *J* = 10.6, 7.5 Hz, C^{14}**H**$_2$), 1.17 (3H, t, *J* = 7.1 Hz, C^{18}**H**$_3$);
^{13}C NMR ($(CD_3)_2SO$) δ 172.5 (C^{16}), 157.7 (C), 150.9 (C), 146.0 (2 x C^2), 141.2 (C), 140.5 (C), 128.3 (C$^{8/9}$), 126.2 (C$^{8/9}$), 125.1 (2 x C^3), 121.3 (C$^{7/10}$), 111.8 (C$^{7/10}$), 60.5 (C^{12}), 59.9 (C^{17}), 32.7 (C^{15}), 30.1 (C^{13}), 20.9 (C^{14}), 14.2 (C^{18});
IR (neat) (cm^{-1}): 3492, 2980, 2161, 1978, 1722, 1647, 1552, 1507, 1469, 1375, 1351, 1300, 1238, 1197, 1160, 1113.

N-(Ethyl pentanoate)-4-(benzoxazole)pyridinium Iodide (1p)

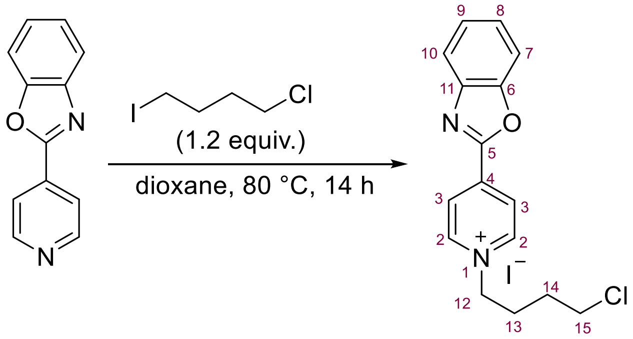

4-(Benzoxazole)pyridine (392 mg, 2.0 mmol) and 4-chloro-1-iodobutane (524 mg, 2.4 mmol) were dissolved in dioxane (5 mL) and heated to 80 °C for 14 hours. The solution was cooled, added to diethyl ether (100 mL) and sonicated for 10 minutes, before being filtered. The solids were washed with diethyl ether to give *salt* **1p** (691 mg, 83%) as an orange solid.
m.p. (acetone): 216-218 °C;
HRMS (ESI): Exact mass calculated for $C_{16}H_{16}ON_2Cl$ $[M]^+$: 287.09457, found: 287.09454;
^{1} H NMR ($(CD_3)_2SO$) δ 9.30 (2H, d, *J* = 5.5 Hz, 2 x C^2**H**), 8.81 (2H, d, *J* = 5.4 Hz, 2 x C^3**H**), 8.02 (1H, d, *J* = 8.0 Hz, C^{10}**H**), 7.96 (1H, d, *J* = 8.2 Hz, C^7**H**), 7.65 (1H, tt, *J* = 7.9, 1.6 Hz, C$^{8/9}$**H**), 7.59-7.54 (1H, m, C$^{8/9}$**H**), 4.75 (2H, t, *J* = 7.4 Hz, C^{12}**H**$_2$), 3.71 (2H, td, *J* = 6.5, 1.4 Hz, C^{15}**H**$_2$), 2.12-2.04 (2H, m, C^{13}**H**$_2$), 1.84-1.74 (2H, m, C^{14}**H**$_2$);
^{13}C NMR ($(CD_3)_2SO$) δ 157.7 (C), 150.9 (C), 146.0 (2 x C^2), 141.2 (C), 140.5 (C), 128.3 (C$^{8/9}$), 126.2 (C$^{8/9}$), 125.1 (2 x C^3), 121.3 (C^{10}), 111.8 (C^7), 60.2 (C^{12}), 44.6 (C^{15}), 28.5 (C^{14}), 28.3 (C^{13});
IR (neat) (cm^{-1}): 2981, 2160, 2024, 1637, 1553, 1502, 1455, 1350, 1298, 1210, 1172, 1110, 1073, 936, 857, 822.

***N*-Benzyl-4-(benzothiazole)pyridin-1-ium Iodide (1q)** The title compound was prepared according to

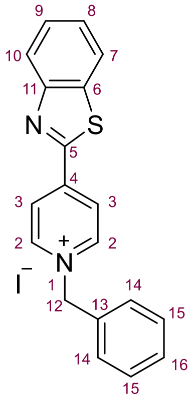

General Procedure B using 4-(benzothiazole)pyridine (424 mg, 2.0 mmol) to give salt **1q** (790 mg, 92%) as an yellow solid.

m.p. (acetone): 242-244 °C;

HRMS (ESI): Exact mass calculated for $C_{19}H_{15}N_2S$ [M]$^+$: 303.09505, found: 303.09508;

^{1}H NMR ((CD_3)$_2$SO) δ 9.33 (2H, d, *J* = 7.0 Hz, 2 x C^{2}**H**), 8.78 (2H, d, *J* = 6.9 Hz, 2 x C^{3}**H**), 8.35 (1H, dd, *J* = 7.6, 1.7 Hz, C$^{7/10}$**H**), 8.25 (1H, dd, *J* = 7.8, 1.7 Hz, C$^{7/10}$**H**), 7.71-7.67 (1H, m, C$^{8/9}$**H**), 7.66-7.62 (1H, m, C$^{8/9}$**H**), 7.61-7.58 (2H, m, 2 x C^{15}**H**), 7.51-7.42 (3H, m, 2 x C^{14}**H** + C^{16}**H**), 5.95 (2H, s, C^{12}**H$_2$**);

^{13}C NMR ((CD_3)$_2$SO) δ 161.3 (C)¸153.3 (C), 146.7 (C), 145.8 (2 x C^{2}),136.2 (C), 134.1 (C), 129.4 (C^{16}), 129.3 (2 x C^{14}), 128.9 (2 x C^{15}), 127.83 (C$^{8/9}$), 127.76 (C$^{8/9}$), 125.3 (2 x C^{3}), 124.3 (C$^{7/10}$), 123.2 (C$^{7/10}$), 63.1 (C^{12});

IR (neat) (cm^{-1}): 3017, 1635, 1562, 1519, 1485, 1445, 1314, 1256, 1154, 1129, 1077, 1038, 984, 921, 850, 830.

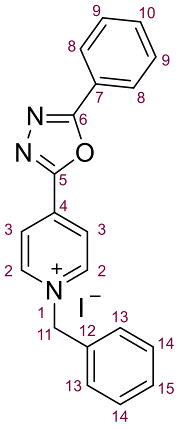

***N*-(Benzyl)-4-(5-phenyl-1,3,4-oxadiazol-2-yl)pyridinium Iodide (1r)** The title compound was prepared according to General Procedure B using 4-(5-phenyl-1,3,4-oxadiazol-2-yl)pyridine[7] (446 mg, 2.0 mmol) to give salt **1r** (568 mg, 64%) as a yellow solid.

m.p. (acetone): 209-211 °C;

HRMS (ESI): Exact mass calculated for $C_{20}H_{16}ON_3$ [M]$^+$: 314.1288, found: 314.1288;

^{1}H NMR ((CD_3)$_2$SO) δ 9.45 (2H, d, *J* = 7.0 Hz, 2 x C^{2}**H**), 8.83 (2H, d, *J* = 7.0 Hz, 2 x C^{3}**H**), 8.24 (2H, dt, *J* = 6.9, 1.6 Hz, 2 x C^{8}**H**), 7.79-7.65 (3H, m, 2 x C^{9}**H** + C^{10}**H**), 7.64-7.56 (2H, m, 2 x C^{14}**H**), 7.54-7.41 (3H, m, 2 x C^{13}**H** + C^{15}**H**), 5.97 (2H, s, 2 x C^{11}**H$_2$**);

^{13}C NMR ((CD_3)$_2$SO) δ 166.0 (C^{4}), 160.9 (C^{5}), 146.2 (2 x C^{2}H), 137.9 (C), 134.1 (C), 133.1 (C^{10}H), 129.7 (2 x C^{9}H), 129.5 (C^{15}H), 129.3 (2 x C^{13}H), 128.9 (2 x C^{14}H), 127.3 (2 x C^{8}H), 124.9 (2 x C^{3}H), 122.5 (C), 63.5 (C^{11}H$_2$);

IR (neat) (cm^{-1}): 3416, 3056, 2161, 2033, 1644, 1606, 1545, 1496, 1479, 1456, 1327, 1256, 1207, 1154, 1128, 1070.

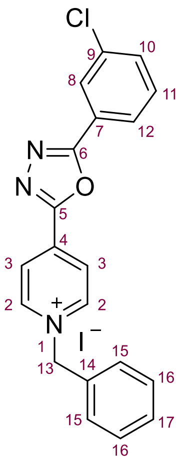

***N*-(Benzyl)-4-(5-(3-chlorophenyl)-1,3,4-oxadiazol-2-yl)pyridinium Iodide (1s)** The title compound was prepared according to General Procedure B using 4-(5-(3-chlorophenyl)-1,3,4-oxadiazol-2-yl)pyridine[8] (475 mg, 1.73 mmol) to give salt **1s** (340 mg, 41%) as an orange solid.

m.p. (acetone): 230-232 °C;

HRMS (ESI): Exact mass calculated for $C_{20}H_{15}O_1N_3Cl$ [M]$^+$: 348.08982, found: 348.08981;

^{1}H NMR ((CD_3)$_2$SO) δ 9.45 (2H, d, *J* = 6.9 Hz, 2 x C^{2}**H**), 8.87 (2H, d, *J* = 6.8 Hz, 2 x C^{3}**H**), 8.29 (1H, t, *J* = 1.8 Hz, C^{8}**H**), 8.20 (1H, dt, *J* = 7.7, 1.4 Hz, C^{10}**H**), 7.81 (1H, ddd, *J* = 8.2, 2.2, 1.2 Hz, C^{12}**H**), 7.72 (1H, t, *J* = 7.9 Hz, C^{11}**H**), 7.61-7.57 (2H, m, 2 x C^{16}**H**), 7.51-7.44 (3H, m, C^{17}**H** + 2 x C^{15}**H**), 5.97 (2H, s, C^{13}**H$_2$**);

^{13}C NMR ((CD_3)$_2$SO) δ 164.9 (C), 161.1 (C), 146.2 (2 x C^{2}), 137.7 (C), 134.3 (C), 134.0 (C), 132.8 (C^{12}), 131.7 (C^{11}), 129.5 (C^{17}), 129.3 (2 x C^{15}), 128.9 (2 x C^{16}), 126.8 (C^{8}), 126.0 (C^{10}), 125.0 (2 x C^{3}), 124.5 (C), 63.6 (C^{13});

IR (neat) (cm^{-1}): 2973, 2161, 1643, 1535, 1498, 1479, 1454, 1404, 1354, 1282, 1210, 1155, 1126, 1073, 1017, 921.

---

[7] Rao, V. S.; Chadra Sekhar, K. V. G. *Synth, Commun.* **2004**, *24*, 2153-2157.

[8] Rao, V. S.; Chadra Sekhar, K. V. G. *Synth, Commun.* **2004**, *24*, 2153-2157.

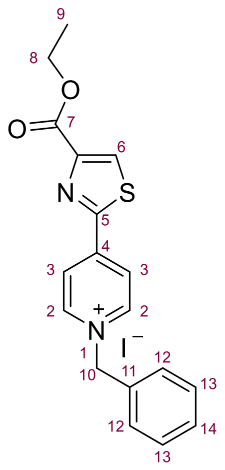

***N*-Benzyl-4-(4-(ethoxycarbonyl)thiazol-2-yl)pyridin-1-ium Iodide (1t)** The title compound was prepared according to General Procedure B using ethyl 2-(4-pyridyl)-1,3-thiazole-4-carboxylate[9] (468 mg, 2.0 mmol) to give salt **1t** (745 mg, 82%) as an orange solid.

m.p. (acetone): 140-142 °C;

HRMS (ESI): Exact mass calculated for $C_{18}H_{17}O_2N_2S$ $[M]^+$: 325.1005, found: 325.1005;

^{1}H NMR ($(CD_3)_2SO$) δ 9.30 (2H, d, *J* = 7.0 Hz, 2 x C^2**H**), 8.96 (1H, s, C^6**H**), 8.69 (2H, d, *J* = 6.9 Hz, 2 x C^3**H**), 7.58-7.54 (2H, m, 2 x C^{13}**H**), 7.49-7.42 (3H, m, 2 x C^{12}**H** + C^{14}**H**), 5.92 (2H, s, C^{10}**H**$_2$), 4.38 (2H, q, *J* = 7.1 Hz, C^8**H**$_2$), 1.34 (3H, t, *J* = 7.1 Hz, C^9**H**$_3$);

^{13}C NMR ($(CD_3)_2SO$) δ 161.5 (C), 160.1 (C), 148.3 (C), 145.9 (C)¸145.8 (2 x C^2), 134.6 (C^6)¸134.2 (C), 129.4 (C^{14}), 129.2 (2 x C^{12}), 128.8 (2 x C^{13}), 124.7 (2 x C^3), 63.0 (C^{10}), 61.3 (C^8), 14.2 (C^9);

IR (neat) (cm^{-1}): 3039, 3011, 2969, 2161, 1979, 1715, 1633, 1562, 1519, 1495, 1468, 1394, 1364, 1321, 1306, 1243.

***N*-Benzyl-4-(4,5-diphenyloxa-2-zole)pyridin-1-ium Iodide (1u)** The title compound was prepared according to General Procedure B using 4-(4,5-diphenyloxa-2-zole)pyridine (298 mg, 1.0 mmol) to give salt **1u** (444 mg, 86%) as an orange solid.

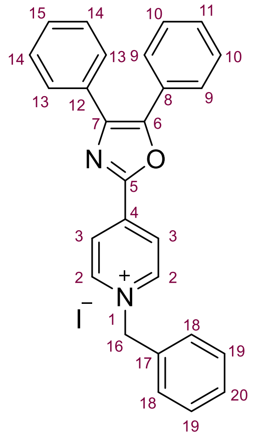

m.p. (acetone): 204-206 °C

HRMS (ESI): Exact mass calculated for $C_{27}H_{21}ON_2$ $[M]^+$: 389.16484, found: 389.16482.

^{1}H NMR ($(CD_3)_2SO$) δ 9.32 (2H, d, *J* = 7.0 Hz, 2 x C^2**H**), 8.71 (2H, d, *J* = 7.1 Hz, 2 x C^3**H**), 7.78-7.74 (2H, m, 2 x Ar**H**), 7.69-7.65 (2H, m, 2 x Ar**H**), 7.60-7.43 (11H, m, 11 x Ar**H**), 5.92 (2H, s, C^{16}**H**$_2$);

^{13}C NMR ($(CD_3)_2SO$) δ 154.9 (C), 148.9 (C), 145.8 (2 x C^2), 139.9 (C)¸138.4 (C), 134.2 (C), 130.8 (C)¸130.3 (CH), 129.4 (CH), 129.3 (2 x CH), 129.19 (CH), 129.18 (2 x CH), 129.0 (2 x CH), 128.8 (2 x CH), 127.8 (2 x CH), 127.0 (CH), 126.9 (2 x CH), 123.9 (2 x C^3), 63.1 (C^{16});

IR (neat) (cm^{-1}): 3456, 3011, 2162, 2021, 1669, 1632, 1510, 1479, 1451, 1419, 1391, 1352, 1320, 1274, 1227, 1178.

***N*-Benzyl-4-(4,5-diphenyloxa-2-zole)pyridin-1-ium Iodide (1v)** The title compound was prepared according to General Procedure B using 4-(4,5-di(4-methoxyphenyl)oxa-2-zole)pyridine (447 mg, 1.25 mmol) to give salt **1v** (515 mg, 72%) as a light orange solid.

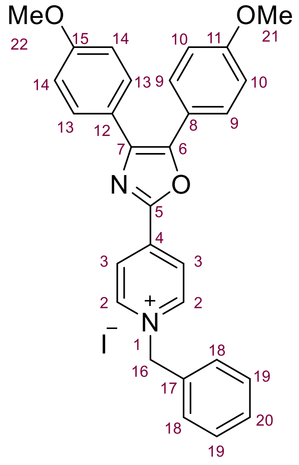

m.p. (acetone): 220-222 °C;

HRMS (ESI): Exact mass calculated for $C_{27}H_{25}O_3N_3Cl$ $[M]^+$: 474.1579, found: 474.1577;

^{1}H NMR ($(CD_3)_2SO$) δ 9.28 (2H, d, *J* = 7.1 Hz, 2 x C^2**H**), 8.65 (2H, d, *J* = 7.0 Hz, 2 x C^3**H**), 7.70-7.66 (2H, m, 2 x $C^{9/13}$**H**), 7.60-7.54 (4H, m, 2 x $C^{9/13}$**H** + 2 x C^{19}**H**), 7.50-7.43 (3H, m, 2 x C^{18}**H** + C^{20}**H**), 7.08 (2H, d, *J* = 8.9 Hz, 2 x $C^{10/14}$**H**), 7.05 (2H, d, *J* = 8.8 Hz, 2 x $C^{10/14}$**H**), 5.90 (2H, s, C^{16}**H**$_2$), 3.82 (3H, s, $C^{21/22}$**H**$_3$), 3.81 (3H, s, $C^{21/22}$**H**$_3$);

^{13}C NMR ($(CD_3)_2SO$) δ 160.6 (C), 159.7 (C), 154.2 (C), 148.6 (C), 145.6 (2 x C^2), 139.9 (C)¸137.3 (C), 134.2 (C), 129.5 (C^{20}), 129.3 (2 x C^{Ar}), 129.2 (2 x C^{Ar}), 128,8 (2 x C^{Ar}), 128.6 (2 x C^{Ar}), 123.6 (2 x C^3), 123.2 (C), 119,4 (C), 114.7 (C^{Ar}), 114.4 (C^{Ar}), 63.0 (C^{16}), 55.5 ($C^{21/22}$), 55.3 ($C^{21/22}$);

[9] Rzasa, R. M. et. al. *Bioorg. & Med. Chem.* **2007**, *15*, 6574-6595.

IR (neat) (cm^{-1}): 3458, 3012, 2161, 2022, 1668, 1640, 1553, 1511, 1481, 1457, 1419, 1391, 1352, 1293, 1253, 1227.

***N*-Benzyl-4-(4-(4-chlorophenyl)-5-phenyloxa-2-zole)pyridin-1-ium Iodide (1w)** The title compound was prepared according to General Procedure B using 4-(4-(4-chlorophenyl)-5-phenyloxa-2-zole)pyridine (166 mg, 0.5 mmol) to give salt **1w** (237 mg, 86%) as a yellow solid.

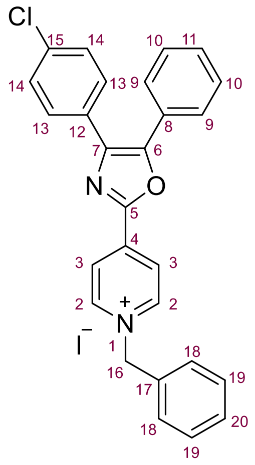

m.p. (acetone): 255-257 °C;

HRMS (ESI): Exact mass calculated for $C_{27}H_{20}ON_2Cl$ $[M]^+$: 423.12587, found: 423.12567;

^{1}H NMR ($(CD_3)_2SO$) δ 9.33 (2H, d, *J* = 7.1 Hz, 2 x C^{2}**H**), 8.71 (2H, d, *J* = 7.0 Hz, 2 x C^{3}**H**), 7.80-7.42 (14H, m, 14 x C^{Ar}), 5.95 (2H, s, C^{16}**H**$_2$);

^{13}C NMR ($(CD_3)_2SO$) δ 155.0 (C), 149.2 (C), 145.8 (2 x C^{2}), 139.8 (C), 137.2 (C), 134.1 (C), 133.8 (C), 130.4 (C), 129.5 (C^{Ar}), 129.4 (C^{Ar}), 129.3 (C^{Ar}), 129.1 (C^{Ar}), 128.8 (C^{Ar}), 128.6 (C^{Ar}), 127.9 (C^{Ar}), 127.1 (C^{Ar}), 126.8 (C), 124.0 (2 x C^{3}), 63.1 (C^{16});

IR (neat) (cm^{-1}): 3459, 2993, 2161, 1978, 1636, 1546, 1517, 1475, 1457, 1420, 1391, 1349, 1296, 1252, 1227, 1180.

***N*-Benzyl-4-(benzisoxazole)pyridin-1-ium Iodide (1x)** The title compound was prepared according to General Procedure B using **S23** (294 mg, 1.5 mmol) to give salt **1x** (450 mg, 72%) as a yellow solid.

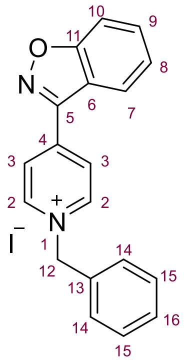

m.p. (acetone): 224-226 °C;

HRMS (ESI): Exact mass calculated for $C_{19}H_{15}ON_2$ $[M]^+$: 287.11789, found: 287.11778;

^{1}H NMR ($(CD_3)_2SO$) δ 9.42 (2H, d, *J* = 6..9 Hz, 2 x C^{2}**H**), 8.83 (2H, d, *J* = 6.9 Hz, 2 x C^{3}**H**), 8.35 (1H, dt, *J* = 8.1, 1.0 Hz, C$^{7/10}$**H**), 8.00 (1H, dt, *J* = 8.6, 0.8 Hz, C$^{7/10}$**H**), 7.84 (1H, ddd, *J* = 8.4, 7.1, 1.1 Hz, C$^{8/9}$**H**), 7.66-7.60 (3H, m, C$^{8/9}$**H** + 2 x C^{15}**H**), 7.52-7.43 (3H, m, 2 x C^{14}**H** + C^{16}**H**), 6.00 (2H, s, C^{12}**H**$_2$);

^{13}C NMR ($(CD_3)_2SO$) δ 164.0 (C), 153.2 (C), 145.8 (2 x C^{2}), 143.6 (C), 134.1 (C), 131.5 (C$^{8/9}$), 129.5 (C^{16}), 129.3 (2 x C^{14}), 129.0 (2 x C^{15}), 126.6 (2 x C^{3}), 125.6 (C$^{8/9}$), 122.5 (C$^{7/10}$), 118.5 (C)¸110.6 (C$^{7/10}$), 63.3 (C^{12});

IR (neat) (cm^{-1}): 2971, 1638, 1606, 1563, 1494, 1450, 1421, 1392, 1316, 1281, 1227, 1140, 941, 865, 820, 764, 754, 700.

4-(4-Chlorophenyl)-1-(4-iodobutyl)piperidine-2,6-dione (S24)

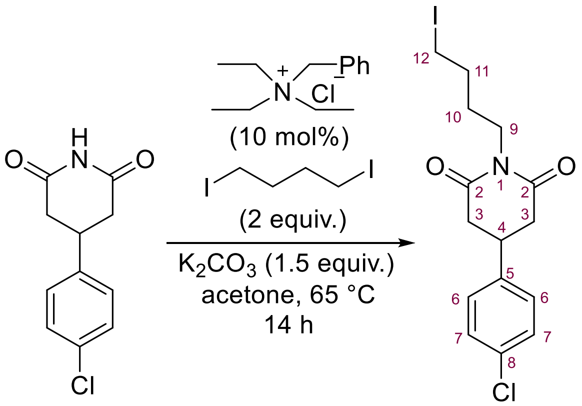

4-(4-Chlorophenyl)-piperidine-2,6-dione[10] (1.11 g, 5 mmol), potassium carbonate (1.03 g, 7.5 mmol), triethylbenzylammonium chloride (114 mg, 10 mol%), and 1,4-diiodobutane (1.32 mL, 10 mmol) were dissolved in acetone (50 mL) and heated at refluxed for 14 hours. The solution was cooled, filtered and concentrated *in vacuo*. The crude material was purified by FCC (10-20% EtOAc in pentane) to give *alkyl iodide* **S24** (1.7 g, 84%) as a light yellow oil.

HRMS (ESI): Exact mass calculated for $C_{15}H_{18}O_2N^{35}Cl^{127}I$ $[M+H]^+$: 406.00653, found: 406.00674;

^{1} H NMR ($CDCl_3$) δ 7.35 (2H, d, *J* = 8.5 Hz, 2 x C^6**H**), 7.15 (2H, d, *J* = 8.1 Hz, 2 x C^7**H**), 3.83 (2H, t, *J* = 7.2 Hz, C^{12}**H**$_2$), 3.35 (1H, tt, *J* = 11.4, 4.3 Hz, C^4**H**), 3.20 (t, *J* = 6.9 Hz, C^9**H**$_2$), 2.99 (2H, ddt, *J* = 17.1, 4.3, 1.0 Hz, 2 x C^3**H**$_2$), 2.78 (2H, ddt, *J* = 17.1, 11.5, 1.1 Hz, 2 x C^3**H**$_2$), 1.85-1.78 (2H, m, C^{11}**H**$_2$), 1.70-1.60 (2H, m, C^{10}**H**$_2$);

^{13}C NMR ($CDCl_3$) δ 171.2 (2 x C^2), 139.0 (C^6), 133.5 (C^5), 129.3 (2 x C^7), 127.7 (2 x C^6), 39.7 (2 x C^3), 38.6 (C^9), 34.1 (C^4), 30.8 (C^{11}), 29.0 (C^{10}), 5.7 (C^{12});

IR (neat) (cm^{-1}): 2972, 2161, 1980, 1719, 1657, 1492, 1443, 1392, 1355, 1336, 1286, 1250, 1211, 1185, 1142, 1093.

_N_-(4-(4-Chlorophenyl)-1-(4-butyl)piperidine-2,6-dione) 4-(benzoxazole)pyridinium Iodide (1aa)

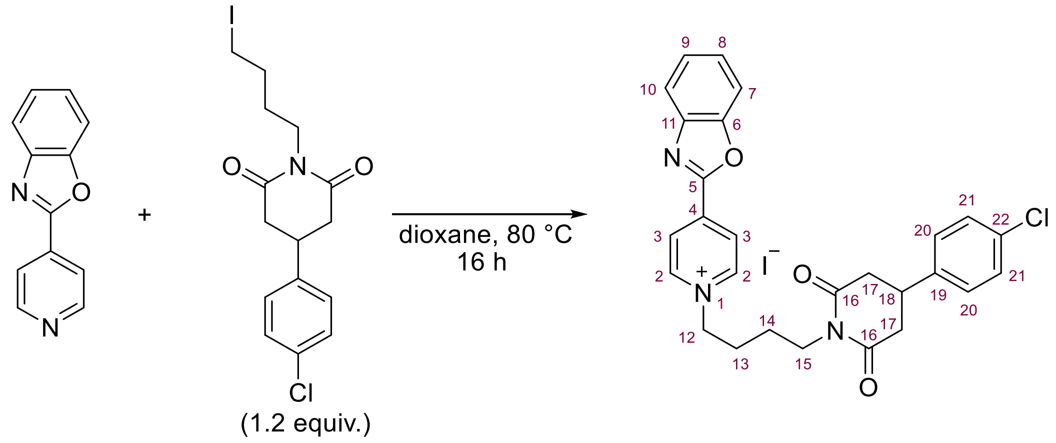

4-(4-Chlorophenyl)-1-(4-iodobutyl)piperidine-2,6-dione **S24** (975 mg, 2.4 mmol) and 4-(benzoxazole)pyridine (392 mg, 2 mmol) were dissolved in 1,4-dioxane (5 mL) and heated in a sealed flask at 80 °C for 14 hours. The solution was cooled and filtered, the resulting solid was washed with diethyl ether and dried to give *salt* **1aa** (800 mg, 66%) as an orange solid.

m.p. (acetone): 190-192 °C;

HRMS (ESI): Exact mass calculated for $C_{27}H_{25}O_3N_3Cl$ $[M]^+$: 474.1579, found: 474.1577;

^{1}H NMR ($(CD_3)_2SO$) δ 9.28 (2H, d, *J* = 6.9 Hz, 2 x C^2**H**), 8.80 (2H, d, *J* = 6.8 Hz, 2 x C^3**H**), 8.02 (1H, dt, *J* = 8.0, 1.1 Hz, $C^{7/10}$**H**), 7.95 (1H, dt, *J* = 8.3, 0.9 Hz, $C^{7/10}$**H**), 7.65 (1H, ddd, *J* = 8.3, 7.3, 1.3 Hz, $C^{8/9}$**H**), 7.57 (1H, ddd, *J* = 8.4, 7.4, 1.1 Hz, $C^{8/9}$**H**), 7.40 (2H, d, *J* = 8.6 Hz, 2 x C^{20}**H**), 7.33 (2H, d, *J* = 8.6 Hz, 2 x C^{21}**H**), 4.70 (2H, t, *J* = 7.5 Hz, C^{12}**H**$_2$), 3.73 (2H, t, *J* = 7.2 Hz, C^{15}**H**$_2$), 3.50-3.44 (1H, m, C^{18}**H**), 2.95 (2H, dd, *J* = 16.6, 11.2 Hz, 2 x C^{17}**H**$_2$), 2.83 (2H, dd, *J* = 16.6, 4.5 Hz, 2 x C^{17}**H**$_2$), 1.95 (2H, p, *J* = 7.5, 6.9 Hz, C^{13}**H**$_2$), 1.52 (2H, p, *J* = 7,4 Hz, C^{14}**H**$_2$);

^{13}C NMR ($(CD_3)_2SO$) δ 171.8 (2 x C^{16}), 157.7 (C_q), 150.9 (C_q), 146.0 (2 x C^2), 141.2 (C_q), 140.8 (C_q), 140.5 (C_q), 131.6 (C_q), 128.7 (2 x C^{21}), 128.6 (2 x C^{20}), 128.3 ($C^{8/9}$), 126.2 ($C^{8/9}$), 125.1 (2 x C^3), 121.3 ($C^{7/10}$), 111.8 ($C^{7/10}$), 60.5 (C^{12}), 38.8 (2 x C^{17}), 38.0 (C^{15}), 33.0 (C^{18}), 28.2 (C^{13}), 24.1 (C^{14});

IR (neat) (cm^{-1}): 3402, 3079, 2955, 2161, 1978, 1715, 1662, 1646, 1542, 1403, 1351, 1323, 1215, 1173, 1161, 1119.

[10] Romeo, G.; Materia, L.; Modica, M. N.; Pittala, V.; Salerno, L.; Siracusa, M. A.; Manetti, F.; Botta, M.; Minneman, K. P. *Eur. J. Med. Chem.* **2011**, *46*, 2676-2690.

N-(4-(4-*bis*(4-Fluorophenyl)-1-butyl-4-(benzoxazole)pyridinium Iodide (1ab)

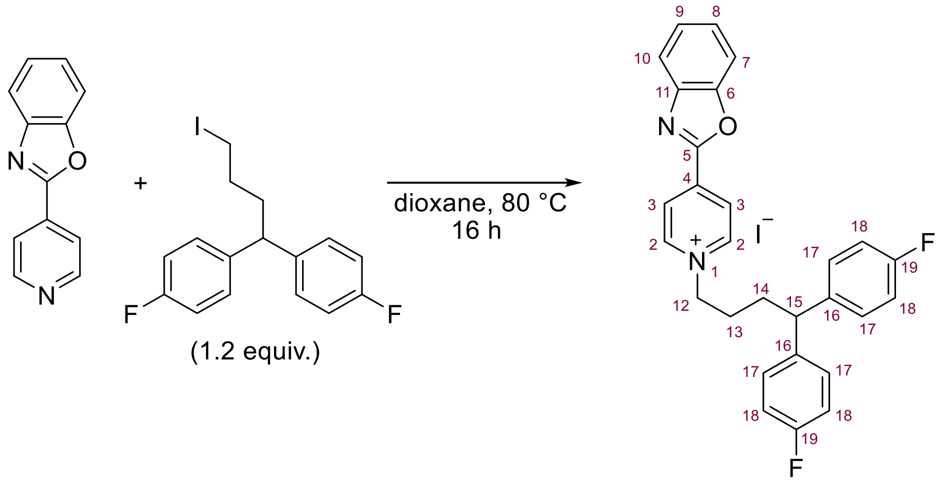

4-*bis*-(4-Fluorophenyl)-1-iodobutane (744 mg, 2.4 mmol) and 4-(benzoxazole)pyridine (392 mg, 2 mmol) were dissolved in 1,4-dioxane (5 mL) and heated in a sealed flask at 80 °C for 14 hours. The solution was concentrated *in vacuo* and the crude material purified by FCC (0-5% MeOH in CH_2Cl_2) to give *salt* **1ab** (329 mg, 29%) as a red solid.

The sample was contaminated by 2.6% of a phosphonium salt (^{1}H NMR ($(CD_3)_2SO$) δ 7.75 (15H m) + ^{31}P NMR ($(CD_3)_2SO$) δ +24.2. The 15H are worth 0.4H compared to 1H of compound **1ab**, thus 0.4/15 = 0.0267.

m.p. (acetone): 115-117 °C;

HRMS (ESI): Exact mass calculated for $C_{28}H_{23}ON_2F_2$ $[M]^+$: 441.17730, found: 441.17695;

^{1}H NMR ($(CD_3)_2SO$) δ 9.24 (2H, d, *J* = 7.1 Hz, 2 x C^2**H**), 8.78 (2H, d, *J* = 6.9 Hz, 2 x C^3**H**), 8.02 (1H, dt, *J* = 7.9, 0.9 Hz, $C^{7/10}$**H**), 7.95 (1H, dt, *J* = 8.2, 0.9 Hz, $C^{7/10}$**H**), 7.65 (1H, ddd, *J* = 8.4, 7.3, 1.3 Hz, $C^{8/9}$**H**), 7.57 (1H, ddd, *J* = 8.3, 7.3, 1.1 Hz, $C^{8/9}$**H**), 7.34-7.28 (4H, m, 4 x C^{17}**H**), 7.14-7.06 (4H, m, 4 x C^{18}**H**)' 4.71 (2H, t, *J* = 7.1 Hz, C^{12}**H**$_2$), 4.03 (1H, t, *J* = 7.8 Hz, C^{15}**H**), 2.08-2.01 (2H, m, C^{14}**H**$_2$), 1.90-1.80 (2H, m, C^{13}**H**$_2$),

^{13}C NMR ($(CD_3)_2SO$) δ 160.7 (d, *J* = 242.2 Hz, 2 x C^{19}), 157.7 (C_q), 150.9 (C_q), 145.9 (2 x C^2), 141.2 (C_q), 140.6 (C_q), 140.5 (C_q), 129.3 (d, *J* = 8.1 Hz, 2 x C^{17}), 128.3 ($C^{8/9}$), 126.1 ($C^{8/9}$), 125.1 (2 x C^3), 121.3 ($C^{7/10}$), 115.2 (d, *J* = 21.1 Hz, 2 x C^{18}), 111.8 ($C^{7/10}$), 60.7 (C^{12}), 48.3 (C^{15}), 31.1 (C^{14}), 29.5 (C^{13});

^{19}F NMR ($(CD_3)_2SO$) d –116.8 (ddt, *J* = 11.3, 8.6, 5.3 Hz);

IR (neat) (cm^{-1}): 3431, 3036, 2926, 2162, 1640, 1604, 1552, 1505, 1461, 1391, 1353, 1319, 1222, 1175, 1158, 1112.

N-3-(3-acetylindole)-1-propyl-4-(benzothiazole)pyridinium Iodide (1ac)

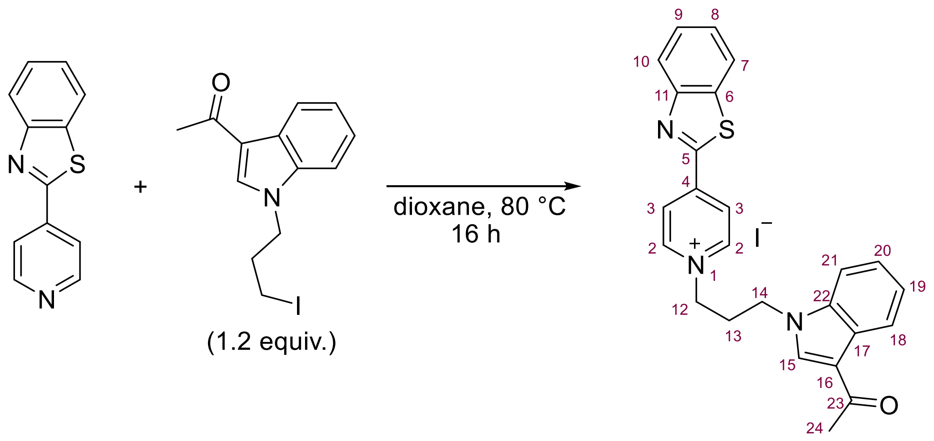

3-(3-Acetylindole)-1-iodopropane[11] (817 mg, 2.4 mmol) and 4-(benzothiazole)pyridine (424 mg, 2 mmol) were dissolved in 1,4-dioxane (5 mL) and heated in a sealed flask at 80 °C for 14 hours. The solution was cooled and filtered, the resulting solid was washed with diethyl ether and dried to give *salt* **1ac** (896 mg, 83%) as an orange solid.
m.p. (acetone): 182-184 °C;
HRMS (ESI): Exact mass calculated for $C_{25}H_{22}O_1N_3S$ $[M]^+$: 412.14781, found: 412.14767;
^{1}H NMR ($(CD_3)_2SO$) δ 9.14 (2H, d, *J* = 7.0 Hz, 2 x C^2**H**), 8.62 (2H, d, *J* = 7.0 Hz, 2 x C^3**H**), 8.37-8.35 (2H, m, C^{15}**H** + C^{Ar}**H**), 8.26 (1H, dd, *J* = 7.4, 1.2 Hz, C^{Ar}**H**), 8.09 (1H, dt, *J* = 7.8, 1.0 Hz, C^{Ar}**H**), 7.73-7.60 (3H, m, 3 x C^{Ar}**H**), 7.28 (1H, ddd, *J* = 8.3, 7.1, 1.3 Hz, C^{Ar}**H**), 7.17 (1H, ddd, *J* = 8.0, 7.1, 1.0 Hz, C^{Ar}**H**), 4.79 (2H, t, *J* = 7.1 Hz, C^{12}**H**$_2$), 4.44 (2H, t, *J* = 7.0 Hz, C^{14}**H**$_2$), 2.63 (2H, p, *J* = 7.1 Hz, C^{13}**H**$_2$), 2.45 (3H, s, C^{24}**H**$_3$);
^{13}C NMR ($(CD_3)_2SO$) δ 192.1 (C^{23}), 161.3 (C_q), 153.3 (C_q), 146.0 (C_q), 145.6 (2 x C^2), 136.8 (C^{15}), 136.3 (C_q), 136.1 (C_q), 127.8 (C^{Ar}), 127.7 (C^{Ar}), 125.7 (C_q), 124.5 (2 x C^3), 124.3 (C^{Ar}), 123.3 (C^{Ar}), 122.9 (C^{Ar}), 122.1 (C^{Ar}), 121.6 (C^{Ar}), 116.2 (C_q), 110.7 (C^{Ar}), 58.6 (C^{12}), 43.5 (C^{14}), 30.4 (C^{13}), 27.4 (C^{24});
IR (neat) (cm^{-1}): 3051, 2162, 1632, 1561, 1528, 1480, 1451, 1422, 1391, 1336, 1320, 1228, 1208, 1181, 1130, 1113.

1-(4-(3-iodopropoxy)-3-methoxyphenyl)ethenone (S25)

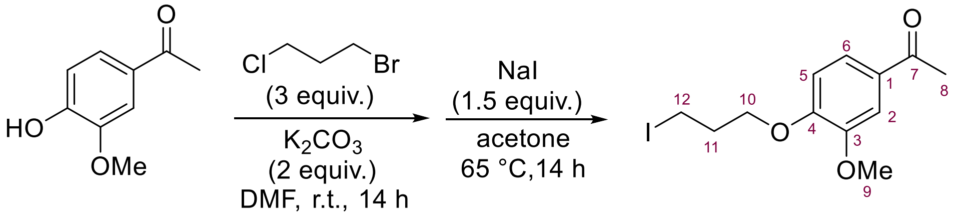

Acetovanilin (2.50 g, 15 mmol), potassium carbonate (4.10 g, 30 mmol), and 3-bromo-1-chloropropane (4.5 mL, 45 mmol) were dissolved in DMF (20 mL) and stirred at room temperature for 14 hours. The solution was poured in water (50 mL) and extracted with CH_2Cl_2 (3 x 20 mL). The organic layers were combined, dried ($MgSO_4$), filtered, and concentrated *in vacuo.* The material was eluted through a silica pad (30% EtOAc in pentane) and ^{1}H NMR analysis of the resulting material indicated the materials was 90% pure and it was taken onto the next step without any further purification.
The crude material and sodium iodide (3.4 g, 22.5 mmol) were dissolved in acetone (25 mL) and heated at reflux for 14 hours. The solution was diluted with water (50 mL) and Et_2O (50 mL) and separated. The aqueous layer was washed with Et_2O (2 x 50 mL) and the organic layers were combined, dried ($MgSO_4$), filtered, and concentrated *in vacuo* to give an *alkyl iodide* **S25** (3.05 g, 61% over 2 steps) as a yellow solid.
m.p. (acetone): 64-66 °C;
HRMS (ESI): Exact mass calculated for $C_{12}H_{16}O_3{}^{127}I$ $[M+H]^+$: 335.01386, found: 335.01279;
^{1}H NMR ($CDCl_3$) δ 7.57 (1H, dd, *J* = 8.3, 2.0 Hz, C^6**H**), 7.54 (1H, d, *J* = 2.0 Hz, C^2**H**), 6.92 (1H, d, *J* = 8.3 Hz, C^5**H**), 4.17 (2H, t, *J* = 5.9 Hz, C^{12}**H**$_2$), 3.92 (3H, s, C^9**H**$_3$), 3.40 (2H, t, *J* = 6.6 Hz, C^{10}**H**$_2$), 2.57 (3H, s, C^8**H**$_3$), 2.45-2.23 (2H, m, C^{11}**H**$_2$);
^{13}C NMR ($CDCl_3$) δ 196.7 (C^7), 152.5 (C), 149.4 (C), 130.8 (C), 123.1 (ArC), 111.6 (ArC), 110.6 (ArC), 68.5 (C), 56.0 (C), 32.7 (C), 26.3 (C), 2.1 (C);
IR (neat) (cm^{-1}): 2972, 1724, 1670, 1584, 1508, 1466, 1445, 1414, 1391, 1345, 1266, 1245, 1218, 1180, 1146, 1077.

[11] Artis, D. R.; Cho, I.-S.; Jaime-Figueroa, S.; Muchowski, J. M. *J. Org. Chem.* **1994**, *59*, 2456.

N-(3-(4-acetyl-2-methoxyphenoxy)propyl)-4-(benzoxazole)pyridinium Iodide (1ad)

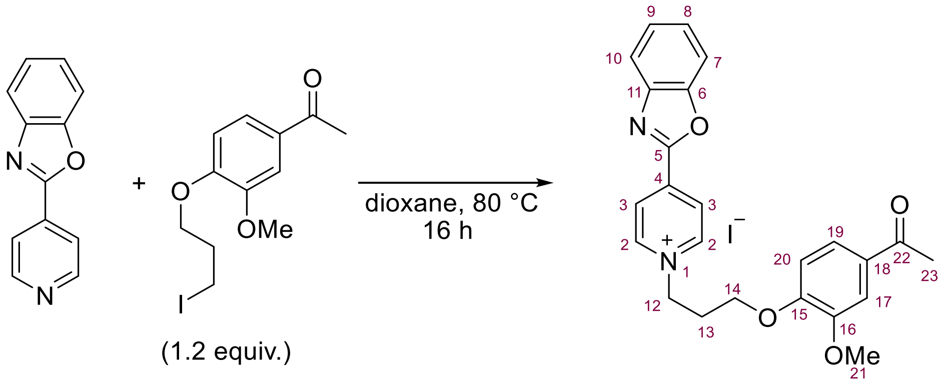

1-(4-(3-iodopropoxy)-3-methoxyphenyl)ethenone **S25** (801 mg, 2.4 mmol) and 4-(benzoxazole)pyridine (392 mg, 2 mmol) were dissolved in 1,4-dioxane (5 mL) and heated in a sealed flask at 80 °C for 14 hours. The solution was filtered and washed with Et_2O to give *salt* **1ad** (1.04 g, 98%) as red solid.

m.p. (acetone): 180-182 °C;

HRMS (ESI): Exact mass calculated for $C_{24}H_{23}O_4N_2$ $[M]^+$: 403.16523, found: 403.16507;

^1H NMR ($(CD_3)_2SO$) δ 9.33 (2H, d, J = 7.1 Hz, 2 x C^2**H**), 8.79 (2H, d, J = 7.0 Hz, 2 x C^3**H**), 8.02 (1H, ddd, J = 8.1, 1.3, 0.7 Hz, $C^{7/10}$**H**), 7.97 (1H, dt, J = 8.3, 0.9 Hz, $C^{7/10}$**H**), 7.66 (1H, ddd, J = 8.4, 7.4, 1.3 Hz, $C^{8/9}$**H**), 7.62-7.56 (2H, m, $C^{8/9}$**H** + C^{20}**H**), 7.32 (1H, d, J = 2.1 Hz, C^{17}**H**), 7.05 (1H, d, J = 8.5 Hz, C^{19}**H**), 4.91 (2H, t, J = 6.4 Hz, C^{12}**H_2**), 4.26 (2H, t, J = 5.6 Hz, C^{14}**H_2**), 3.57 (3H, s, C^{21}**H_3**), 2.56-2.44 (5H, m, C^{13}**H_2** + C^{23}**H_2**);

^{13}C NMR ($(CD_3)_2SO$) δ 196.3 (C^{22}), 157.7 (C_q), 151.4 (C_q), 150.9 (C_q), 148.3 (C_q), 146.2 (2 x C^1), 141.2 (C_q), 140.3 (C_q), 130.2 (C_q), 128.3 ($C^{8/9}$), 126.2 ($C^{8/9}$), 124.8 (2 x C^3), 122.8 (C^{20}), 121.3 ($C^{7/10}$), 111.8 ($C^{7/10}$), 111.7 (C^{19}), 110.1 (C^{17}), 66.2 (C^{14}), 59.5 (C^{12}), 55.2 (C^{21}), 29.7 (C^{13}), 26.3 (C^{23});

IR (neat) (cm^{-1}): 3457, 3010, 2162, 2022, 1668, 1642, 1586, 1556, 1510, 1458, 1419, 1391, 1352, 1320, 1273, 1225.

Reductive Hydroxymethylation

General Procedure C

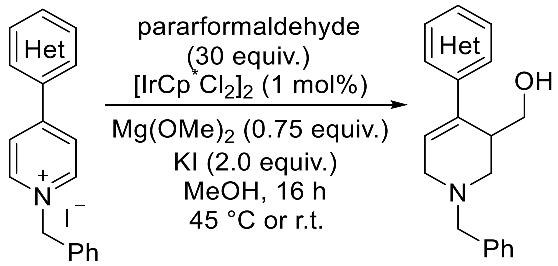

Substrate (0.5 mmol), KI (166 mg, 1.0 mmol), paraformaldehyde (450 mg, 15 mmol), and $[IrCp^*Cl_2]_2$ (1 mol%) was added to a microwave vial. MeOH (1.04 mL) and $Mg(OMe)_2$ (0.21 mL, 0.75 mmol, 0.89M in MeOH) was added and the solution heated at the required temperature for 16 hours. The solution was cooled to room temperature, diluted with EtOAc (50 mL) and brine (50 mL). The solution was separated, and the aqueous layer was washed with EtOAc (2 x 50 mL). The organic layers were combined, dried ($MgSO_4$), filtered, and concentrated *in vacuo*. The crude material was purified by flash column chromatography (FCC) to furnish *amines* **X**-**X**.

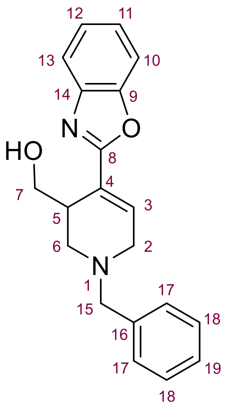

(4-(Benzoxazole)-*N*-benzyl-1,2,3,6-tetrahydropyridin-3-yl)methanol (2a)

The title compound was prepared according to General Procedure C using salt **X** (207 mg, 0.5 mmol) at room temperature for 16 hours. The product was purified by FCC (10%-30% EtOAc in pentane) to give *amine* **2a** (122 mg, 76%) as an orange solid.

m.p. (acetone): 119-121 °C;

HRMS (ESI): Exact mass calculated for $C_{20}H_{21}O_2N_2$ $[M+H]^+$: 321.15975, found: 321.15988;

^{1}H NMR ($CDCl_3$) δ 7.62-7.57 (1H, m, $C^{10/13}$**H**), 7.41-7.36 (1H, m, $C^{10/13}$**H**), 7.32-7.19 (7H, m, C^{11}**H** + C^{12}**H** + 2 x C^{17}**H** + 2 x C^{18}**H** + C^{19}**H**), 7.02 (1H, dd, *J* = 4.6, 2.4 Hz, C^3**H**), 4.81 (1H, br s, O**H**), 4.02 (1H, dt, *J* = 10.4, 2.4 Hz, C^7**H**$_2$), 3.91 (1H, dd, *J* = 10.5, 2.8 Hz, C^7**H**$_2$), 3.58 (2H, s, C^{15}**H**$_2$), 3.48 (1H, dd, *J* = 18.9, 4.7 Hz, C^2**H**$_2$), 3.09 (1H, dt, *J* = 11.3, 1.4 Hz, C^6**H**$_2$), 3.01 (1H, dt, *J* = 4.4, 2.3 Hz, C^5**H**), 2.87 (1H, dt, *J* = 18.8, 2.5 Hz, C^1**H**$_2$), 2.56 (1H, ddd, *J* = 11.3, 3.6, 2.0 Hz, C^6**H**$_2$);

^{13}C NMR (($CDCl_3$) δ 161.9 (C^8), 150.0 (C), 141.5 (C), 136.5 (C), 133.5 (C^3), 128.8 (2 x C^{18}), 128.4 (2 x C^{17}), 127.4 (C^{19}), 125.0 (C), 124.9 ($C^{11/12}$) , 124.1 ($C^{11/12}$), 119.7 ($C^{10/13}$), 110.1 ($C^{10/13}$), 66.1 (C^7), 62.3 (C^{15}), 54.6 (C^6), 52.7 (C^2), 36.7 (C^5);

IR (neat) (cm^{-1}): 2981, 2161, 2032, 1654, 1540, 1453, 1364, 1242, 1013, 920, 798, 744, 699, 624.

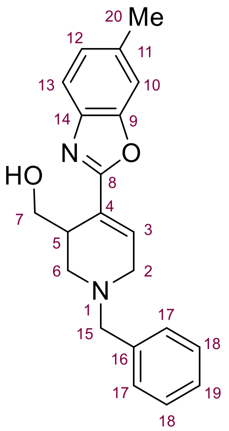

(4-(5-Methylbenzoxazole)-*N*-benzyl-1,2,3,6-tetrahydropyridin-3-yl)methanol (2b)

The title compound was prepared according to General Procedure C using salt **X** (214 mg, 0.5 mmol) at 45 °C for 16 hours. The product was purified by FCC (10%-30% EtOAc in pentane) to give *amine* **2b** (115 mg, 69%) as a yellow solid.

m.p. (acetone): 130-132 °C;

HRMS (ESI): Exact mass calculated for $C_{21}H_{23}O_2N_2$ $[M+H]^+$: 335.17540, found: 335.17558;

^{1}H NMR ($CDCl_3$) δ 7.47 (1H, d, *J* = 8.1 Hz, C^{13}**H**), 733-7.17 (6H, C^{10}**H** + 2 x C^{17}**H** + 2 x C^{18}**H** + C^{19}**H**), 7.04 (1H, dd, *J* = 8.2, 1.5 Hz, C^{12}**H**), 6.99 (1H, dd, *J* = 4.6, 2.4 Hz, C^3**H**), 5.25 (1H, s, O**H**), 4.02 (1H, dt, *J* = 11.2, 2.3 Hz, C^7**H**$_2$), 3.91 (1H, dd, *J* = 10.5, 2.9 Hz, C^7**H**$_2$), 3.60 (2H, s, C^{15}**H**$_2$), 3.49 (1H, dd, *J* = 18.8, 4.6 Hz, C^2**H**$_2$), 3.09 (1H, d, *J* = 10.8 Hz, C^6**H**$_2$), 3.04-2.95 (1H, m, C^5**H**), 2.89 (1H, dt, *J* = 18.7, 2.5 Hz, C^2**H**$_2$), 2.58 (1H, ddd, *J* = 11.4, 3.6, 2.0 Hz, C^6**H**$_2$), 2.40 (3H, s, C^{20}**H**$_3$);

^{13}C NMR ($CDCl_3$) δ 161.6 (C), 150.5 (C), 139.5 (C), 136.7 (C), 135.6 (C), 133.0 (C^{3}). 129.0 (2 x C$^{17/18}$), 128.6 (2 x C$^{17/18}$), 127.6 (C^{19}), 125.5 (C^{12}), 125.3 (C), 119.2 (C^{13}), 110.5 (C^{10}), 66.5 (C^{7}), 62.6 (C^{15}), 55.1 (C^{6}), 53.0 (C^{2}), 36.8 (C^{5}), 21.8 (C^{20});
IR (neat) (cm^{-1}): 3365, 2919, 2807, 2361, 2161, 2032, 1719, 1654, 1602, 1539, 1452, 1395, 1358, 1329, 1287, 1263, 1242.

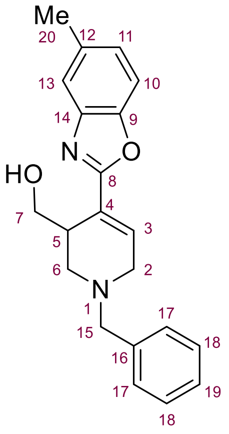

(4-(4-Methylbenzoxazole)-*N*-benzyl-1,2,3,6-tetrahydropyridin-3-yl)methanol (2c)

The title compound was prepared according to General Procedure C using salt **X** (214 mg, 0.5 mmol) at 45 °C for 16 hours. The product was purified by FCC (10%-30% EtOAc in pentane) to give *amine* **2c** (118 mg, 71%) as a yellow solid.
m.p. (acetone): 85-87 °C;
HRMS (ESI): Exact mass calculated for $C_{21}H_{23}O_2N_2$ $[M+H]^+$: 335.17540, found: 335.17538;
^{1}H NMR ($CDCl_3$) δ 7.47 (1H, s, C^{13}**H**), 7.40-7.29 (6H, m, C^{10}**H** + 2 x C^{17}**H** + 2 x C^{18}**H** + C^{19}**H**), 7.12 (1H, dd, *J* = 8.3, 1.6 Hz, C^{11}**H**), 7.09 (1H, dd, *J* = 4.6, 2.4 Hz, C^{3}**H**), 5.22 (1H br s, O**H**), 4.10 (1H, dt, *J* = 10.5, 2.5 Hz, C^{7}**H**$_2$), 3.99 (1H, dd, *J* = 10.5, 2.8 Hz, C^{7}**H**$_2$), 3.68 (2H, s, C^{15}**H**$_2$), 3.57 (1H, dd, *J* = 18.8, 4.6 Hz, C^{2}**H**$_2$), 3.18 (1H, dt, *J* = 11.3, 1.4 Hz, C^{6}**H**$_2$), 3.10 (1H, t, *J* = 2.9 Hz, C^{5}**H**), 2.97 (1H, dt, *J* = 18.7, 2.5 Hz, C^{2}**H**$_2$), 2.67 (1H, ddd, *J* = 11.3, 3.6, 2.0 Hz, C^{6}**H**$_2$), 2.46 (3H, s, C^{20}**H**$_3$);
^{13}C NMR ($CDCl_3$) δ 162.2 (C), 148.4 (C), 141.9 (C), 136.7 (C), 134.1 (C), 133.2 (C^{3}), 129.0 (2 x C^{18}), 128.6 (2 x C^{17}), 127.7 (C^{19}), 126.2 (C^{11}), 125.3 (C), 119.8 (C^{13}), 109.6 (C^{10}), 66.5 (C^{7}), 62.8 (C^{15}), 55.1 (C^{6}), 53.0 (C^{2}), 36.8 (C^{5}), 21.5 (C^{20});
IR (neat) (cm^{-1}): 2924, 2161, 1657, 1541, 1452, 1432, 1364, 1345, 1304, 1263, 1192, 1141, 1069, 1027, 1016, 989, 957.

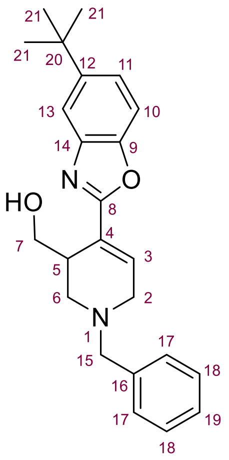

(4-(4-*tert*-Butylbenzoxazole)-*N*-benzyl-1,2,3,6-tetrahydropyridin-3-yl)methanol (2d)

The title compound was prepared according to General Procedure C using salt **X** (xx mg, 0.5 mmol) at 45 °C for 16 hours. The product was purified by FCC (10%-30% EtOAc in pentane) to give *amine* **2d** (141 mg, 75%) as a yellow solid.
m.p. (acetone): 140-142 °C;
HRMS (ESI): Exact mass calculated for $C_{24}H_{29}O_2N_2$ $[M+H]^+$: 377.22235, found: 377.22238;
^{1}H NMR ($CDCl_3$) δ 7.73 (1H, dd, *J* = 1.7, 0.8 Hz, C^{13}**H**), 7.42-7.29 (7H, m, C^{10}**H** + C^{11}**H** + 2 x C^{17}**H** + 2 x C^{18}**H** + C^{19}**H**), 7.09 (1H, dd, *J* = 4.6, 2.4 Hz, C^{3}**H**), 5.22 (1H, br s O**H**), 4.10 (1H, ddd, *J* = 10.5, 2.9, 2.1 Hz, C^{7}**H**$_2$), 4.00 (1H, dd, *J* = 10.5, 2.9 Hz, C^{7}**H**$_2$), 3.68 (2H, s, C^{15}**H**$_2$), 3.57 (1H, dd, *J* = 18.8, 4.6 Hz, C^{2}**H**$_2$), 3.18 (1H, dt, *J* = 11.3, 1.4 Hz, C^{6}**H**$_2$), 3.12-3.08 (1H, m, C^{5}**H**), 2.98 (1H, dt, *J* = 18.8, 2.4 Hz, C^{2}**H**$_2$), 2.68 (1H, ddd, *J* = 11.3, 3.6, 2.0 Hz, C^{6}**H**$_2$), 1.38 (9H, s, 3 x C^{21}**H**$_3$);
^{13}C NMR ($CDCl_3$) δ 162.3 (C), 148.2 (C), 147.8 (C), 141.6 (C), 136.7 (C), 133.1 (C^{3}), 129.1 (2 x C^{17}), 128.6 (2 x C^{18}), 127.7 (C^{19}), 125.4 (C), 122.9 (C^{11}), 116.4 (C^{13}), 109.4 (C^{10}), 66.5 (C^{7}), 62.6 (C^{15}), 55.1 (C^{6}), 53.0 (C^{2}), 36.9 (C^{5}), 34.9 (C^{20}), 31.7 (3 x C^{21});
IR (neat) (cm^{-1}): 2951, 1633, 1540, 1510, 1454, 1408, 1374, 1341, 1301, 1239, 1190, 1146, 1110, 1060, 1023, 986, 957.

(4-(5-Chlorobenzoxazole)-*N*-benzyl-1,2,3,6-tetrahydropyridin-3-yl)methanol (2e)

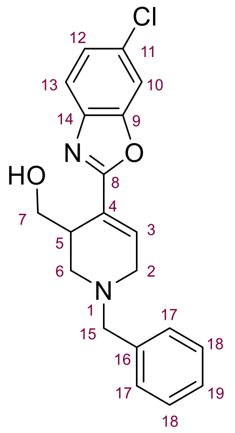

The title compound was prepared according to General Procedure C using salt **X** (224 mg, 0.5 mmol) at 45 °C for 16 hours. The product was purified by FCC (10%-30% EtOAc in pentane) to give *amine* **2e** (105 mg, 59%) as a yellow solid.
m.p. (acetone): 154-156 °C;
HRMS (ESI): Exact mass calculated for $C_{20}H_{20}O_2N_2{}^{35}Cl$ $[M+H]^+$: 355.12078, found: 355.12082;
^1H NMR ($CDCl_3$) δ 7.59 (1H, d, J = 8.6 Hz, C^{13}**H**), 7.50 (1H, d, J = 1.9 Hz, C^{10}**H**), 7.42-7.32 (5H, m, 2 x C^{17}**H** + 2 x C^{18}**H** + C^{19}**H**), 7.29 (1H, dd, J = 8.5, 1.9 Hz, C^{12}**H**), 7.12 (1H, dd, J = 4.6, 2.4 Hz, C^{3}**H**), 5.15 (1H, br s, O**H**), 4.09 (1H, ddd, J = 10.6, 2.9, 2.1 Hz, C^7**H**$_2$), 3.99 (1H, dd, J = 10.6, 2.7 Hz, C^7**H**$_2$), 3.69 (2H, s, C^{15}**H**$_2$), 3.59 (1H, dd, J = 19.0, 4.6 Hz, C^2**H**$_2$), 3.20 (1H, dt, J = 11.4, 1.5 Hz, C^6**H**$_2$), 3.10-3.04 (1H, m, C^5**H**), 2.98 (1H, dt, J = 19.0, 2.4 Hz, C^2**H**$_2$), 2.67 (1H, ddd, J = 11.2, 3.6, 2.1 Hz, C^6**H**$_2$);
^{13}C NMR ($CDCl_3$) δ 162.7 (C), 150.4 (C), 140.5 (C), 136.6 (C), 134.3 (C^3), 130.7 (C), 129.1 (2 x $C^{17\text{ or }18}$), 128.7 (2 x $C^{17\text{ or }18}$), 127.7 (C^{19}), 125.0 (C^{12}), 124.9 (C), 120.4 (C^{13}), 111.0 (C^{10}), 66.4 (C^7), 62.6 (C^{15}), 55.1 (C^6), 53.0 (C^2), 36.7 (C^5);
IR (neat) (cm^{-1}): 2925, 1634, 1541, 1512, 1451, 1240, 1071, 1024, 987, 918, 818, 749, 726, 695.

(3-Methoxy-4-(5-methylbenzoxazole)-*N*-benzyl-1,2,3,6-tetrahydropyridin-3-yl)methanol (2g)

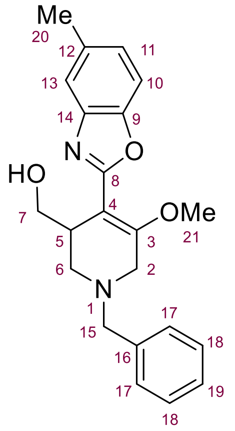

The title compound was prepared according to General Procedure C using salt **X** (229 mg, 0.5 mmol) at 45 °C for 16 hours. The product was purified by FCC (10%-30% EtOAc in pentane) to give *amine* **2g** (129 mg, 71%) as a yellow solid.
m.p. (acetone): 97-99 °C;
HRMS (ESI): Exact mass calculated for $C_{22}H_{25}O_3N_2$ $[M+H]^+$: 365.18597, found: 365.18588;
^1H NMR ($CDCl_3$) δ 7.47 (1H, s, C^{13}**H**), 7.41-7.30 (6H, m, C^{10}**H** + 2 x C^{17}**H** + 2 x C^{18}**H** + C^{19}**H**), 7.10 (1H, dd, J = 8.3, 1.6 Hz, C^{11}**H**), 4.73 (1H, br s, O**H**), 3.95-3.88 (2H, m, C^7**H**$_2$), 3.80 (3H, s, C^{21}**H**$_3$), 3.72-3.61 (3H, m, 2 x C^{15}**H**$_2$ + C^2**H**$_2$), 3.17-3.13 (2H, m, C^5**H** + C^6**H**$_2$), 2.96 (1H, dd, J = 16.8, 1.8 Hz, C^2**H**$_2$), 2.61 (1H, ddd, J = 11.5, 4.0, 1.4 Hz, C^6**H**$_2$), 2.46 (3H, s, C^{20}**H**$_2$);
^{13}C NMR ($CDCl_3$) δ 162.2 (C), 158.7 (C), 148.4 (C), 141.5 (C), 136.5 (C), 133.8 (C), 129.0 (2 x C^{17}), 128.7 (2 x C^{18}), 127.8 (C^{19}), 125.5 (C^{11}), 119.4 (C^{13}), 109.7 (C^{10}), 103.3 (C^4), 66.7 (C^7), 62.5 (C^{15}), 57.0 (C^{21}), 54.9 (C^6), 52.5 (C^2), 37.2 (C^3), 21.5 (C^{20});
IR (neat) (cm^{-1}): 2925, 1633, 1510, 1453, 1408, 1374, 1341, 1301, 1239, 1190, 1146, 1110, 1060, 1023, 986, 957, 886.

(3-Benzoxy-4-(5-methylbenzoxazole)-*N*-benzyl-1,2,3,6-tetrahydropyridin-3-yl)methanol (2h)

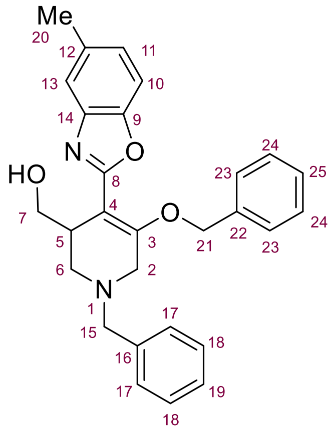

The title compound was prepared according to General Procedure C using salt **X** (267 mg, 0.5 mmol) at 45 °C for 16 hours. The product was purified by FCC (10%-30% EtOAc in pentane) to give *amine* **2h** (158 mg, 72%) as a yellow solid.
m.p. (acetone): 141-143 °C;
HRMS (ESI): Exact mass calculated for $C_{28}H_{29}O_3N_2$ $[M+H]^+$: 441.21727, found: 441.21719;
^1H NMR ($CDCl_3$) δ 7.48 (1H, d, J = 1.4 Hz, C^{13}**H**), 7.46-7.42 (2H, m, 2 x Ar**H**), 7.40-7.30 (9H, m, 9 x Ar**H**), 7.11 (1H, dd, J = 8.2, 1.6 Hz, Ar**H**), 5.06-4.99 (2H, m, C^{21}**H**$_2$), 4.91 (1H, br s, O**H**), 3.97 (1H, ddd, J = 10.6, 3.5, 1.6 Hz, C^7**H**), 3.92 (1H, dd, J = 10.6, 3.0 Hz, C^7**H**$_2$), 3.71 (1H, d, J = 12.8 Hz, C^{15}**H**$_2$), 3.65 (1H, d, J = 12.8 Hz, C^{15}**H**$_2$), 3,63 (1H, d, J = 16.9 Hz, C^6**H**$_2$),

3.22-3.18 (1H, m, C^{5}**H**), 3.15 (1H, d, *J* = 11.3 Hz, C^{2}**H**$_2$), 2.94 (1H, dd, *J* = 16.9, 1.9 Hz, C^{6}**H**$_2$), 2.61 (1H, ddd, *J* = 11.2, 3.7, 1.5 Hz, C^{2}**H**$_2$), 2.47 (3H, s, C^{20}**H**$_3$);
^{13}C NMR ($CDCl_3$) δ 162.1 (C), 158.1 (C), 148.6 (C), 141.5 (C), 136.7 (C), 136.4 (C), 133.9 (C), 129.0 (2 x ArC), 128.7 (2 x ArC), 128.4 (2 x ArC), 128.1 (ArC), 127.8 (ArC), 127.5 (2 x ArC), 125.7 (ArC), 119.4 (C^{13}), 109.7 (ArC), 105.8 (C), 72.2 (C^{21}), 66.6 (C^{7}), 62.4 (C^{15}), 54.9 (C^{2}), 53.4 (C^{6}), 37.4 (C^{5}), 21.5 (C^{20});
IR (neat) (cm^{-1}): 2929, 2860, 2163, 1648, 1607, 1512, 1454, 1434, 1389, 1363, 1298, 1265, 1250, 1226, 1174, 1103, 1081.

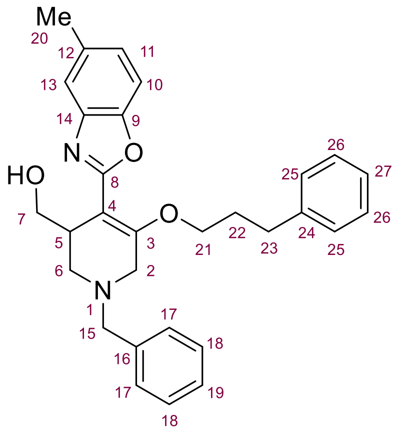

(3-(3-Phenylpropoxy)-4-(5-methylbenzoxazole)-*N*-benzyl-1,2,3,6-tetrahydropyridin-3-yl)methanol (2i)

The title compound was prepared according to General Procedure C using salt **1i** (281 mg, 0.5 mmol) at 45 °C for 16 hours. The product was purified by FCC (10%-30% EtOAc in pentane) to give *amine* **2i** (165 mg, 71%) as a yellow oil.
HRMS (ESI): Exact mass calculated for $C_{30}H_{33}O_3N_2$ $[M+H]^+$: 469.24857, found: 469.24850;
^{1}H NMR ($CDCl_3$) δ 7.47 (1H, dt, *J* = 1.7, 0.8 Hz, C^{13}**H**), 7.40-7.27 (8H, m, 8 x Ar**H**), 7.24-7.16 (3H, m, 3 x Ar**H**), 7.09 (1H, dd, *J* = 8.2, 1.6 Hz, C^{11}**H**), 4.83 (1H, br s, O**H**), 4.00-3.90 (4H, m, C^{7}**H**$_2$ + C^{21}**H**$_2$), 3.75-3.66 (2H, C^{15}**H**$_2$), 3.61 (1H, d, *J* = 16.7 Hz, C^{6}**H**$_2$), 3.21-3.18 (1H, m, C^{5}**H**), 3.16 (1H, d, *J* = 12.2 Hz, C^{2}**H**$_2$), 2.94 (1H, dd, *J* = 16.8, 1.7 Hz, C^{6}**H**$_2$), 2.90-2.85 (2H, m, C^{23}**H**$_2$), 2.62 (1H, ddd, *J* = 11.0, 3.5, 1.4 Hz, C^{2}**H**$_2$), 2.46 (3H, s, C^{20}**H**$_3$), 2.06 (2H, dtd, *J* = 8.8, 7.4, 6.0 Hz, C^{22}**H**$_2$);
^{13}C NMR ($CDCl_3$) δ 162.5 (C), 158.2 (C), 148.5 (C), 141.5 (2 x C), 136.4 (C), 133.8 (C), 129.0 (2 x ArC), 128.7 (2 x ArC), 128.5 (2 x ArC), 128.4 (2 x ArC), 127.8 (C$^{19/27}$), 125.9 (C$^{19/27}$), 125.6 (C^{11}), 119.3 (C^{13}), 109.5 (C^{10}), 103.9 (C), 68.5 (C$^{7/21}$), 66.7 (C$^{7/21}$), 62.5 (C^{15}), 54.9 (C^{2}), 53.0 (C^{6}), 37.3 (C^{5}), 32.0 (C^{23}), 31.5 (C^{22}), 21.5 (C^{20});
IR (neat) (cm^{-1}): 2922, 1638, 1516, 1496, 1454, 1403, 1379, 1299, 1264, 1227, 1184, 1105, 1018, 914, 870, 798, 744.

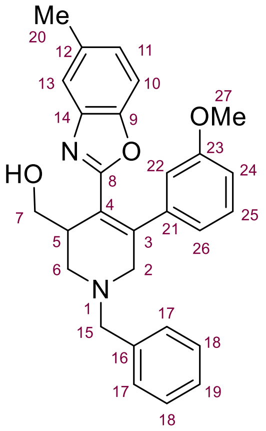

(3-(3-Methoxyphenyl)-4-(5-methylbenzoxazole)-*N*-benzyl-1,2,3,6-tetrahydropyridin-3-yl)methanol (2j)

The title compound was prepared according to General Procedure C using salt **1j** (267 mg, 0.5 mmol) at 45 °C for 16 hours. The product was purified by FCC (10%-30% EtOAc in pentane) to give *amine* **2j** (116 mg, 53%) as an orange oil.
HRMS (ESI): Exact mass calculated for $C_{28}H_{29}O_3N_2$ $[M+H]^+$: 441.21727, found: 441.21697;
^{1}H NMR ($CDCl_3$) δ 7.42 (1H, dt, *J* = 1.6, 0.8 Hz, C^{13}**H**), 7.40-7.36 (4H, m, 2 x C^{17}**H** + 2 x C^{18}**H**), 7.38 (1H, dd, *J* = 8.0, 4.2 Hz, C^{19}**H**), 7.19 (1H, dd, *J* = 8.3, 7.5 Hz, C^{25}**H**), 7.04-7.00 (2H, m, C^{10}**H** + C^{11}**H**), 6.85 (1H, ddd, *J* = 8.3, 2.5, 1.0 Hz, C$^{24/26}$**H**), 6.77-6.73 (2H, m, C^{22}**H** + C$^{24/26}$**H**), 5.37 (1H, br s, O**H**), 3.99-3.93 (2H, m, C^{7}**H**$_2$), 3.84-3.73 (3H, m, C^{15}**H**$_2$ + C^{6}**H**$_2$), 3.71 (3H, s, C^{27}**H**$_3$), 3.35-3.30 (2H, m, C^{5}**H** + C^{2}**H**$_2$), 3.09 (1H, d, *J* = 18.3 Hz, C^{6}**H**$_2$), 2.79 (1H, d, *J* = 11.0 Hz, C^{2}**H**$_2$), 2,42 (3H, d, *J* = 0.7 Hz, C^{20}**H**$_3$);
^{13}C NMR ($CDCl_3$) δ 159.4 (C), 148.4 (C), 141.3 (C), 134.0 (C), 129.3 (2 x C^{17}), 129.2 (C^{25}), 128.7 (2 x C^{18}), 127.9 (C^{19}), 126.1 (C^{11}), 121.7 (C), 120.6 (C^{24}), 119.6 (C^{13}), 113.8 (C$^{22/26}$), 113.4 (C$^{22/26}$), 109.8 (C^{10}), 66.3 (C^{7}), 62.4 (C^{15}), 58.7 (C^{6}), 55.3 (C^{27}), 54.6 (C^{2}), 39.0 (C^{5}), 21.4 (C^{20}), 4 quaternary aromatic carbons are not observed.
IR (neat) (cm^{-1}): 3433, 2970, 2161, 2033, 1652, 1585, 1540, 1510, 1453, 1419, 1360, 1269, 1243, 1220, 1178, 1149, 1078.

(4-(Benzoxazole)-*N*-*para*-methoxybenzyl-1,2,3,6-tetrahydropyridin-3-yl)methanol (2k)

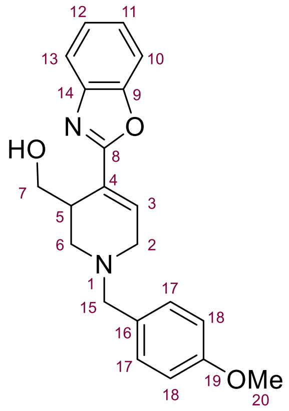

The title compound was prepared according to General Procedure C using salt **1k** (222 mg, 0.5 mmol) at 45 °C for 16 hours. The product was purified by FCC (20%-30% EtOAc in pentane) to give *amine* **2k** (119 mg, 68%) as a light brown solid.

m.p. (acetone): 71-73 °C;

HRMS (ESI): Exact mass calculated for $C_{21}H_{23}O_3N_2$ $[M+H]^+$: 351.17032, found: 351.17023;

^{1}H NMR ($CDCl_3$) δ 7.77-7.61 (1H, m, C$^{10/13}$**H**), 7.53-7.42 (1H, m, C$^{10/13}$**H**), 7.37-7.29 (2H, m, C^{11}**H** + C^{12}**H**), 7.26 (2H, d, *J* = 8.3 Hz, 2 x C^{17}**H**), 7.13 (1H, dd, *J* = 4.7, 2.4 Hz, C^3**H**), 6.91 (2H, d, *J* = 8.6 Hz, 2 x C^{18}**H**), 4.10 (1H, dt, *J* = 10.5, 2.5 Hz, C^7**H**$_2$), 3.99 (1H, dd, *J* = 10.5, 2.7 Hz, C^7**H**$_2$), 3.83 (3H, s, C^{20}**H**$_3$), 3.67-3.53 (3H, m, C^{15}**H**$_2$ + C^2**H**$_2$), 3.18 (1H, dt, *J* = 11.3, 1.4 Hz, C^6**H**$_2$), 3.10 (1H, q, *J* = 2.7, 2.1 Hz, C^5**H**), 2.96 (1H, dt, *J* = 18.9, 2.5 Hz, C^2**H**$_2$), 2.64 (1H, ddd, *J* = 11.3, 3.6, 2.1 Hz, C^6**H**$_2$);

^{13}C NMR (($CDCl_3$) δ 162.1 (C), 159.1 (C), 150.2 (C), 141.7 (C), 133.7 (C^3), 130.2 (2 x C^{17}), 128.7 (C), 125.2 (C), 125.1 (C$^{11/12}$), 124.3 (C$^{11/12}$), 119.9 (C$^{10/13}$), 113.9 (2 x C^{18}), 110.3 (C$^{10/13}$), 66.5 (C^7), 61.8 (C^{18}), 55.3 (C^6), 54.9 (C^{20}), 52.9 (C^2), 36.7 (C^5);

IR (neat) (cm^{-1}): 2926, 2161, 1656, 1610, 1540, 1513, 1455, 1342, 1304, 1241, 1188, 1133, 1070, 1024, 988, 956, 840.

(4-(Benzoxazole)-*N*-butyl-1,2,3,6-tetrahydropyridin-3-yl)methanol (2l)

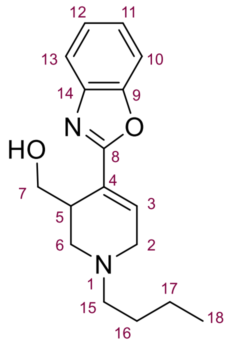

The title compound was prepared according to General Procedure C using salt **1l** (190 mg, 0.5 mmol) at 45 °C for 16 hours. The product was purified by FCC (10%-15% Et_2O in CH_2Cl_2) to give *amine* **2l** (103 mg, 72%) as a light brown glass.

m.p. (acetone): 137-139 °C;

HRMS (ESI): Exact mass calculated for $C_{17}H_{23}O_2N_2$ $[M+H]^+$: 287.17540, found: 287.17535;

^{1}H NMR ($CDCl_3$) δ 7.74-7.62 (1H, m, C$^{10/13}$**H**), 7.53-7.46 (1H, m, C$^{10/13}$**H**), 7.35-7.28 (2H, C^{11}**H** + C^{12}**H**), 7.15 (1H, dd, *J* = 4.6, 2.4 Hz, C^3**H**), 5.30 (1H, br s, O**H**), 4.14 (1H, dt, *J* = 10.5, 2.4 Hz, C^7**H**$_2$), 4.08 (1H, dd, *J* = 10.5, 2.7 Hz, C^7**H**$_2$), 3.65 (1H, dd, *J* = 18.8, 4.6 Hz, C^2**H**$_2$), 3.18 (1H, dt, *J* = 11.3, 1.5 Hz, C^6**H**$_2$), 3.10 (1H, dq, *J* = 4.5, 1.5 Hz, C^5**H**), 2.92 (1H, dt, *J* = 18.8, 2.5 Hz, C^2**H**$_2$), 2.63 (1H, ddd, *J* = 11.2, 3.5, 2.1 Hz, C^6**H**$_2$), 2.57-2.44 (2H, m, C^{15}**H**$_2$), 1.65-1.51 (2H, m, C^{16}**H**$_2$), 1.39 (2H, dt, *J* = 15.0, 7.4 Hz, C^{17}**H**$_2$), 0.95 (3H, t, *J* = 7.3 Hz, C^{18}**H**$_3$);

^{13}C NMR (($CDCl_3$) δ 162.1 (C), 150.3 (C), 141.8 (C), 133.8 (C^3), 125.3 (C), 125.1 (C$^{11/12}$), 124.3 (C$^{11/12}$), 119.9 (C$^{10/13}$), 110.3 (C$^{10/13}$), 66.6 (C^7), 57.6 (C^{15}), 55.7 (C^6), 53.0 (C^2), 36.6 (C^5), 28.9 (C^{16}), 20.5 (C^{17}), 14.0 (C^{18});

IR (neat) (cm^{-1}): 2916, 1634, 1541, 1512, 1455, 1373, 1238, 1177, 1092, 1052, 1026, 989, 971, 918, 844, 799, 764.

(4-(Benzoxazole)-*N*-(3-tosylpropyl)-1,2,3,6-tetrahydropyridin-3-yl)methanol (2m)

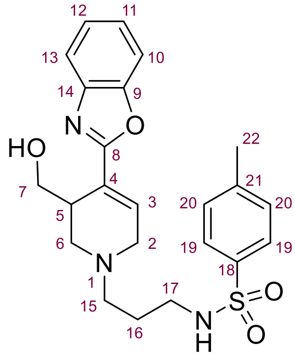

The title compound was prepared according to General Procedure C using salt **1m** (289.5 mg, 0.5 mmol) at 45 °C for 16 hours. The product was purified by FCC (10-40% EtOAc;pentane) to give *amine* **2m** (162 mg, 73%) as a sticky yellow gum.

HRMS (ESI): Exact mass calculated for $C_{23}H_{28}O_4N_3{}^{32}S$ $[M+H]^+$: 442.18005, found: 442.17935;

^{1}H NMR ($CDCl_3$) δ 7.76 (2H, d, *J* = 8.3 Hz, 2 x C^{19}**H**), 7.71-7.67 (1H, m, C$^{10/13}$**H**), 7.51-7.48 (1H, m, C$^{10/13}$**H**), 7.36-7.28 (4H, m, 2 x C^{20} + C^{11} + C^{12}), 7.08 (1H, dd, *J* = 4.4, 2.5 Hz, C^3**H**), 5.57 (1H, br s, O**H**), 4.03 (2H, d, *J* = 3.6 Hz, C^7**H**$_2$), 3.60 (1H, dd, *J* = 18.7, 4.5 Hz, C^2**H**$_2$), 3.17 (1H, dd, *J* = 11.1, 2.5 Hz, C^6**H**$_2$), 3.13-3.09 (1H, m, C^5**H**), 3.04

(1H, t, *J* = 6.4 Hz, C^{15}**H**$_2$), 2.93 (1H, dt, *J* = 18.8, 2.5 Hz, C^{2}**H**$_2$), 2.62-2.52 (3H, m, C^{6}**H**$_2$ + C^{17}**H**$_2$), 2.42 (3H, s, C^{22}**H**$_3$), 1.79 (2H, pd, *J* = 5.9, 1.1 Hz, C^{16}**H**$_2$);

^{13}C NMR ($CDCl_3$) δ 162.1 (C), 150.2 (C), 143.3 (C), 141.6 (C), 136.9 (C), 133.2 (C^{3}), 129.7 (2 x C^{20}), 127.1 (2 x C^{19}), 125.4 (C), 125.2 (C$^{11/12}$), 124.4 (C$^{11/12}$), 119.9 (C$^{10/13}$), 110.3 (C$^{10/13}$), 65.6 (C^{7}), 55.7 (C^{17}), 54.2 (C^{6}), 53.1 (C^{2}), 41.9 (C^{15}), 37.2 (C^{5}), 26.2 (C^{16}), 21.5 (C^{22});

IR (neat) (cm^{-1}): 3263, 2970, 1654, 1598, 1540, 1454, 1322, 1242, 1155, 1092, 1034, 981, 815, 763, 746, 659.

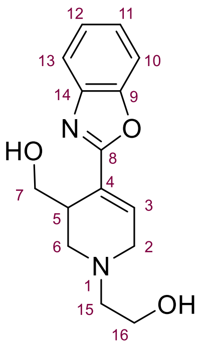

(4-(Benzoxazole)-*N*-(2-ethan-1-ol)-1,2,3,6-tetrahydropyridin-3-yl)methanol (2n)

The title compound was prepared according to General Procedure C using salt **1n** (184 mg, 0.5 mmol) at 45 °C for 16 hours. The product was purified by FCC (0-4% MeOH in CH_2Cl_2) to give *amine* **2n** (99 mg, 72%) as a peach solid.

m.p. (acetone): 128-130 °C;

HRMS (ESI): Exact mass calculated for $C_{15}H_{19}O_3N_2$ $[M+H]^+$: 275.14012, found: 275.13901;

^{1}H NMR ($CDCl_3$) δ 7.71-7.68 (1H, m, C$^{10/13}$**H**), 7.51-7.47 (1H, m, C$^{10/13}$**H**), 7.35-7.29 (2H, m, 2 x C$^{11/12}$**H**), 7.12 (1H, dd, *J* = 4.6, 2.3 Hz, C^{3}**H**), 4.05 (2H, d, *J* = 3.6 Hz, C^{7}**H**$_2$), 3.78 (2H, dtd, *J* = 17.3, 11.5, 4.7 Hz, C^{16}**H**$_2$), 3.65 (1H, dd, *J* = 18.2, 4.3 Hz, C^{2}**H**$_2$), 3.22 (1H, dd, *J* = 10.9, 1.7 Hz, C^{6}**H**$_2$), 3.12-3.04 (2H, m, C^{2}**H**$_2$ + C^{5}**H**), 2.73-2.64 (3H, m, C^{6}**H**$_2$ + 2 x C^{15}**H**$_2$);

^{13}C NMR (($CDCl_3$) δ 162.2 (C), 150.2 (C), 141.6 (C)¸133.8 (C^{3}), 125.5 (C), 125.2 (C$^{11/12}$), 124.4 (C$^{11/12}$), 119.9 (C$^{10/13}$), 110.3 (C$^{10/13}$), 66.0 (C^{7}), 59.1 (C^{16}), 59.0 (C^{15}), 54.1 (C^{6}), 53.3 (C^{2}), 37.4 (C^{5});

IR (neat) (cm^{-1}): 3306, 2915, 2161, 2032, 1654, 1538, 1472, 1452, 1357, 1299, 1263, 1238, 1181, 1145, 1104, 1083, 630, 511.

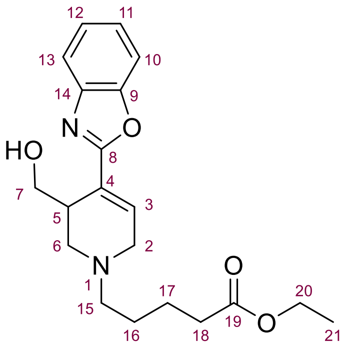

(4-(Benzoxazole)-*N*-ethylpentanoate-1,2,3,6-tetrahydropyridin-3-yl)methanol (2o)

The title compound was prepared according to General Procedure C using salt **1o** (233mg, 0.5 mmol) at 45 °C for 16 hours. The product was purified by FCC (20-50% EtOAc:pentane) to give *amine* **2o** (135 mg, 73%) as an orange oil.

HRMS (ESI): Exact mass calculated for $C_{20}H_{27}O_4N_2$ $[M+H]^+$: 359.19653, found: 359.19659;

^{1}H NMR ($CDCl_3$) δ 7.74-7.64 (1H, m, C$^{10/13}$**H**), 7.54-7.44 (1H, m, C$^{10/13}$**H**), 7.35-7.28 (2H, m, C^{111}**H** + C^{12}**H**), 7.14 (1H, dd, *J* = 4.6, 2.4 Hz, C^{3}**H**), 5.30 (1H, br s, O**H**), 4.18-4.09 (3H, m, 2 x C^{20}**H**$_2$ + C^{7}**H**$_2$), 4.06 (1H, dd, *J* = 10.5, 2.8 Hz, C^{7}**H**$_2$), 3.65 (1H, dd, *J* = 18.7, 4.6 Hz, C^{2}**H**$_2$), 3.18 (1H, dt, *J* = 11.2, 1.4 Hz, C^{6}**H**$_2$), 3.10 (1H, br q, *J* = 2.3 Hz, C^{5}**H**), 2.92 (1H, dt, *J* = 18.8, 2.5 Hz, C^{2}**H**$_2$), 2.62 (1H, ddd, *J* = 11.3, 3.5, 2.0 Hz, C^{6}**H**$_2$), 2.52 (2H, td, *J* = 7.0, 6.5, 1.7 Hz, 2 x C^{15}**H**$_2$), 2.35 (2H, t, *J* = 6.9 Hz, 2 x C^{18}**H**$_2$), 1.73-1.58 (4H, m, 2 x C^{16}**H**$_2$ + 2 x C^{17}**H**$_2$), 1.27 (3H, t, *J* = 7.1 Hz, 3 x C^{21}**H**$_3$);

^{13}C NMR (($CDCl_3$) δ 173.4 (C^{19}), 162.1 (C), 150.2 (C), 141.7 (C), 133.6 (C^{3}), 125.2 (C), 125.1 (C$^{11/12}$), 124.3 (C$^{11/12}$), 119.9 (C$^{10/13}$), 110.3 (C$^{10/13}$), 66.5 (C^{7}), 60.3 (C^{20}), 57.5 (C^{15}), 55.4 (C^{6}), 53.0 (C^{2}), 36.7 (C^{5}), 33.9 (C^{18}), 26.3 (C$^{16/17}$), 22.6 (C$^{16/17}$), 14.2 (C^{21});

IR (neat) (cm^{-1}): 2980, 1728, 1638, 1540, 1454, 1373, 1242, 1181, 1029, 747, 695, 623.

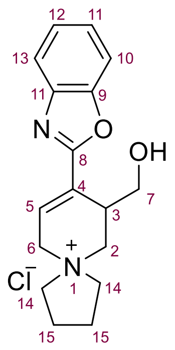

8-(Benzoxazole)-9-(hydroxymethyl)-5-azaspiro[4.5]dec-7-en-5-ium Chloride (2p)

The title compound was prepared according to modified General Procedure C using salt **1p** (207.3 mg, 0.5 mmol) at 45 °C for 16 hours. The reaction was concentrated *in vacuo* and was intermediately purified by FCC (0-5% MeOH:CH_2Cl_2) to give *amine* **2p** as a hydroscopic yellow solid. Due to its propensity to readily incorporate water, 0.5 mmol of trimethoxybenzene was added to the sample and quantitative ^{1}H NMR analysis indicated 0.365 mmol of **2p** (117 mg, 73%).

HRMS (ESI): Exact mass calculated for $C_{17}H_{21}O_2N_2$ $[M]^+$: 285.15975, found: 285.15964;

^{1}H NMR (CD_3OD) δ 7.75-7.71 (1H, m, C^{13}**H**), 7.65-7.61 (1H, m, C^{10} **H**), 7.47-7.37 (2H, m, 2 x $C^{11\text{ or }12}$**H**), 7.11 (1H, dd, *J* = 4.9, 2.4 Hz, C^5**H**), 4.45-4.35 (3H, m), 4.09-3.97 (2H, m, 2 x C^7**H**$_2$), 3.90-3.65 (5H, m), 3.59-3.51 (1H, m, C^3**H**), 2.38-2.30 (4H, m, 4 x C^{15}**H**$_2$);

^{13}C NMR (CD_3OD) δ 161.3 (C), 151.2 (C), 142.3 (C), 129.1 (C^3), 127.4 (C), 127.2 ($C^{11/12}$), 125.9 ($C^{11/12}$), 121.0 (C^{13}), 111.6 (C^{10}), 67.7 (C^7), 61.6 (CH_2), 61.2 (CH_2), 60.3 (CH_2), 59.5 (CH_2), 37.1 (C^3), 23.0 (C^{15}), 22.6 (C^{15});

IR (neat) (cm^{-1}): 3392, 2521, 1610, 1540, 1513, 1454, 1303, 1241, 1190, 1152, 1070, 1024, 988, 955, 824.

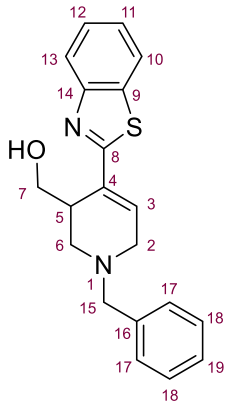

(4-(Benzothiazole)-*N*-benzyl-1,2,3,6-tetrahydropyridin-3-yl)methanol (2q)

The title compound was prepared according to General Procedure C using salt **1q** (215 mg, 0.5 mmol) at 45 °C for 16 hours. The product was purified by FCC (10%-30% EtOAc in pentane) to give *amine* **2q** (111 mg, 66%) as an orange solid.

m.p. (acetone): 97-99 °C;

HRMS (ESI): Exact mass calculated for $C_{20}H_{21}ON_2S$ $[M+H]^+$: 337.1369, found: 337.1369;

^{1}H NMR ($CDCl_3$) δ 7.96 (1H, dt, *J* = 8.3, 0.9 Hz, $C^{10/13}$**H**), 7.84-7.81 (1H, m, $C^{10/13}$**H**), 7.44 (1H, ddd, *J* = 8.3, 7.2, 1.3 Hz, $C^{11/12}$**H**), 7.40-7.30 (6H, m, $C^{11/12}$**H** + 2 x C^{17}**H** + 2 x C^{18}**H** + C^{19}**H**), 6.75 (1H, dd, *J* = 4.6, 2.4 Hz, C^3**H**), 5.18 (1H, br s, O**H**), 4.06 (1H, ddd, *J* = 10.5, 3.0, 1.9 Hz, C^7**H**$_2$), 4.00 (1H, dd, *J* = 10.5, 3.1 Hz, C^7**H**$_2$), 3.70 (1H, d, *J* = 12.8 Hz, C^{15}**H**$_2$), 3.66 (1H, d, *J* = 12.8 Hz, C^{15}**H**$_2$), 3.54 (1H, dd, *J* = 18.6, 4.6 Hz, C^2**H**$_2$), 3.27 (1H, q, *J* = 2.7 Hz, C^5**H**), 3.19 (1H, dt, *J* = 11.2, 1.5 Hz, C^6**H**$_2$), 2.96 (1H, d, *J* = 18.6, 2.5 Hz, C^2**H**$_2$), 2.68 (1H, ddd, *J* = 11.2, 3.6, 1.8 Hz, C^6**H**$_2$);

^{13}C NMR (($CDCl_3$) δ 168.0 (C), 153.6 (C),136.9 (C), 134.2 (C), 132.7 (C), 132.3 (C^3), 129.0 (2 x $C^{17/18}$), 128.6 (2 x $C^{17/18}$), 127.6 (C^{19}), 126.0 ($C^{11/12}$), 125.3 ($C^{11/12}$), 123.0 ($C^{10/13}$), 121.4 ($C^{10/13}$), 66.6 (C^7), 62.6 (C^{15}), 55.3 (C^6), 53.1 (C^2), 38.1 (C^5);

IR (neat) (cm^{-1}): 3057, 2911, 2853, 2162, 2034, 1650, 1491, 1456, 1435, 1360, 1313, 1263, 1204, 1158, 1124, 1088, 1069.

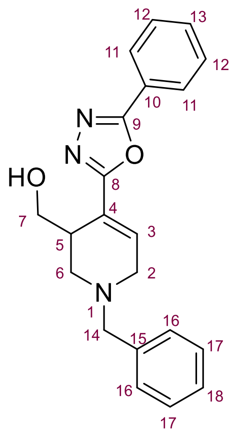

(4-(5-Phenyl-1,3,4-oxadiazol-2-yl)-*N*-benzyl-1,2,3,6-tetrahydropyridin-3-yl)methanol (2r)

The title compound was prepared according to General Procedure C using salt **1r** (221 mg, 0.5 mmol) at 45 °C for 16 hours. The product was purified by FCC (30-50% EtOAc in pentane) to give *amine* **2r** (99 mg, 57%) as a yellow oil.

HRMS (ESI): Exact mass calculated for $C_{21}H_{22}O_2N_3$ $[M+H]^+$: 348.17065, found: 348.17078;

^{1}H NMR ($CDCl_3$) δ 8.06 (2H, dd, *J* = 8.0, 1.7 Hz, 2 x C^{12}**H**), 7.56-7.47 (3H, m, 2 x C^{11}**H** + C^{13}**H**), 7.42-7.29 (5H, m, 2 x C^{16}**H** + 2 x C^{17}**H** + C^{18}**H**), 6.95 (1H, dd, *J* = 4.7, 2.3 Hz, C^3**H**), 5.07 (1H, br, O**H**), 4.11 (1H, dt, *J* = 10.7, 2.5 Hz, C^7**H**$_2$), 3.98 (1H, dd, *J* = 10.7, 2.5 Hz, C^7**H**$_2$), 3.69 (2H, s, C^{14}**H**$_2$), 3.58 (1H, dd, *J* = 18.8, 4.7 Hz, C^2**H**$_2$), 3.21 (1H, dt, *J* = 11.3, 1.3 Hz, C^6**H**$_2$), 3.09 (1H, q, *J* = 2.6 Hz, C^5**H**), 2.97 (1H, dt, *J* = 18.8, 2.5 Hz, C^2**H**$_2$), 2.68 (1H, ddd, *J* = 11.4, 3.6, 2.1 Hz, C^6**H**$_2$);

^{13}C NMR ($CDCl_3$) δ 164.1 (C), 163.4 (C), 136.5 (C), 132.8 (C^3), 131.7 (C^{13}), 129.1 (2 x C^{11}), 129.0 (2 x C^{17}), 128.7 (2 x C^{16}), 127.8 (C^{18}), 126.9 (2 x C^{12}), 123.8 (C), 122.4 (C), 66.2 (C^7), 62.6 (C^{14}), 55.0 (C^6), 52.7 (C^2), 36.5 (C^5);
IR (neat) (cm^{-1}): 2925, 1633, 1511, 1451, 1407, 1364, 1341, 1301, 1239, 1189, 1146, 1110, 1071, 1023, 986, 958, 915.

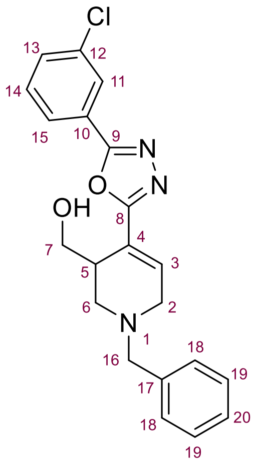

(4-(5-Phenyl-1,3,4-oxadiazol-2-yl)-*N*-benzyl-1,2,3,6-tetrahydropyridin-3-yl)methanol (2s)

The title compound was prepared according to General Procedure C using salt **1s** (237.75mg, 0.5 mmol) at 45 °C for 16 hours. The product was purified by FCC (10%-15% Et_2O in CH_2Cl_2) to give *amine* **2s** (100 mg, 52%) as a yellow solid.
m.p. (acetone): 115-117 °C;
HRMS (ESI): Exact mass calculated for $C_{21}H_{21}O_2N_3Cl$ $[M+H]^+$: 382.1317, found: 382.1317.
^1H NMR ($CDCl_3$) δ 8.04 (1H, t, *J* = 1.8 Hz, C^{11}**H**), 7.96 (1H, dt, *J* = 7.6, 1.4 Hz, C^{13}**H**), 7.52 (1H, ddd, *J* = 8.1, 2.1, 1.2 Hz, C^{15}**H**), 7.45 (1H, t, *J* = 7.9 Hz, C^{14}**H**), 7.41-7.30 (5H, m, 2 x C^{18}**H** + 2 x C^{19}**H** + C^{20}**H**), 6.97 (1H, dd, *J* = 4.6, 2.3 Hz, C^3**H**), 4.96 (1H, br s, O**H**), 4.09 (1H, ddd, *J* = 10.7, 3.1, 2.2 Hz, C^7**H**$_2$), 3.98 (1H, dd, *J* = 10.7, 2.5 Hz, C^7**H**$_2$), 3.70 (2H, s, C^{16}**H**$_2$), 3.59 (1H, dd, *J* = 18.8, 4.7 Hz, C^2**H**$_2$), 3.21 (1H, dt, *J* = 11.3, 1.4 Hz, C^6**H**$_2$), 3.08 (1H, br s, C^5**H**), 2.98 (1H, dt, *J* = 18.8, 2.4 Hz, C^2**H**$_2$), 2.68 (1H, ddd, *J* = 11.4, 3.6, 2.1 Hz, C^6**H**$_2$);
^{13}C NMR ($CDCl_3$) δ 163.7 (C), 162.9 (C), 136.5 (C), 135.2 (C), 133.4 (C^3), 131.7 (C^{15}), 130.4 (C^{14}), 129.1 (2 x $C^{18/19}$), 128.7 ($C^{18/19}$), 127.8 (C^{20}), 126.9 (C^{11}), 125.5 (C), 125.0 (C^{13}), 122.3 (C), 66.2 (C^7), 62.6 (C^{16}), 55.0 (C^6), 52.8 (C^2), 36.6 (C^5);
IR (neat) (cm^{-1}): 2843, 1657, 1580, 1547, 1524, 1428, 1367, 1306, 1260, 1141, 1073, 1041, 1008, 991, 966, 895, 839.

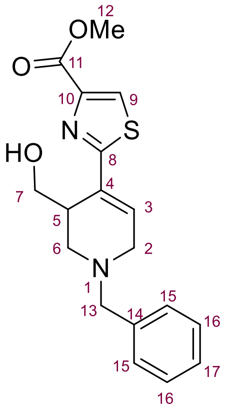

Methyl-2-(1-benzyl-3-(hydroxymethyl)-1,2,3,6-tetrahydropyridin-4-yl)thiazole-4-carboxylate (2t)

The title compound was prepared according to General Procedure C using salt **1t** (226 mg, 0.5 mmol) at 45 °C for 16 hours. The product was purified by FCC (30%-50% EtOAc in pentane) to give *amine* **2t** (93 mg, 54%) as an orange oil.
HRMS (ESI): Exact mass calculated for $C_{18}H_{21}O_3N_2S$ $[M+H]^+$: 345.12674, found: 345.12671;
^1H NMR ($CDCl_3$) δ 8.05 (1H, s, C^9**H**), 7.39-7.29 (5H, m, 2 x C^{15}**H** + 2 x C^{16}**H** + C^{17}**H**), 6.64 (1H, dd, *J* = 4.6, 2.4 Hz, C^3**H**), 5.11 (1H, s, O**H**), 3.95-2.93 (5H, m, 3 x C^{12}**H**$_3$ + 2 x C^7**H**$_2$), 3.68 (1H, d, *J* = 12.8 Hz, C^{13}**H**$_2$), 3.63 (1H, d, *J* = 12.8 Hz, C^{13}**H**$_2$), 3.48 (1H, dd, *J* = 18.5, 4.6 Hz, C^2**H**$_2$), 3.25-3.20 (1H, m, C^5**H**), 3.16 (1H, dt, *J* = 11.3, 1.4 Hz, C^6**H**$_2$), 2.91 (1H, dt, *J* = 18.5, 2.5 Hz, C^2**H**$_2$), 2.65 (1H, dd, *J* = 11.3, 3.6 Hz, C^6**H**$_2$);
^{13}C NMR ($CDCl_3$) δ 168.6 (C^{11}), 161.8 (C), 146.9 (C), 136.8 (C), 131.8 (C), 130.6 (C^3), 129.0 (2 x C^{16}), 128.6 (2 x C^{15}), 127.6 (C^{17}), 126.6 (C^9), 66.3 (C^7), 62.6 (C^{13}), 55.4 (C^6), 52.9 (C^2), 52.4 (C^{12}), 38.0 (C^5);
IR (neat) (cm^{-1}): 3381, 2970, 1723, 1648, 1464, 1344, 1212, 1161, 1130, 1099, 991, 951, 816, 736, 699, 656.

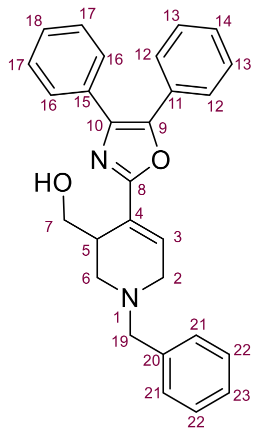

(4-(4,5-Diphenyloxa-2-zole)-*N*-benzyl-1,2,3,6-tetrahydropyridin-3-yl)methanol (2u)

The title compound was prepared according to General Procedure C using salt **1u** (129 mg, 0.25 mmol) at 45 °C for 16 hours. The product was purified by FCC (10%-30% EtOAc in pentane) to give *amine* **2u** (69 mg, 65%) as a sticky yellow semi-solid.

HRMS (ESI): Exact mass calculated for $C_{28}H_{27}O_2N_2$ $[M+H]^+$: 423.20670, found: 423.20621;

^{1}H NMR ($CDCl_3$) δ 7.66-7.62 (2H, m, 2 x Ar**H**), 7.61-7.57 (2H, m, 2 x Ar**H**), 7.40-7.30 (11H, m, 11 x Ar**H**), 6.92 (1H, dd, *J* = 4.6, 2.5 Hz, C^{3}**H**), 5.20 (1H, br s, O**H**), 4.12 (1H, ddd, *J* = 10.5, 2.9, 2.0 Hz, C^{7}**H**$_2$), 3.98 (1H, dd, *J* = 10.5, 3.1 Hz, C^{7}**H**$_2$), 3.68 (2H, s, C^{19}**H**$_2$), 3.54 (1H, dd, *J* = 18.5, 4.6 Hz, C^{2}**H**$_2$), 3.14 (1H, dt, *J* = 11.2, 1.4 Hz, C^{6}**H**$_2$), 3.08 (1H, q, *J* = 2.8 Hz, C^{5}**H**), 2.97 (1H, dt, *J* = 18.5, 2.5 Hz, C^{2}**H**$_2$), 2.66 (1H, ddd, *J* = 11.2, 3.7, 1.9 Hz, C^{6}**H**$_2$);

^{13}C NMR (($CDCl_3$) δ 159.5 (C), 145.0 (C), 136.9 (C), 136.1 (C), 132.5 (C), 130.0 (C^{3}), 129.0 (2 x ArC), 128.9 (C), 128.61 (4 x ArC), 128.56 (2 x ArC), 128.5 (ArC), 128.12 (ArC), 128.05 (2 x ArC), 127.6 (ArC), 126.5 (2 x ArC), 125.1 (C), 66.7 (C^{7}), 62.7 (C^{19}), 55.2 (C^{6}), 52.9 (C^{2}), 36.9 (C^{5});

IR (neat) (cm^{-1}): 2927, 1634, 1541, 1512, 1450, 1240, 1071, 1024, 988, 749, 692.

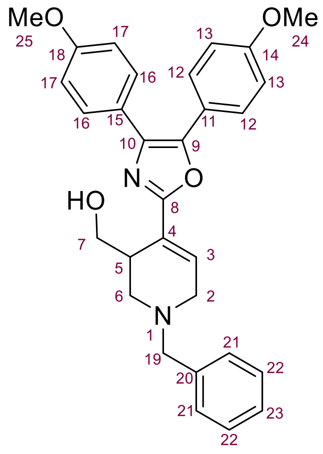

(4-(4,5-Di(4-methoxyphenyl)oxa-2-zole)-*N*-benzyl-1,2,3,6-tetrahydropyridin-3-yl)methanol (2v)

The title compound was prepared according to General Procedure C using salt **1v** (144 mg, 0.25 mmol) at 45 °C for 16 hours. The product was purified by FCC (20%-30% EtOAc in pentane) to give *amine* **2v** (74 mg, 62%) as an orange solid.

m.p. (acetone): 100-102 °C;

HRMS (ESI): Exact mass calculated for $C_{30}H_{31}O_4N_2$ $[M+H]^+$: 483.2284, found: 483.2283;

^{1}H NMR ($CDCl_3$) δ 7.53 (4H, app dd, *J* = 17.1, 8.8 Hz, 2 x C^{12}**H** + 2 x C^{16}**H**), 7.40-7.30 (5H, m, 2 x C^{21}**H** + 2 x C^{22}**H** + C^{23}**H**), 6.92-6.84 (5H, m, 2 x C^{13}**H** + 2 x C^{17}**H** + C^{3}**H**), 4.10 (1H, ddd, *J* = 10.5, 2.9, 1.8 Hz, C^{7}**H**$_2$), 3.97 (1H, dd, *J* = 10.5, 3.2 Hz, C^{7}**H**$_2$), 3.84 (6H, s, C^{24}**H**$_3$ + C^{25}**H**$_3$), 3.70 (2H, s, C^{19}**H**$_2$), 3.54 (1H, dd, *J* = 18.4, 4.6 Hz, C^{2}**H**$_2$), 3.14 (1H, d, *J* = 10.9 Hz, C^{6}**H**$_2$), 3.09-3.05 (1H, m, C^{5}**H**), 2.98 (1H, dt, *J* = 18.4, 2.6 Hz, C^{2}**H**$_2$), 2.67 (1H, ddd, *J* = 11.3, 3.8, 1.8 Hz, C^{6}**H**$_2$);

^{13}C NMR (($CDCl_3$) δ 159.7 (C$^{14/18}$), 159.4 (C$^{14/18}$), 158.8 (C), 157.7 (C), 144.4 (C), 134.7 (C)¸129.2 (2 x C$^{12/16}$ + C^{3}), 129.1 (2 x C^{21}), 128.6 (2 x C^{22}), 128.0 (2 x C$^{12/16}$), 127.7 (C^{23}), 125.3 (C)¸125.1 (C), 121.6 (C), 114.1 (C$^{13/17}$), 114.0 (C$^{13/17}$), 66.6 (C^{7}), 62.6 (C^{19}), 55.3 (C^{24} + C^{25}), 55.0 (C^{6}), 52.8 (C^{2}), 36.9 (C^{5});

IR (neat) (cm^{-1}): 2929, 2860, 2162, 2034, 1648, 1608, 1513, 1498, 1455, 1389, 1298, 1250, 1226, 1174, 1104, 1081, 1062, 1018.

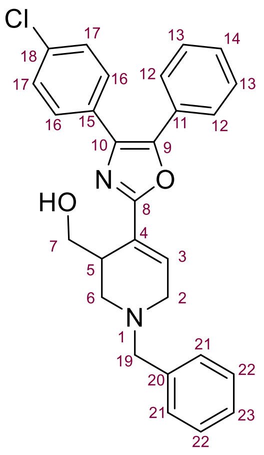

(4-(4-Phenyl-5-(4-chloro)phenyl)oxa-2-zole)-*N*-benzyl-1,2,3,6-tetrahydropyridin-3-yl)methanol (2w)

The title compound was prepared according to General Procedure C using salt **1w** (138 mg, 0.25 mmol) at 45 °C for 16 hours. The product was purified by FCC (10%-30% EtOAc in pentane) to give *amine* **2w** (57 mg, 50%) as an orange oil.

HRMS (ESI): Exact mass calculated for $C_{28}H_{26}O_2N_2{}^{35}Cl$ $[M+H]^+$: 457.16883, found: 457.16740;

^{1}H NMR ($CDCl_3$) δ 7.60-7.54 (4H, m, 4 x C^{Ar}), 7.41-7.31 (10H, m, 10 x C^{Ar}), 6.92 (1H, dd, *J* = 4.6, 2.4 Hz, C^3**H**), 5.27 (1H, br s, O**H**), 4.12 (1H, dt, *J* = 10.5, 2.4 Hz, C^7**H**$_2$), 3.99 (1H, dd, *J* = 10.5, 2.9 Hz, C^7**H**$_2$), 3.69 (2H, s, C^{19}**H**$_2$), 3.55 (1H, dd, *J* = 18.5, 4.6 Hz, C^2**H**$_2$), 3.16 (1H, dd, *J* = 11.4, 1.9 Hz, C^6**H**$_2$), 3.08-3.04 (1H, m, C^5**H**), 2.97 (1H, dt, *J* = 18.4, 2.5 Hz, C^2**H**$_2$), 2.67 (1H, ddd, *J* = 11.3, 3.7, 1.8 Hz, C^6**H**$_2$);

^{13}C NMR (($CDCl_3$) δ 159.5 (C)¸145.2 (C), 136.7 (C), 135.0 (C), 133.9 (C), 130.9 (C), 130.2 (C^3), 129.2 (2 x C^{Ar}), 129.0 (2 x C^{Ar}), 128.74 (2 x C^{Ar} + C^{Ar}), 128.71 (2 x C^{Ar}), 128.6 (2 x C^{Ar}), 127.7 (C^{Ar}), 127.6 (C), 126.6 (2 x C^{Ar}), 124.9 (C), 66.5 (C^7), 62.6 (C^{19}), 55.1 (C^6), 52.8 (C^2), 36.8 (C^5);

IR (neat) (cm^{-1}): 2928, 2162, 1648, 1499, 1454, 1389, 1364, 1298, 1225, 1174, 1091, 1063, 1017, 965, 908, 835, 817, 789.

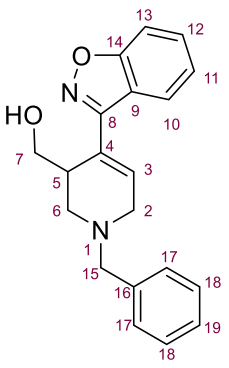

(4-(Benzisoxazole)-*N*-benzyl-1,2,3,6-tetrahydropyridin-3-yl)methanol (2x)

The title compound was prepared according to General Procedure C using salt **1w** (103.5 mg, 0.25 mmol) at 45 °C for 16 hours. The product was purified by FCC (10%-30% EtOAc in pentane) to give *amine* **2x** (30 mg, 38%) as a yellow solid.

m.p. (Et_2O): 85-87 °C;

HRMS (ESI): Exact mass calculated for $C_{20}H_{21}O_2N_2$ $[M+H]^+$: 321.15975, found: 321.15973;

^{1}H NMR ($CDCl_3$) δ 7.85 (1H, dt, *J* = 8.1, 1.0 Hz, C$^{10/13}$**H**), 7.60 (1H, dt, *J* = 8.5, 1.0 Hz, C$^{10/13}$**H**), 7.57-7.52 (1H, m, C$^{11/12}$), 7.41-.737 (4H, m, 2 x C^{17}**H** + 2 x C^{18}**H**), 7.36-7.31 (2H, m, C$^{11/12}$**H** + C^{19}**H**), 6.79 (1H, dd, *J* = 4.6, 2.3 Hz, C^3**H**), 4.00 (1H, ddd, *J* = 10.6, 3.2, 1.8 Hz, C^7**H**$_2$), 3.95 (1H, dd, *J* = 10.6, 2.7 Hz, C^7**H**$_2$), 3.80 (1H, d, *J* = 12.7 Hz, C^{15}**H**$_2$), 3.73 (1H, d, *J* = 12.7 Hz, C^{15}**H**$_2$), 3.64 (1H, dd, *J* = 18.0, 4.6 Hz, C^2**H**$_2$), 3.30 (1H, dt, *J* = 11.2, 1.5 Hz, C^6**H**$_2$), 3.24 (1H, br s, C^5**H**), 3.03 (1H, dt, *J* = 18.1, 2.5 Hz, C^2**H**$_2$), 2.79 (1H, ddd, *J* = 10.8, 3.3, 1.5 Hz, C^6**H**$_2$);

^{13}C NMR (($CDCl_3$) δ 163.5 (C), 155.5 (C), 130.5 (C^3), 129.6 (C$^{11/12}$), 129.3 (2 x C$^{17/18}$), 128.7 (2 x C$^{17/18}$), 127.9 (C^{19}), 127.8 (C), 123.7 (C$^{11/12}$), 122.4 (C$^{10/13}$), 120.8 (C)¸120.2 (C), 110.1 (C$^{10/13}$), 66.0 (C^7), 62.7 (C^{15}), 55.2 (C^6), 52.8 (C^2), 37.8 (C^5);

IR (neat) (cm^{-1}): 2971, 2161, 1638, 1609, 1493, 1452, 1392, 1337, 1237, 1140, 1070, 1046, 1002, 941, 902, 874, 820, 764.

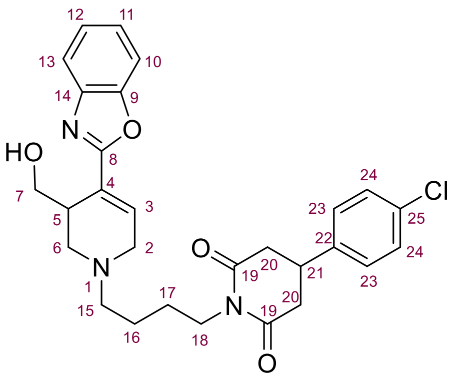

(4-(Benzoxazole)-*N*-(4-(4-Chlorophenyl)-1-(4-butyl)piperidine-2,6-dione)-1,2,3,6-tetrahydropyridin-3-yl)methanol (2aa)

The title compound was prepared according to General Procedure C using salt **1aa** (301 mg, 0.5 mmol) at 45 °C for 16 hours. The product was purified by FCC (30-60% EtOAc:pentane) to give *amine* **2aa** (176 mg, 69%) as a yellow solid.

m.p. (acetone): 158-160 °C;

HRMS (ESI): Exact mass calculated for $C_{28}H_{31}O_4N_3Cl$ $[M+H]^+$: 508.1998, found: 508.1994;

^{1}H NMR (CDCl$_3$) δ 7.71-7.68 (1H, m, C$^{11/12}$**H**), 7.51-7.48 (1H, m, C$^{10/11}$**H**), 7.36-7.29 (4H, m, 2 x C$^{10/13}$**H** + 2 x C^{23}**H**), 7.17-7.12 (3H, m, 2 x C^{24}**H** + C^{3}**H**), 4.12 (1H, ddd, *J* = 10.5, 3.0, 2.0 Hz, C^{7}**H$_2$**), 4.06 (1H, dd, *J* = 10.5, 2.8 Hz, C^{7}**H**$_2$), 3.85-3.80 (2H, m, C^{18}**H**$_2$), 3.64 (1H, dd, *J* = 18.8, 4.6 Hz, C^{2}**H**$_2$), 3.35 (1H, tt, *J* = 11.6, 4.3 Hz, C^{21}**H**), 3.17 (1H, dt, *J* = 11.2, 1.3 Hz, C^{6}**H**$_2$), 3.13-.3.09 (1H, m, C^{5}**H**), 2.98 (2H, dd, *J* = 17.3, 4.4 Hz,2 x C^{20}**H**$_2$), 2.92 (1H, dt, *J* = 18.7, 2.4 Hz, C^{2}**H**$_2$), 2.82-2.73 (2H, m, 2 x C^{20}**H**$_2$), 2.63 (1H, ddd, *J* = 11.3, 3.6, 2.0 Hz, C^{6}**H**$_2$),2.59-2.47 (2H, m, C^{15}**H**$_2$), 1.63-1.56 (4H, m, C^{16}**H**$_2$ + C^{17}**H**$_2$);

^{13}C NMR (CDCl$_3$) δ 171.3 (2 x C^{19}), 162.1 (C$_q$), 150.3 (C$_q$), 141.8 (C$_q$), 139.1 (C$_q$), 133.7 (C^{3}), 133.4 (C$_q$), 129.2 (2 x C^{23}), 127.7 (2 x C^{24}), 125.3 (C$_q$), 125.1 (C$^{10/13}$), 124.3 (C$^{10/13}$), 119.9 (C$^{11/12}$), 110.3 (C$^{11/12}$), 66.5 (C^{7}), 57.3 (C^{18}), 55.5 (C^{6}), 52.9 (C^{2}), 39.8 (C^{20}), 39.7 (C^{20}), 39.4 (C^{15}), 36.8 (C^{5}), 34.1 (C^{21}), 25.7 (C$^{16/17}$), 24.3 (C$^{16/17}$);

IR (neat) (cm^{-1}): 2922, 2868, 2161, 2033, 1719, 1668, 1544, 1494, 1475, 1452, 1395, 1355, 1297, 1262, 1241, 1190, 1142, 1113.

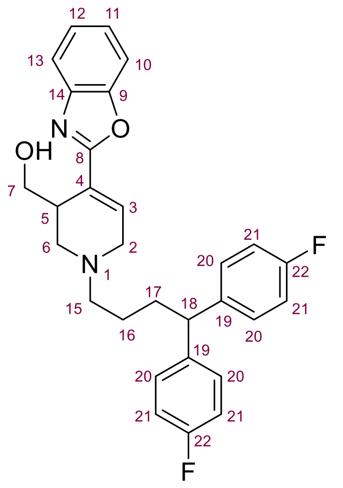

4-(Benzoxazole)-*N*-4-(4-*bis*(4-fluorophenyl)-1-butyl-4-1,2,3,6-tetrahydropyridin-3-yl)methanol (2ab)

The title compound was prepared according to General Procedure C using salt **1ab** (142 mg, 0.25 mmol) at 45 °C for 16 hours. The product was purified by FCC (10-30% EtOAc;pentane) to give *amine* **2ab** (85 mg, 72%) as a yellow gum.

m.p. (acetone): 127-129 °C;

HRMS (ESI): Exact mass calculated for $C_{29}H_{29}O_2N_2F_2$ $[M+H]^+$: 475.21916, found: 475.21884;

^{1}H NMR (CDCl$_3$) δ 7.72-7.68 (1H, m, C$^{10/13}$**H**), 7.51-7.47 (1H, m, C$^{10/13}$**H**), 7.35-7.29 (2H, m, 2 x C$^{11/12}$**H**), 7.22-7.15 (4H, m, 4 x C^{20}**H**), 7.11 (1H, dd, *J* = 4.6, 2.4 Hz, C^{3}**H**), 7.01-6.95 (4H, m, 4 x C^{21}**H**), 4.12 (1H, dt, *J* = 10.5, 2.3 Hz, C^{7}**H**$_2$), 4.05 (1H, dd, *J* = 10.5, 2.8 Hz, C^{7}**H**$_2$), 3.89 (1H, t, *J* = 7.8 Hz, C^{18}**H**), 3.54 (1H, dd, *J* = 18.7, 4.6 Hz, C^{2}**H**$_2$), 3.11-3.07 (2H, m, C^{5}**H** + C^{6}**H**$_2$), 2.85 (1H, dt, *J* = 18.7, 2.4 Hz, C^{2}**H**$_2$), 2.59 (1H, ddd, *J* = 11.6, 3.8, 2.1 Hz, C^{6}**H**$_2$), 2.52 (2H, q, *J* = 7.0 Hz, C^{15}**H**$_2$), 2.11-2.01 (2H, m, C^{17}**H**$_2$), 1.53 (2H, dq, *J* = 8.8, 7.0 Hz, C^{16}**H**$_2$);

^{13}C NMR (CDCl$_3$) δ 162.1 (C$_q$), 161.4 (d, *J* = 244.9 Hz, 2 x C^{22}), 150.3 (C$_q$), 141.7 (C$_q$), 140.3 (C$_q$), 133.5 (C^{3}), 129.0 (d, *J* = 7.9 Hz, 4 x C^{20}), 125.3 (C$_q$), 125.2 (C$^{11/12}$), 124.3 (C$^{11/12}$), 119.9 (C$^{10/13}$), 115.4 (d, *J* = 20.9 Hz, 4 x C^{21}), 110.3 (C$^{10/13}$), 66.6 (C^{7}), 57.7 (C^{15}), 55.7 (C^{6}), 52.9 (C^{2}), 49.8 (C^{18}), 36.7 (C^{5}), 33.6 (C^{17}), 25.2 (C^{16});

^{19}F NMR (CDCl$_3$) δ –116.8 (ddt, *J* = 11.3, 8.6, 5.3 Hz);

IR (neat) (cm^{-1}): 2929, 2853, 2164, 2034, 1652, 1601, 1541, 1505, 1458, 1376, 1342, 1300, 1242, 1216, 1155, 1115, 1088, 1026.

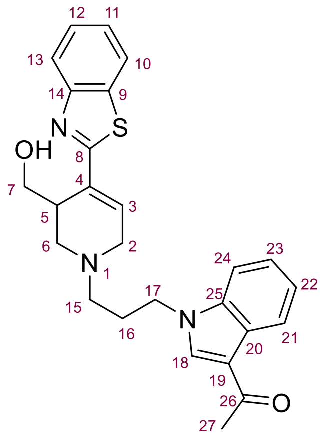

4-(Benzothiazole)-*N*-3-(3-acetylindole)-1-propyl-4-1,2,3,6-tetrahydropyridin-3-yl)methanol (2ac)

The title compound was prepared according to General Procedure C using salt **1ac** (269.5 mg, 0.5 mmol) at 45 °C for 16 hours. The product was purified by FCC (30-60% EtOAc;pentane) to give *amine* **2ac** (120 mg, 53%) as a yellow solid.

m.p. (acetone): 101-103 °C;

HRMS (ESI): Exact mass calculated for $C_{26}H_{28}O_2N_3{}^{32}S$ $[M+H]^+$: 446.18967, found: 446.18966;

^{1}H NMR (CDCl$_3$) δ 8.43-8.38 (1H, m, C^{Ar}**H**), 7.97 (1H, ddd, *J* = 8.1, 1.2, 0.6 Hz, C^{Ar}**H**), 7.87-7.84 (2H, m, C^{18}**H** + C^{Ar}**H**), 7.46 (1H, ddd, *J* = 8.2, 7.2, 1.3 Hz, C^{Ar}**H**), 7.42-7.30 (4H, m, 4 x C^{Ar}**H**), 6.77 (1H, dd, *J* = 4.5, 2.5 Hz, C^{3}**H**), 4.97 (1H, br s , O**H**), 4.31 (2H, t, *J* = 6.8 Hz, C^{17}**H**$_2$), 4.12 (2H, d, *J* = 3.5 Hz, C^{7}**H**$_2$), 3.57 (1H, dd, *J* = 18.4, 4.6 Hz, C^{2}**H**$_2$), 3.34-3.29 (1H, m, C^{5}**H**), 3.16 (1H, dt, *J* = 11.4, 1.5 Hz, C^{6}**H**$_2$), 2.93 (1H, dt, *J* = 18.3, 2.5 Hz, C^{2}**H**$_2$), 2.62 (1H, dd, *J* = 11.3, 3.6 Hz, C^{6}**H**$_2$), 2.56 (3H, s, C^{27}**H**$_3$), 2.47 (2H, td, *J* = 6.6, 2.6 Hz, C^{15}**H**$_2$), 2.22-2.11 (2H, m, C^{16}**H**$_2$);

^{13}C NMR ($CDCl_3$) δ 193.1 (C^{26}), 167.8 (C_q), 153.5 (C_q), 136.7 (C_q), 135.1 (C^{18}), 134.2 (C_q), 132.9 (C_q), 131.8 (C^3), 126.5 (C_q), 126.2 (C^{Ar}), 125.4 (C^{Ar}), 123.4 (C^{Ar}), 123.1 (C^{Ar}), 122.8 (C^{Ar}), 122.6 (C^{Ar}), 121.4 (C^{Ar}), 117.2 (C_q), 109.7 (C^{Ar}), 66.5 (C^7), 55.1 (C^6), 54.3 (C^{15}), 53.3 (C^2), 44.5 (C^{17}), 38.4 (C^5), 27.7 (C^{27}), 26.8 (C^{16});
IR (neat) (cm^{-1}): 2929, 2860, 2163, 2034, 1648, 1609, 1512, 1454, 1435, 1389, 1298, 1265, 1250, 1225, 1174, 1103, 1081, 1062.

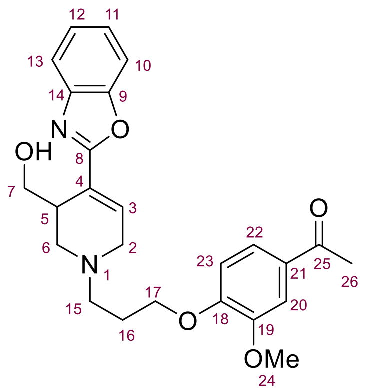

4-(Benzoxazole)-*N*-3-(4-acetyl-2-methoxyphenoxy)propyl)--4-1,2,3,6-tetrahydropyridin-3-yl)methanol (2ad)

The title compound was prepared according to General Procedure C using salt **1ad** (265 mg, 0.50 mmol) at 45 °C for 16 hours. The product was purified by FCC (30-50% EtOAc;pentane) to give *amine* **2ad** (143 mg, 66%) as a yellow gum.
m.p. (acetone): 102-104 °C;
HRMS (ESI): Exact mass calculated for $C_{25}H_{29}O_5N_2$ $[M+H]^+$: 437.20710, found: 437.20634;
^{1}H NMR ($CDCl_3$) δ 7.72-7.69 (1H, m, $C^{10/13}$), 7.57-7.53 (2H, m, C^{23} + C^{20}), 7.51-7.48 (1H, m, $C^{10/13}$), 7.35-7.29 (2H, m, $C^{11/12}$ + $C^{11/12}$), 7.14 (1H, dd, *J* = 4.6, 2.4 Hz, C^3**H**), 6.91 (1H, d, *J* = 8.3 Hz, C^{22}**H**), 5.03 (1H, br s, O**H**), 4.18 (2H, t, *J* = 6.4 Hz, C^{17}**H**$_2$), 4.11 (1H, ddd, *J* = 10.5, 3.0, 1.8 Hz, C^7**H**$_2$), 4.06 (1H, dd, *J* = 10.5, 3.0 Hz, C^7**H**$_2$), 3.93 (3H, s, C^{24}**H**$_3$), 3.69 (1H, dd, *J* = 18.7, 4.6 Hz, C^2**H**$_2$), 3.21 (1H, dt, *J* = 11.2, 1.4 Hz, C^6**H**$_2$), 3.13 (1H, dt, *J* = 4.7, 2.4 Hz, C^5**H**), 3.01 (1H, dt, *J* = 18.7, 2.5 Hz, C^2**H**$_2$), 2.78-2.72 (2H, m, C^{15}**H**$_2$), 2.67 (1H, ddd, *J* = 11.3, 3.6, 1.8 Hz, C^6**H**$_2$), 2.57 (3H, s, C^{26}**H**$_3$), 2.17 (2H, h, *J* = 6.8 Hz, C^{16}**H**$_2$);
^{13}C NMR ($CDCl_3$) δ 196.8 (C^{25}), 162.1 (C_q), 152.6 (C_q), 150.3 (C_q), 149.3 (C_q), 141.7 (C_q), 133.5 (C^3), 130.6 (C_q), 125.4 (C_q), 125.2 ($C^{11/12}$), 124.4 ($C^{11/12}$), 123.3 (C^{23}), 119.9 ($C^{10/13}$), 111.4 (C^{22}), 110.5 (C^{19}), 110.3 ($C^{10/13}$), 66.9 (C^{17}), 66.5 (C^7), 56.0 (C^{24}), 55.3 (C^6), 54.5 (C^{15}), 53.2 (C^2), 37.0 (C^5), 26.6 (C^{16}), 26.2 (C^{26});
IR (neat) (cm^{-1}): 3438, 2970, 2161, 2032, 1650, 1585, 1540, 1510, 1453, 1419, 1359, 1269, 1243, 1221, 1179, 1149, 1078, 1027.

(4-(5-Methylbenzoxazole)-1,2,3,6-tetrahydropyridin-3-yl)methanol (4b)

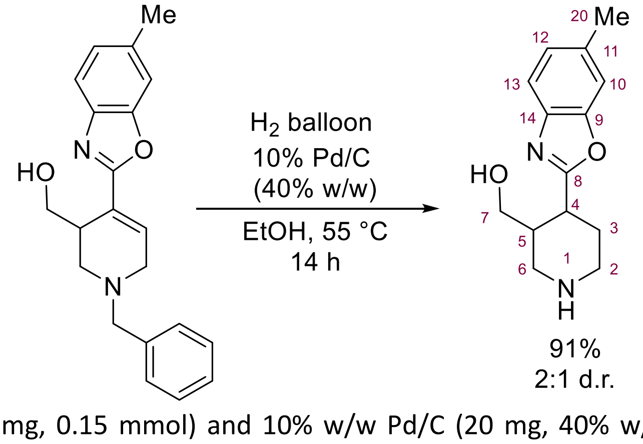

N-Benzylamine **2b** (50 mg, 0.15 mmol) and 10% w/w Pd/C (20 mg, 40% w/w) were suspended in ethanol (7 mL) and the solution was purged with hydrogen. A fresh balloon of hydrogen was added to the suba seal and the reaction was vigorously stirred at 55 °C for 14 hours. The soluion was cooled, filtered through celite and concentrated *in vacuo* to give *amine* **4b** (33 mg, 91%, 2:1 d.r.) as a yellow oil.
HRMS (ESI): Exact mass calculated for $C_{14}H_{19}O_2N_2$ $[M+H]^+$: 247.1441, found: 247.1442;
NMR of diastereomeric mixture ^{1}H NMR ($CDCl_3$) δ 7.50-1.40 (1.5H, m), 7.23-7.20 (1.5H, m), 7.05-6.98 (1.5H, m), 3.67 (1H, d, *J* = 5.2 Hz), 3.48 (1H, dd, *J* = 11.3, 3.9 Hz), 3.39 (1H, dd, *J* = 11.3, 5.7 Hz), 3.30 (1H, dt, *J* = 9.7, 4.6 Hz), 3.22 (1.5H, m), 3.15-3.05 (1.5H, m), 2.98-2.85 (2.5H, m), 2.75 (1H, dt, *J* = 12.4, 9.5, 3.3 Hz), 2.65-2.53 (2H, m), 2.40 (4.5H, s), 2.27-2.18 (1H, m), 2.10-1.90 (2.5 H, m), 1.85-1.75 (1H, m);

^{13}C NMR ($CDCl_3$) δ 167.9 (C_{major}), 167.4 (C_{minor}), 150.7 (C_{major}), 138.5 (C_{minor}), 135.0 (C_{minor}), 133.0 (C_{minor}), 129.0 (C_{major}), 128.5 (C_{major}), 127.6 (C_{minor}), 125.3 (C_{major}), 118.9 (C_{minor}), 118.8 (C_{major}), 110.6 (C_{major}), 110.5 (C_{minor}), 63.7 (C_{major}), 62.9 (C_{minor}), 49.1 (C_{major}), 48.7 (C_{minor}), 45.7 (C_{major}), 45.3 (C_{minor}), 42.6 (C_{minor}), 39.3 (C_{minor}), 39.1 (C_{major}), 38.2 (C_{major}), 31.0 (C_{major}), 29.6 (C_{minor}), 26.8 (C_{minor}), 21.6 (C_{major});
IR (neat) (cm^{-1}): 3392, 2922, 1652, 1609, 1565, 1542, 1487, 1450, 1372, 1328, 1259, 1194, 1165, 1116, 1051, 1016, 992, 940.

Spectra

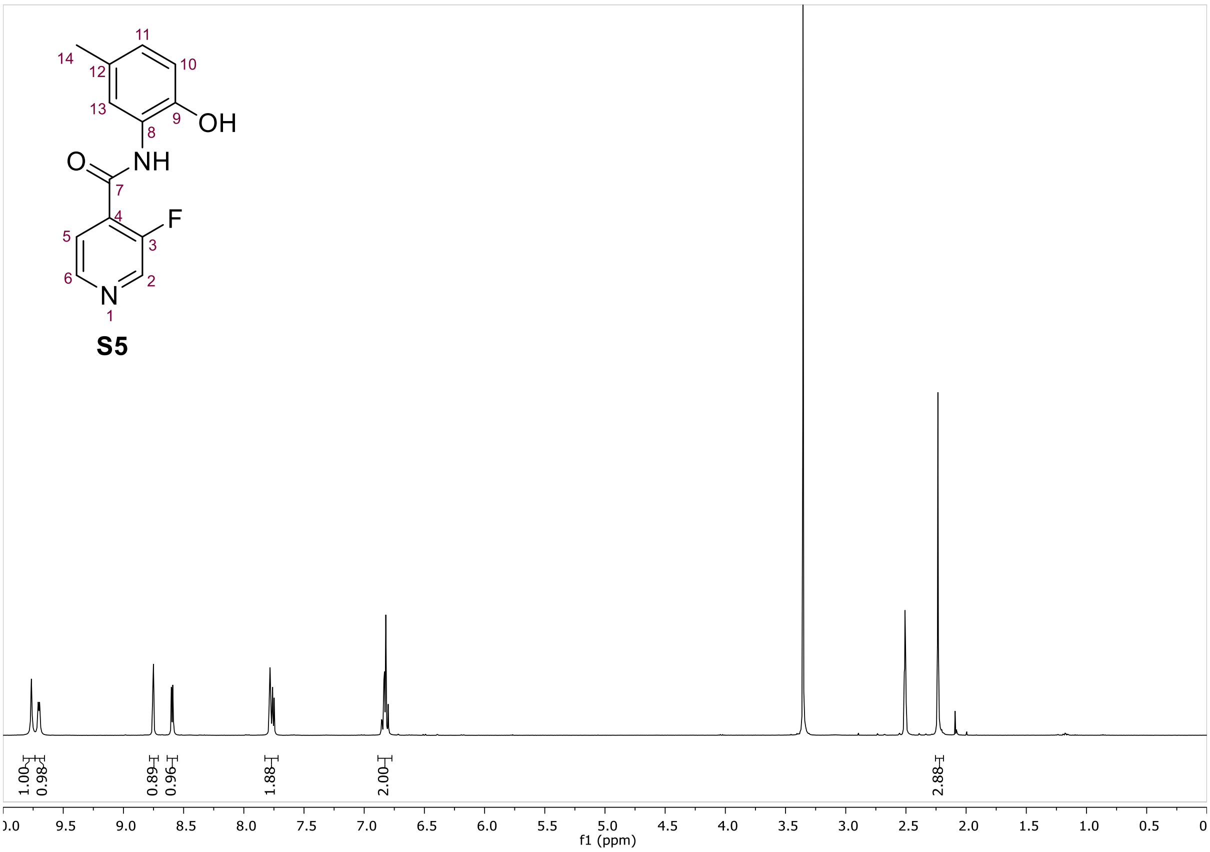

14
11
10
12
13
9
OH
8
O
NH
7
F
4
5
3
6
2
N
1
S5
1.00
0.98
0.89
0.96
1.88
2.00
2.88
f1 (ppm)

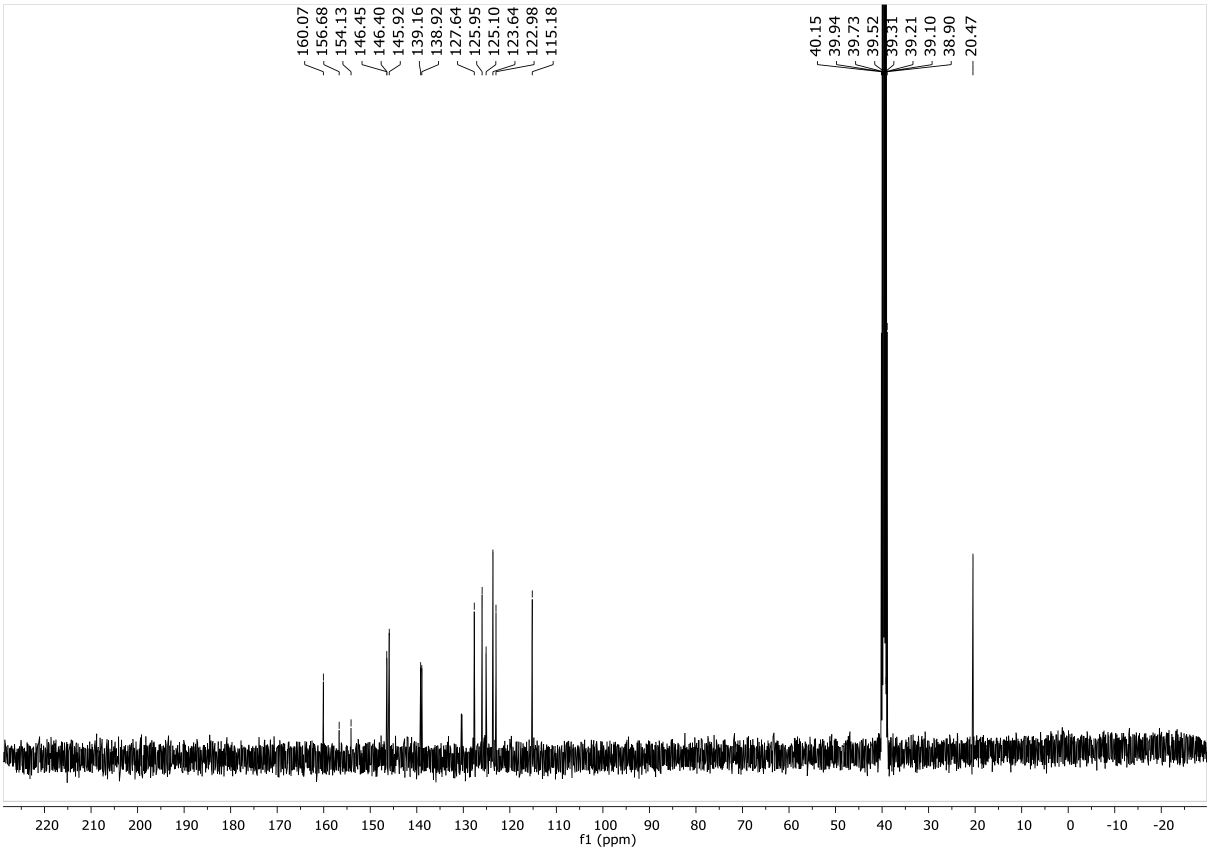

160.07
156.68
154.13
146.45
146.40
145.92
139.16
138.92
127.64
125.95
125.10
123.64
122.98
115.18
40.15
39.94
39.73
39.52
39.31
39.21
39.10
38.90
20.47
f1 (ppm)

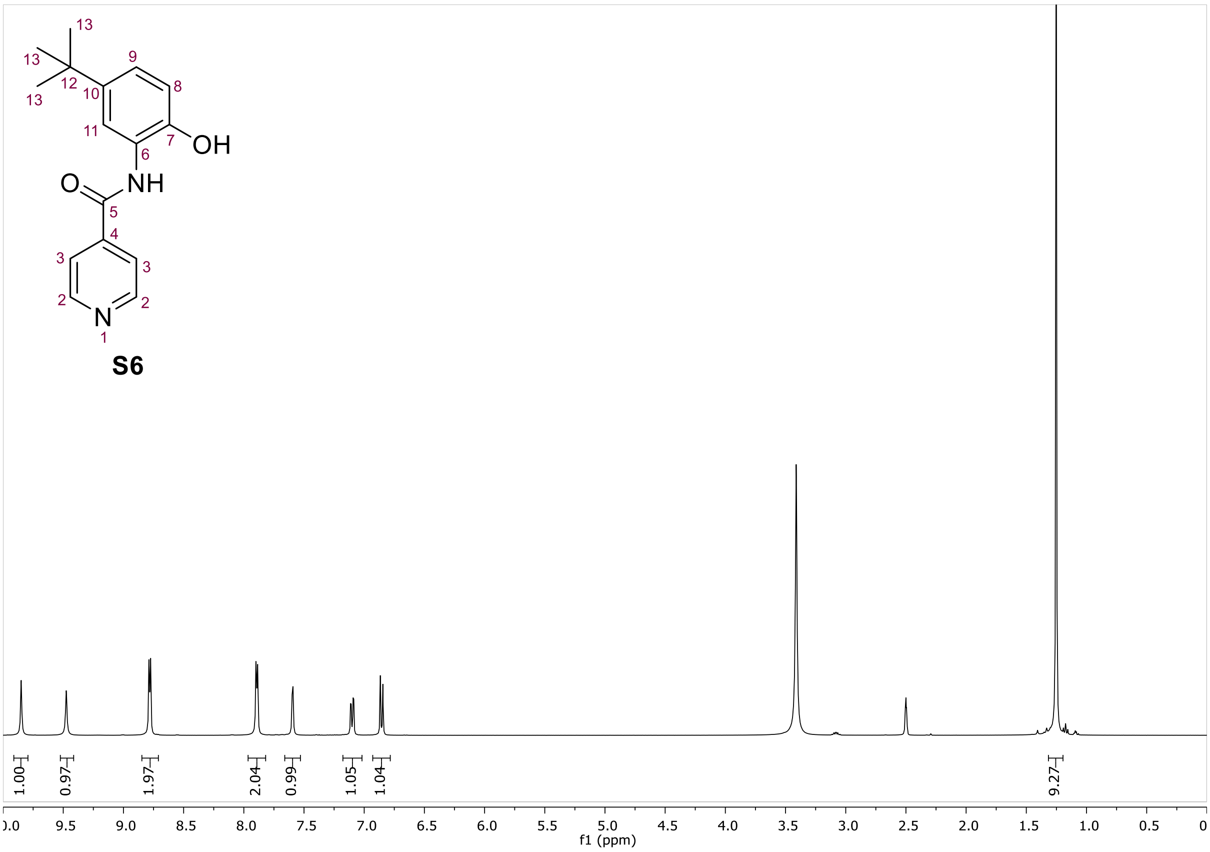
S6
OH
NH
O
N
1.00
0.97
1.97
2.04
0.99
1.05
1.04
9.27
f1 (ppm)

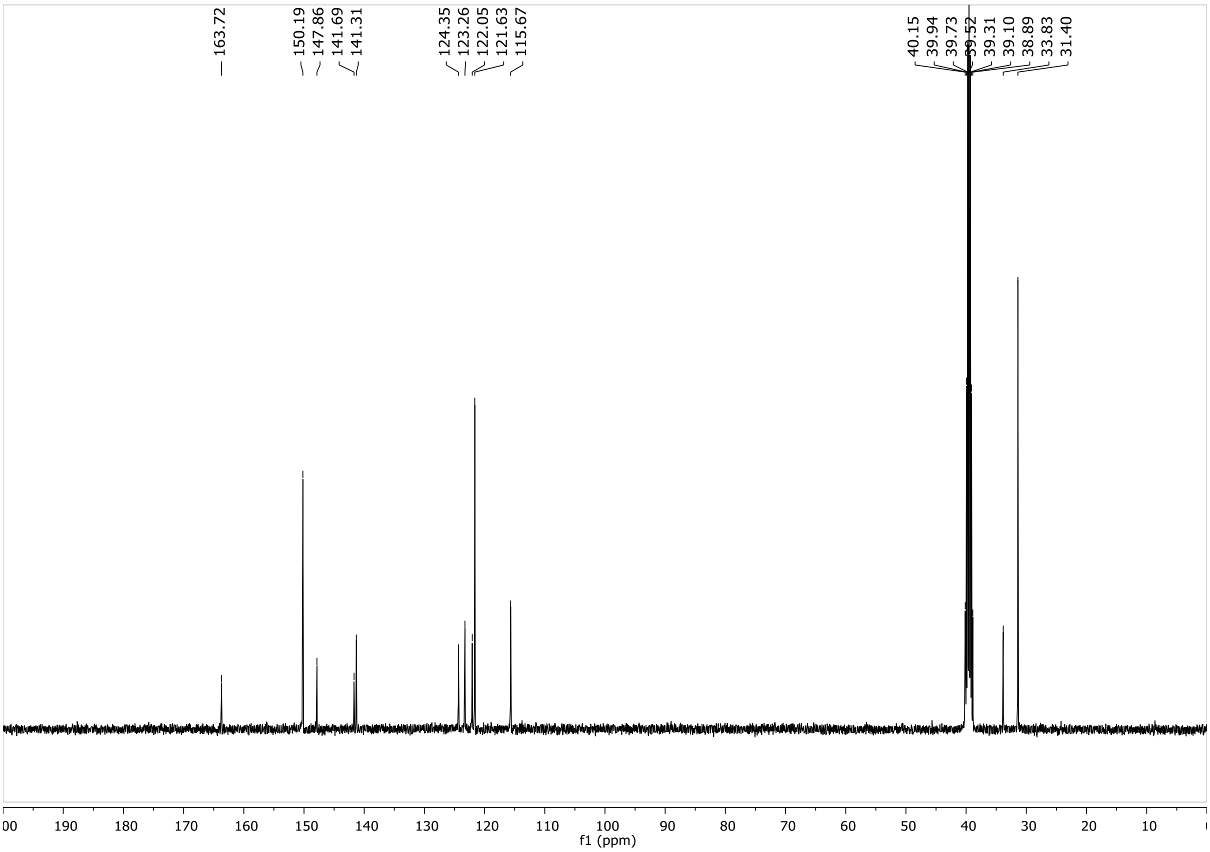
163.72
150.19
147.86
141.69
141.31
124.35
123.26
122.05
121.63
115.67
40.15
39.94
39.73
39.52
39.31
39.10
38.89
33.83
31.40
f1 (ppm)

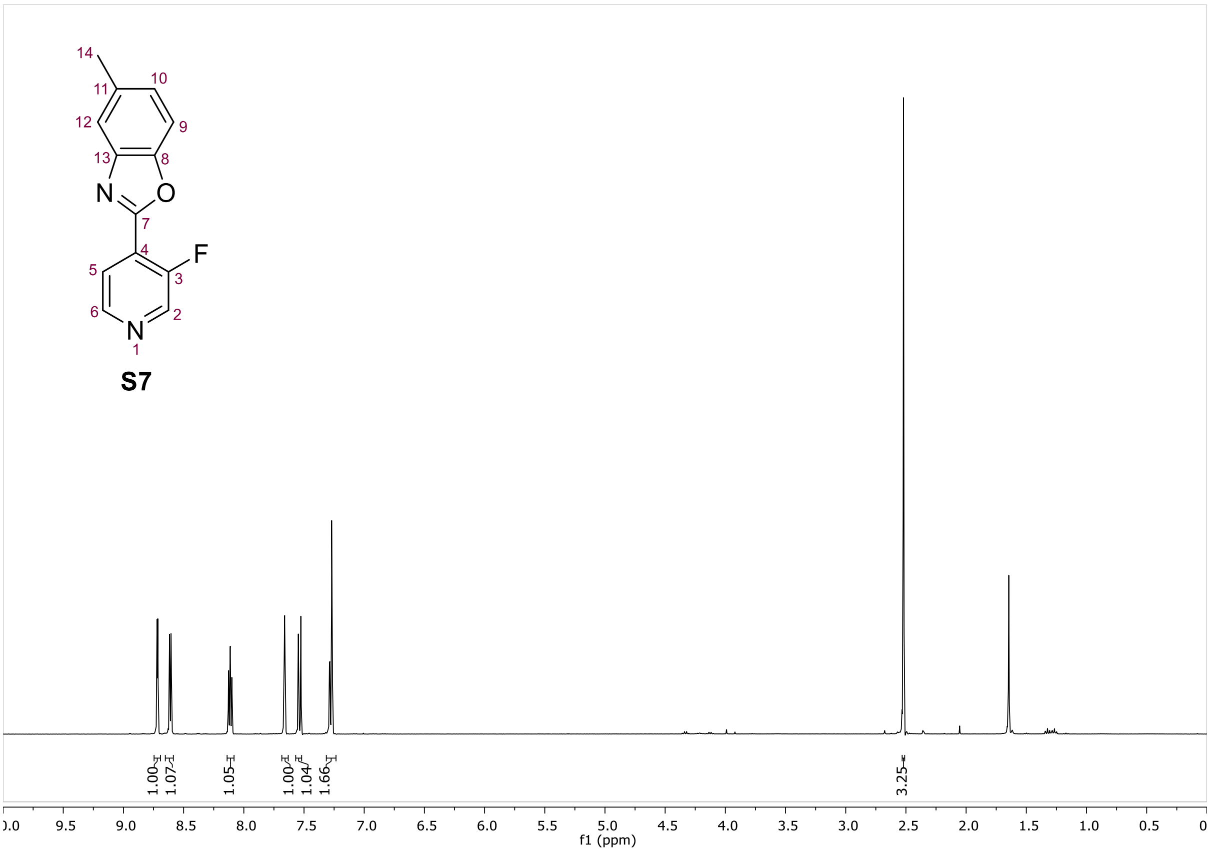
S7
1.00
1.07
1.05
1.00
1.04
1.66
3.25
f1 (ppm)

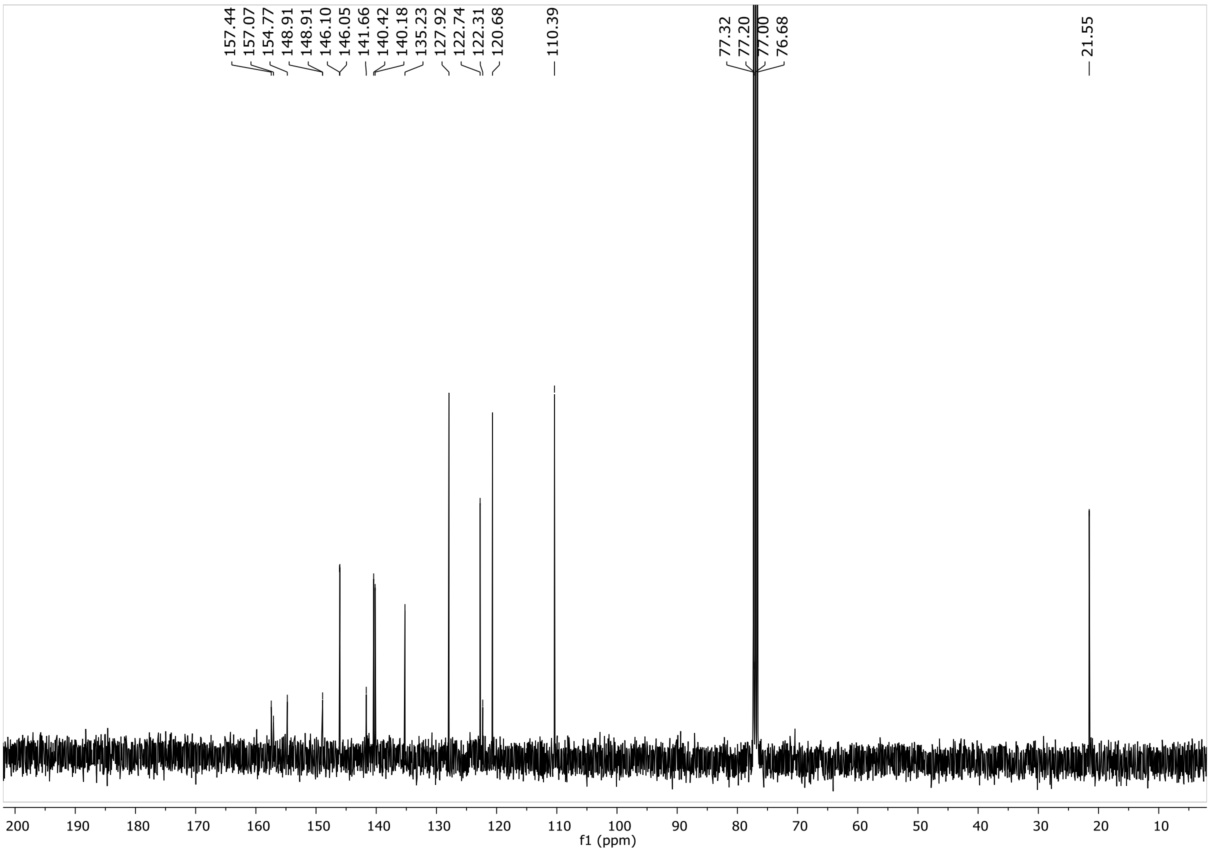
157.44
157.07
154.77
148.91
148.91
146.10
146.05
141.66
140.42
140.18
135.23
127.92
122.74
122.31
120.68
110.39
77.32
77.20
77.00
76.68
21.55
f1 (ppm)

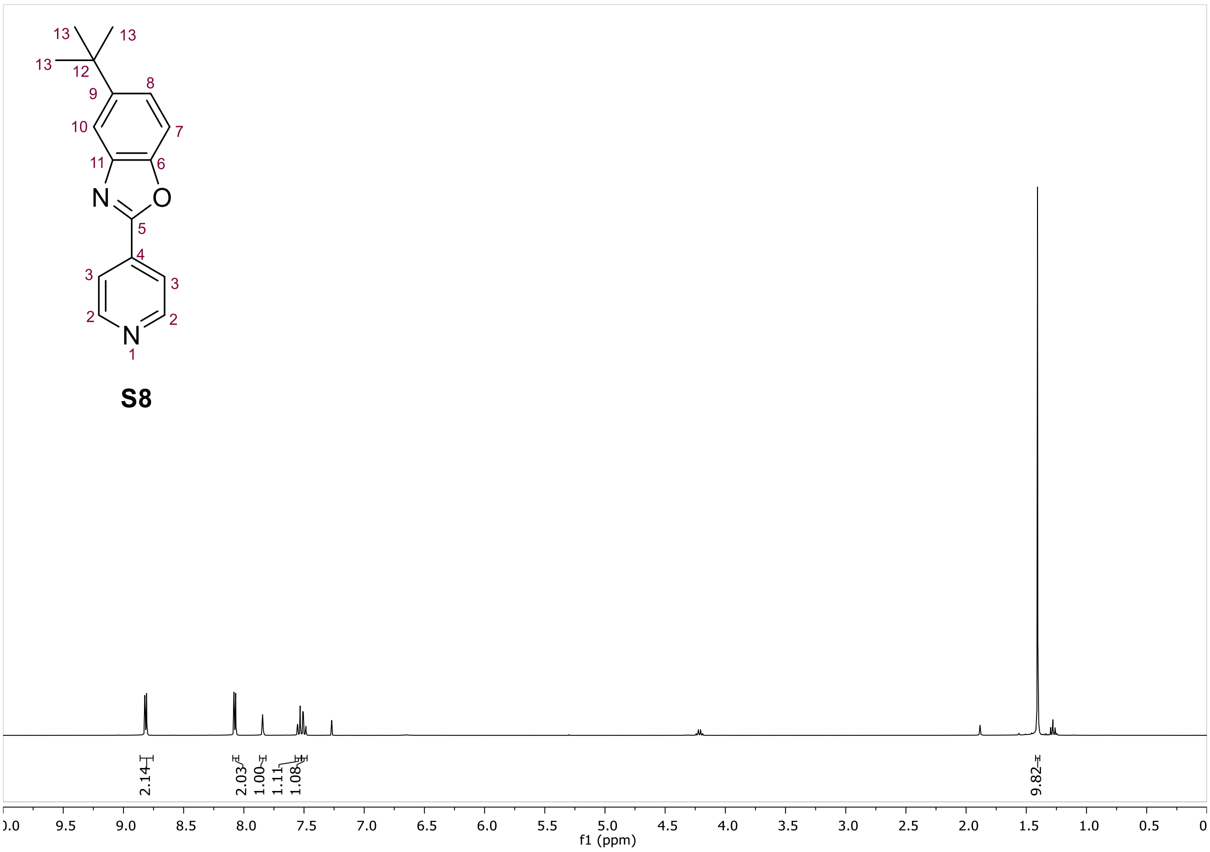
S8
2.14
2.03
1.00
1.11
1.08
9.82
f1 (ppm)

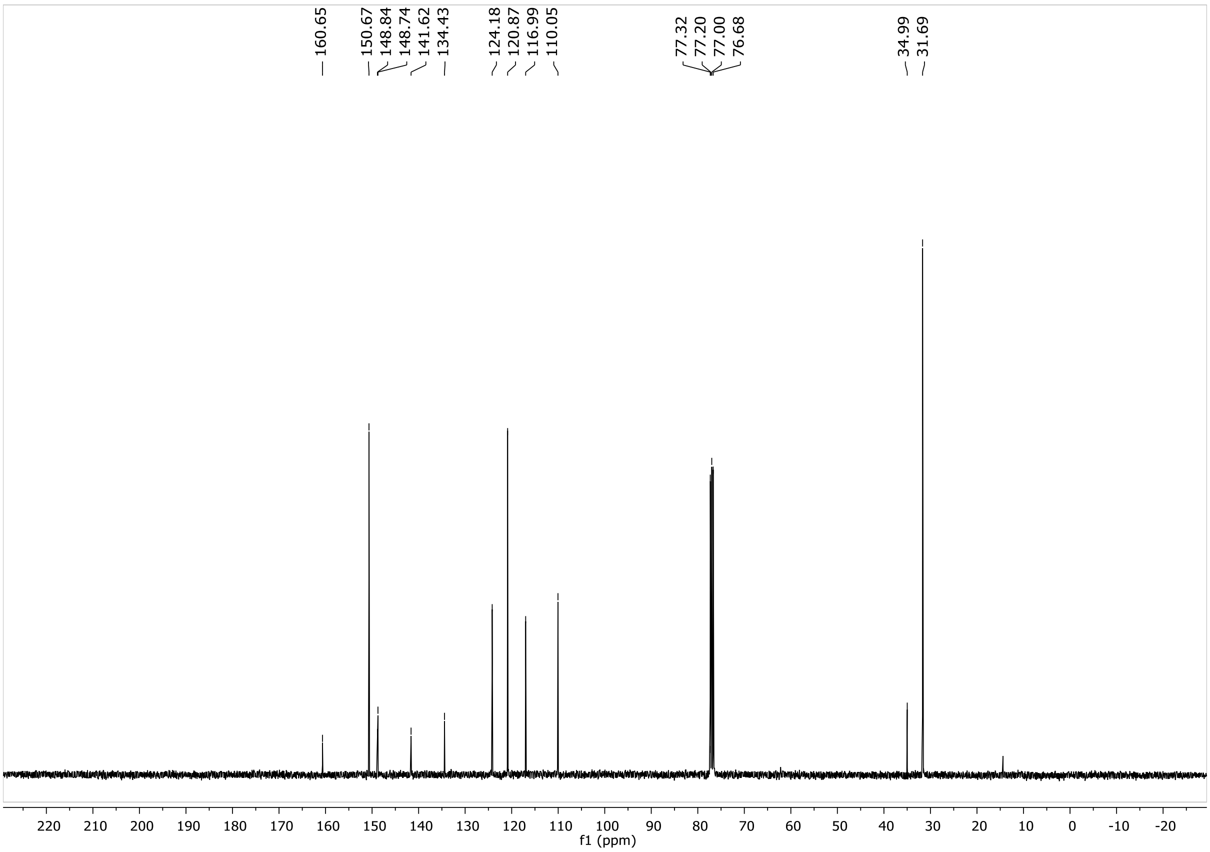
160.65
150.67
148.84
148.74
141.62
134.43
124.18
120.87
116.99
110.05
77.32
77.20
77.00
76.68
34.99
31.69
f1 (ppm)

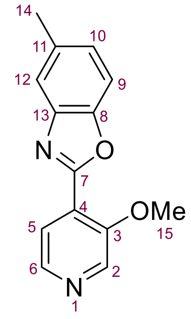
14
10
11
12
9
13
8
N
O
7
4
OMe
5
3
15
6
2
N
1

S9

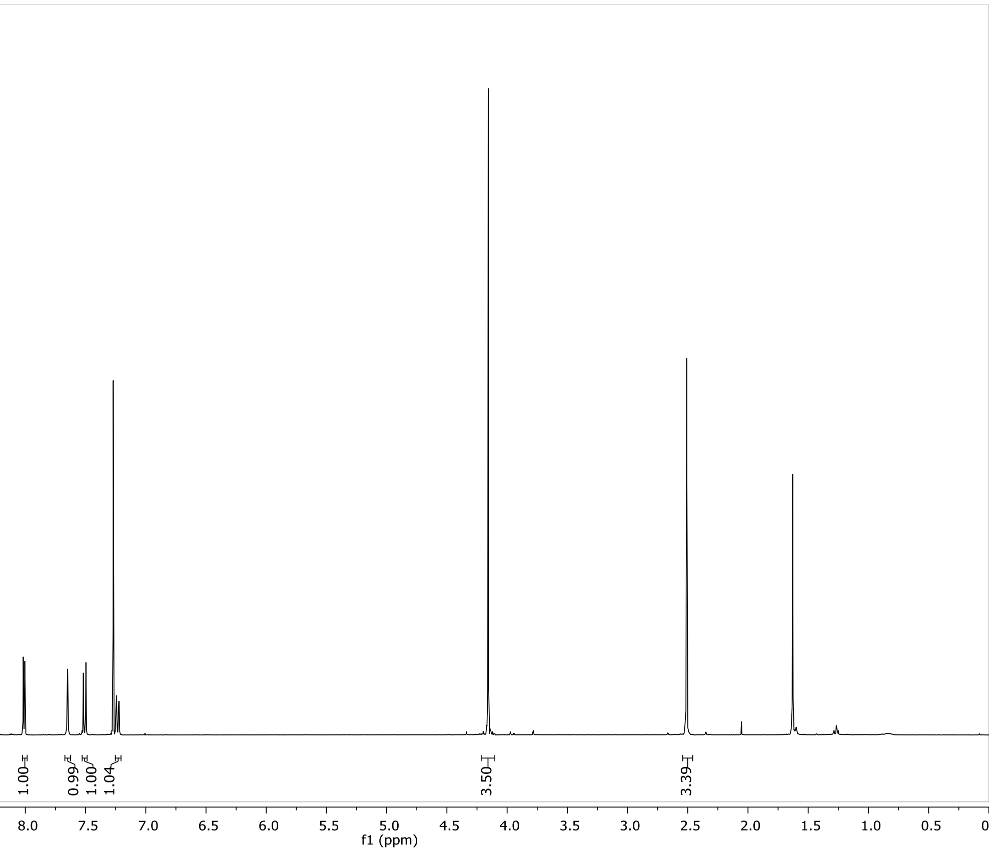
1.00
1.01
1.00
0.99
1.00
1.04
3.50
3.39
9.5 9.0 8.5 8.0 7.5 7.0 6.5 6.0 5.5 5.0 4.5 4.0 3.5 3.0 2.5 2.0 1.5 1.0 0.5 0
f1 (ppm)

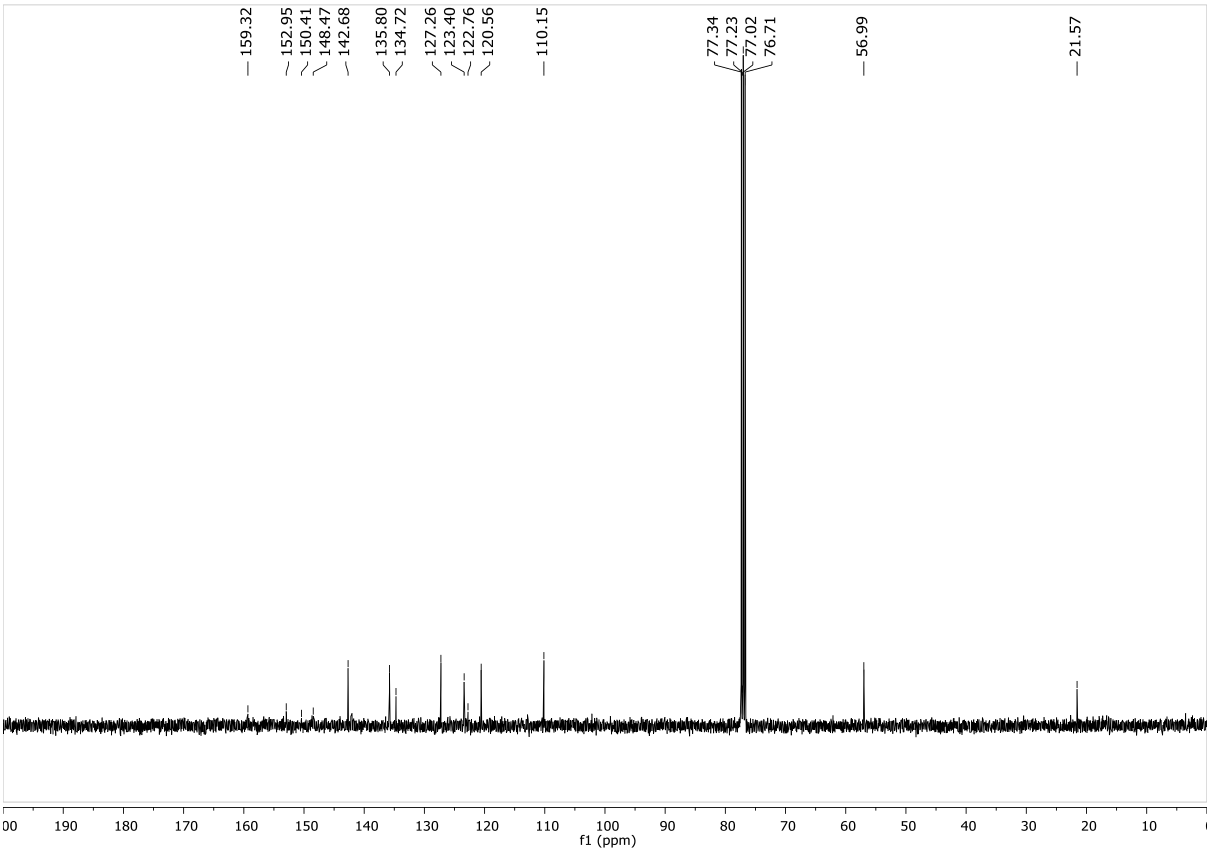
159.32
152.95
150.41
148.47
142.68
135.80
134.72
127.26
123.40
122.76
120.56
110.15
77.34
77.23
77.02
76.71
56.99
21.57
00 190 180 170 160 150 140 130 120 110 100 90 80 70 60 50 40 30 20 10
f1 (ppm)

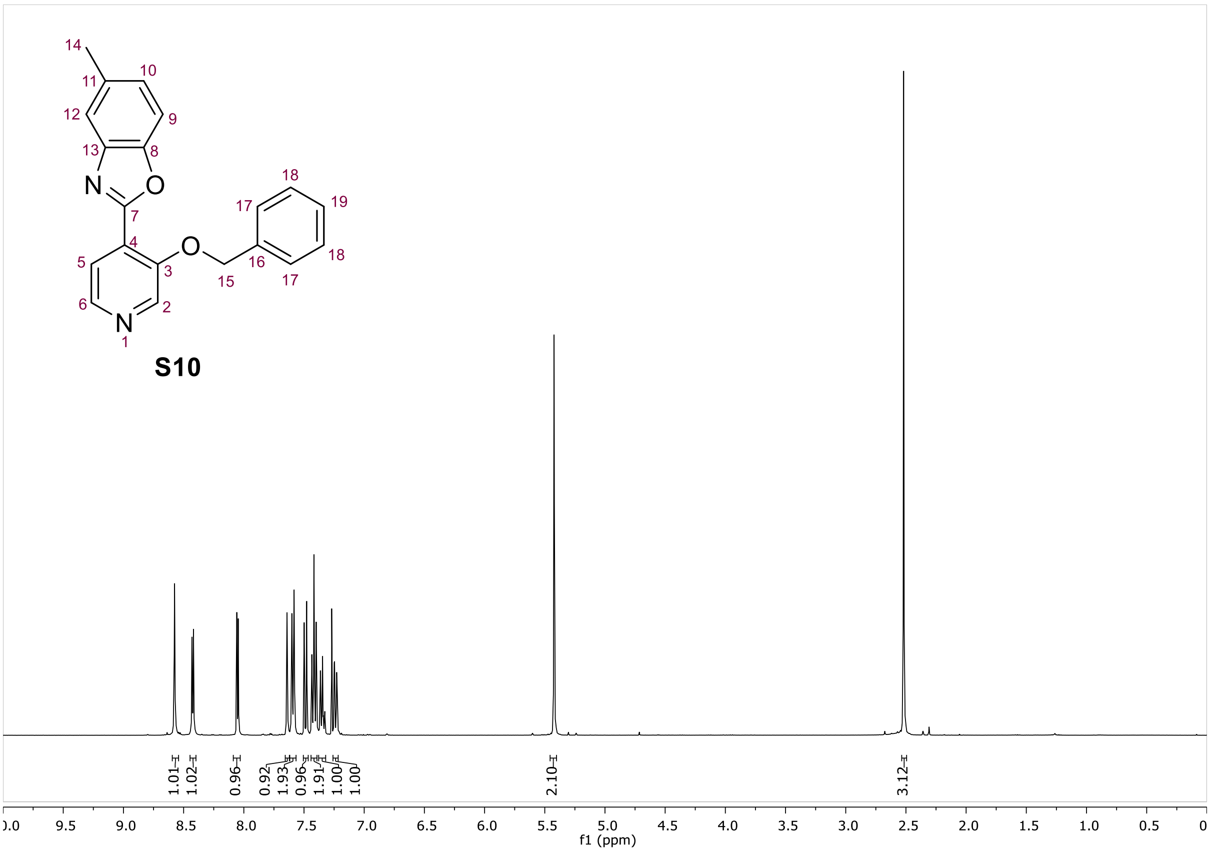
S10
1.01
1.02
0.96
0.92
1.93
0.96
1.91
1.00
1.00
2.10
3.12
f1 (ppm)

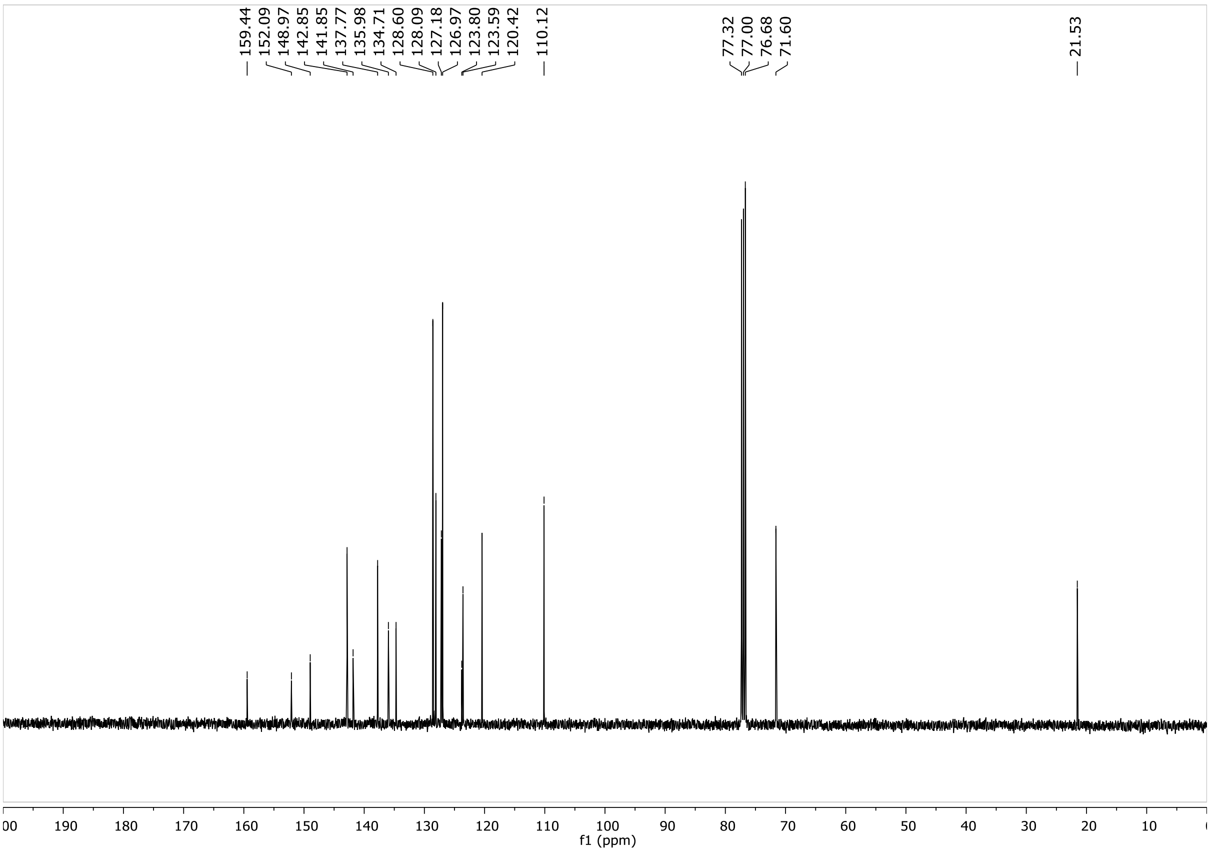
159.44
152.09
148.97
142.85
141.85
137.77
135.98
134.71
128.60
128.09
127.18
126.97
123.80
123.59
120.42
110.12
77.32
77.00
76.68
71.60
21.53
f1 (ppm)

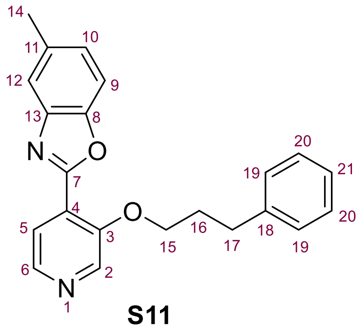
14
10
11
12
9
13
8
N
O
7
20
19
21
4
O
5
3
16
20
15
17
18
19
6
N
2
1

S11

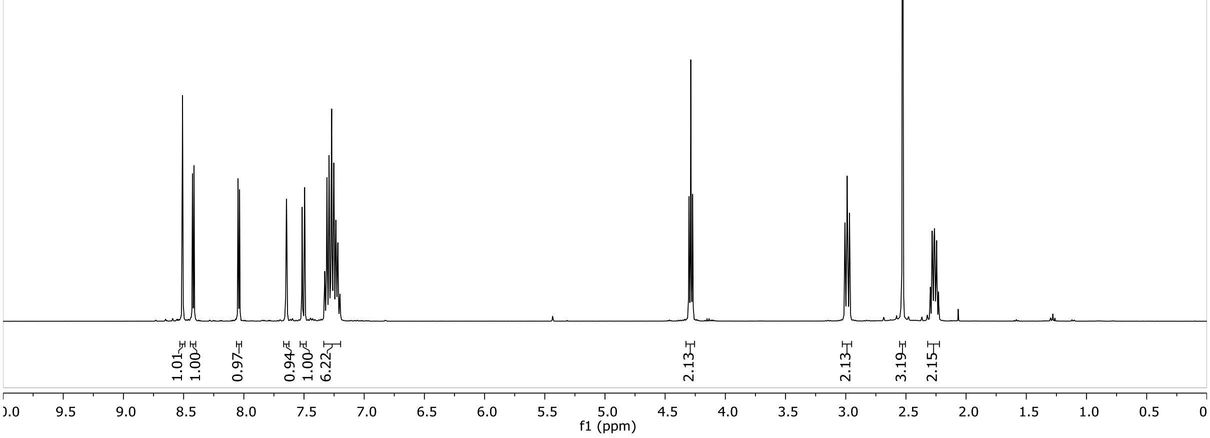
1.01
1.00
0.97
0.94
1.00
6.22
2.13
2.13
3.19
2.15
9.5
9.0
8.5
8.0
7.5
7.0
6.5
6.0
5.5
5.0
4.5
4.0
3.5
3.0
2.5
2.0
1.5
1.0
0.5
f1 (ppm)

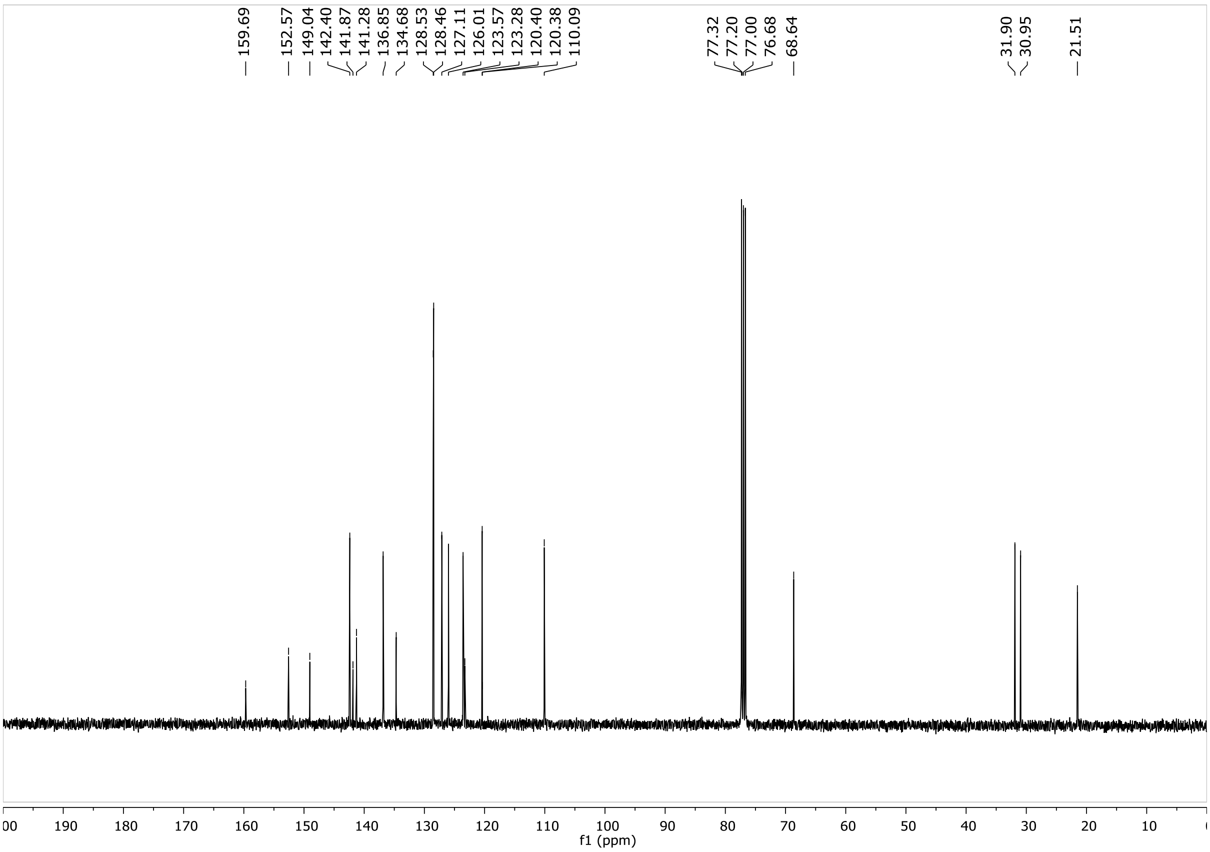
159.69
152.57
149.04
142.40
141.87
141.28
136.85
134.68
128.53
128.46
127.11
126.01
123.57
123.28
120.40
120.38
110.09
77.32
77.20
77.00
76.68
68.64
31.90
30.95
21.51
190
180
170
160
150
140
130
120
110
100
90
80
70
60
50
40
30
20
10
f1 (ppm)

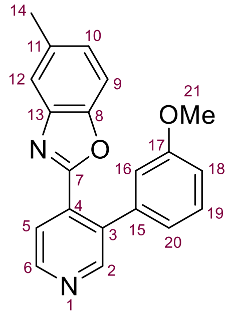
14
11
10
12
9
21
OMe
13
8
N
O
17
16
7
18
4
5
3
15
19
20
6
2
N
1

S12

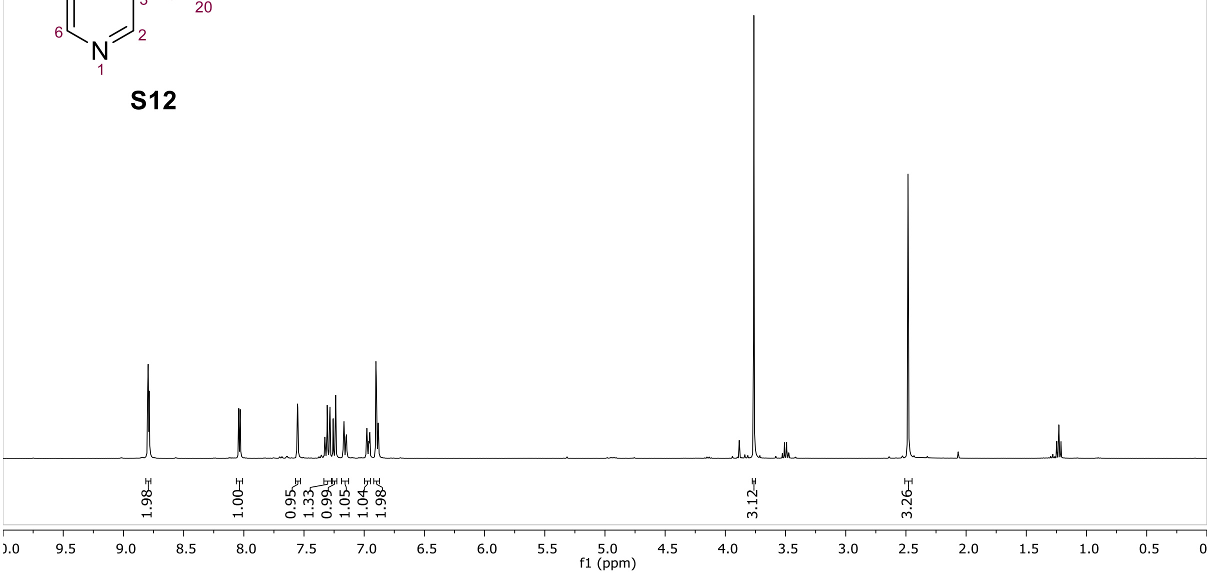
1.98
1.00
0.95
1.33
0.99
1.05
1.04
1.98
3.12
3.26
9.5
9.0
8.5
8.0
7.5
7.0
6.5
6.0
5.5
5.0
4.5
4.0
3.5
3.0
2.5
2.0
1.5
1.0
0.5
0
f1 (ppm)

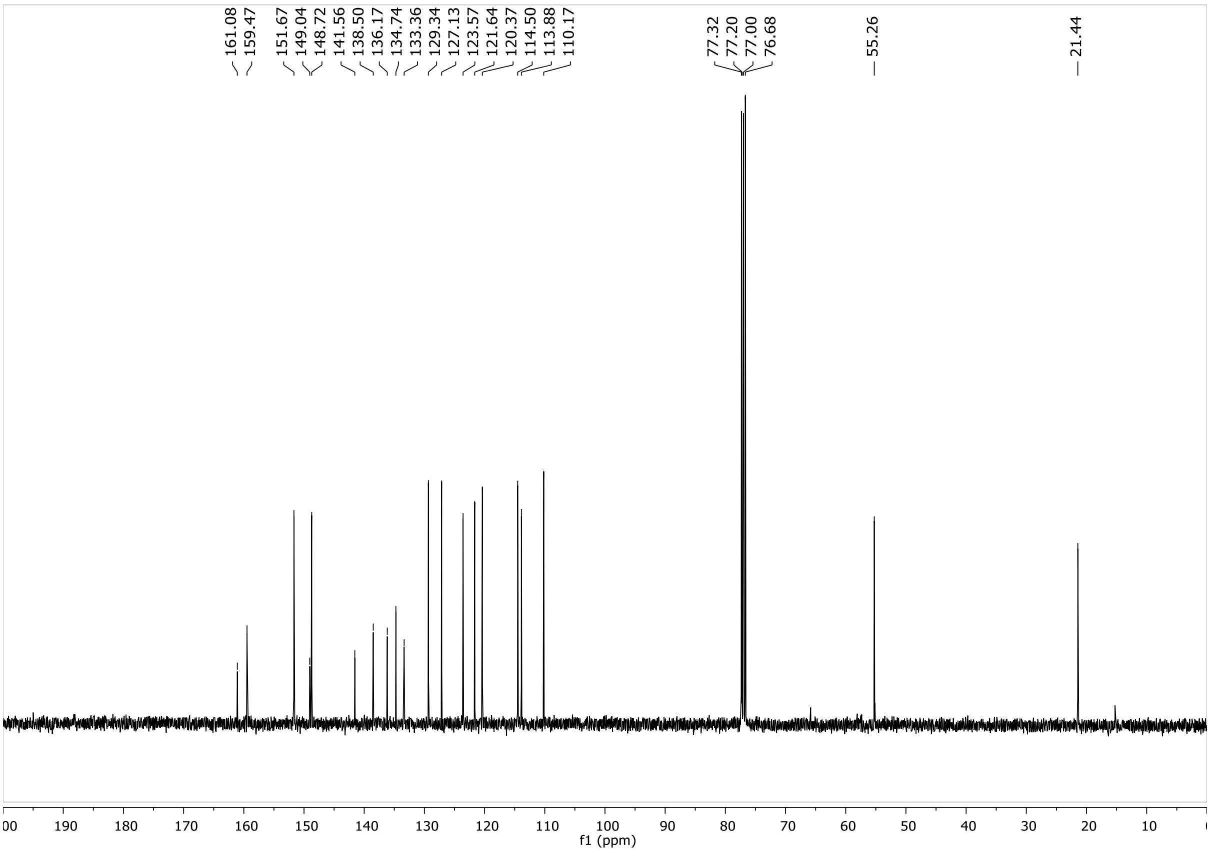
161.08
159.47
151.67
149.04
148.72
141.56
138.50
136.17
134.74
133.36
129.34
127.13
123.57
121.64
120.37
114.50
113.88
110.17
77.32
77.20
77.00
76.68
55.26
21.44
190
180
170
160
150
140
130
120
110
100
90
80
70
60
50
40
30
20
10
f1 (ppm)

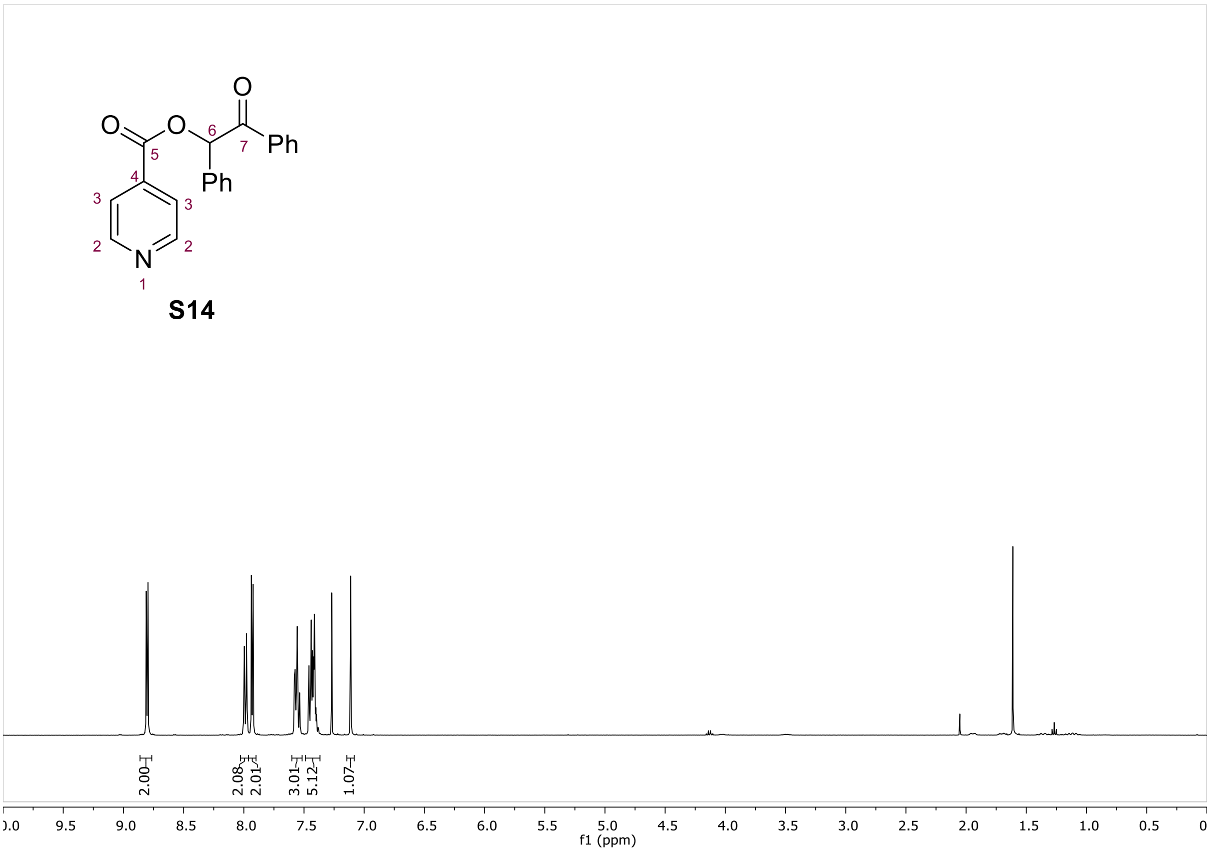

O
O
O
6
7
Ph
5
Ph
4
3
3
2
2
N
1
S14
2.00
2.08
2.01
3.01
5.12
1.07
f1 (ppm)

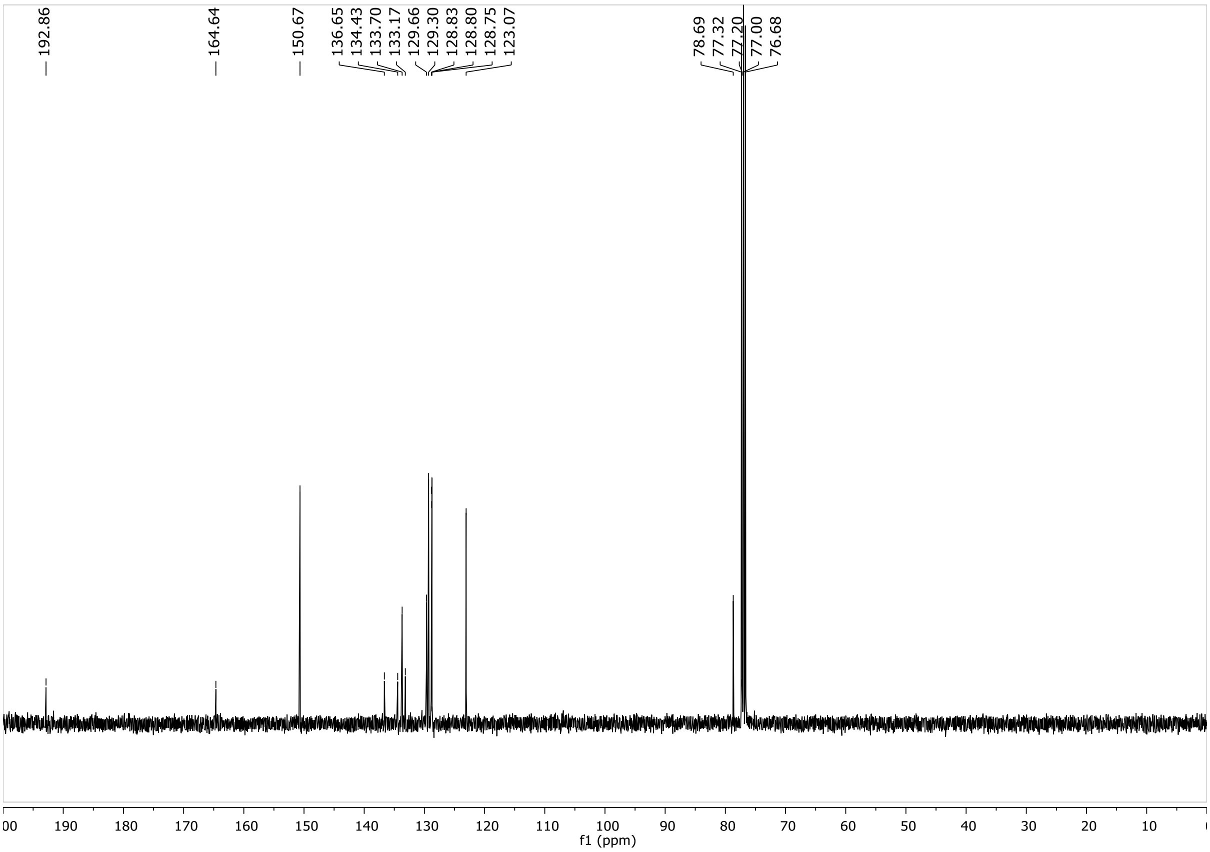

192.86
164.64
150.67
136.65
134.43
133.70
133.17
129.66
129.30
128.83
128.80
128.75
123.07
78.69
77.32
77.20
77.00
76.68
f1 (ppm)

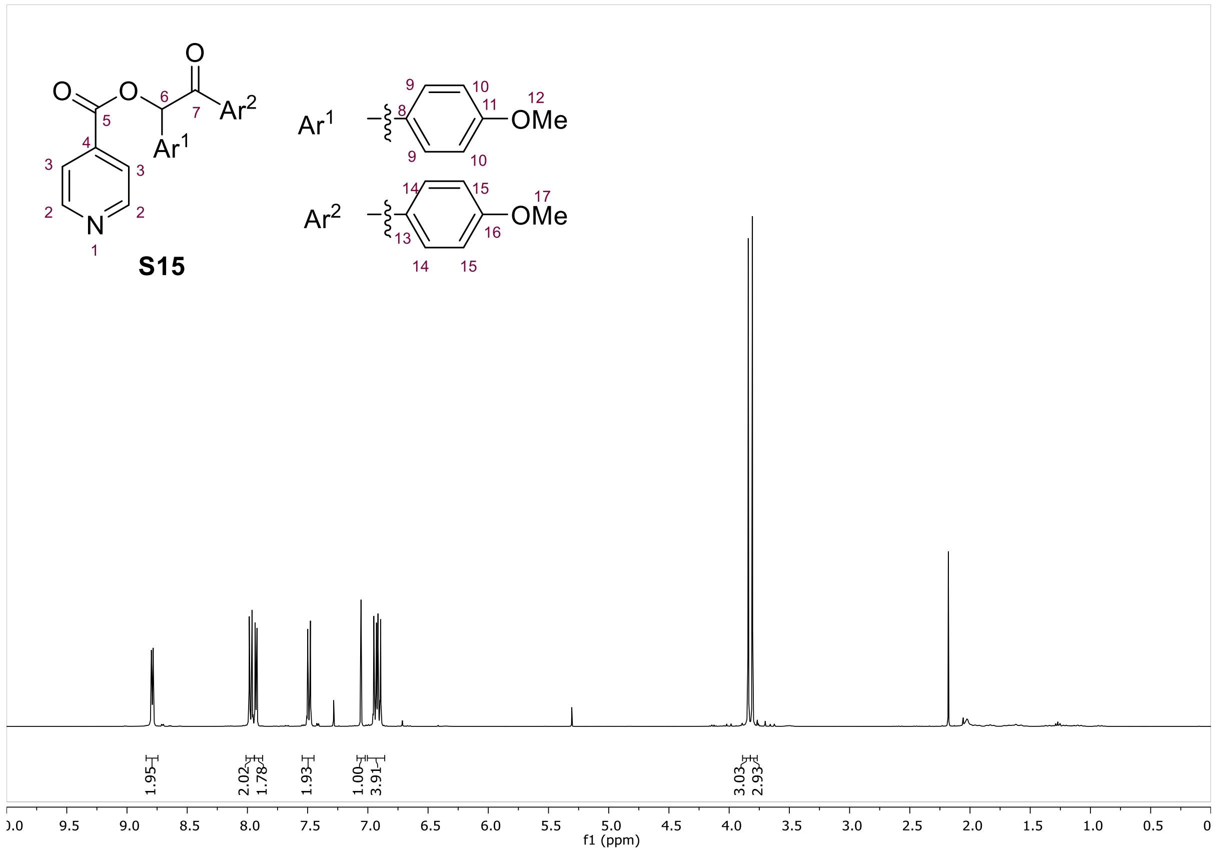
S15
Ar1
Ar2
OMe
1.95
2.02
1.78
1.93
1.00
3.91
3.03
2.93
f1 (ppm)

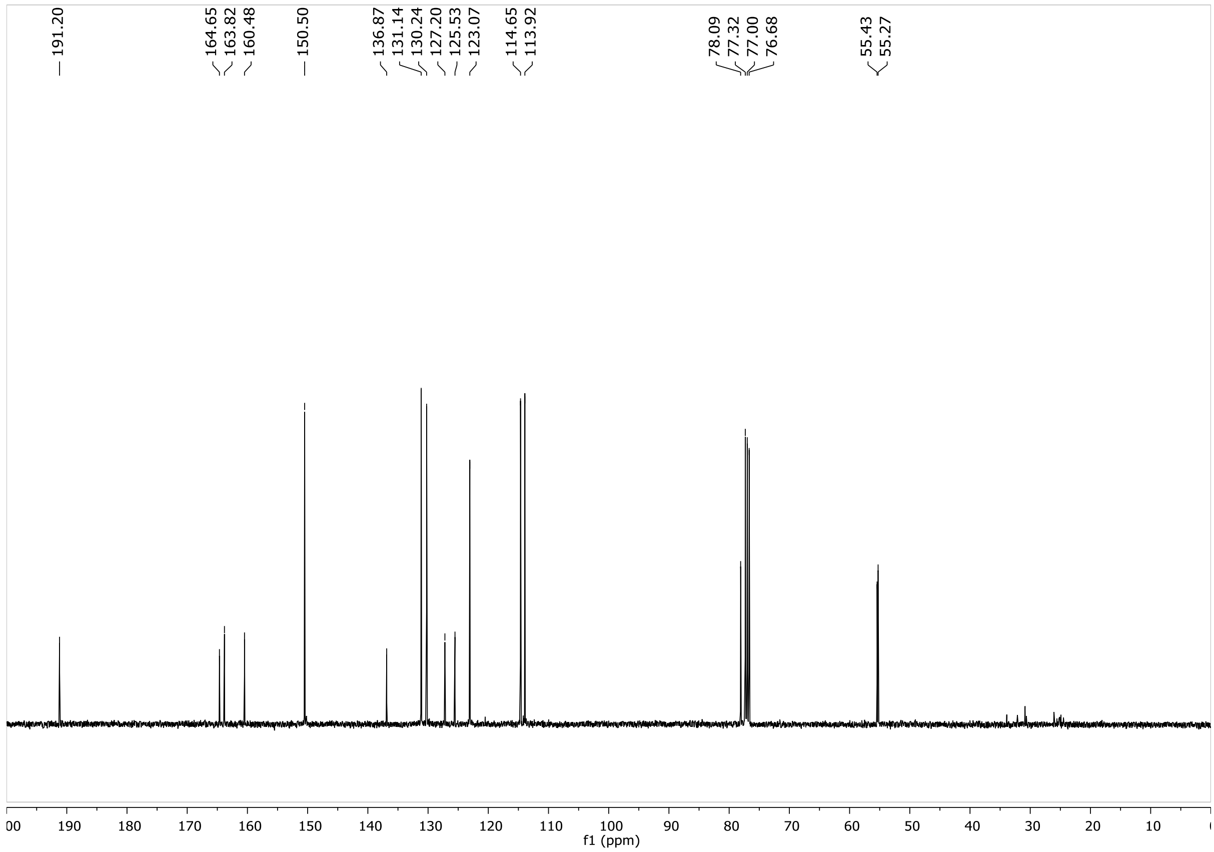
191.20
164.65
163.82
160.48
150.50
136.87
131.14
130.24
127.20
125.53
123.07
114.65
113.92
78.09
77.32
77.00
76.68
55.43
55.27
f1 (ppm)

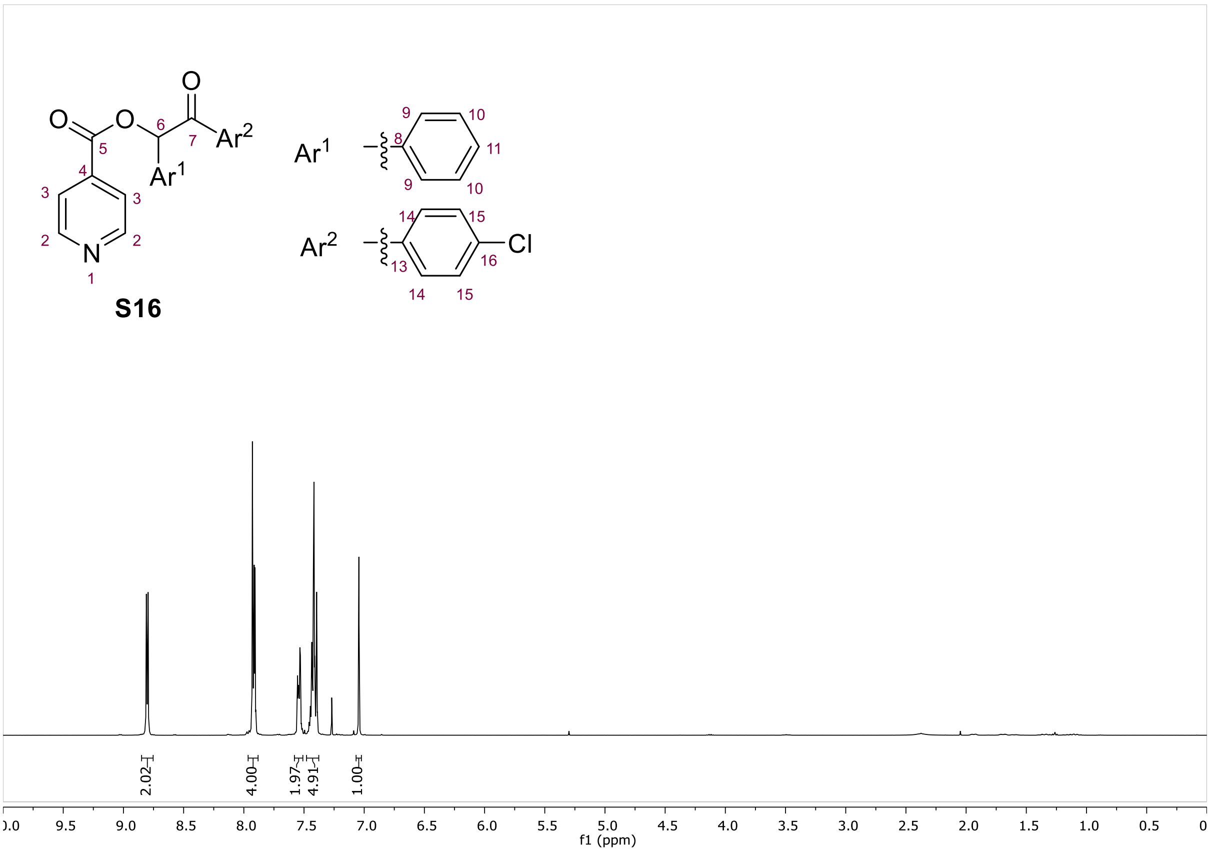

O
O
O
Ar2
Ar1
N
S16
Ar1
Ar2
Cl
2.02
4.00
1.97
4.91
1.00
f1 (ppm)

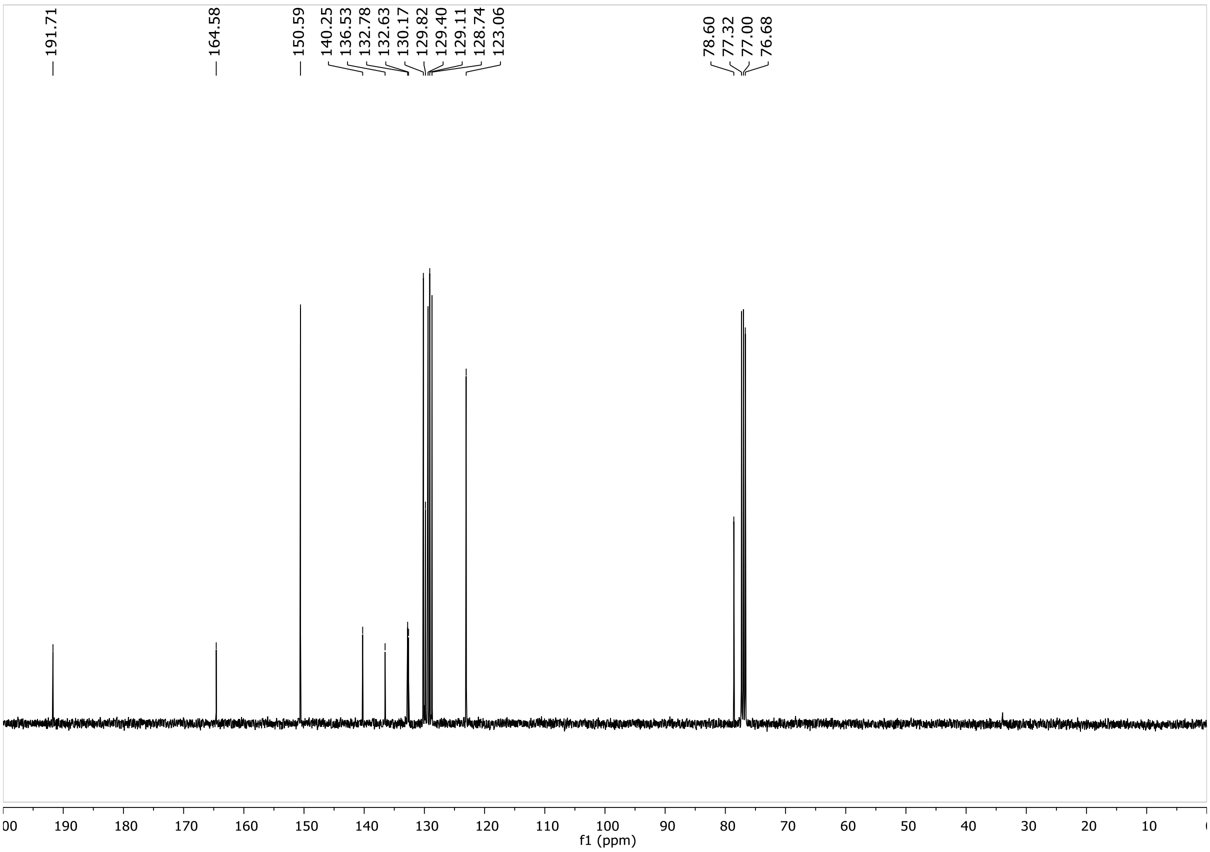

191.71
164.58
150.59
140.25
136.53
132.78
132.63
130.17
129.82
129.40
129.11
128.74
123.06
78.60
77.32
77.00
76.68
f1 (ppm)

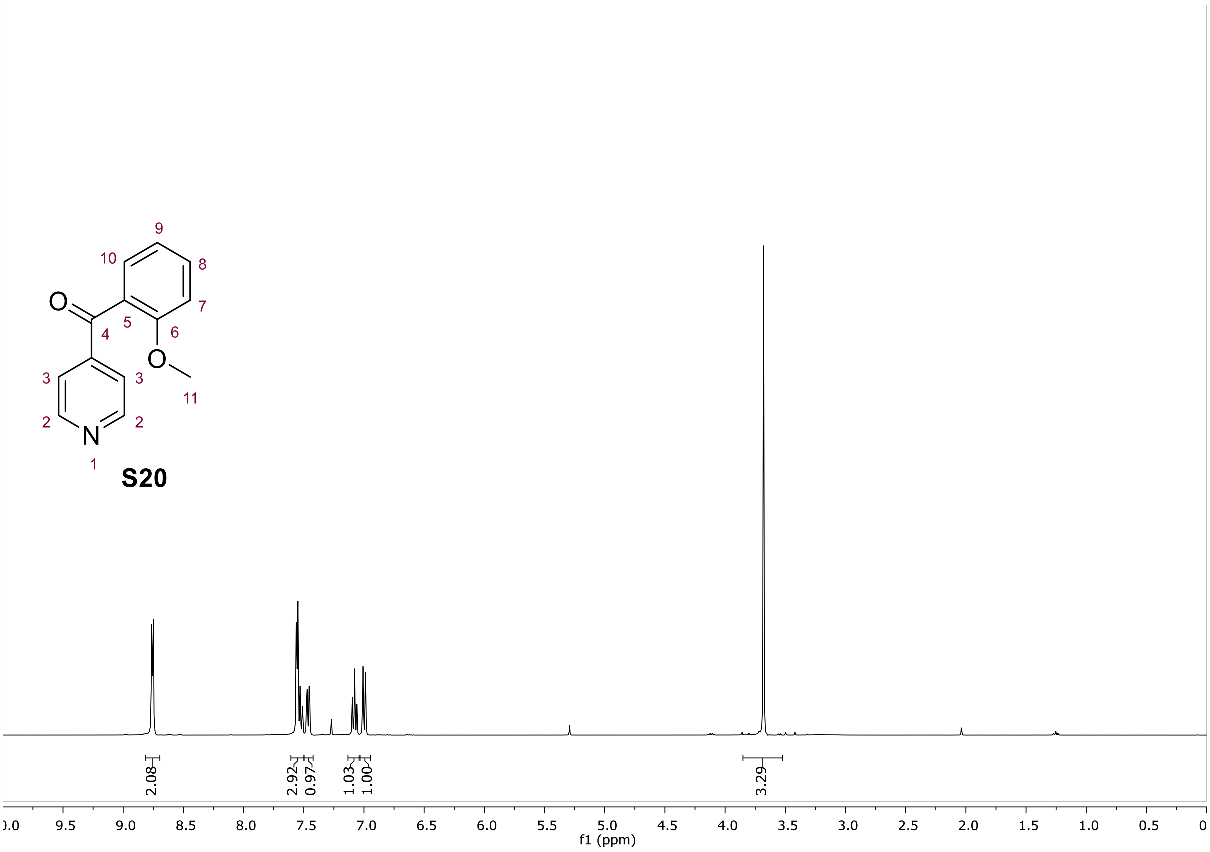
O
N
S20
2.08
2.92
0.97
1.03
1.00
3.29
9.5 9.0 8.5 8.0 7.5 7.0 6.5 6.0 5.5 5.0 4.5 4.0 3.5 3.0 2.5 2.0 1.5 1.0 0.5
f1 (ppm)

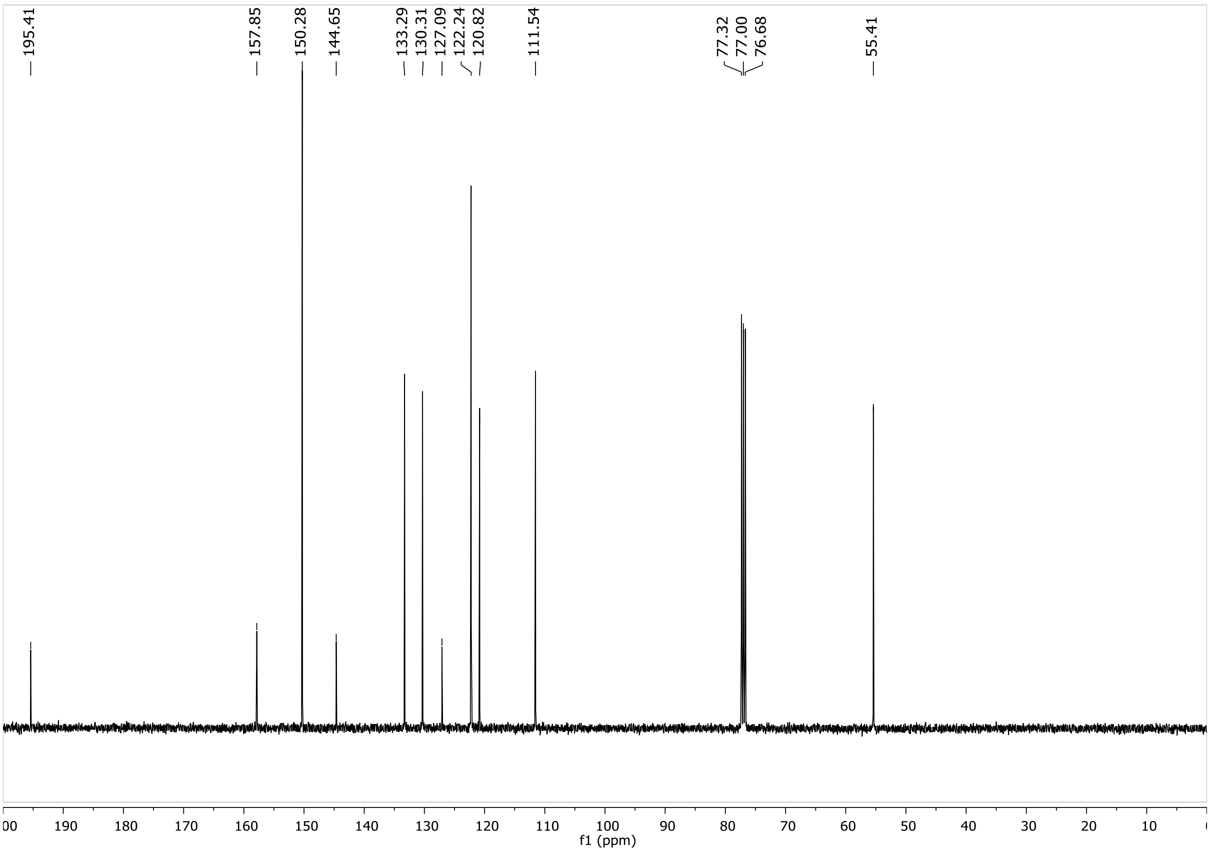
195.41
157.85
150.28
144.65
133.29
130.31
127.09
122.24
120.82
111.54
77.32
77.00
76.68
55.41
190 180 170 160 150 140 130 120 110 100 90 80 70 60 50 40 30 20 10
f1 (ppm)

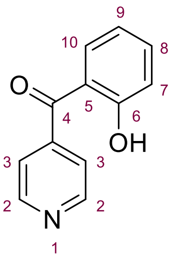
O
OH
N
1
2
2
3
3
4
5
6
7
8
9
10

S21

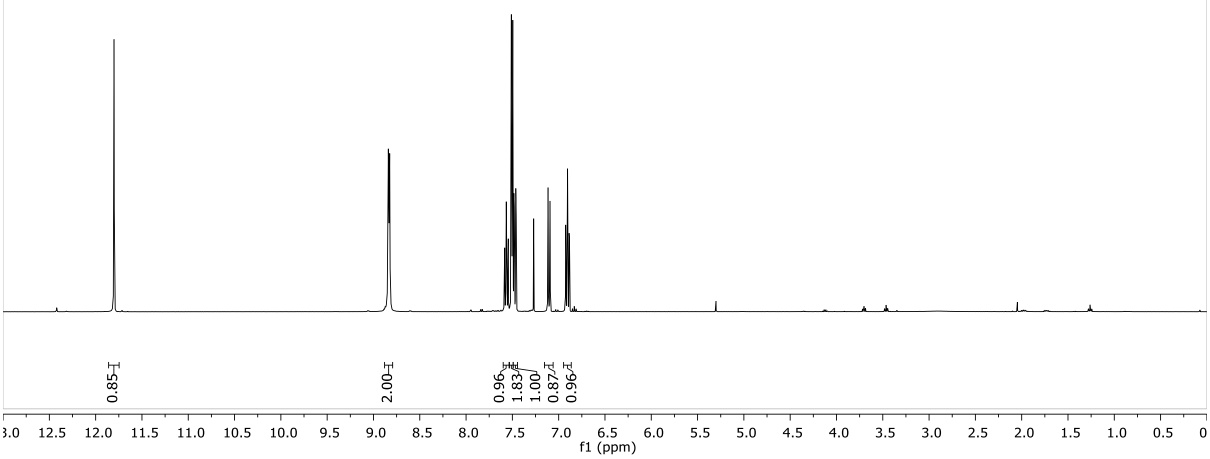
0.85
2.00
0.96
1.83
1.00
0.87
0.96
f1 (ppm)

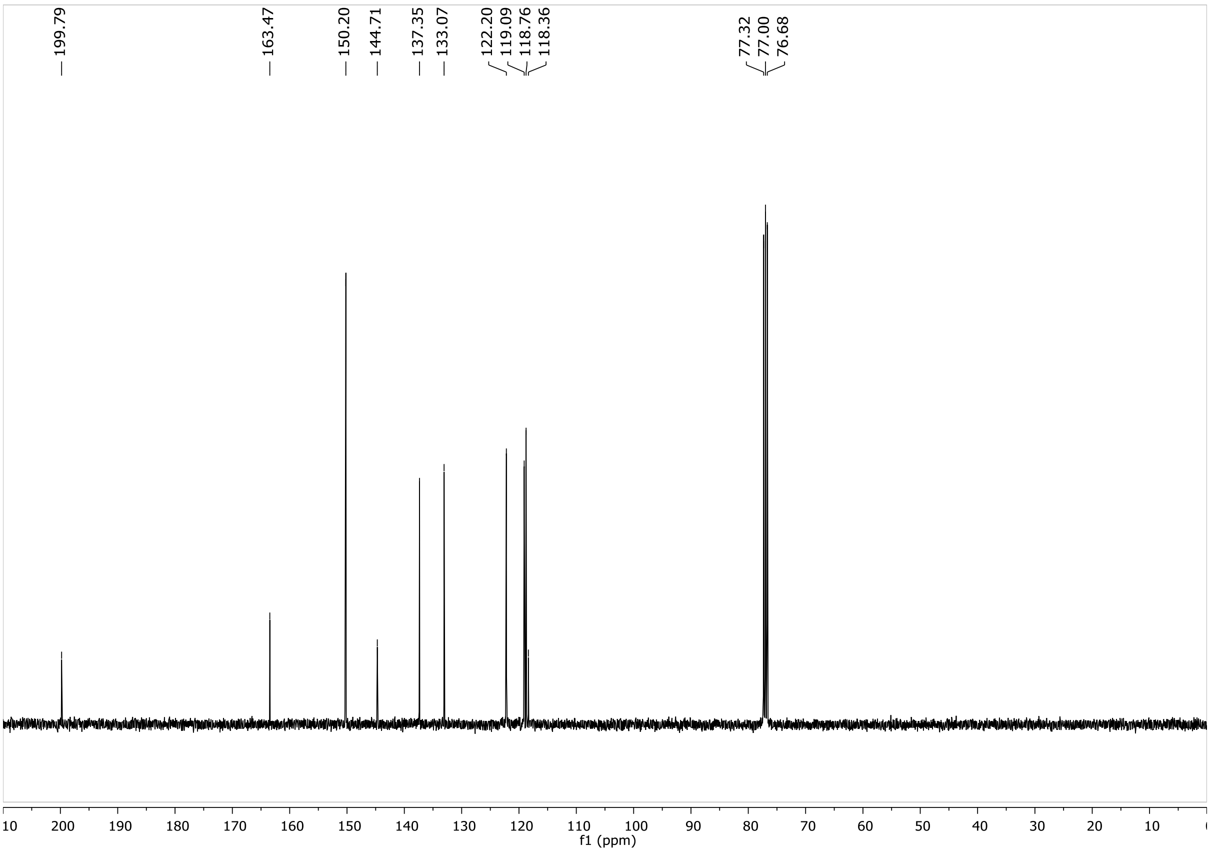
199.79
163.47
150.20
144.71
137.35
133.07
122.20
119.09
118.76
118.36
77.32
77.00
76.68
f1 (ppm)

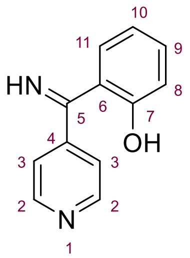

HN
OH
N
1
2
3
4
5
6
7
8
9
10
11

S22

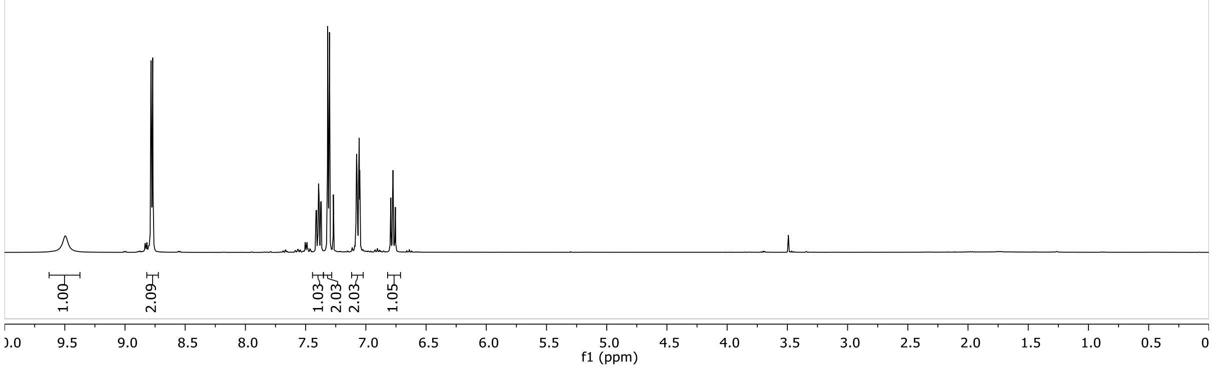

1.00
2.09
1.03
2.03
2.03
1.05
f1 (ppm)

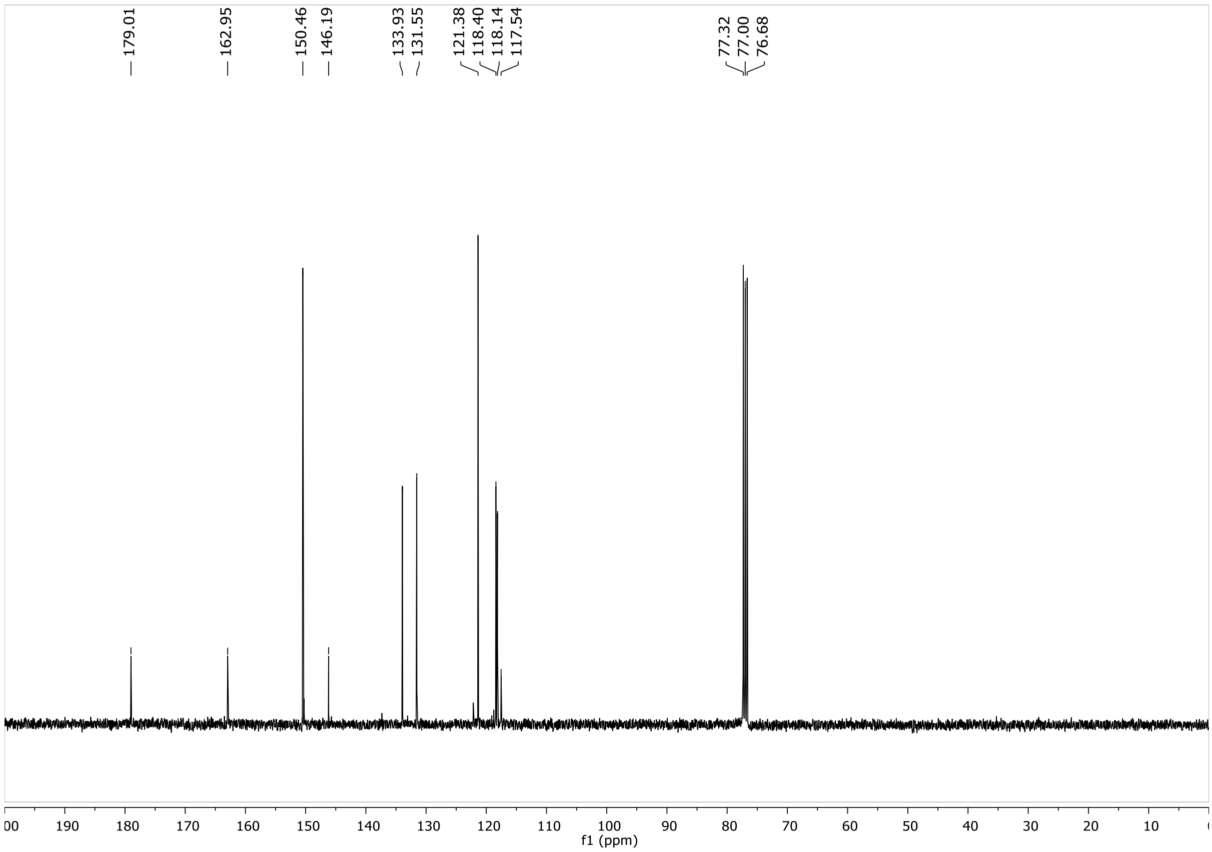

179.01
162.95
150.46
146.19
133.93
131.55
121.38
118.40
118.14
117.54
77.32
77.00
76.68
f1 (ppm)

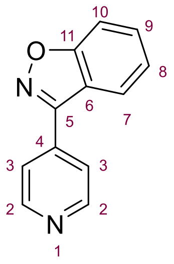

O
N
N
1
2
3
4
5
6
7
8
9
10
11

S23

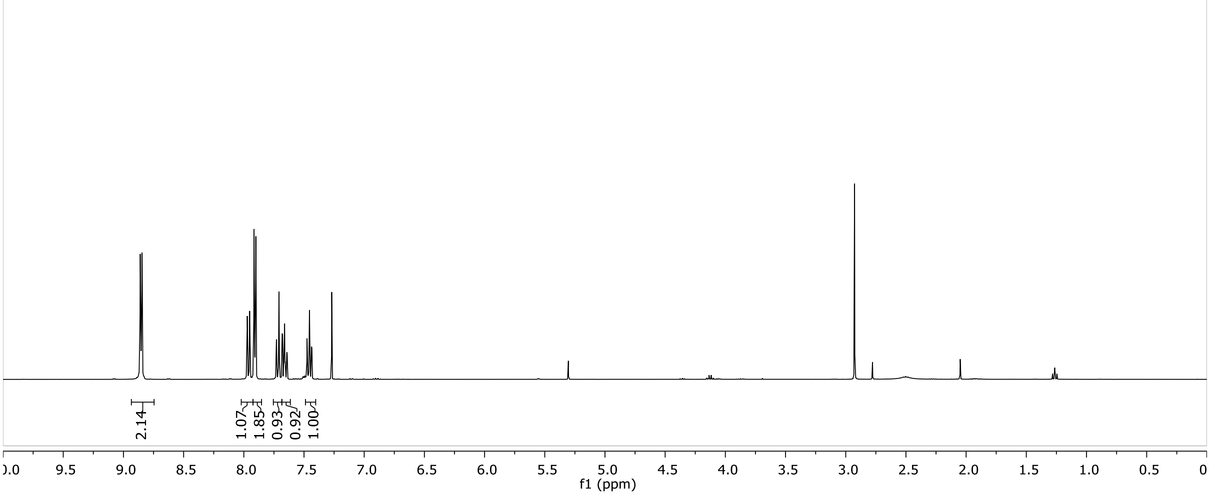

2.14
1.07
1.85
0.93
0.92
1.00
10.0 9.5 9.0 8.5 8.0 7.5 7.0 6.5 6.0 5.5 5.0 4.5 4.0 3.5 3.0 2.5 2.0 1.5 1.0 0.5 0
f1 (ppm)

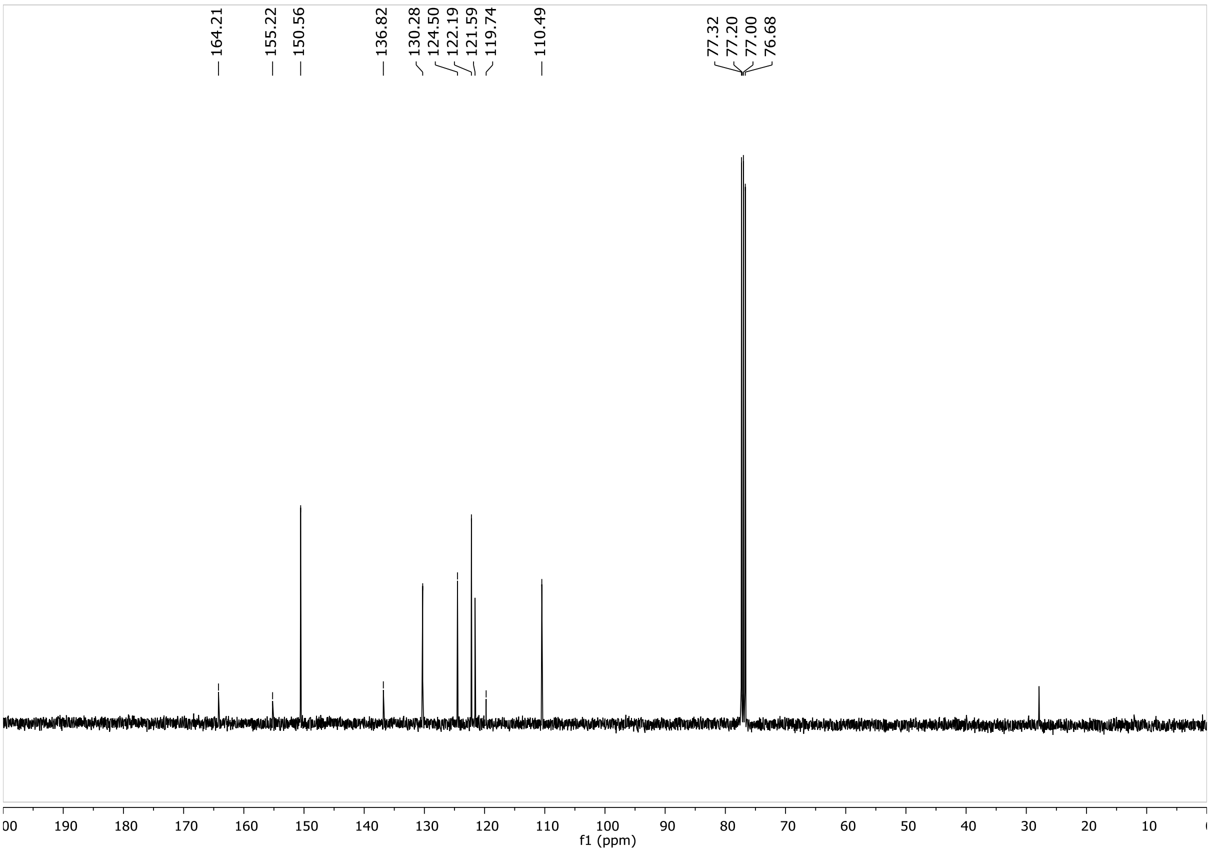

164.21
155.22
150.56
136.82
130.28
124.50
122.19
121.59
119.74
110.49
77.32
77.20
77.00
76.68
200 190 180 170 160 150 140 130 120 110 100 90 80 70 60 50 40 30 20 10 0
f1 (ppm)

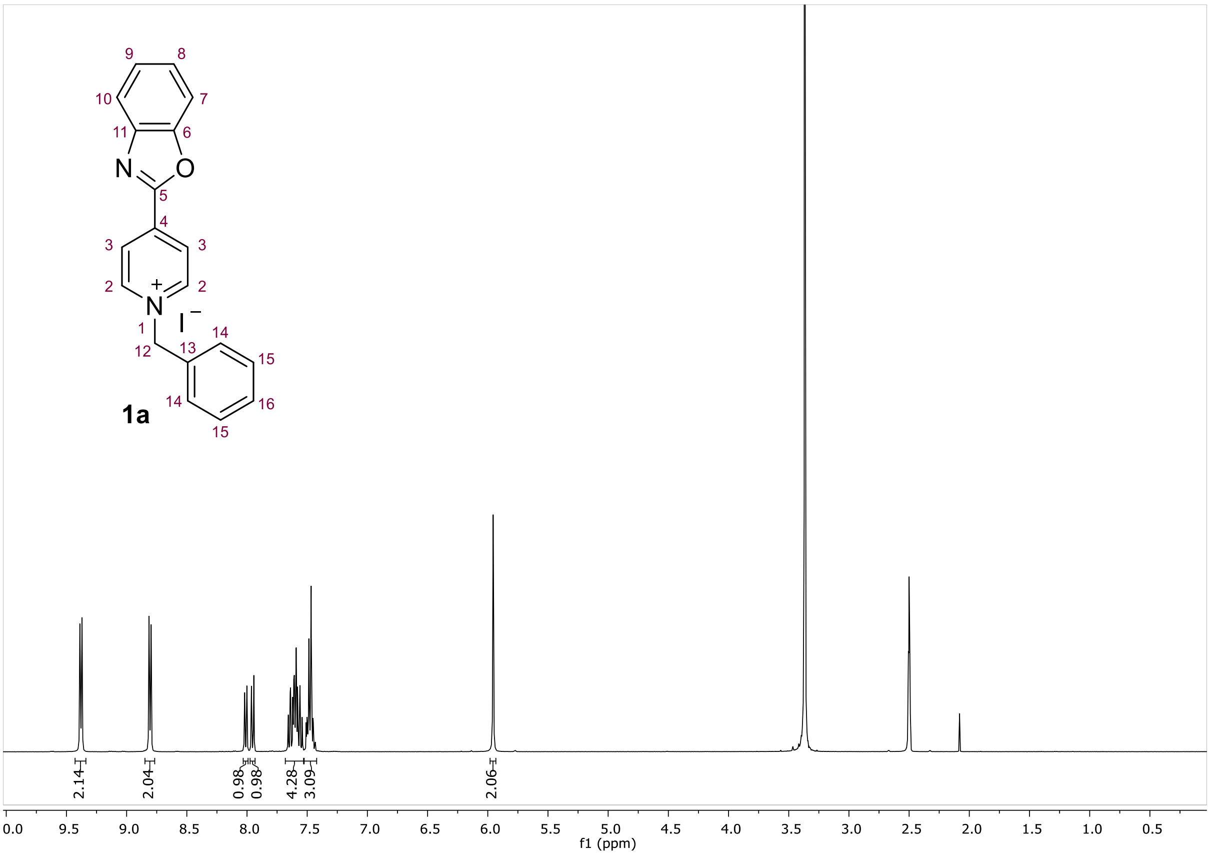
1a
2.14
2.04
0.98
0.98
4.28
3.09
2.06
f1 (ppm)

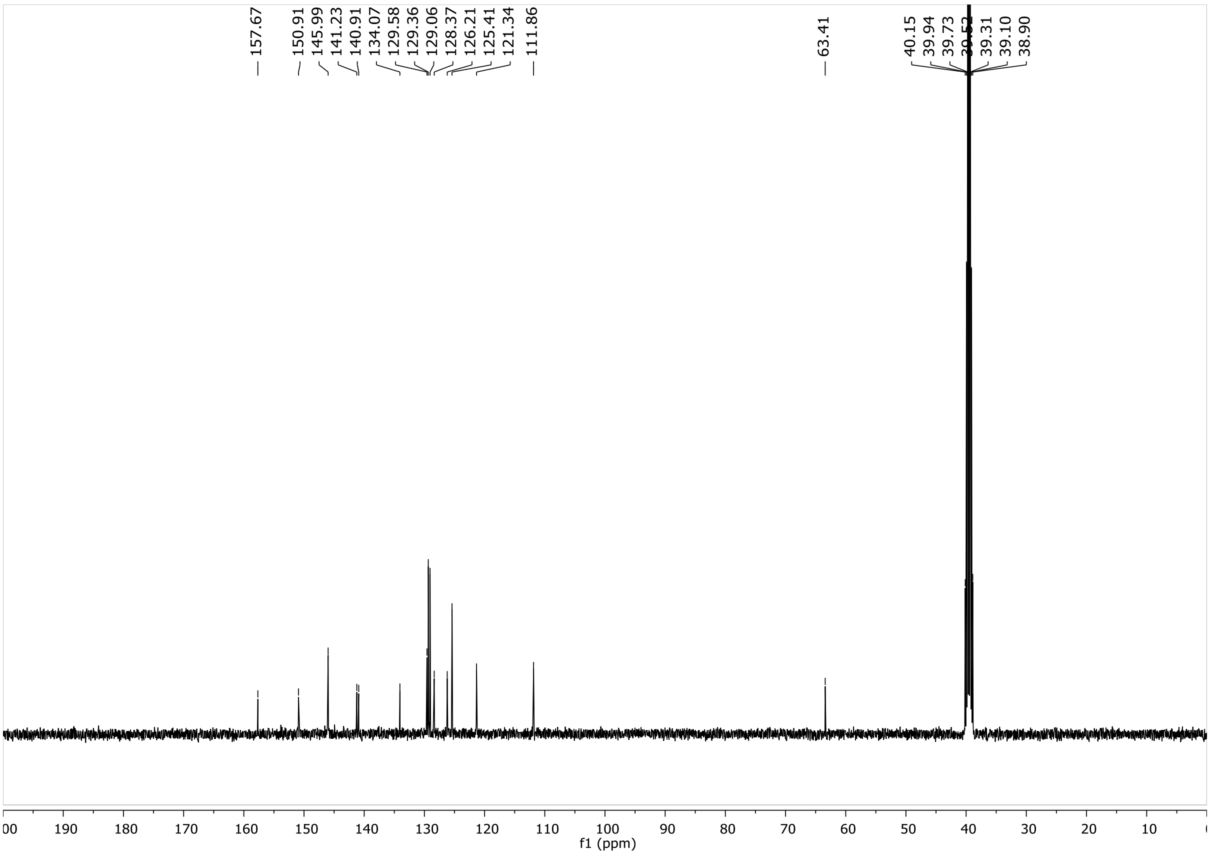
157.67
150.91
145.99
141.23
140.91
134.07
129.58
129.36
129.06
128.37
126.21
125.41
121.34
111.86
63.41
40.15
39.94
39.73
39.31
39.10
38.90
f1 (ppm)

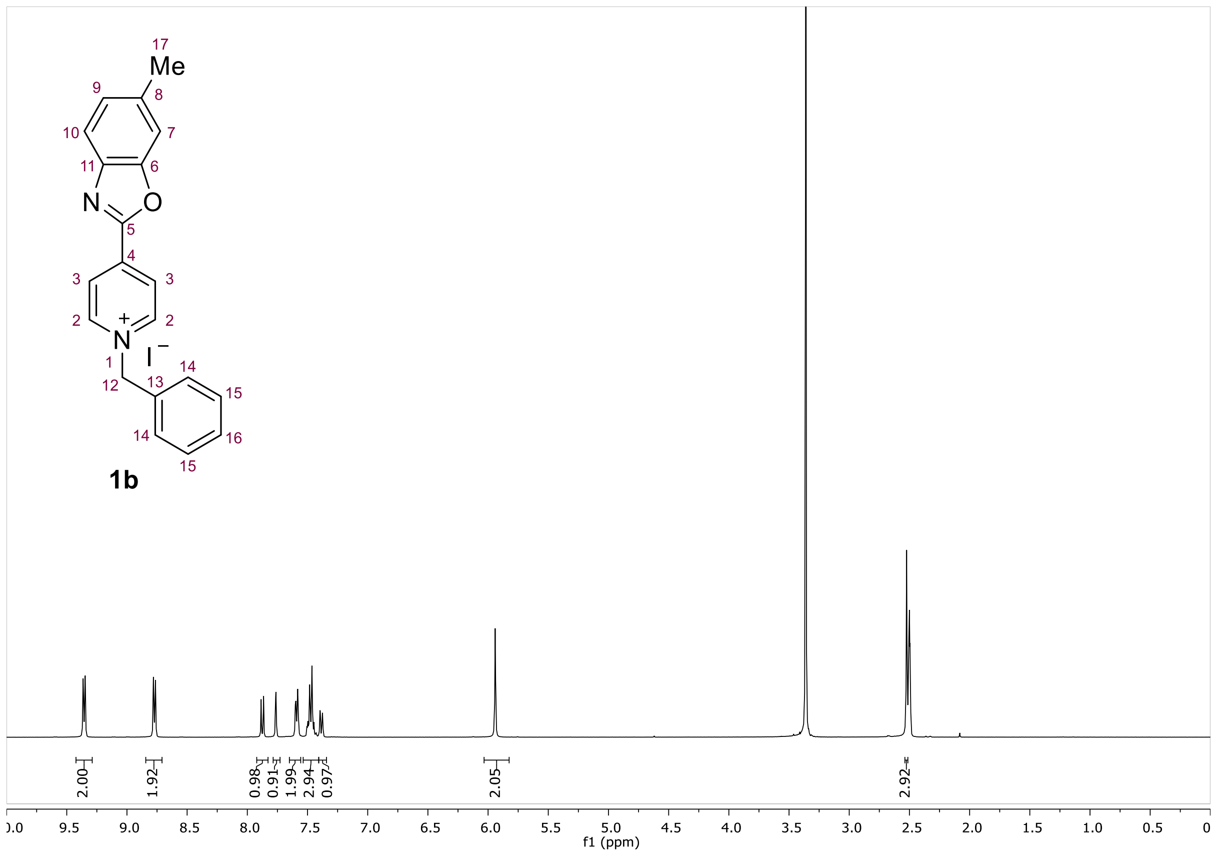

17
Me
1b
2.00
1.92
0.98
0.91
1.99
2.94
0.97
2.05
2.92
f1 (ppm)

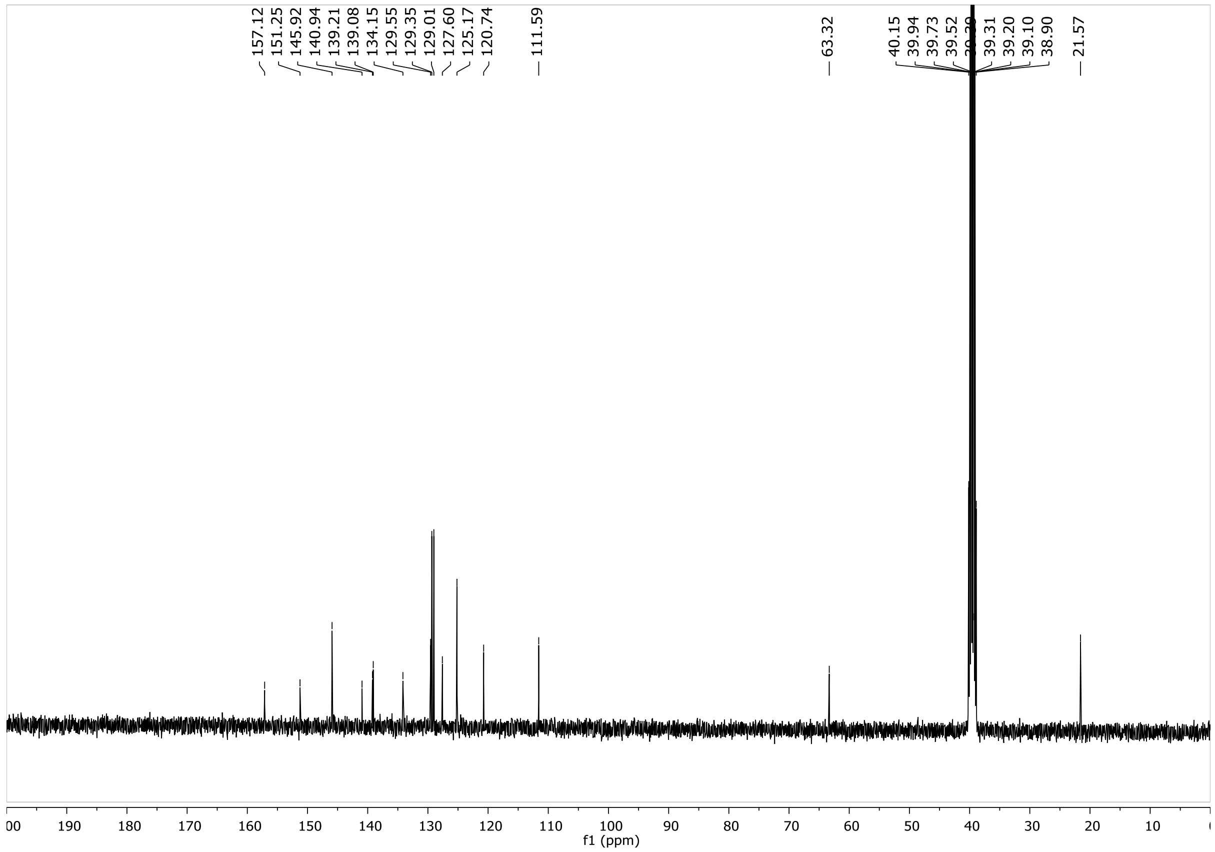

157.12
151.25
145.92
140.94
139.21
139.08
134.15
129.55
129.35
129.01
127.60
125.17
120.74
111.59
63.32
40.15
39.94
39.73
39.52
39.31
39.20
39.10
38.90
21.57
f1 (ppm)

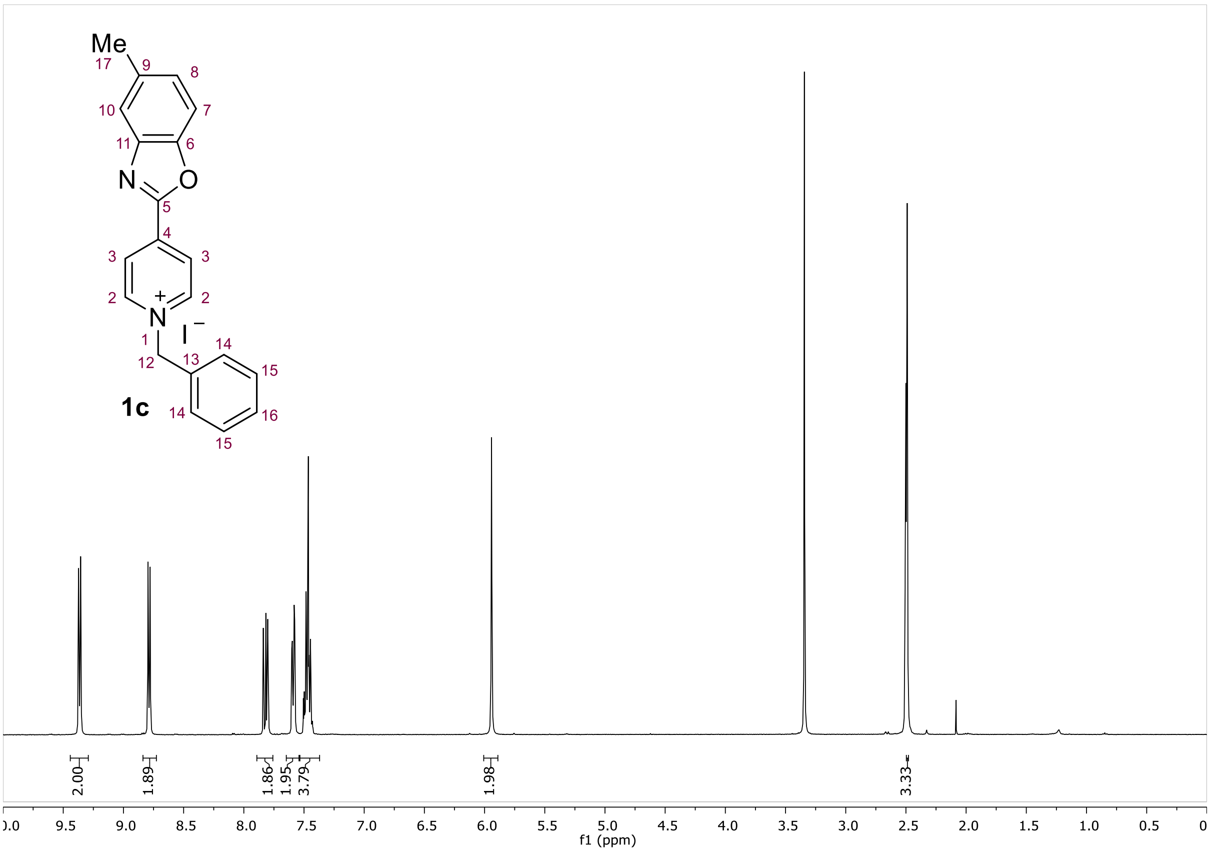
Me
1c
2.00
1.89
1.86
1.95
3.79
1.98
3.33
f1 (ppm)

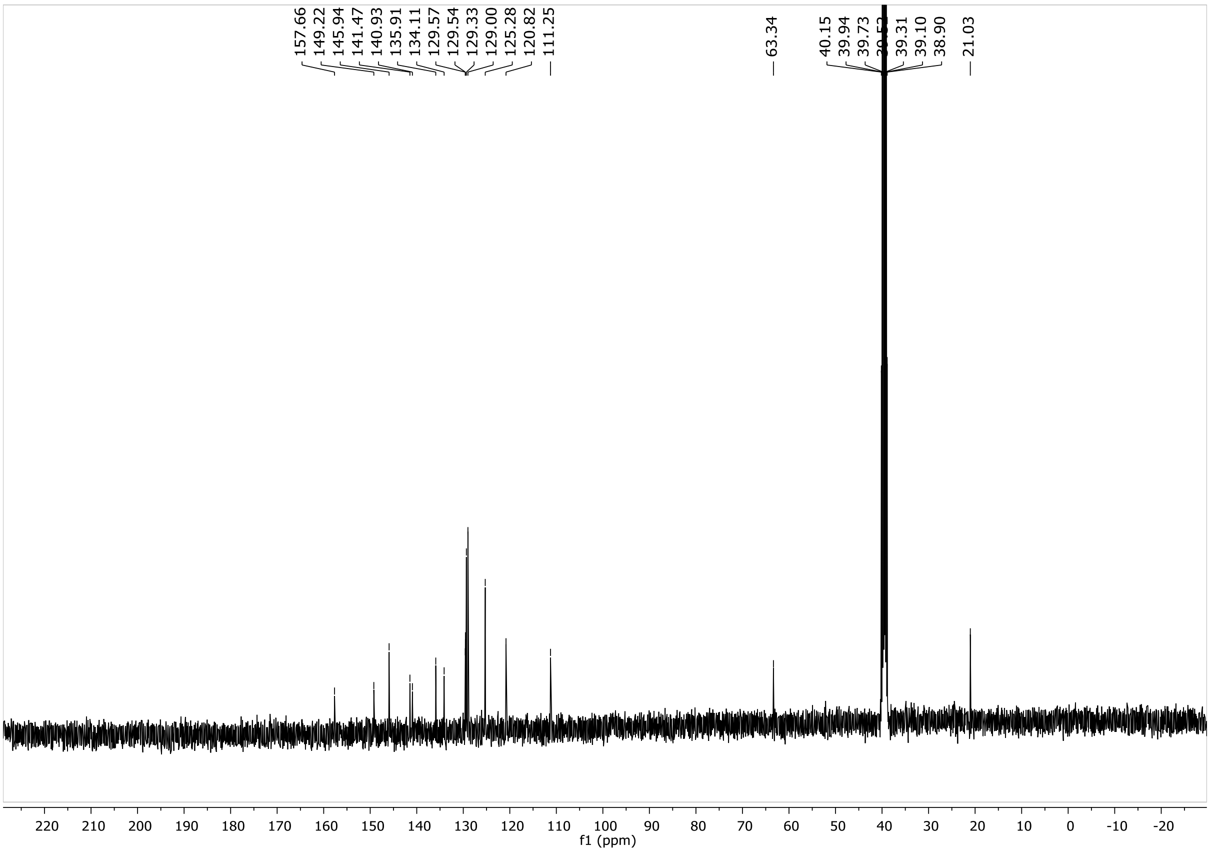
157.66
149.22
145.94
141.47
140.93
135.91
134.11
129.57
129.54
129.33
129.00
125.28
120.82
111.25
63.34
40.15
39.94
39.73
39.31
39.10
38.90
21.03
f1 (ppm)

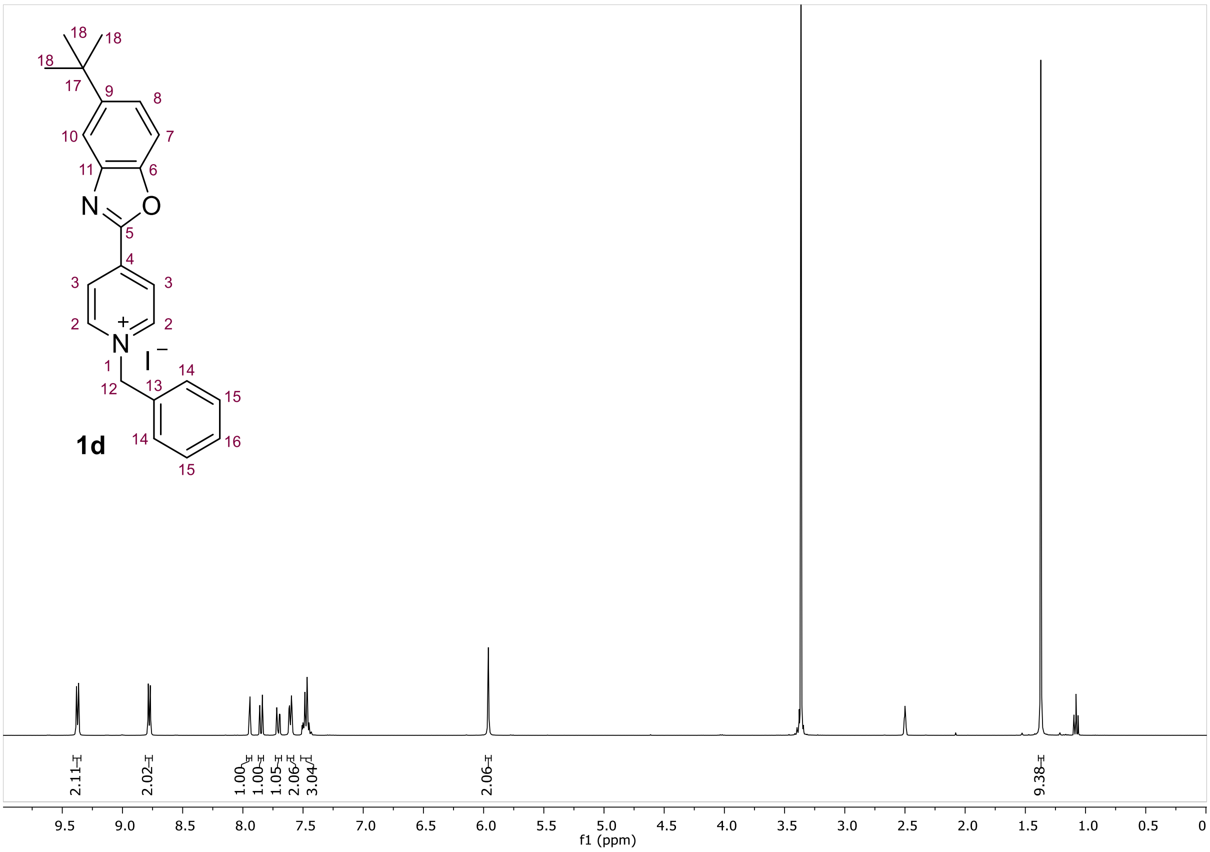
1d
2.11
2.02
1.00
1.00
1.05
2.06
3.04
2.06
9.38
9.5 9.0 8.5 8.0 7.5 7.0 6.5 6.0 5.5 5.0 4.5 4.0 3.5 3.0 2.5 2.0 1.5 1.0 0.5 0
f1 (ppm)

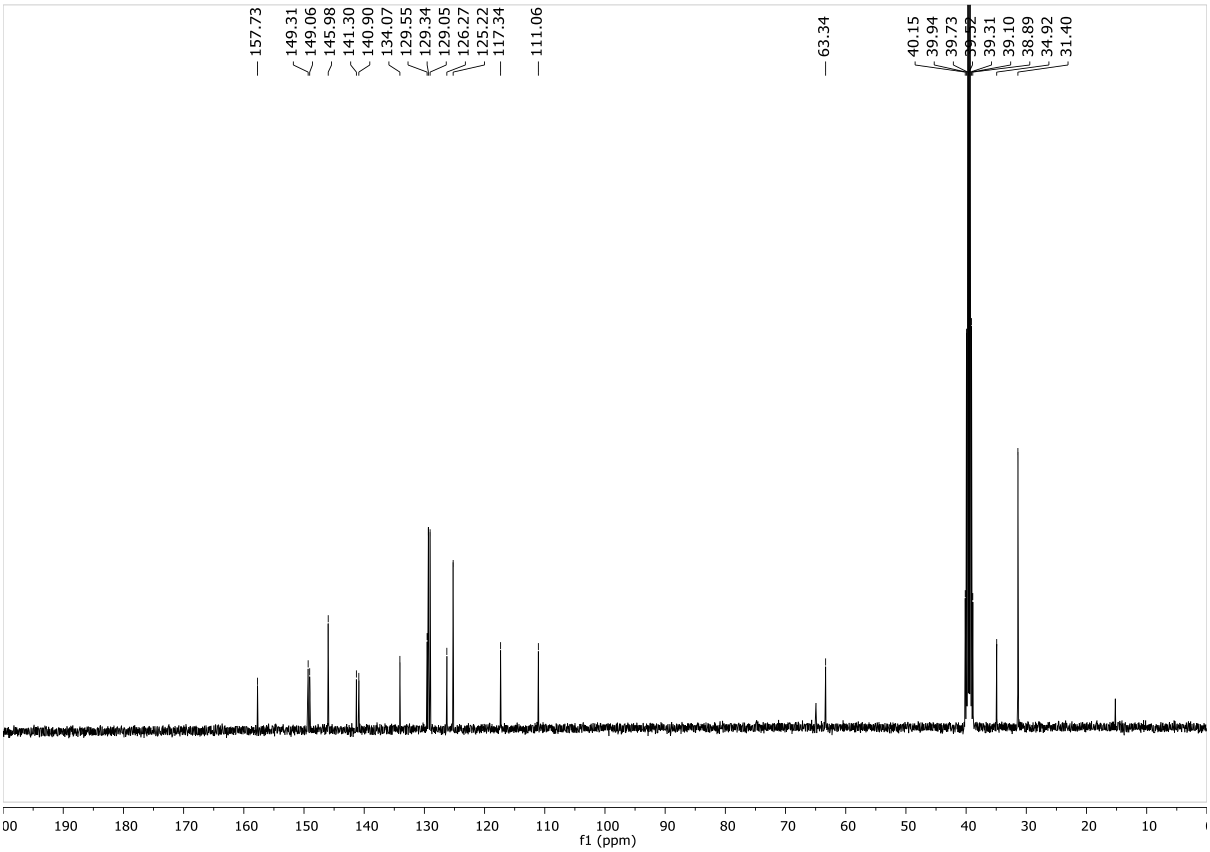
157.73
149.31
149.06
145.98
141.30
140.90
134.07
129.55
129.34
129.05
126.27
125.22
117.34
111.06
63.34
40.15
39.94
39.73
39.52
39.31
39.10
38.89
34.92
31.40
190 180 170 160 150 140 130 120 110 100 90 80 70 60 50 40 30 20 10
f1 (ppm)

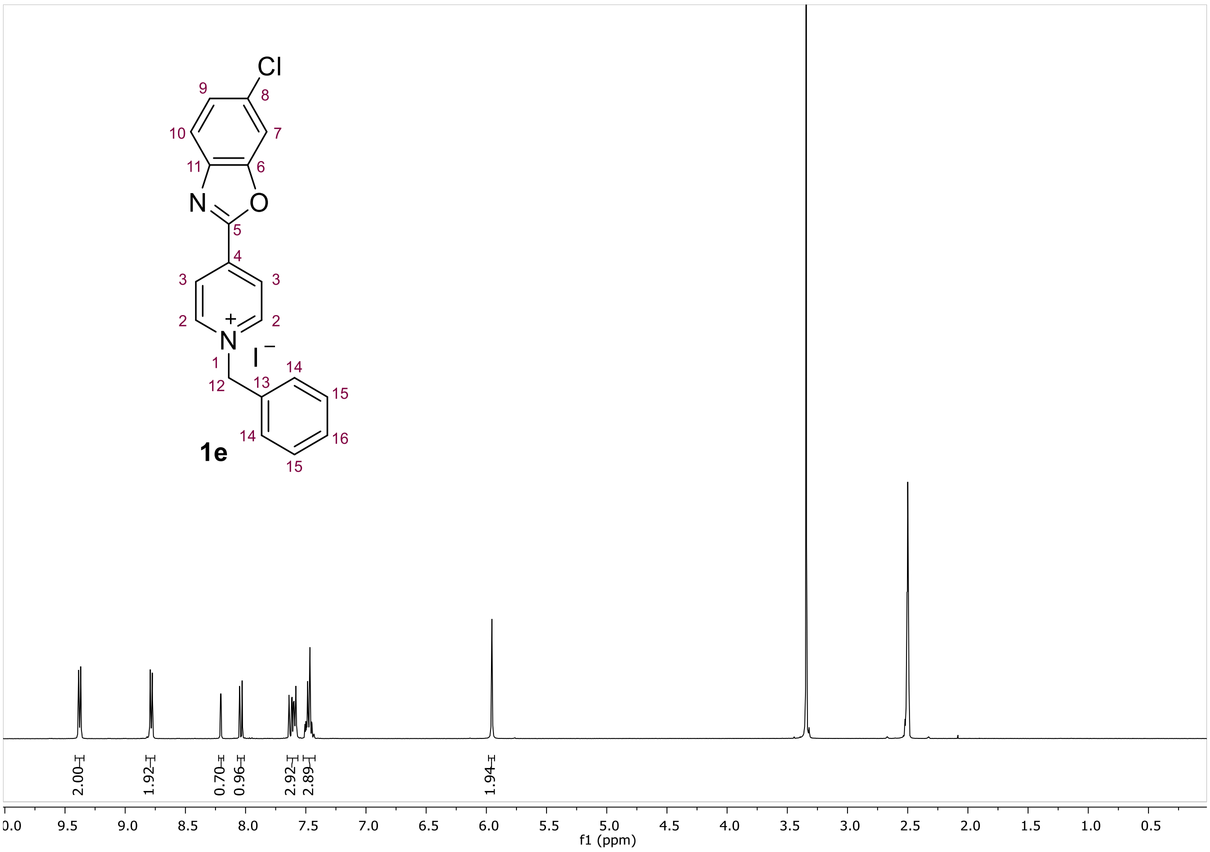
Cl
1e
2.00
1.92
0.70
0.96
2.92
2.89
1.94
f1 (ppm)

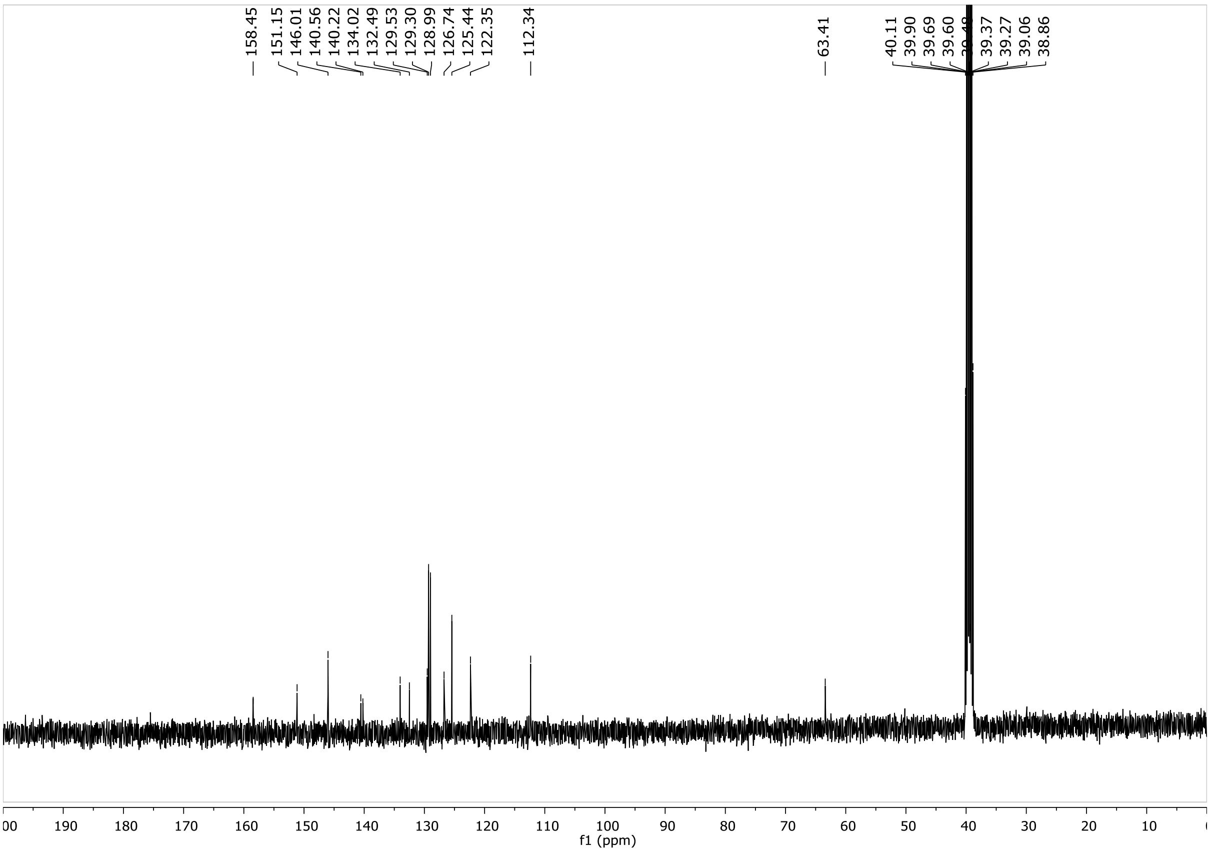
158.45
151.15
146.01
140.56
140.22
134.02
132.49
129.53
129.30
128.99
126.74
125.44
122.35
112.34
63.41
40.11
39.90
39.69
39.60
39.37
39.27
39.06
38.86
f1 (ppm)

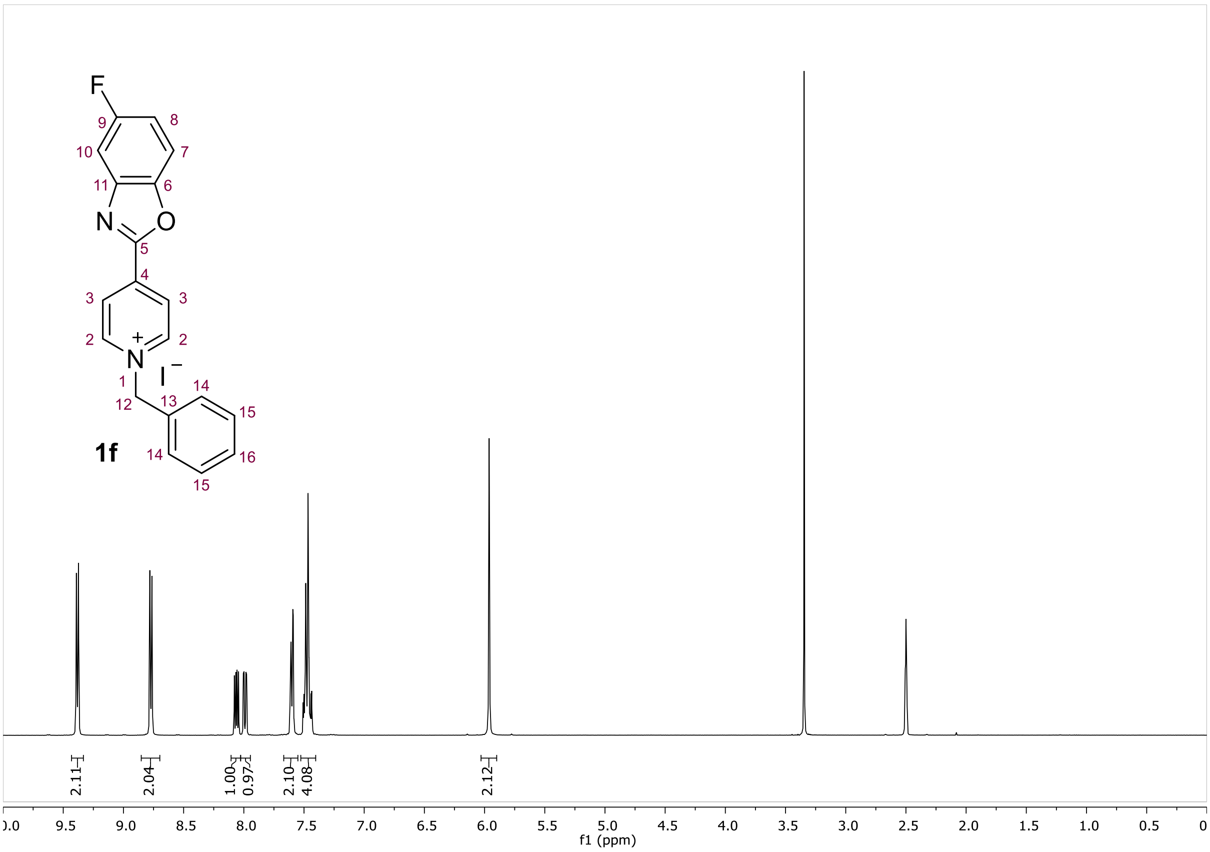
F
N
O
I⁻
1f
2.11
2.04
1.00
0.97
2.10
4.08
2.12
f1 (ppm)

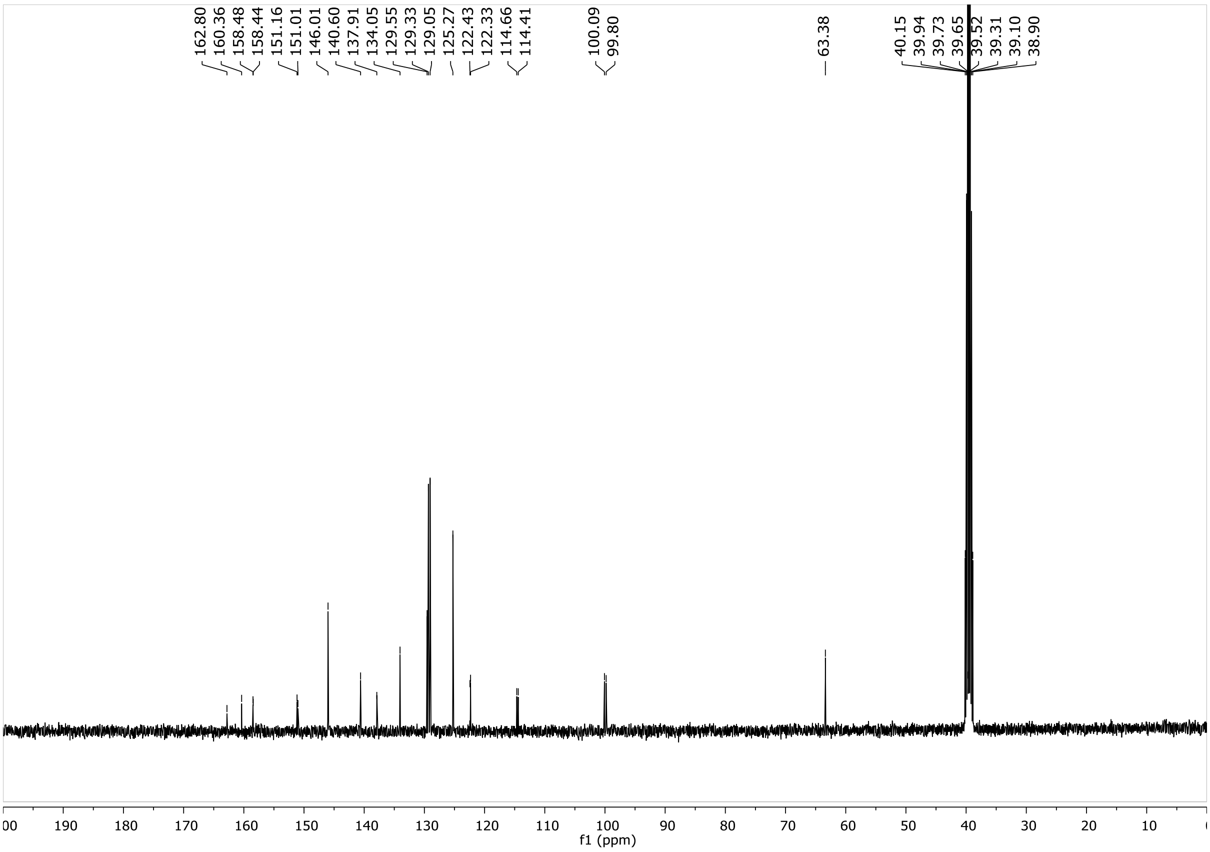
162.80
160.36
158.48
158.44
151.16
151.01
146.01
140.60
137.91
134.05
129.55
129.33
129.05
125.27
122.43
122.33
114.66
114.41
100.09
99.80
63.38
40.15
39.94
39.73
39.65
39.52
39.31
39.10
38.90
f1 (ppm)

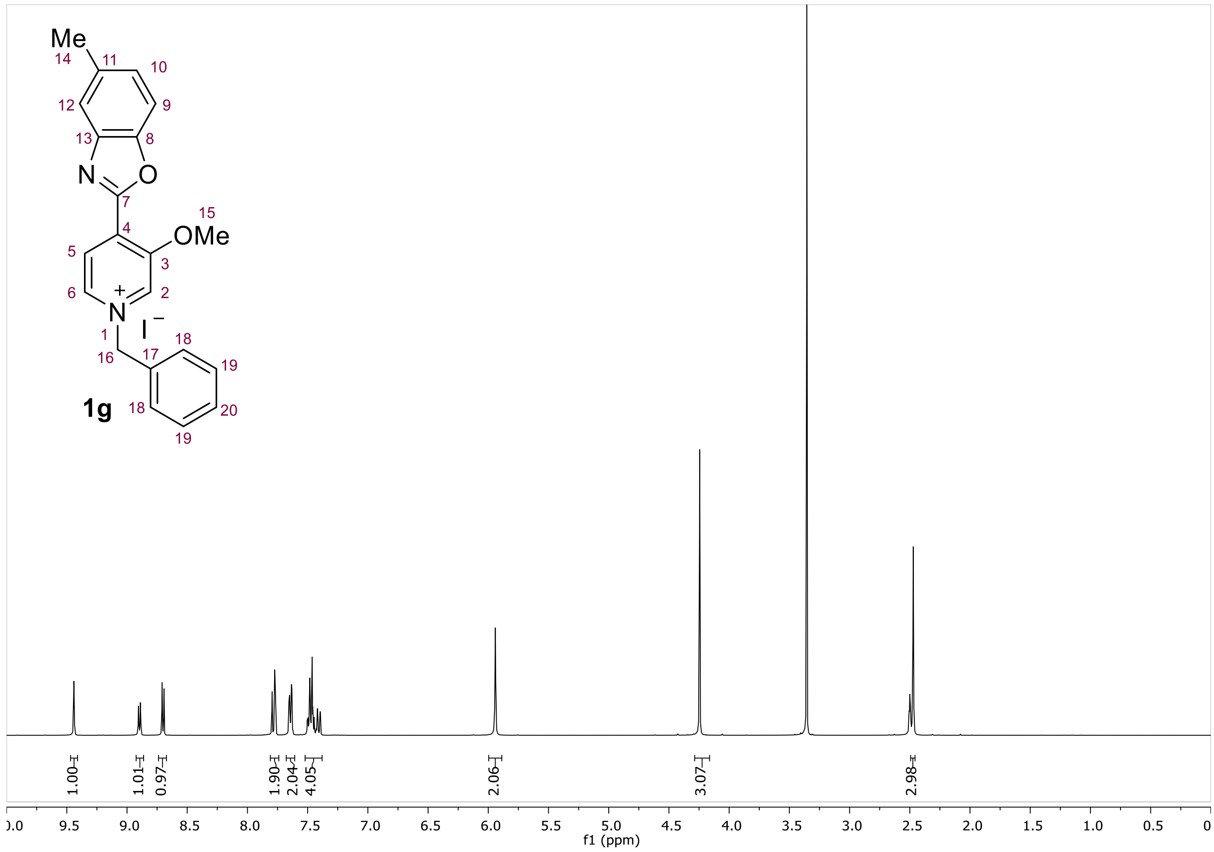
Me
OMe
N
O
I
1g
1.00
1.01
0.97
1.90
2.04
4.05
2.06
3.07
2.98
f1 (ppm)

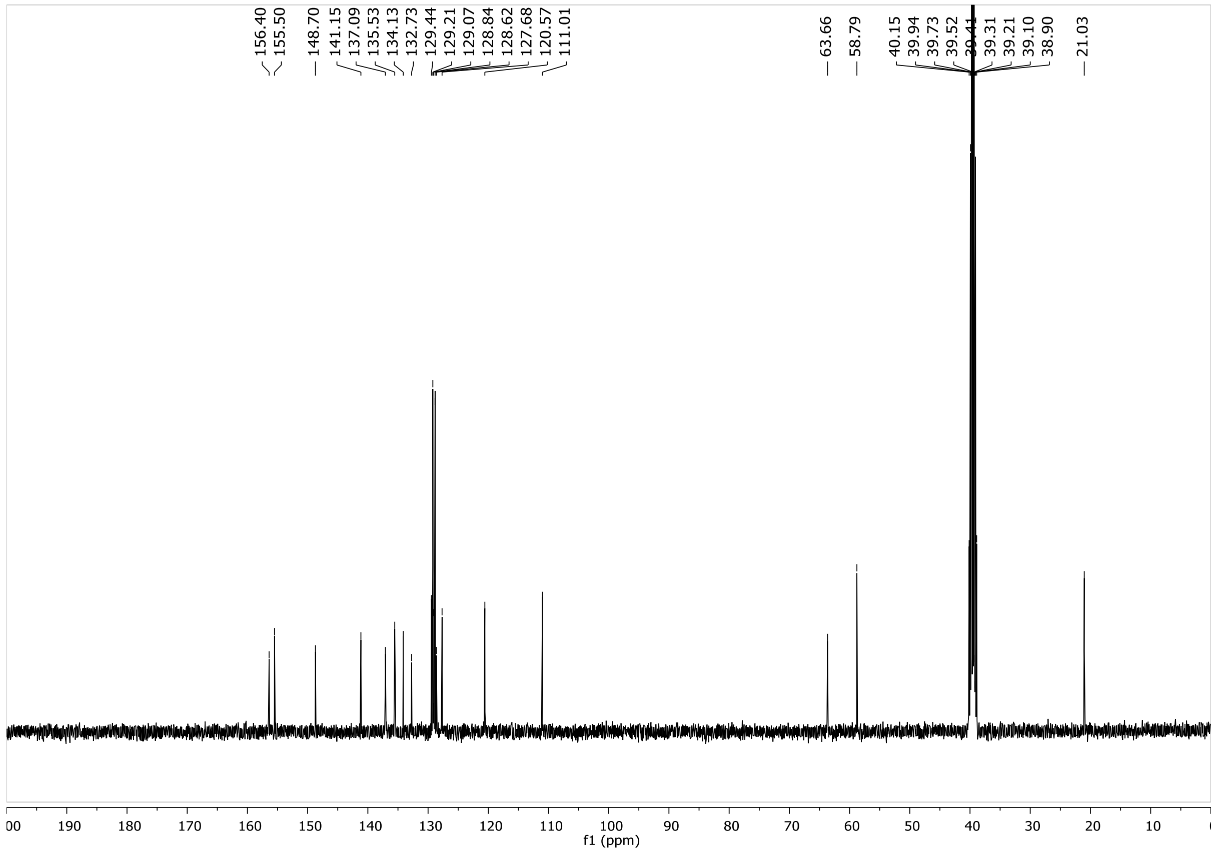
156.40
155.50
148.70
141.15
137.09
135.53
134.13
132.73
129.44
129.21
129.07
128.84
128.62
127.68
120.57
111.01
63.66
58.79
40.15
39.94
39.73
39.52
39.31
39.21
39.10
38.90
21.03
f1 (ppm)

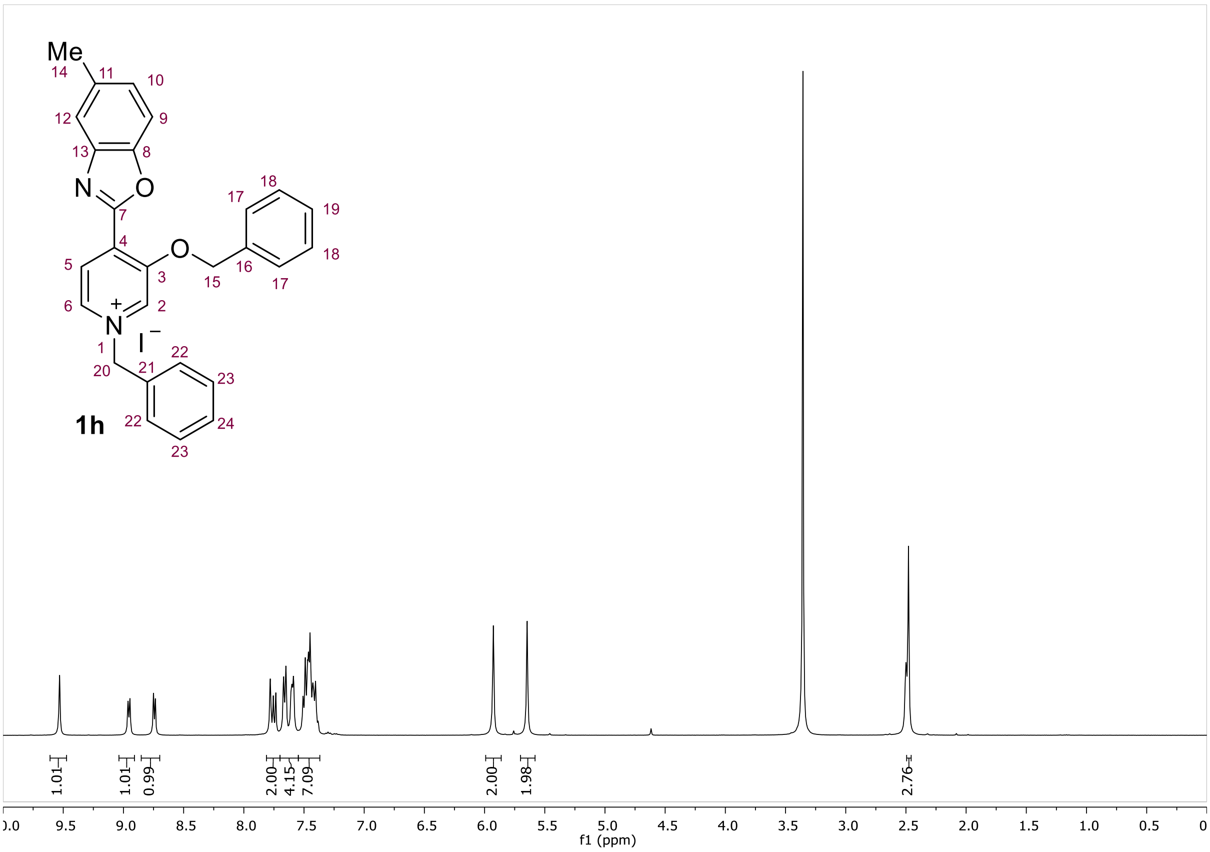
Me
1h
1.01
1.01
0.99
2.00
4.15
7.09
2.00
1.98
2.76
f1 (ppm)

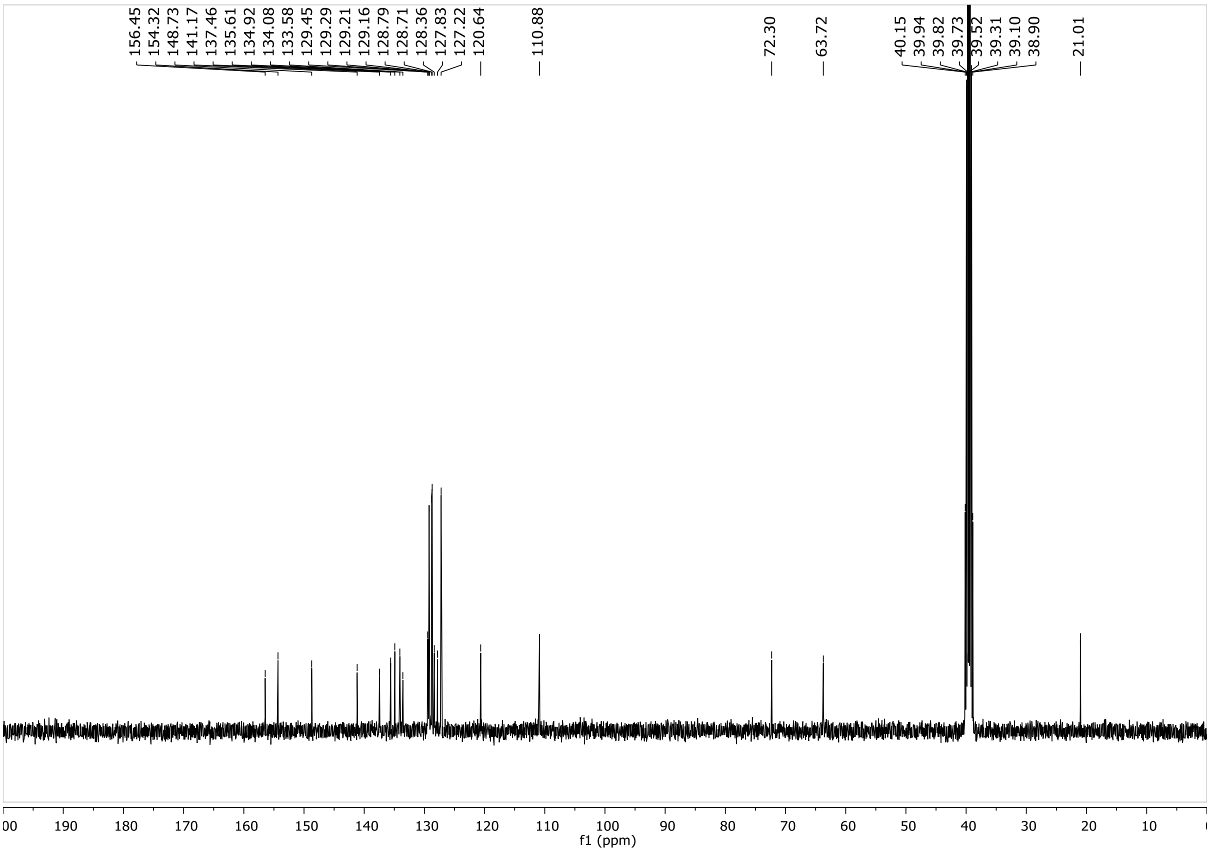
156.45
154.32
148.73
141.17
137.46
135.61
134.92
134.08
133.58
129.45
129.29
129.21
129.16
128.79
128.71
128.36
127.83
127.22
120.64
110.88
72.30
63.72
40.15
39.94
39.82
39.73
39.52
39.31
39.10
38.90
21.01
f1 (ppm)

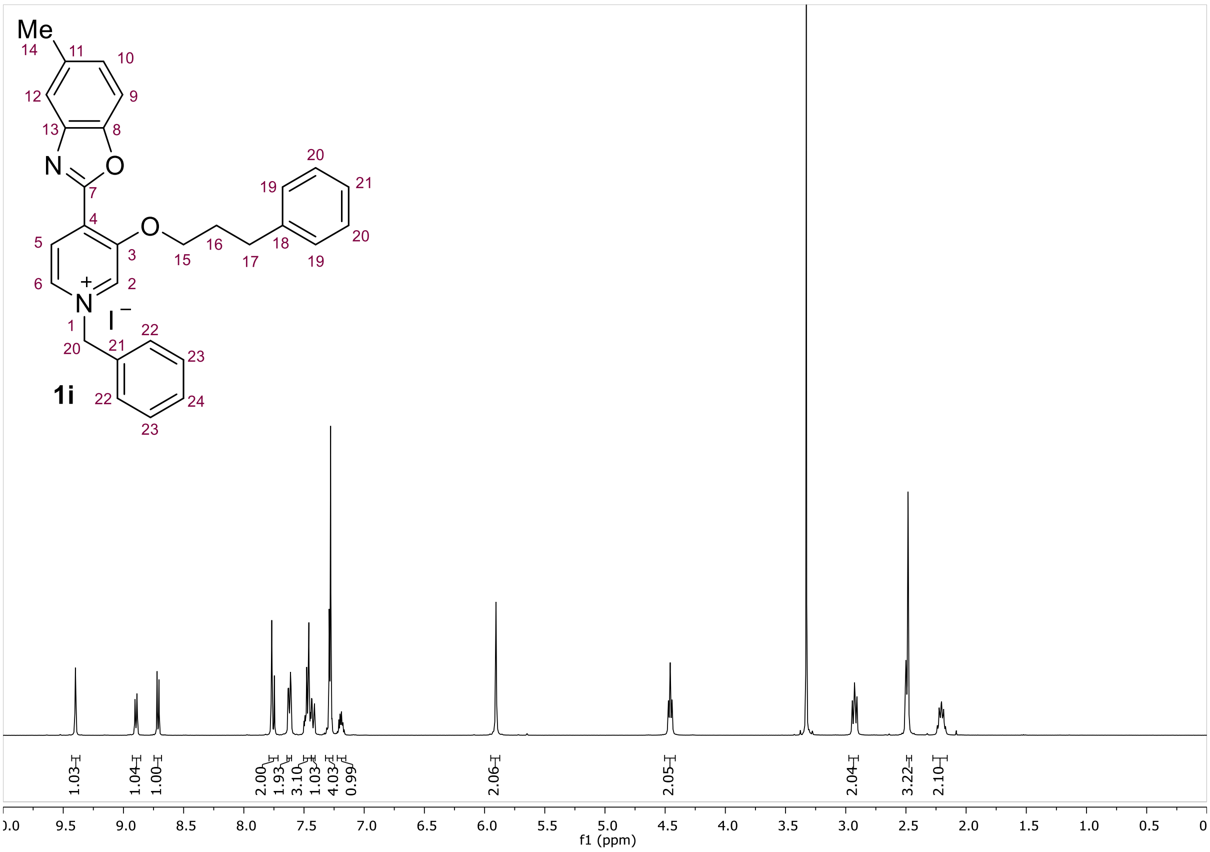

Me
N
O
I⁻
1i
1.03
1.04
1.00
2.00
1.93
3.10
1.03
4.03
0.99
2.06
2.05
2.04
3.22
2.10
f1 (ppm)

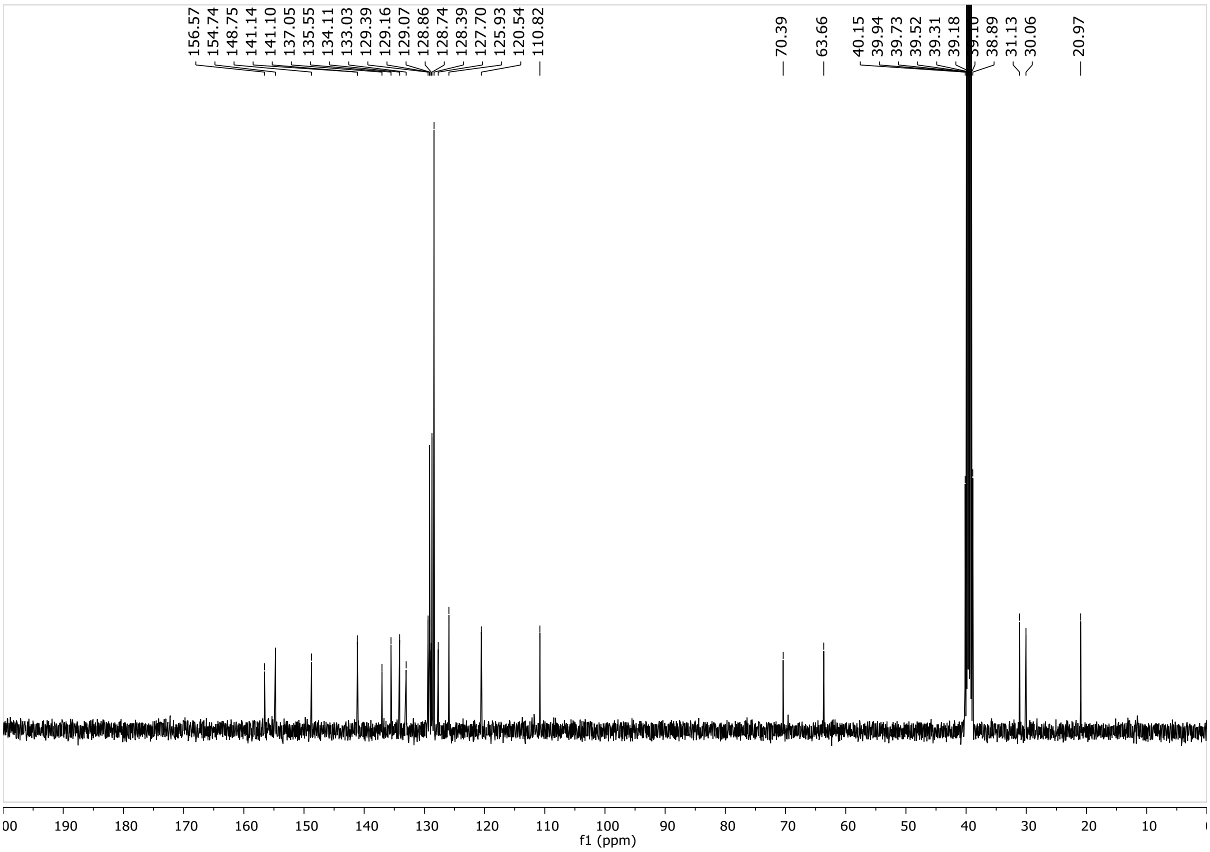

156.57
154.74
148.75
141.14
141.10
137.05
135.55
134.11
133.03
129.39
129.16
129.07
128.86
128.74
128.39
127.70
125.93
120.54
110.82
70.39
63.66
40.15
39.94
39.73
39.52
39.31
39.18
38.89
31.13
30.06
20.97
f1 (ppm)

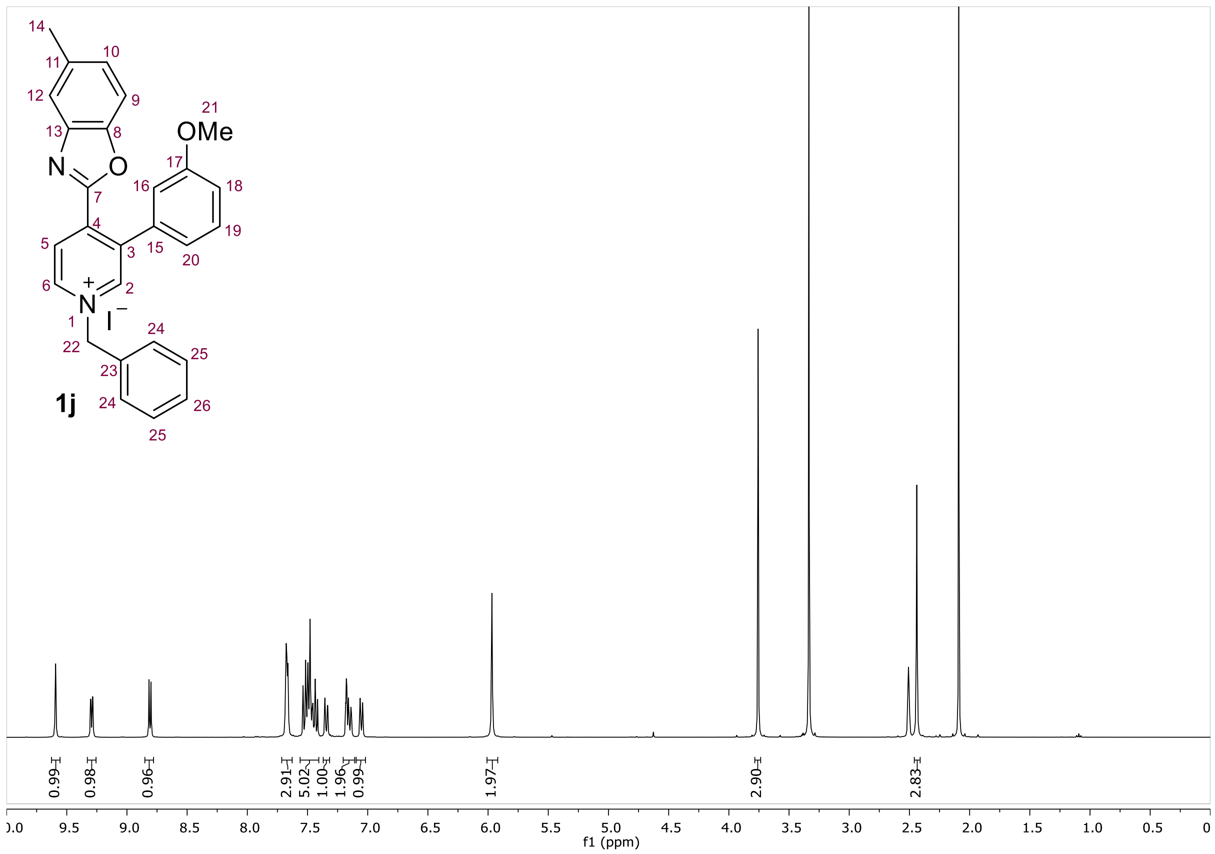
1j
OMe
0.99
0.98
0.96
2.91
5.02
1.00
1.96
0.99
1.97
2.90
2.83
f1 (ppm)

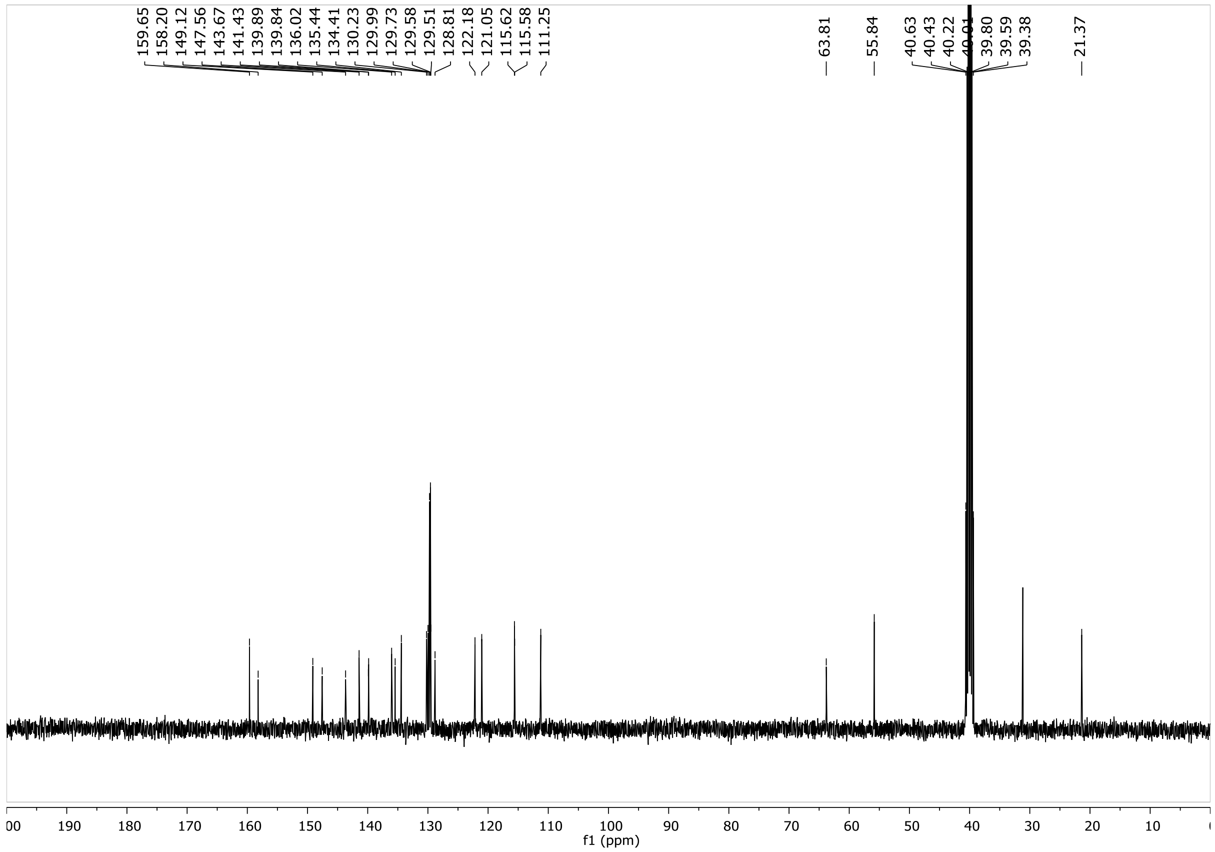
159.65
158.20
149.12
147.56
143.67
141.43
139.89
139.84
136.02
135.44
134.41
130.23
129.99
129.73
129.58
129.51
128.81
122.18
121.05
115.62
115.58
111.25
63.81
55.84
40.63
40.43
40.22
39.80
39.59
39.38
21.37
f1 (ppm)

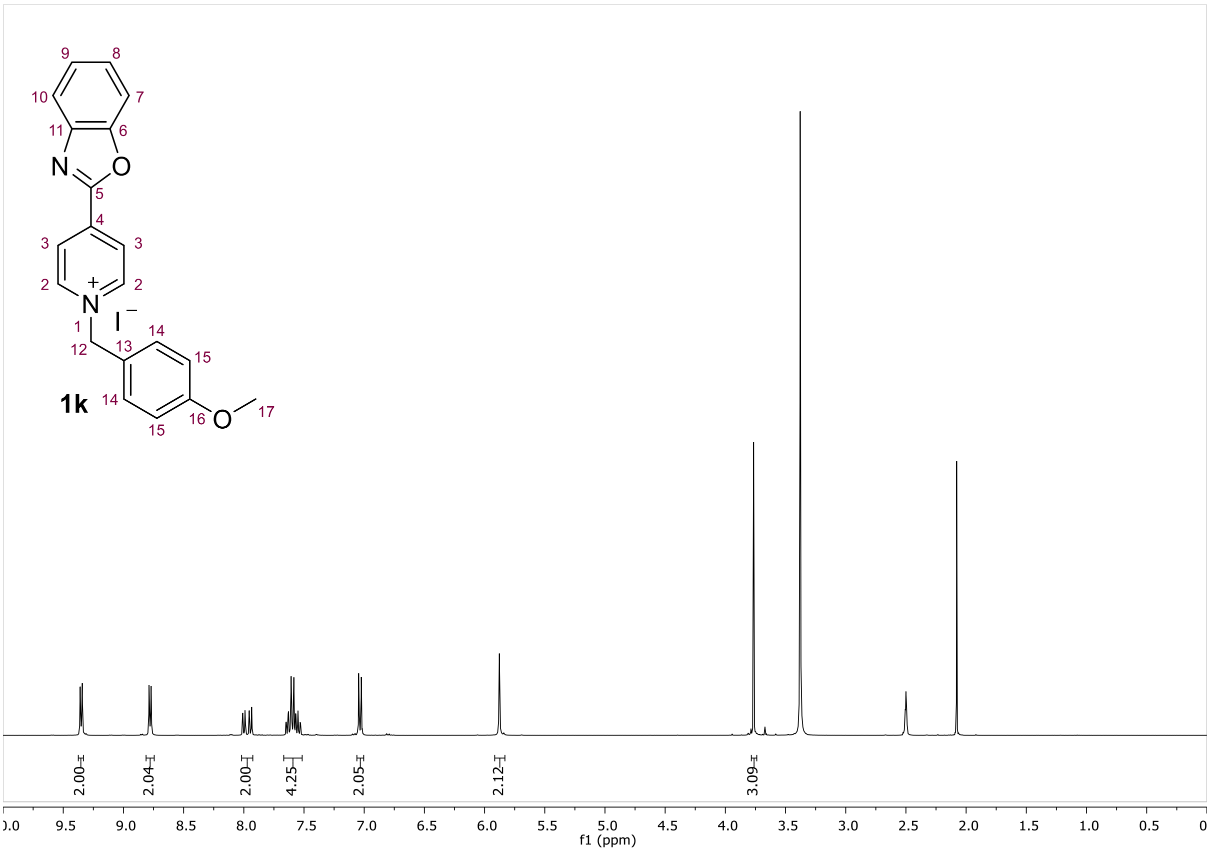
1k
2.00
2.04
2.00
4.25
2.05
2.12
3.09
f1 (ppm)

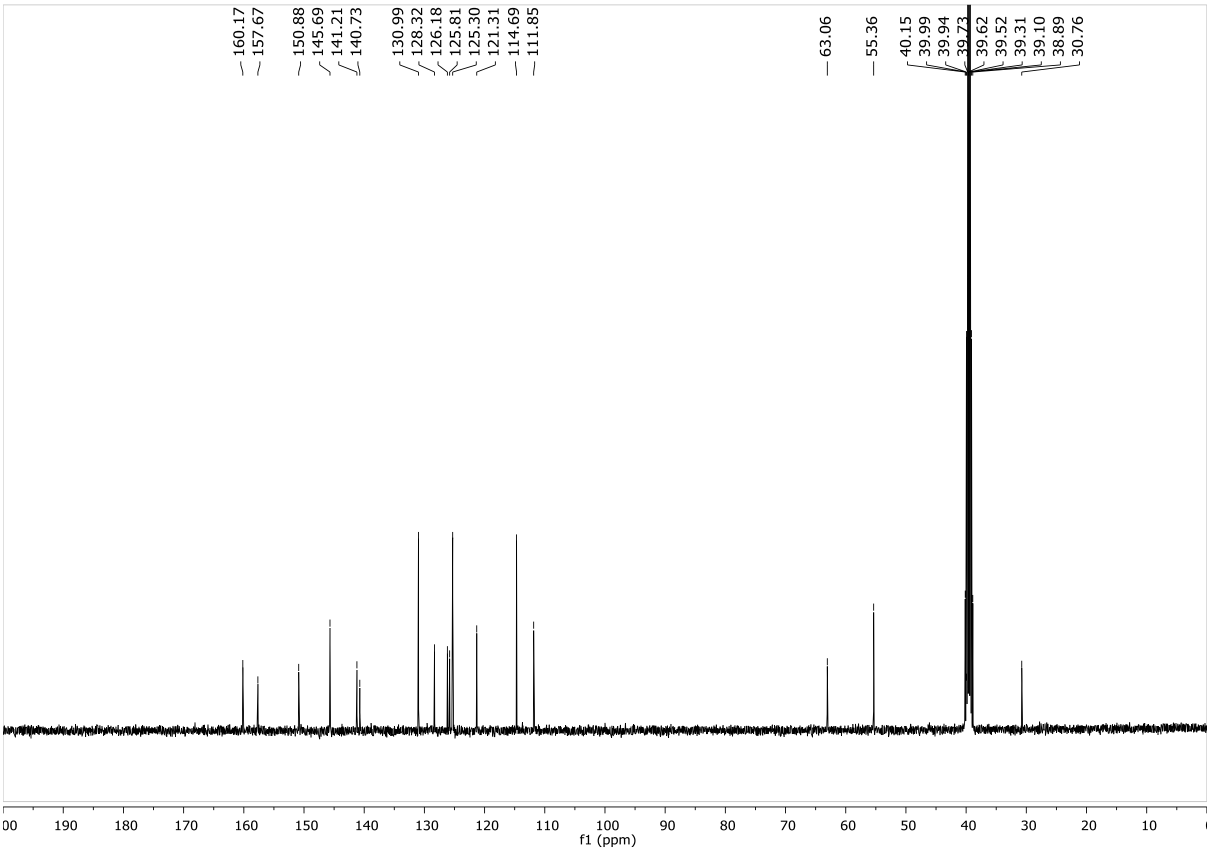
160.17
157.67
150.88
145.69
141.21
140.73
130.99
128.32
126.18
125.81
125.30
121.31
114.69
111.85
63.06
55.36
40.15
39.99
39.94
39.73
39.62
39.52
39.31
39.10
38.89
30.76
f1 (ppm)

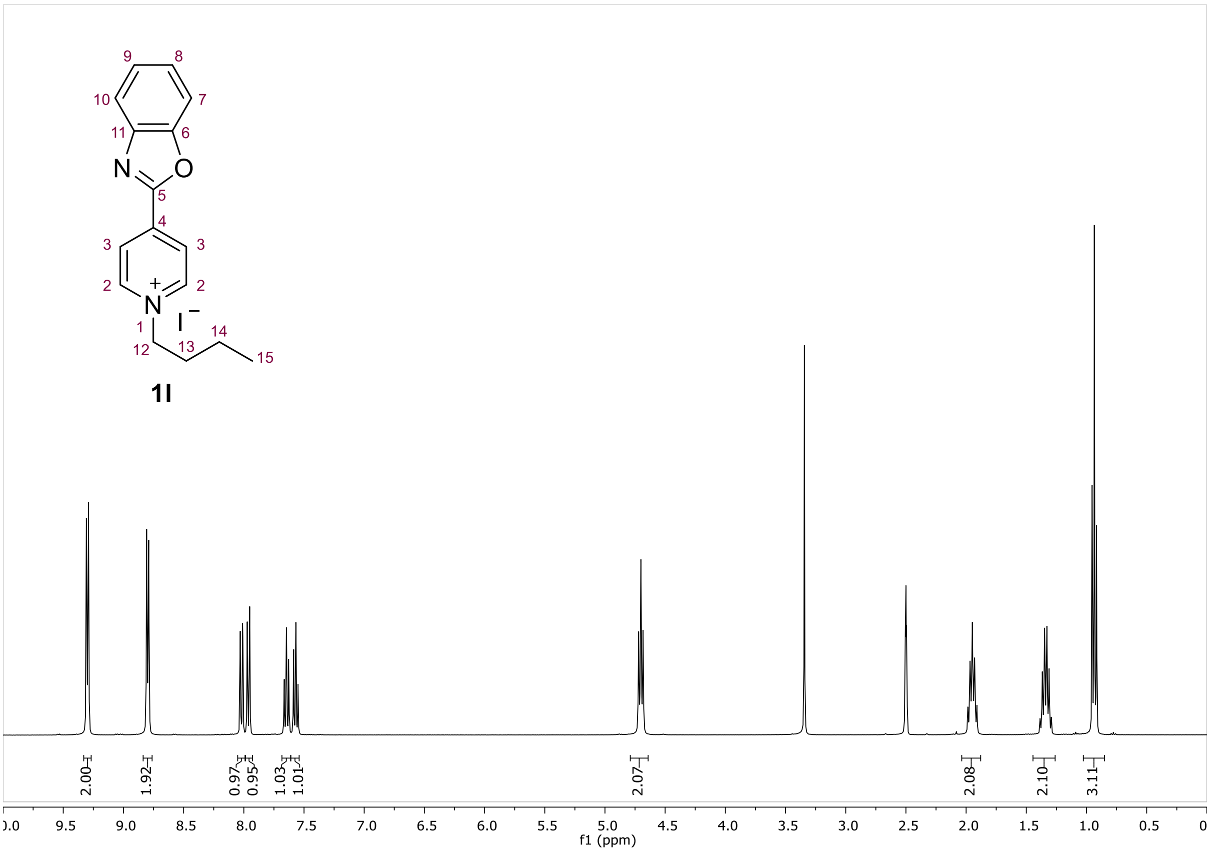
1l
2.00
1.92
0.97
0.95
1.03
1.01
2.07
2.08
2.10
3.11
f1 (ppm)

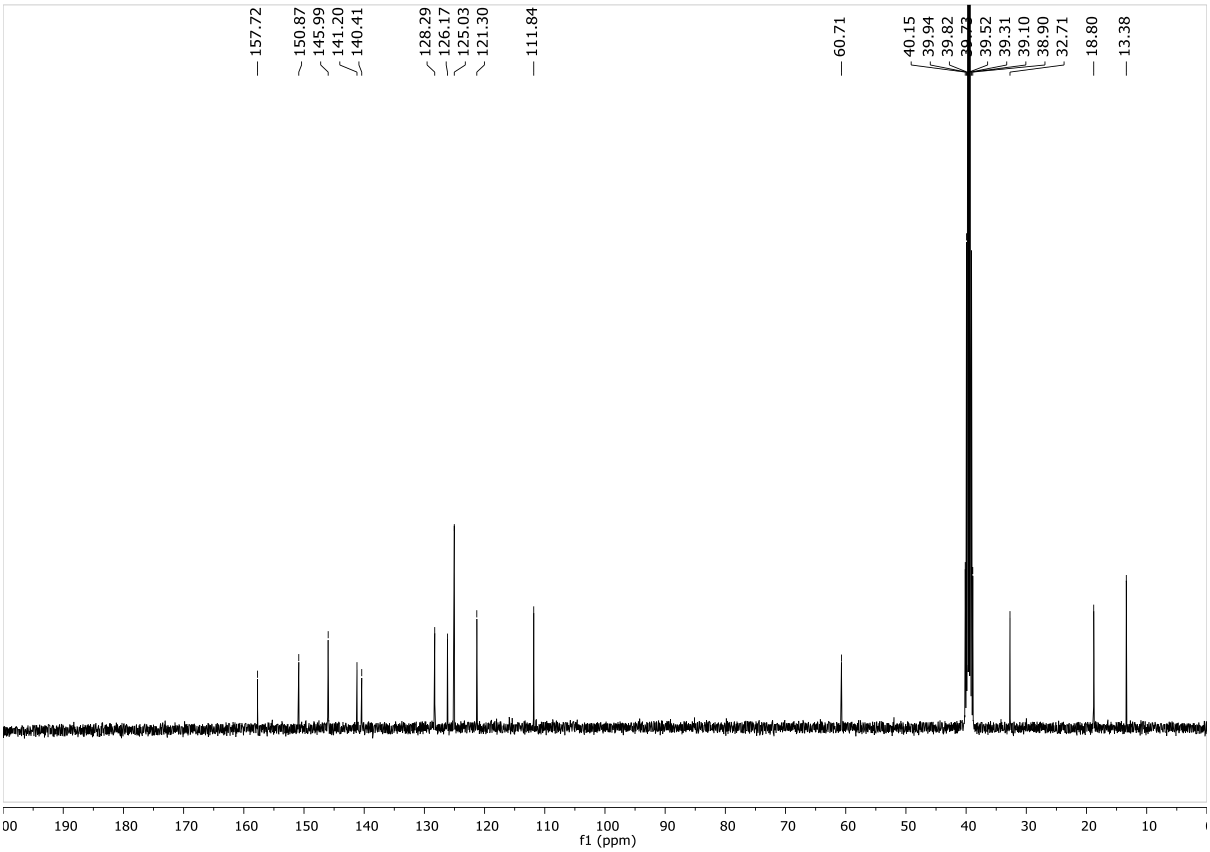
157.72
150.87
145.99
141.20
140.41
128.29
126.17
125.03
121.30
111.84
60.71
40.15
39.94
39.82
39.73
39.52
39.31
39.10
38.90
32.71
18.80
13.38
f1 (ppm)

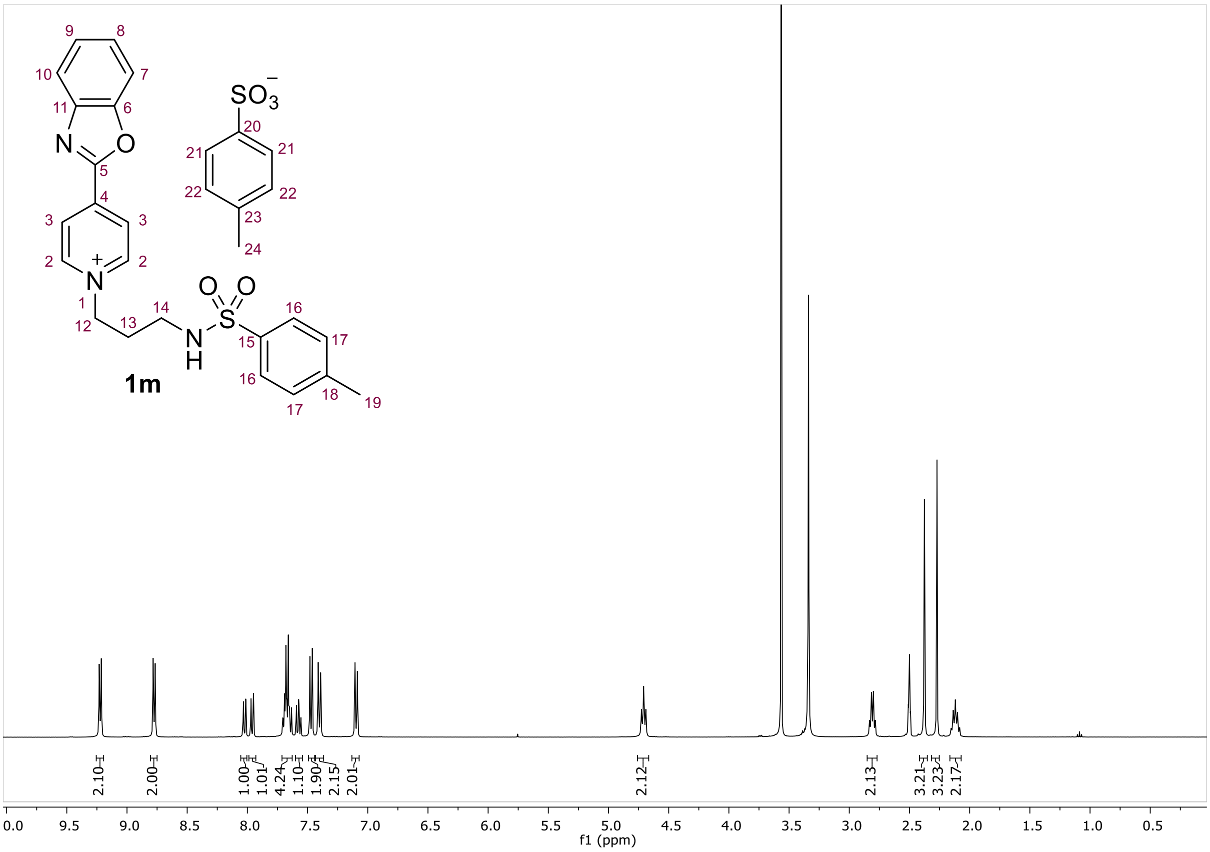
1m
SO3−
2.10
2.00
1.00
1.01
4.24
1.10
1.90
2.15
2.01
2.12
2.13
3.21
3.23
2.17
f1 (ppm)

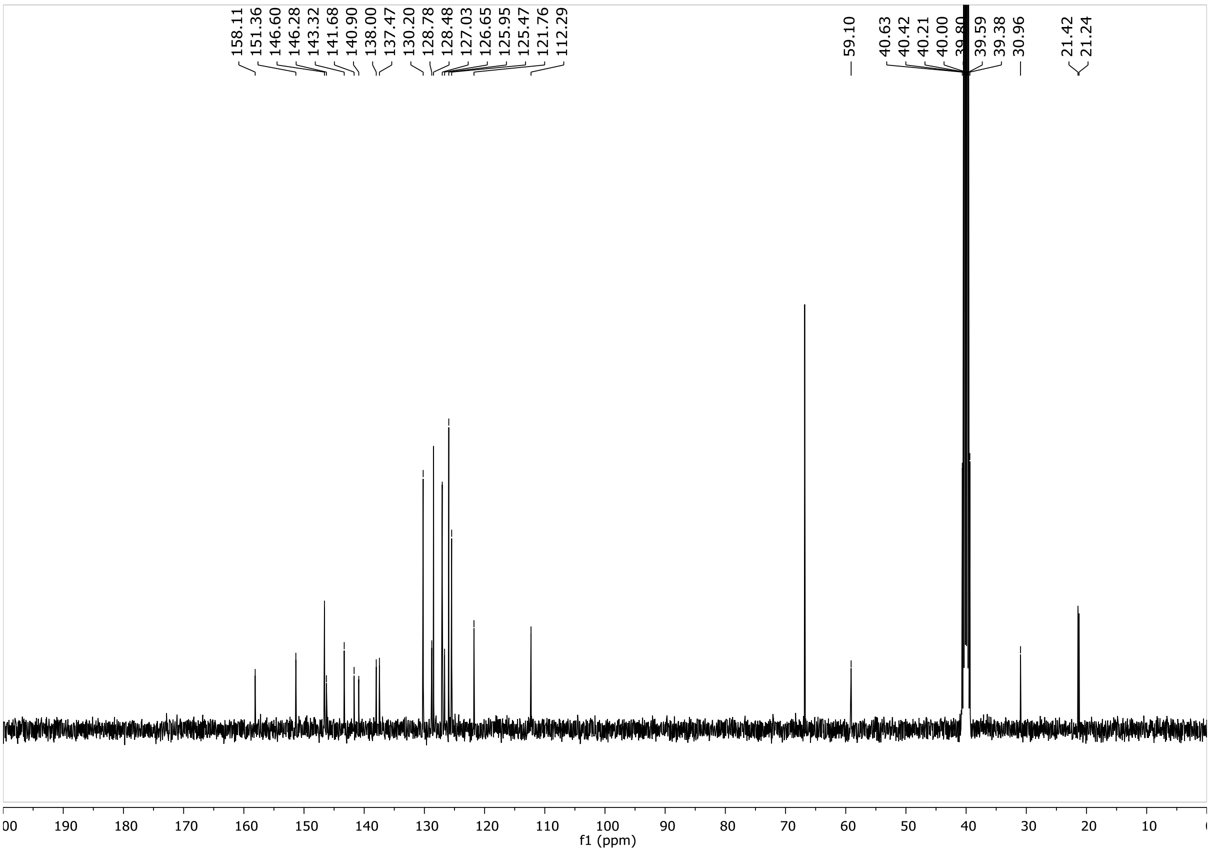
158.11
151.36
146.60
146.28
143.32
141.68
140.90
138.00
137.47
130.20
128.78
128.48
127.03
126.65
125.95
125.47
121.76
112.29
59.10
40.63
40.42
40.21
40.00
39.59
39.38
30.96
21.42
21.24
f1 (ppm)

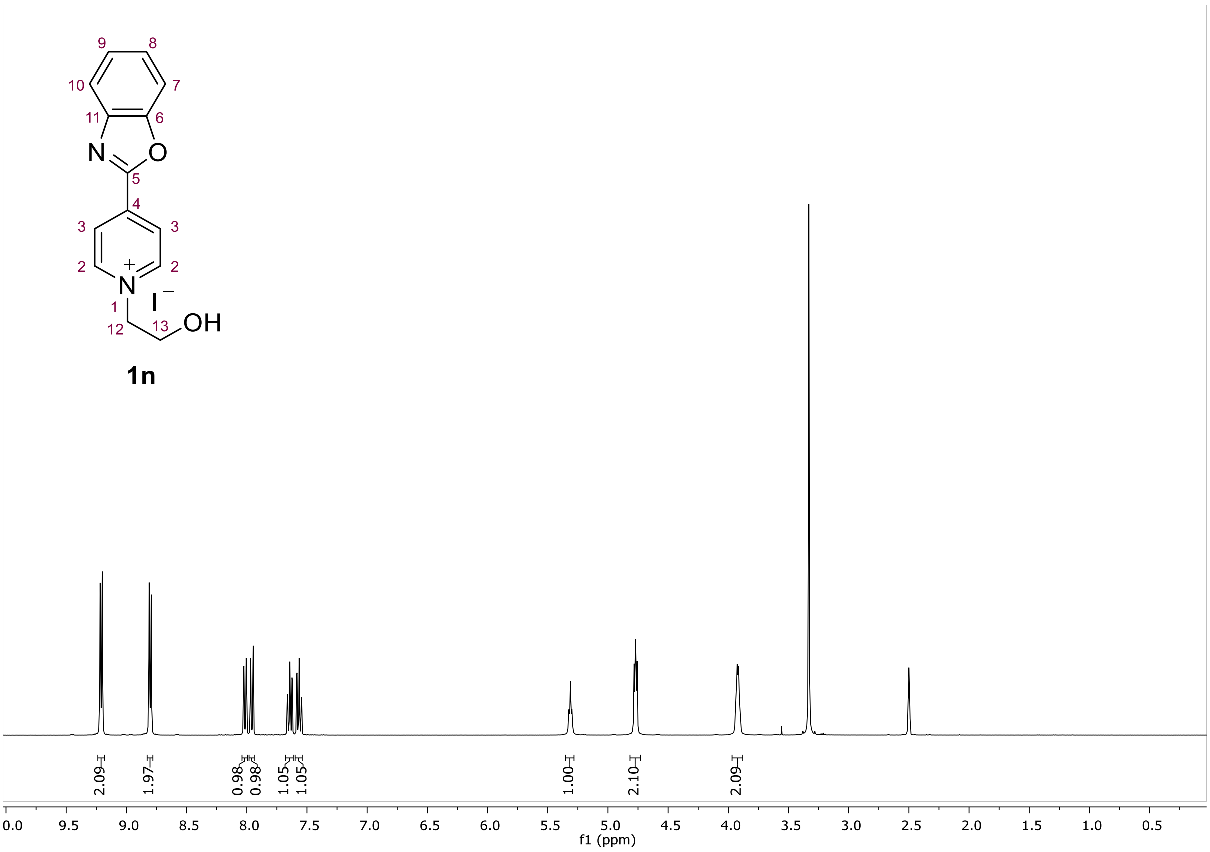

9
8
10
7
11
6
N
O
5
4
3
3
2
2
N
1
I
12
13
OH
1n
2.09
1.97
0.98
0.98
1.05
1.05
1.00
2.10
2.09
f1 (ppm)

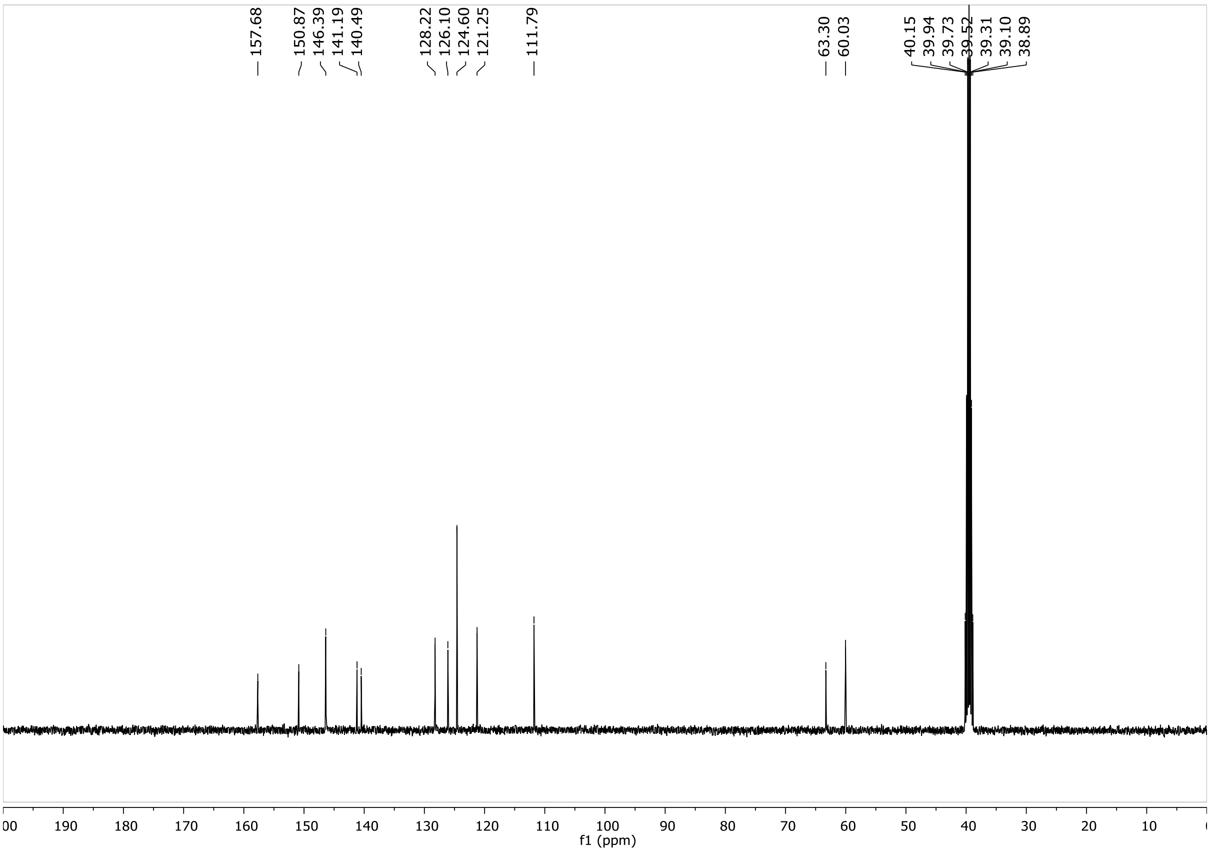

157.68
150.87
146.39
141.19
140.49
128.22
126.10
124.60
121.25
111.79
63.30
60.03
40.15
39.94
39.73
39.52
39.31
39.10
38.89
f1 (ppm)

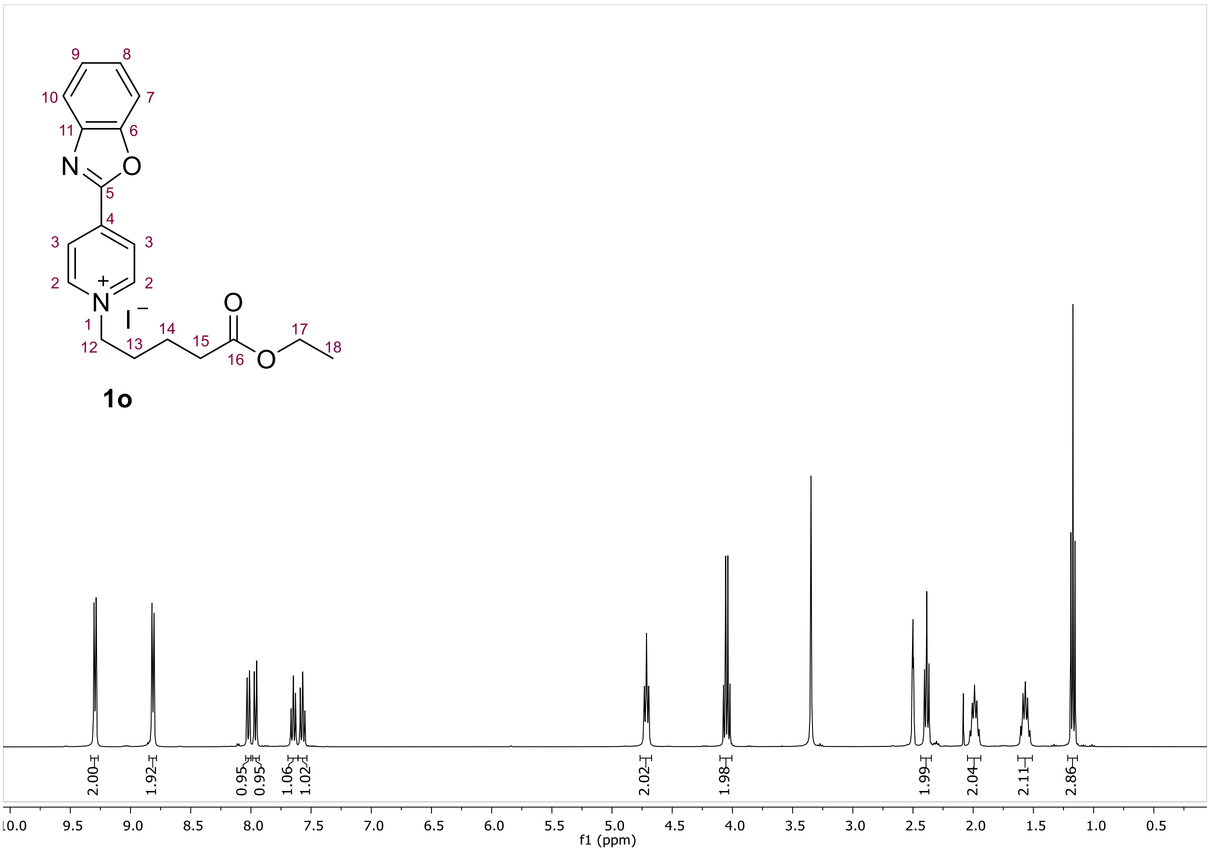
1o
f1 (ppm)

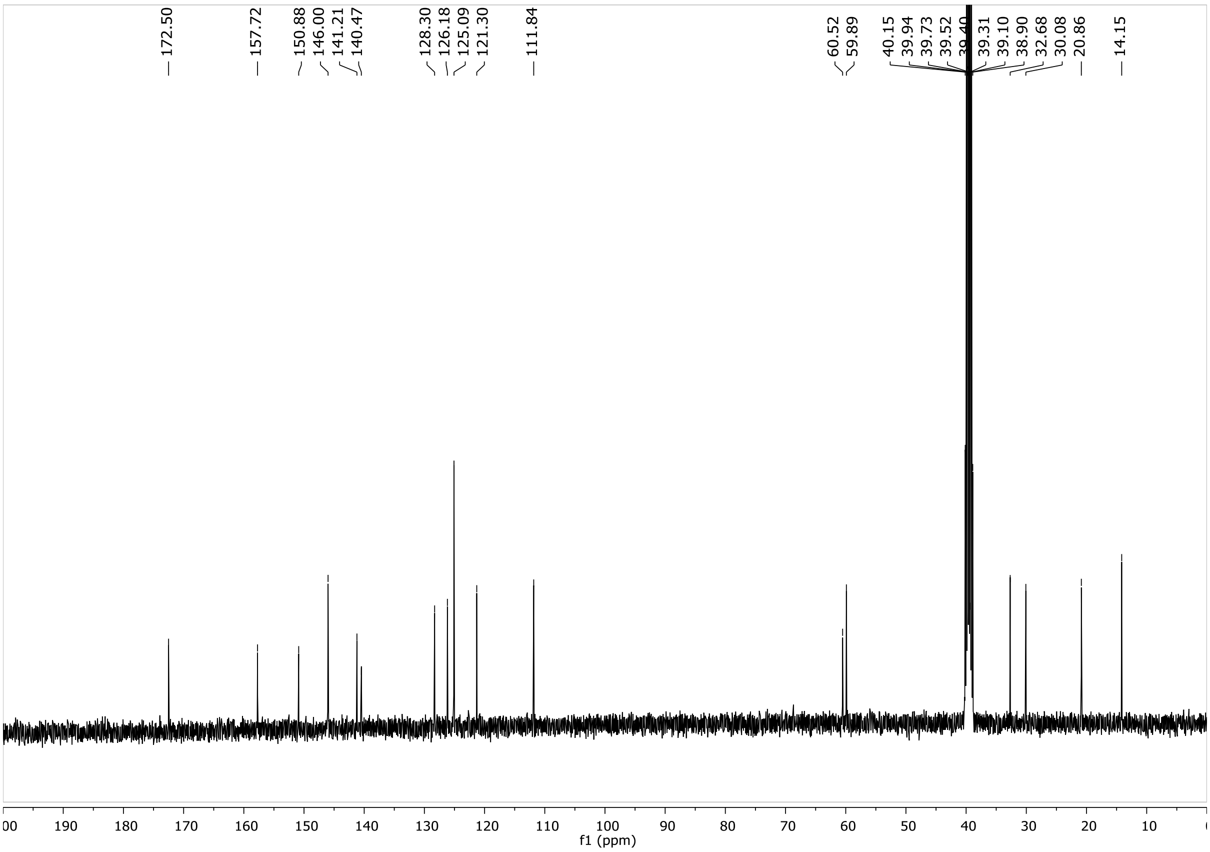
f1 (ppm)

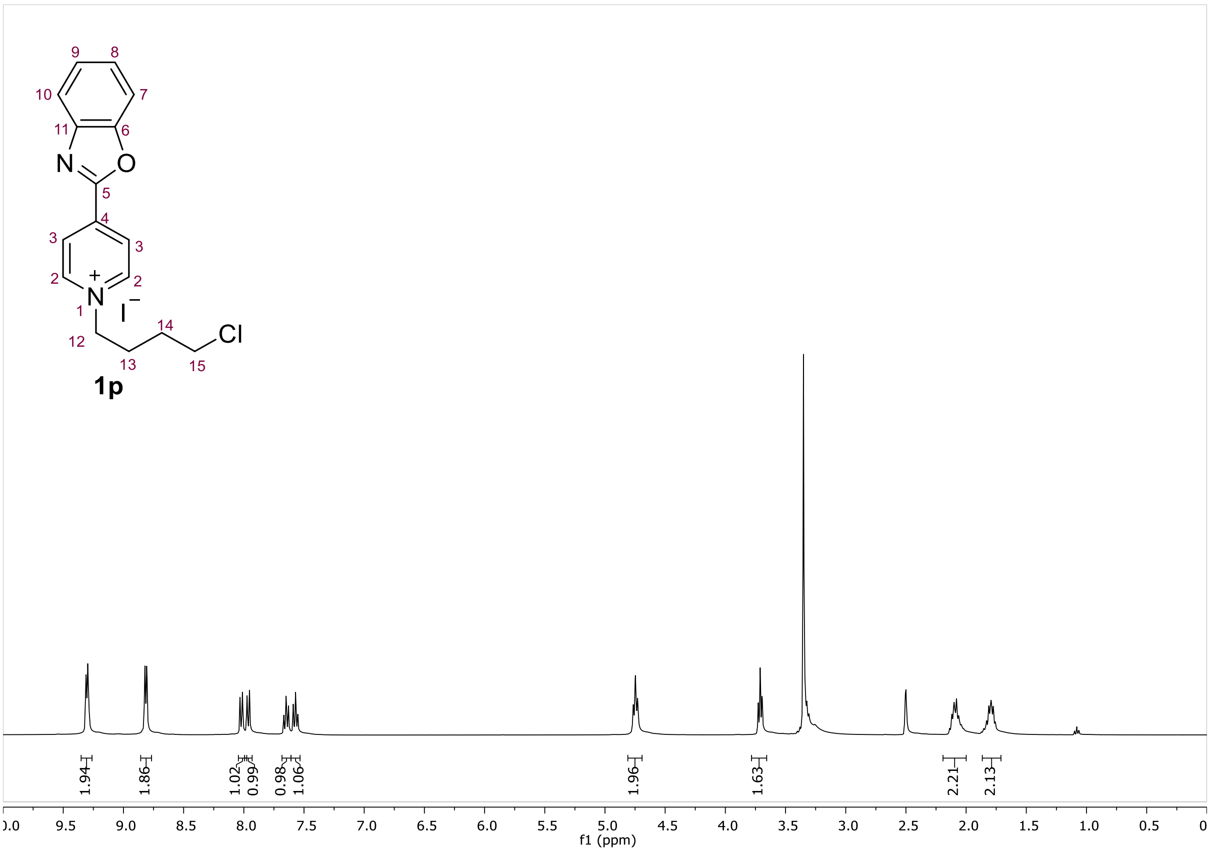

9
8
10
7
11
6
N
O
5
4
3
3
2
2
N
+
I
−
1
12
14
Cl
13
15
1p
1.94
1.86
1.02
0.99
0.98
1.06
1.96
1.63
2.21
2.13
9.5 9.0 8.5 8.0 7.5 7.0 6.5 6.0 5.5 5.0 4.5 4.0 3.5 3.0 2.5 2.0 1.5 1.0 0.5 0
f1 (ppm)

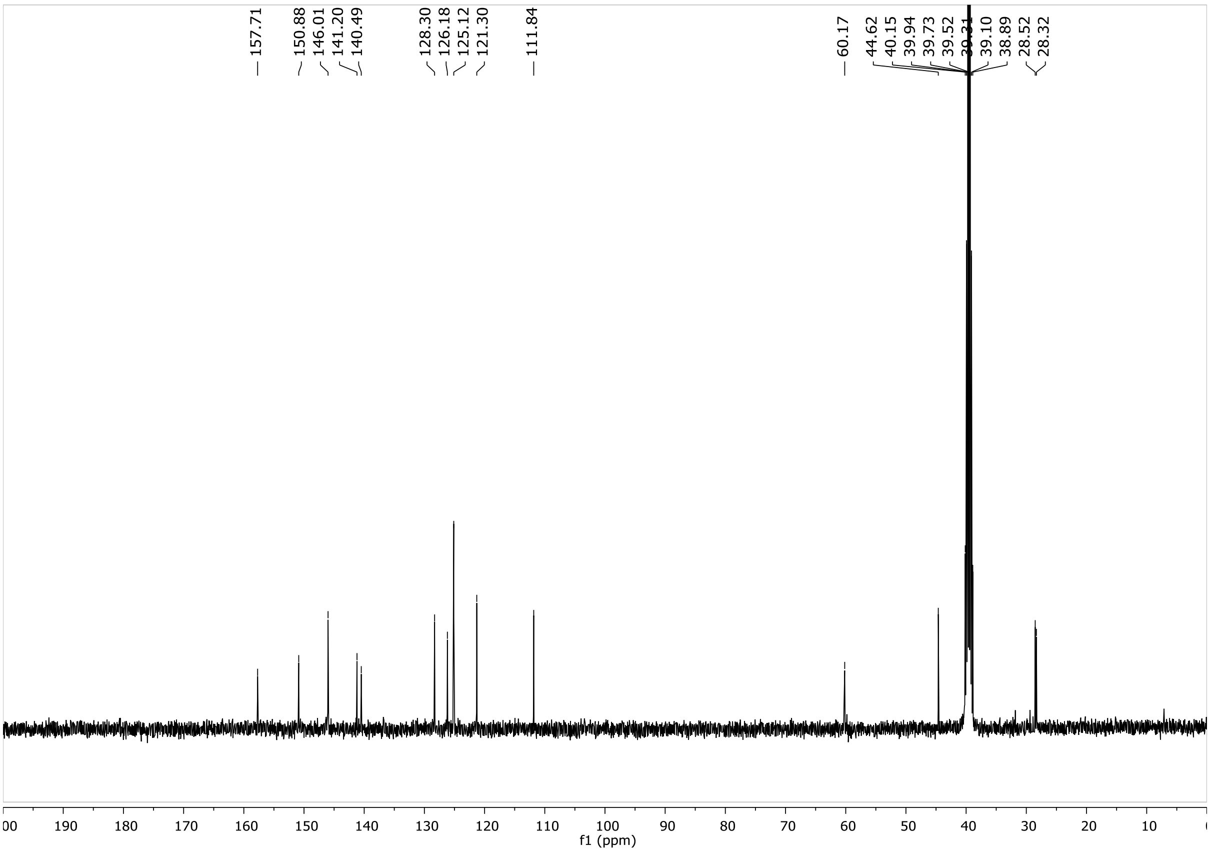

157.71
150.88
146.01
141.20
140.49
128.30
126.18
125.12
121.30
111.84
60.17
44.62
40.15
39.94
39.73
39.52
39.31
39.10
38.89
28.52
28.32
190 180 170 160 150 140 130 120 110 100 90 80 70 60 50 40 30 20 10
f1 (ppm)

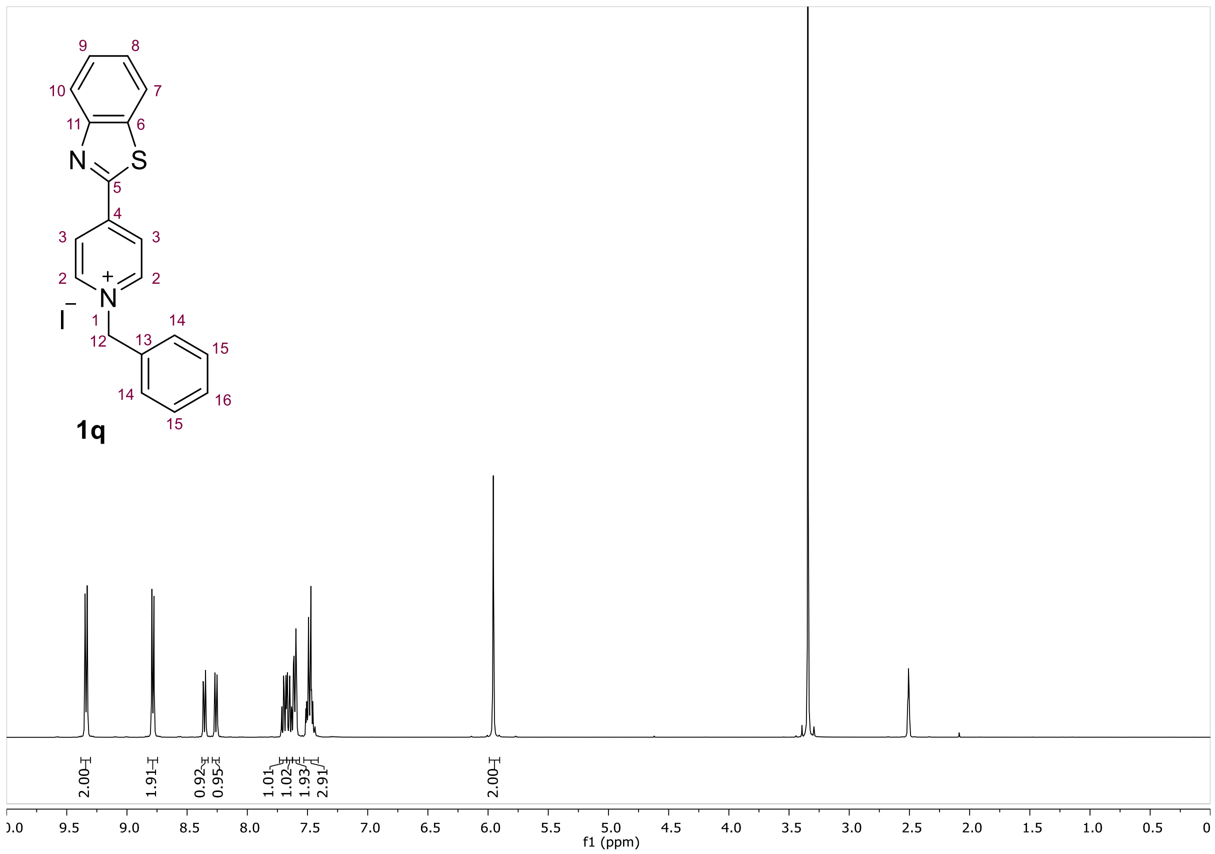
1q
f1 (ppm)

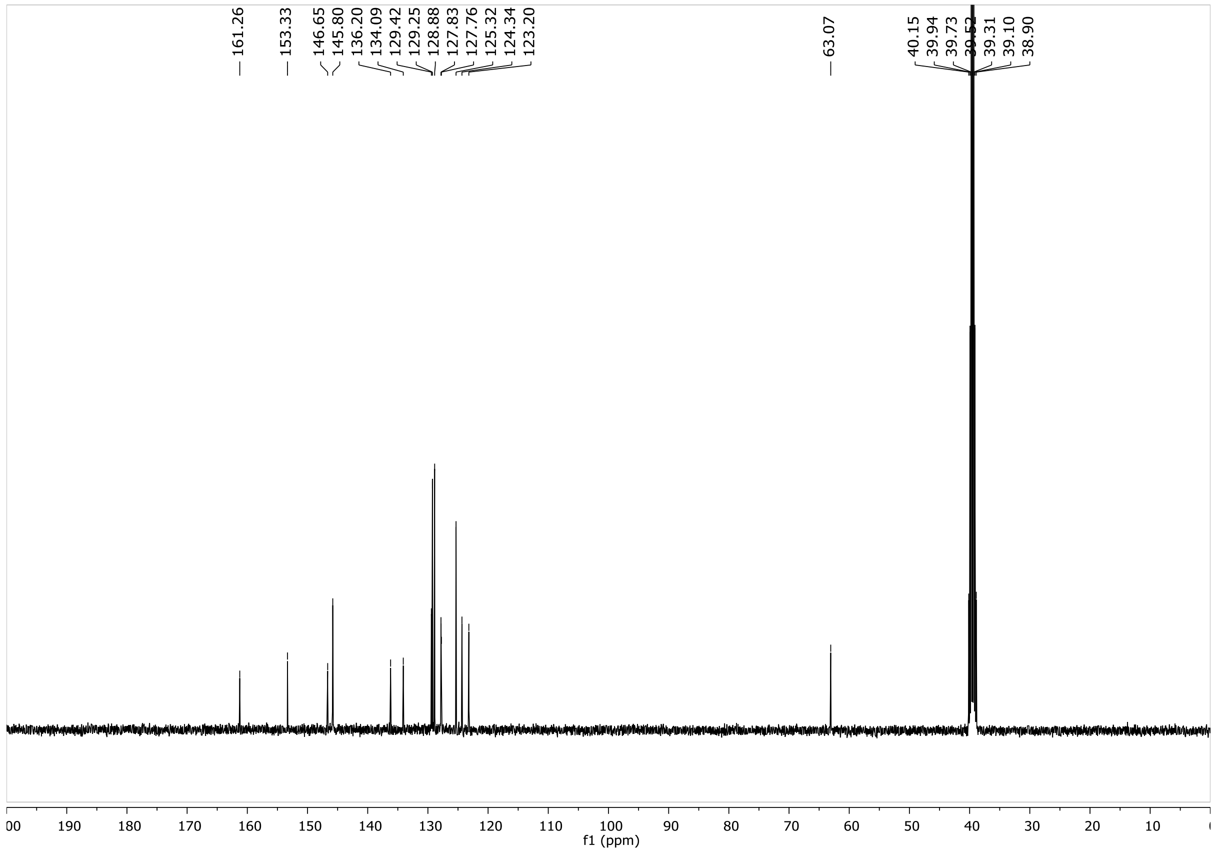
f1 (ppm)

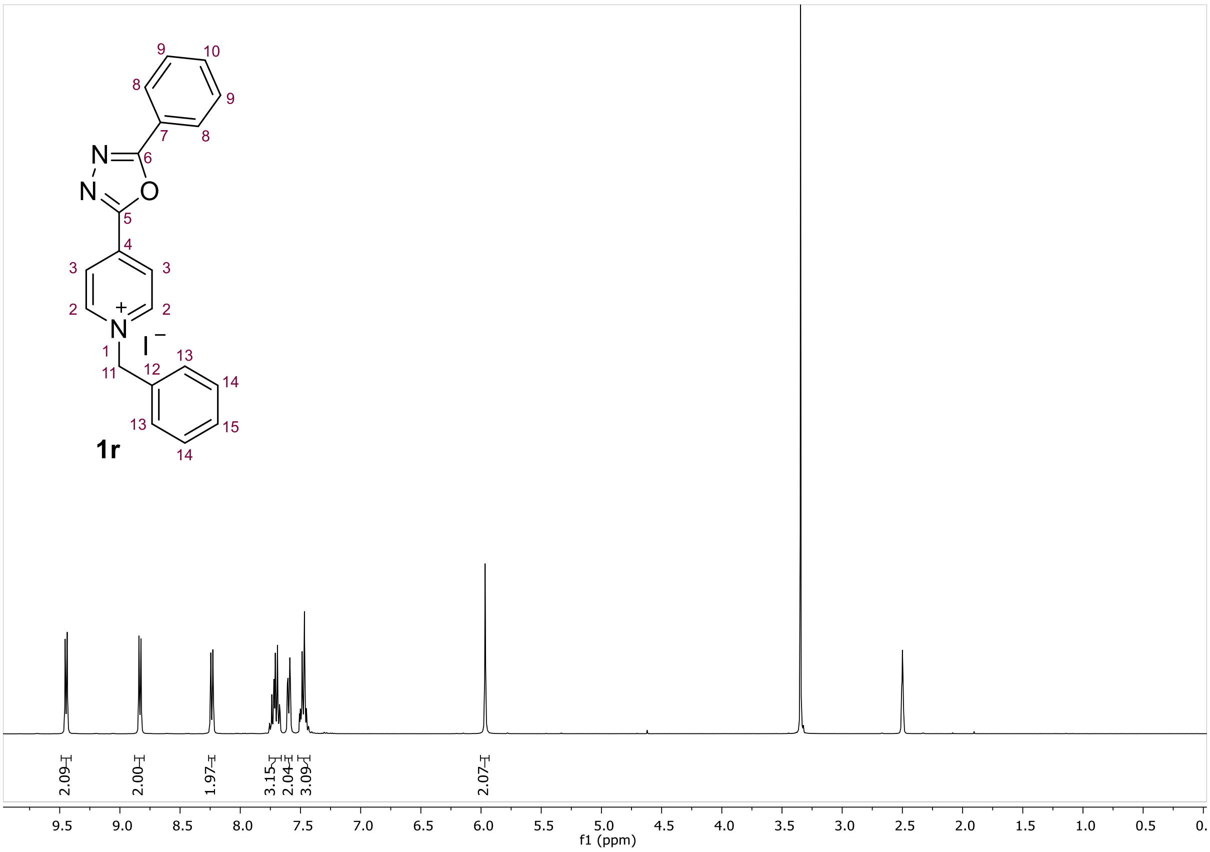

N
N
O
I⁻
1r
2.09
2.00
1.97
3.15
2.04
3.09
2.07
9.5
9.0
8.5
8.0
7.5
7.0
6.5
6.0
5.5
5.0
4.5
4.0
3.5
3.0
2.5
2.0
1.5
1.0
0.5
f1 (ppm)

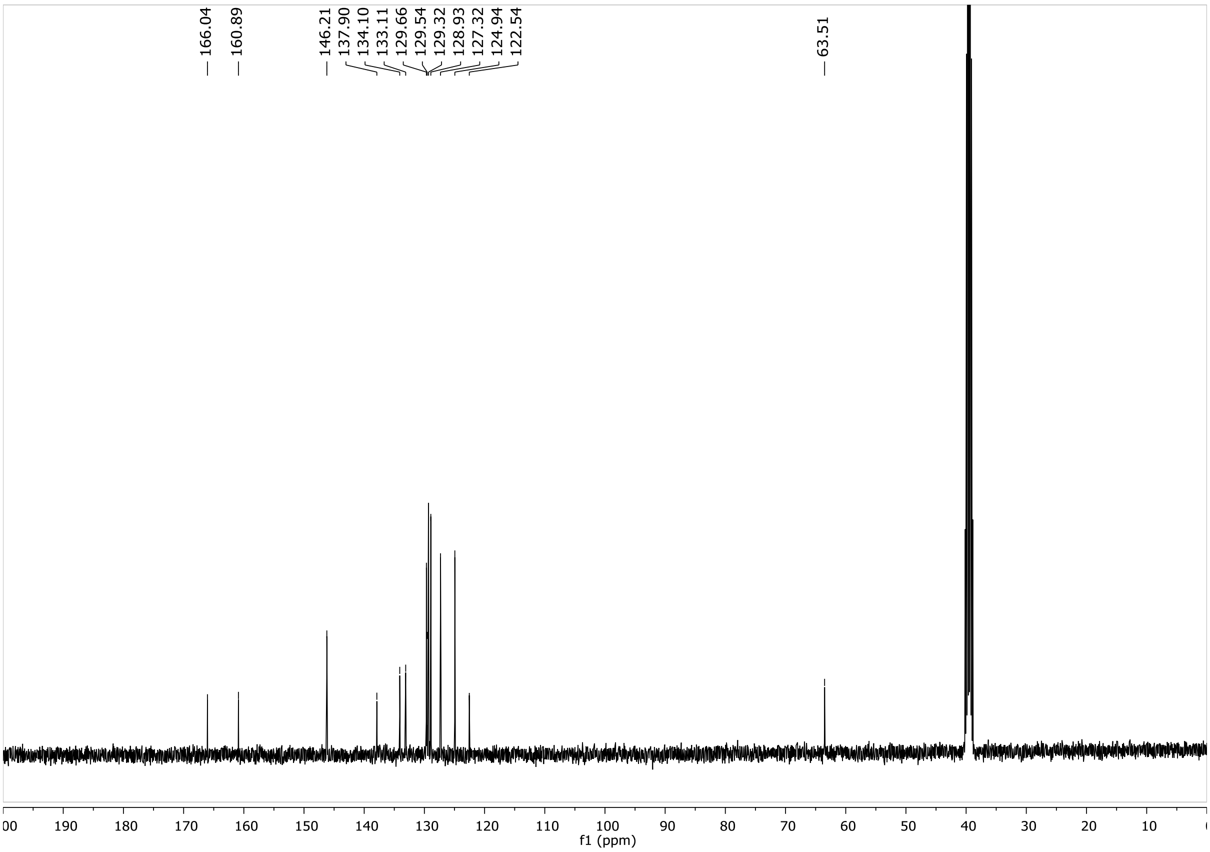

166.04
160.89
146.21
137.90
134.10
133.11
129.66
129.54
129.32
128.93
127.32
124.94
122.54
63.51
190
180
170
160
150
140
130
120
110
100
90
80
70
60
50
40
30
20
10
f1 (ppm)

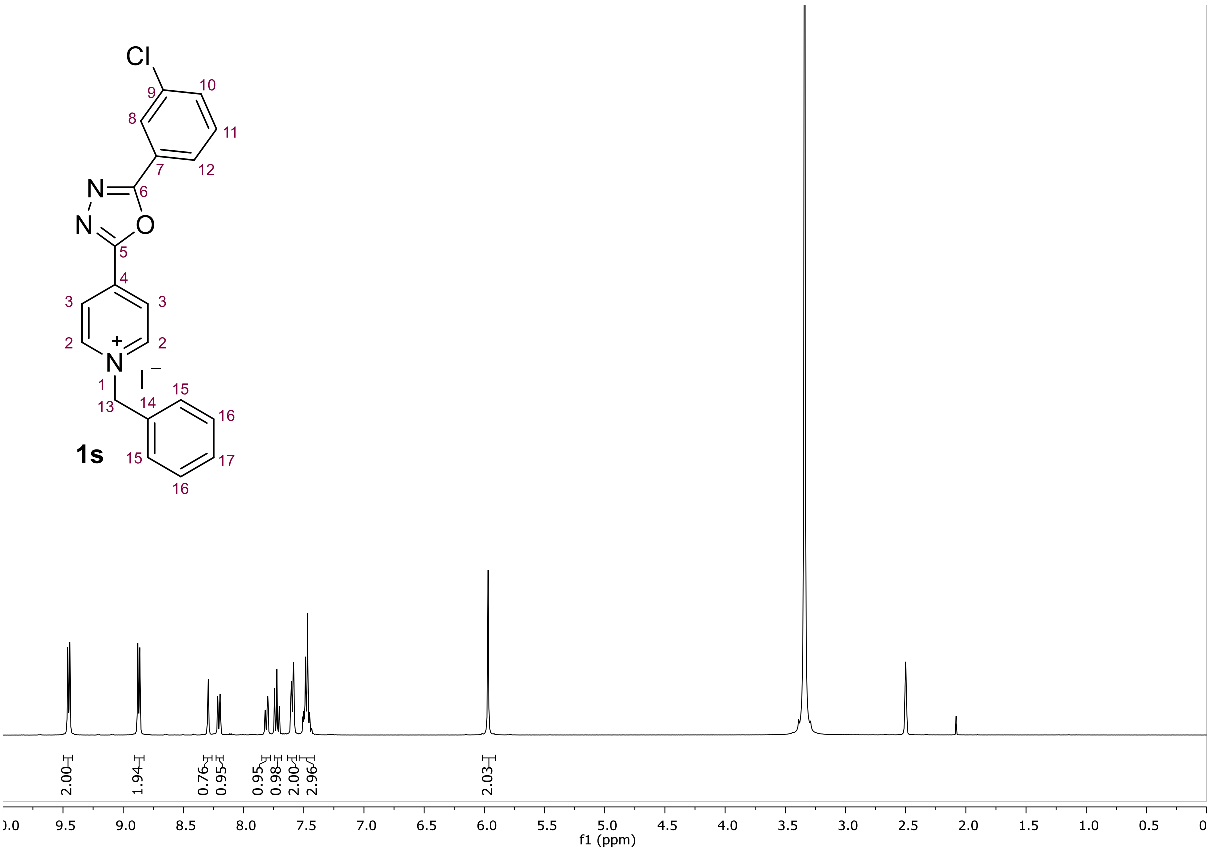

Cl
N
N
O
N+
I−
1s
2.00
1.94
0.76
0.95
0.95
0.98
2.00
2.96
2.03
f1 (ppm)

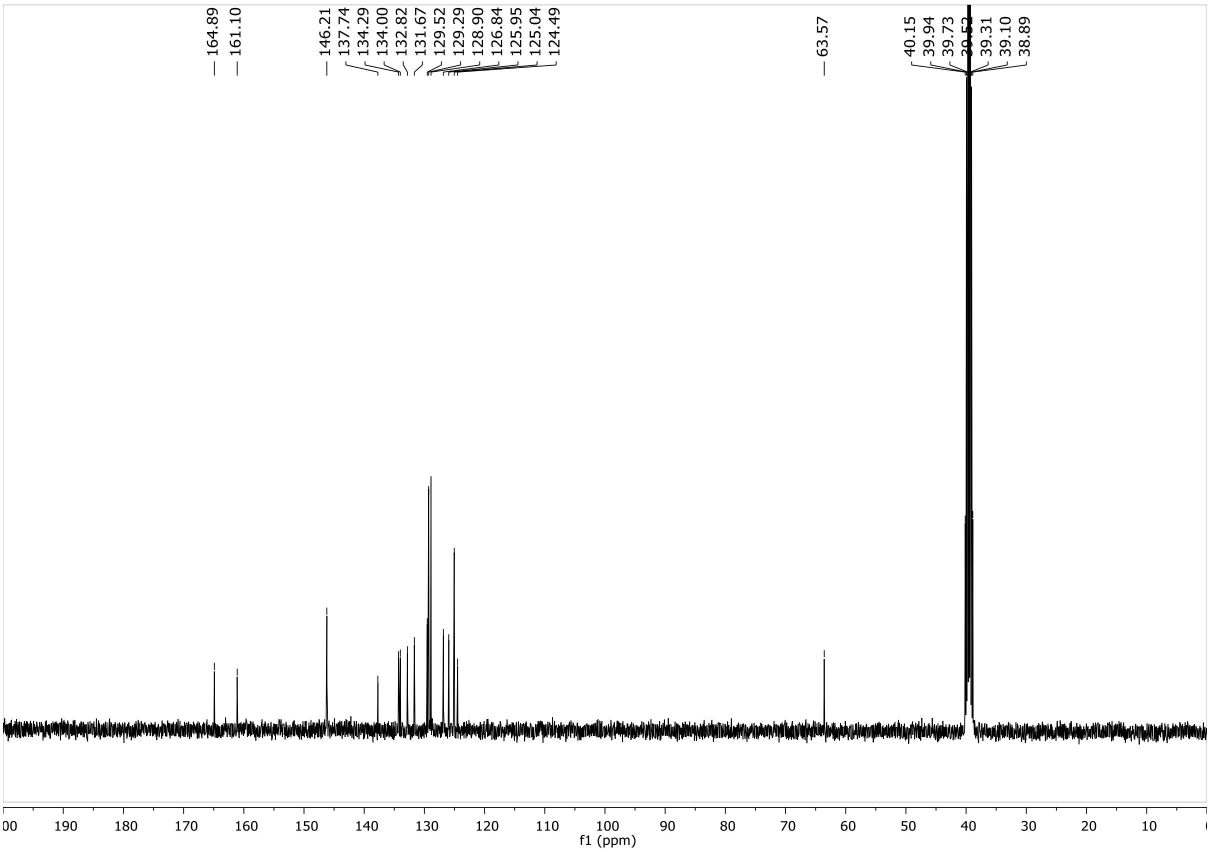

164.89
161.10
146.21
137.74
134.29
134.00
132.82
131.67
129.52
129.29
128.90
126.84
125.95
125.04
124.49
63.57
40.15
39.94
39.73
39.52
39.31
39.10
38.89
f1 (ppm)

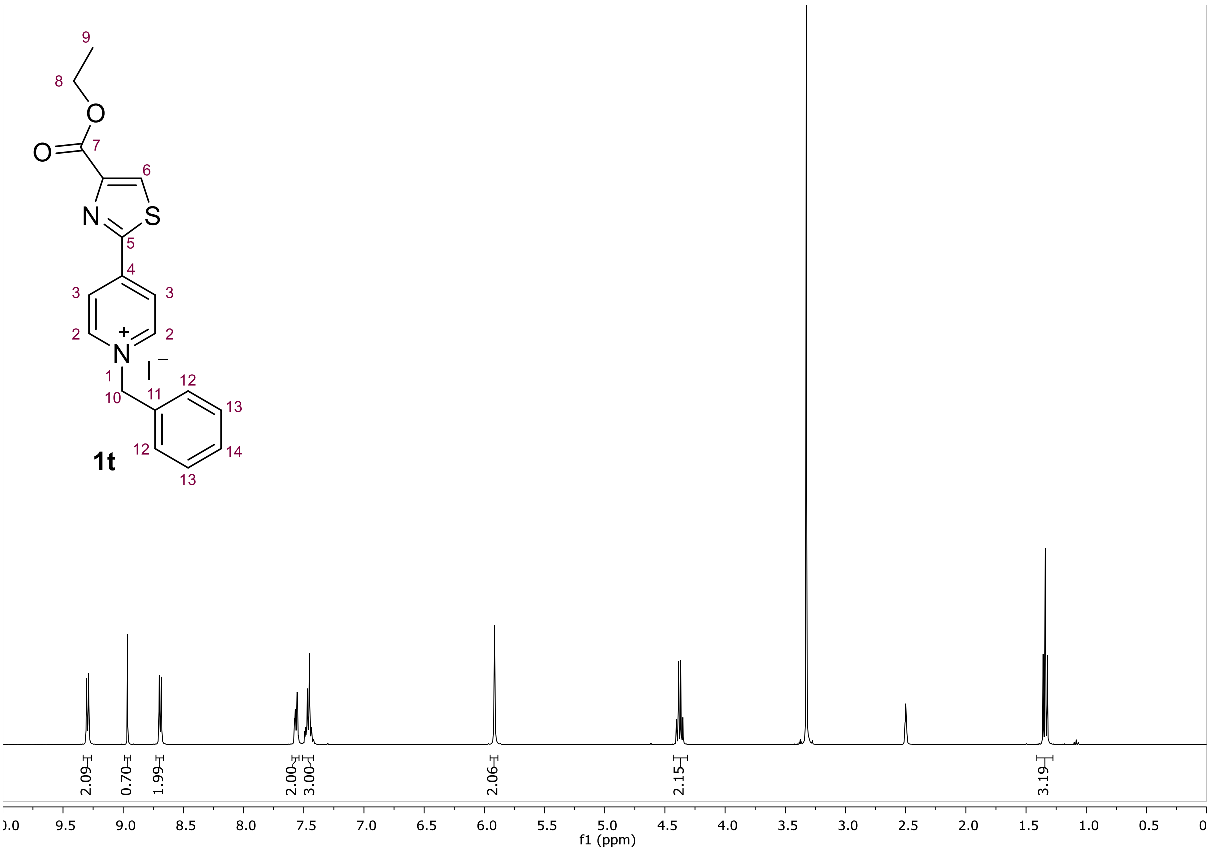
1t
2.09
0.70
1.99
2.00
3.00
2.06
2.15
3.19
f1 (ppm)

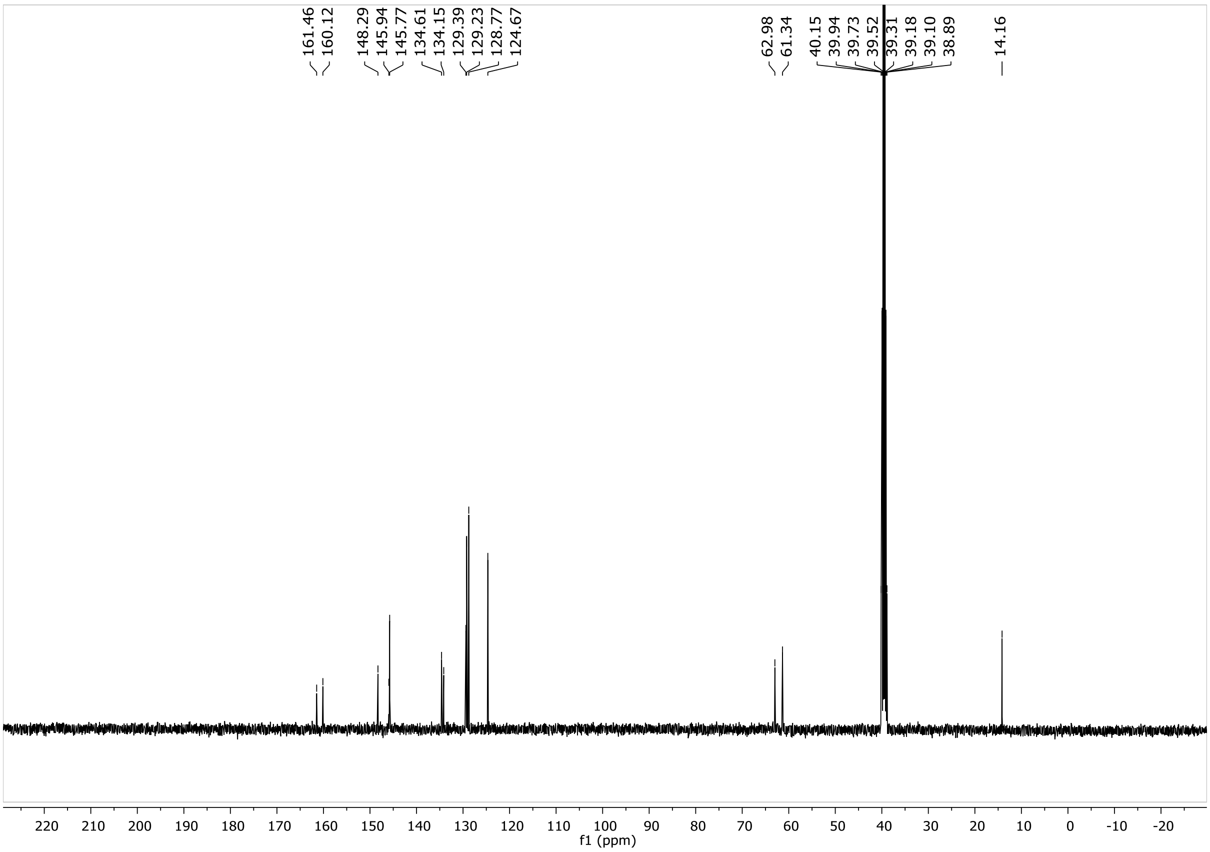
161.46
160.12
148.29
145.94
145.77
134.61
134.15
129.39
129.23
128.77
124.67
62.98
61.34
40.15
39.94
39.73
39.52
39.31
39.18
39.10
38.89
14.16
f1 (ppm)

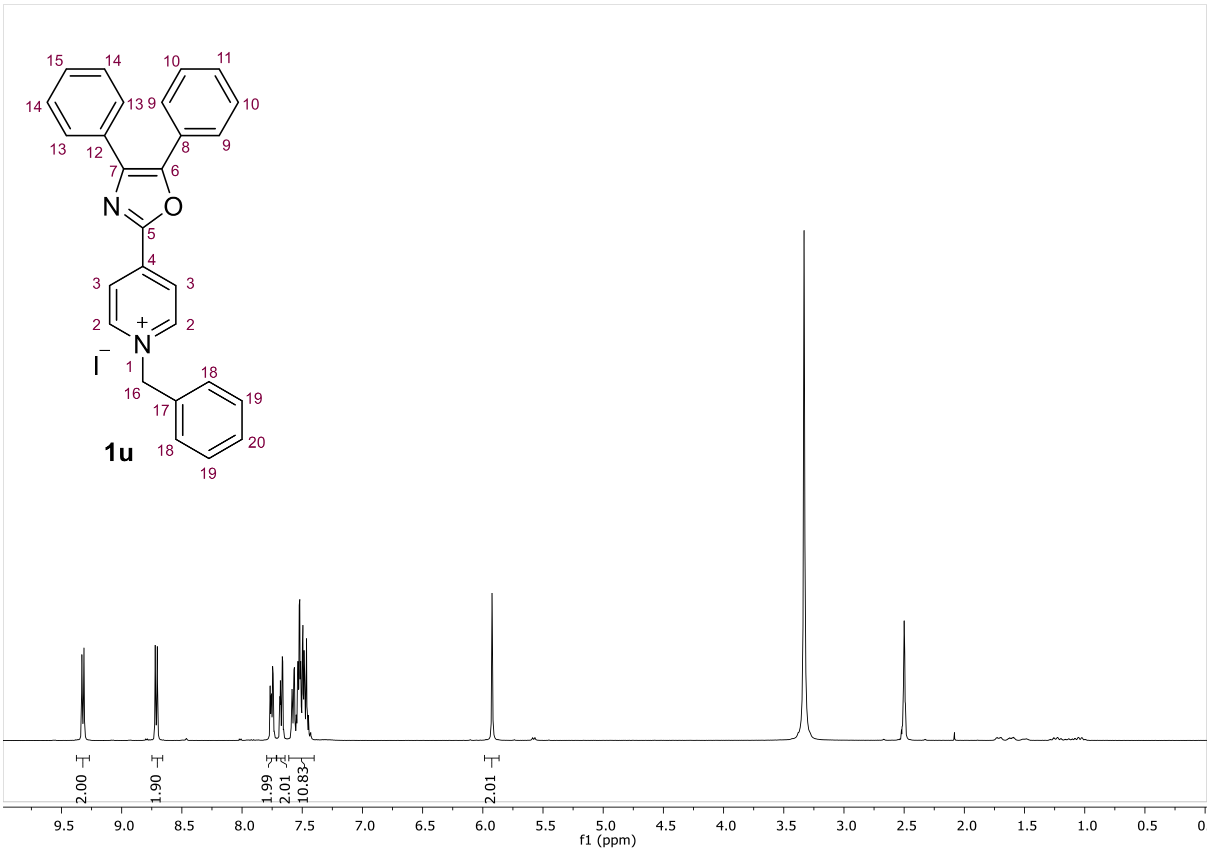
1u
2.00
1.90
1.99
2.01
10.83
2.01
9.5 9.0 8.5 8.0 7.5 7.0 6.5 6.0 5.5 5.0 4.5 4.0 3.5 3.0 2.5 2.0 1.5 1.0 0.5 0
f1 (ppm)

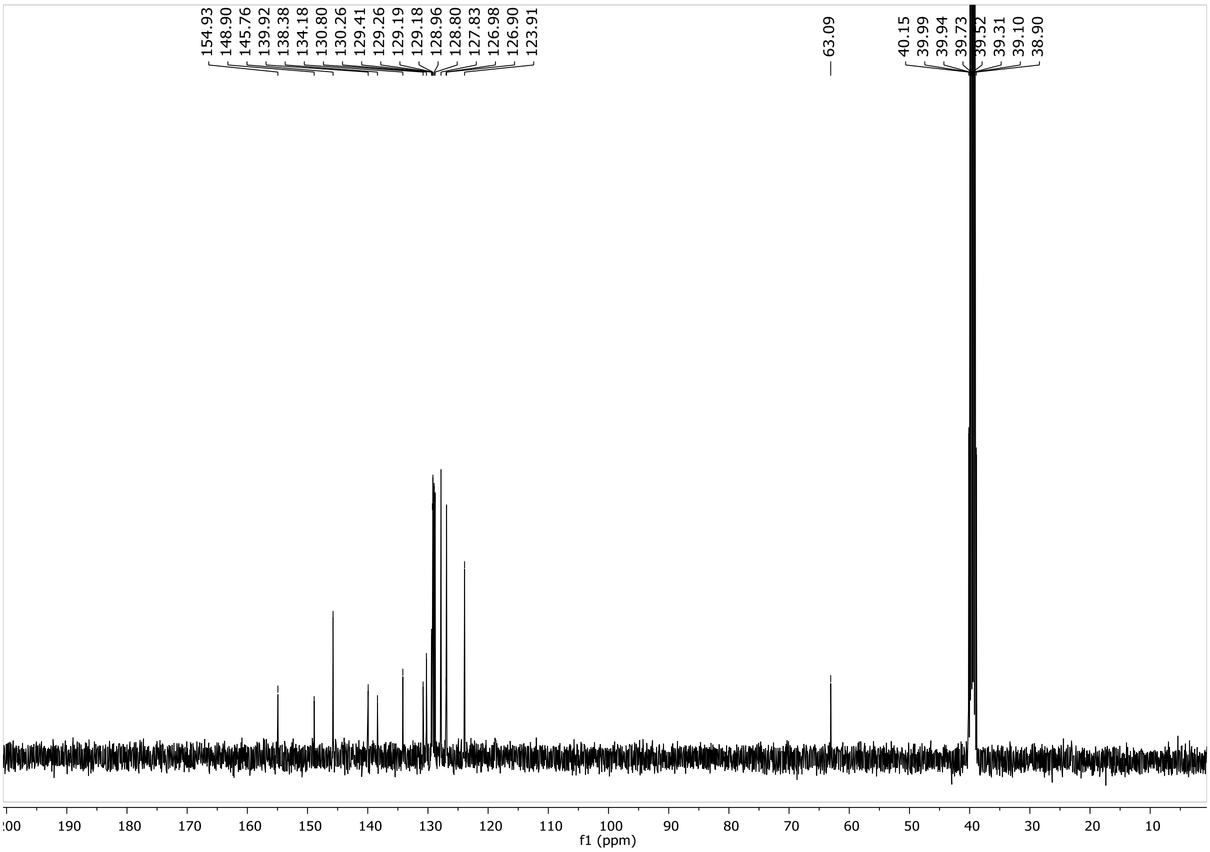
154.93
148.90
145.76
139.92
138.38
134.18
130.80
130.26
129.41
129.26
129.19
129.18
128.96
128.80
127.83
126.98
126.90
123.91
63.09
40.15
39.99
39.94
39.73
39.52
39.31
39.10
38.90
190 180 170 160 150 140 130 120 110 100 90 80 70 60 50 40 30 20 10
f1 (ppm)

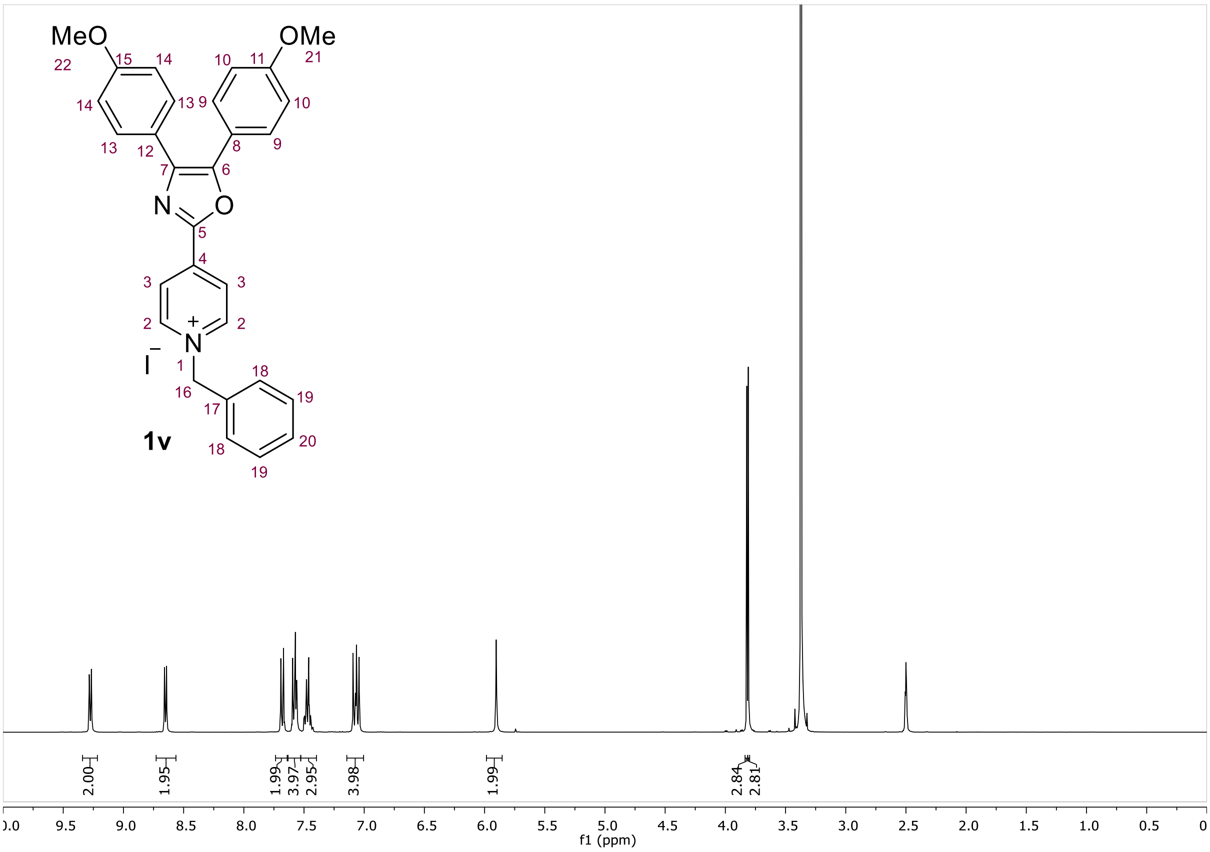

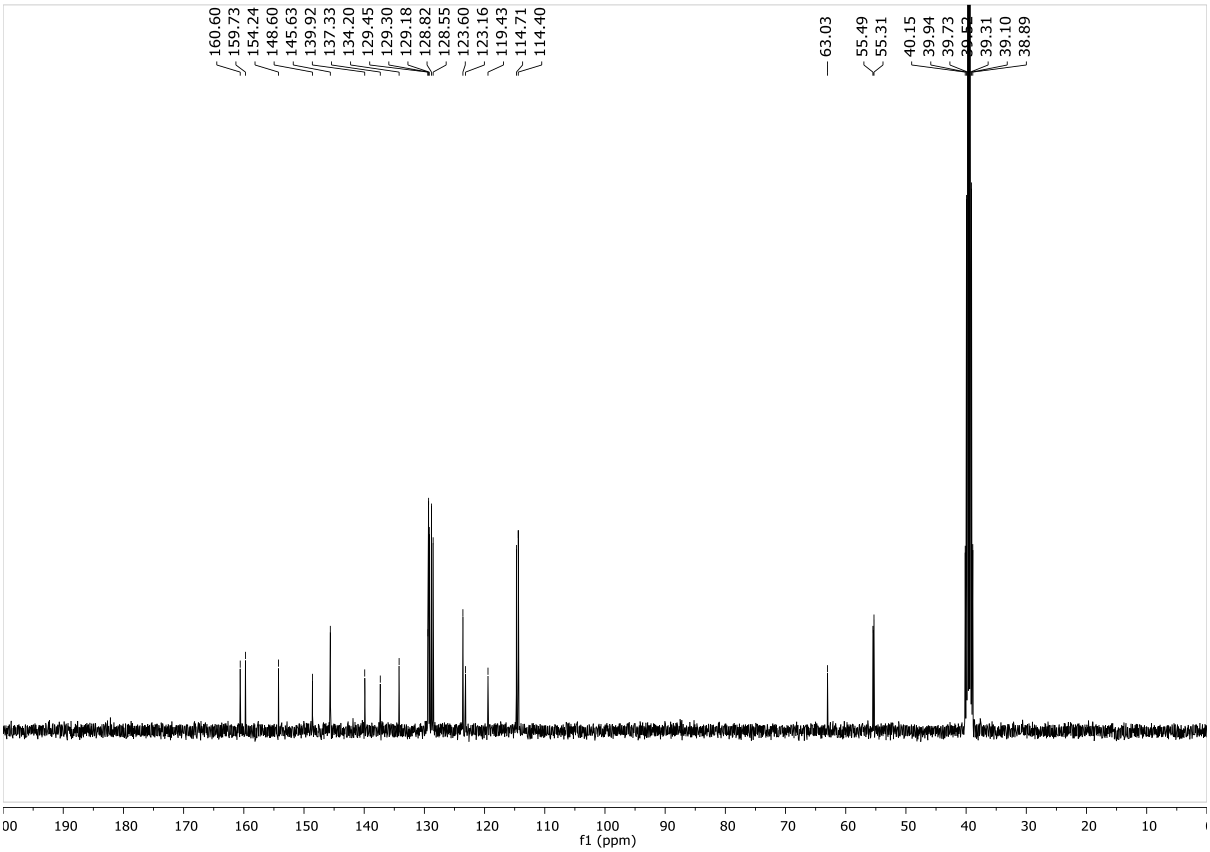

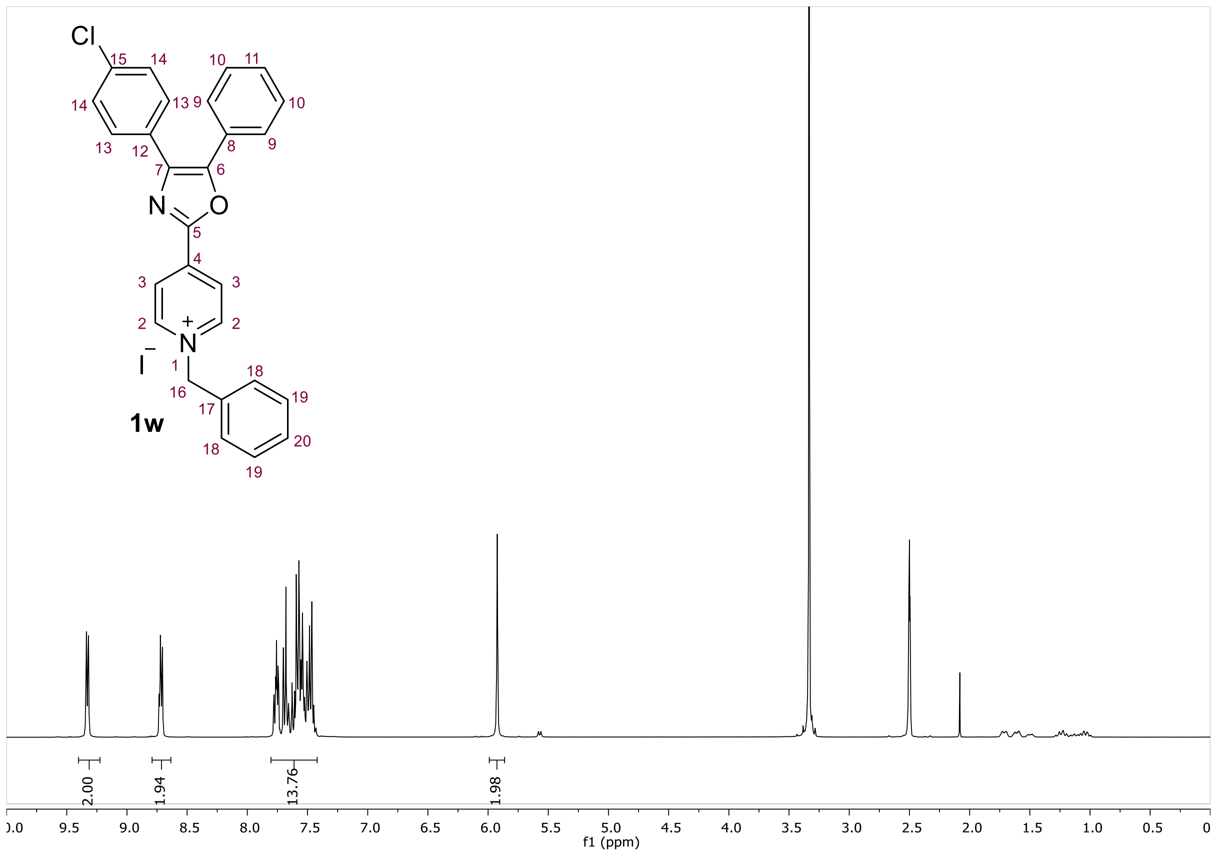
Cl
1w
2.00
1.94
13.76
1.98
f1 (ppm)

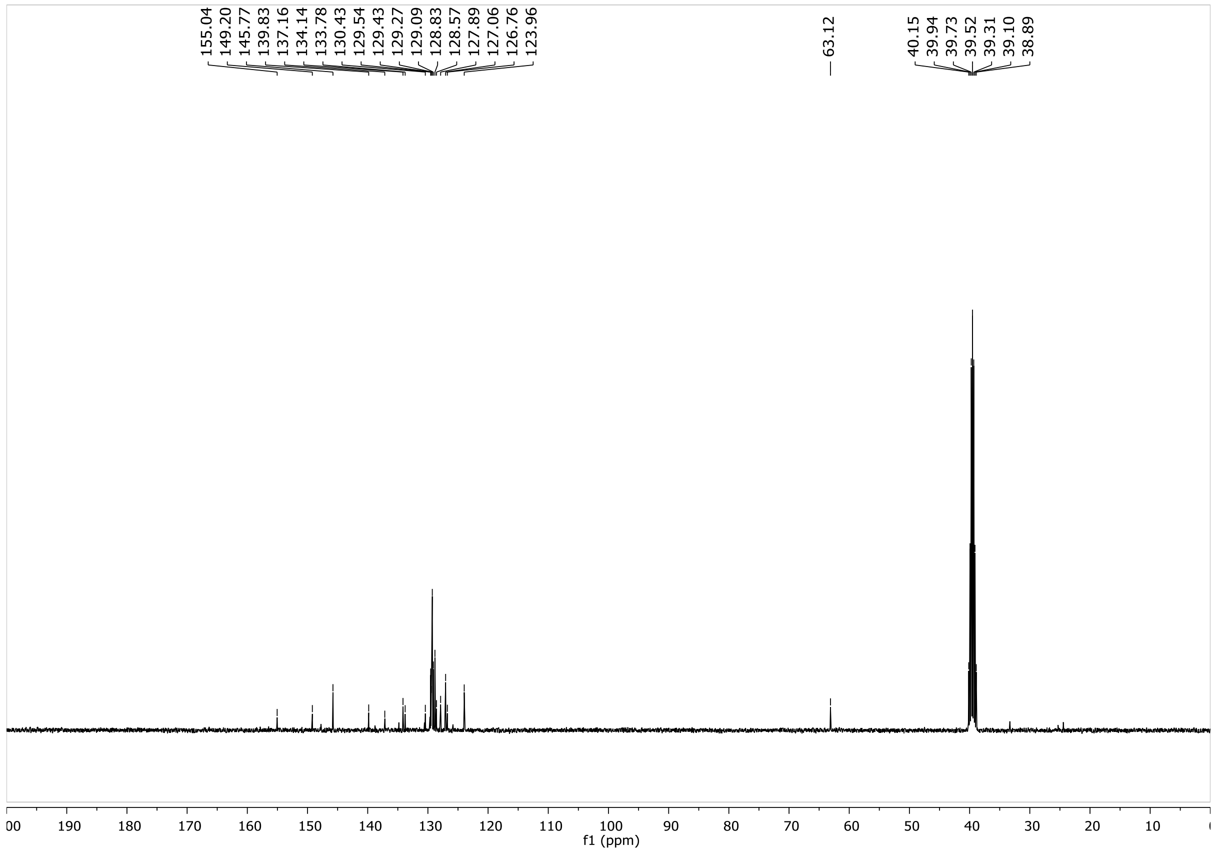
155.04
149.20
145.77
139.83
137.16
134.14
133.78
130.43
129.54
129.43
129.27
129.09
128.83
128.57
127.89
127.06
126.76
123.96
63.12
40.15
39.94
39.73
39.52
39.31
39.10
38.89
f1 (ppm)

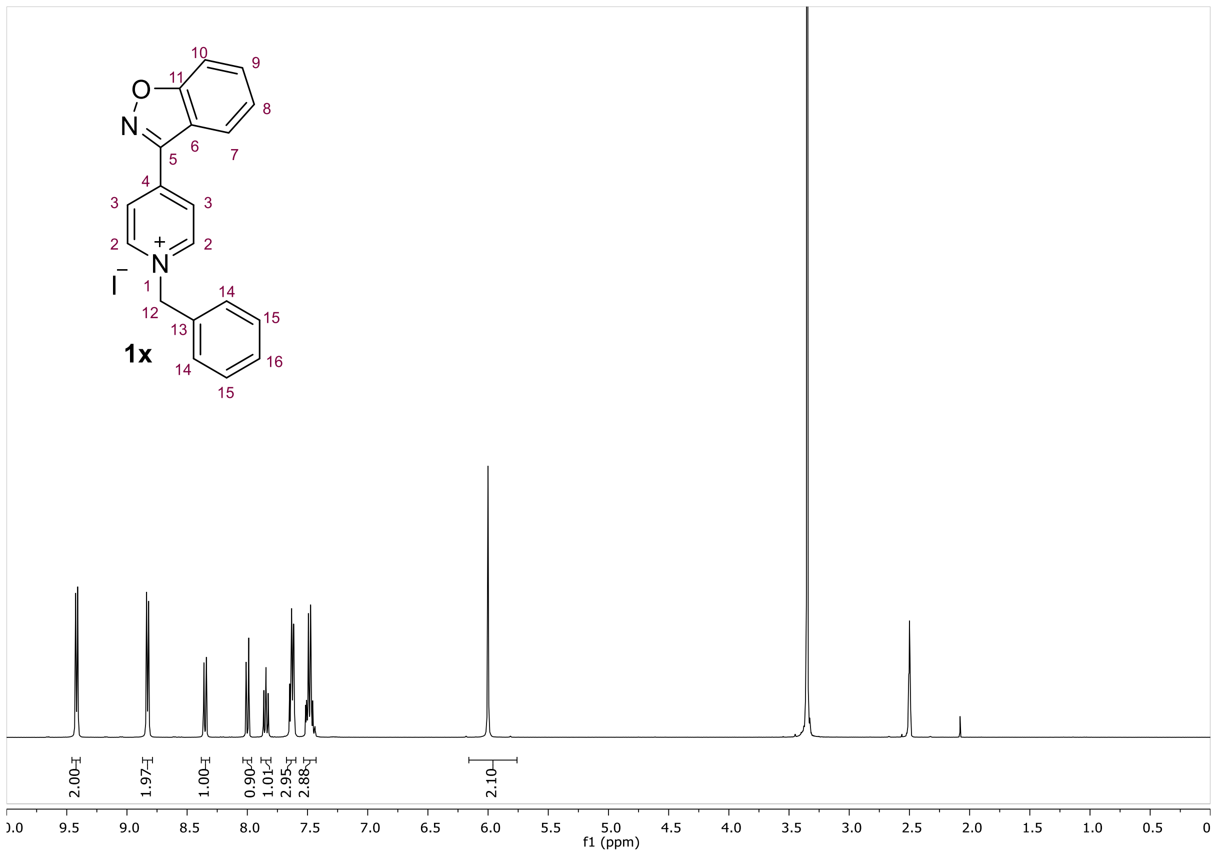

1x
2.00
1.97
1.00
0.90
1.01
2.95
2.88
2.10
f1 (ppm)

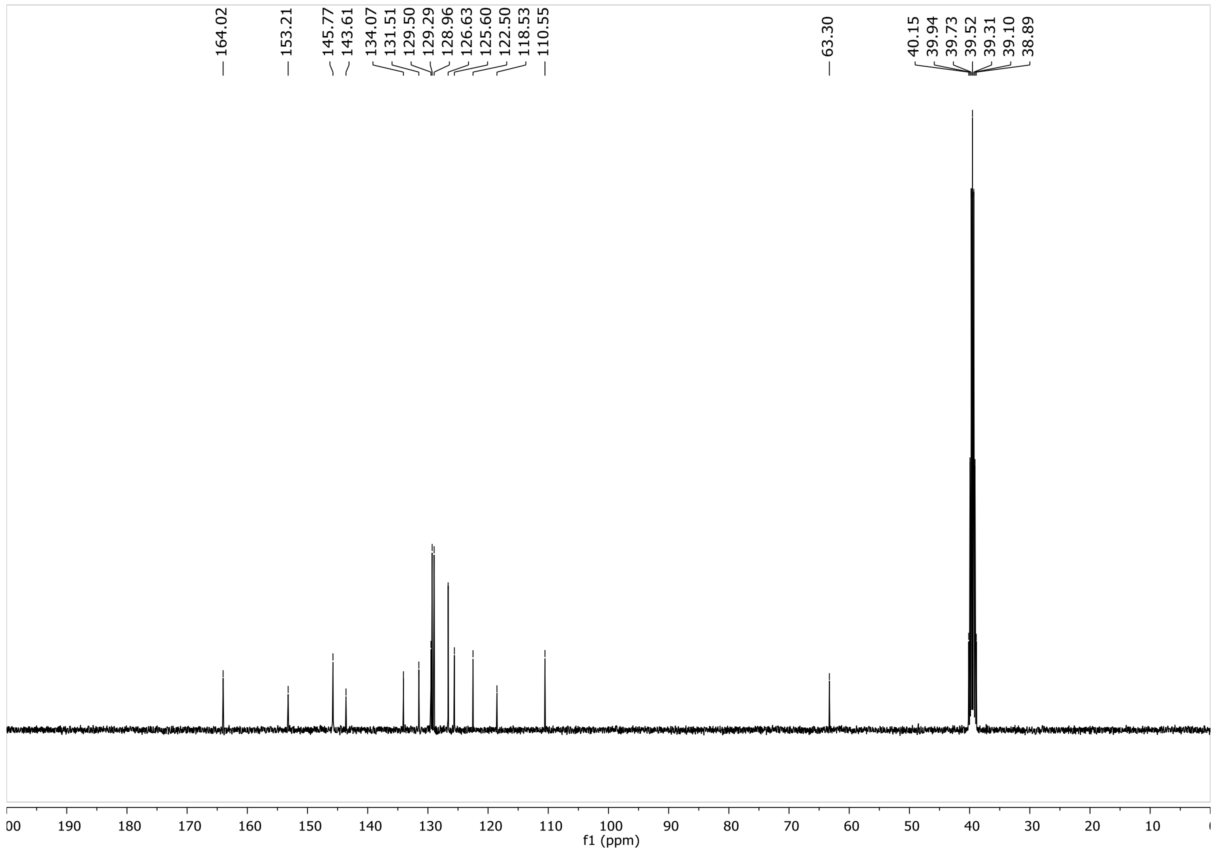

164.02
153.21
145.77
143.61
134.07
131.51
129.50
129.29
128.96
126.63
125.60
122.50
118.53
110.55
63.30
40.15
39.94
39.73
39.52
39.31
39.10
38.89
f1 (ppm)

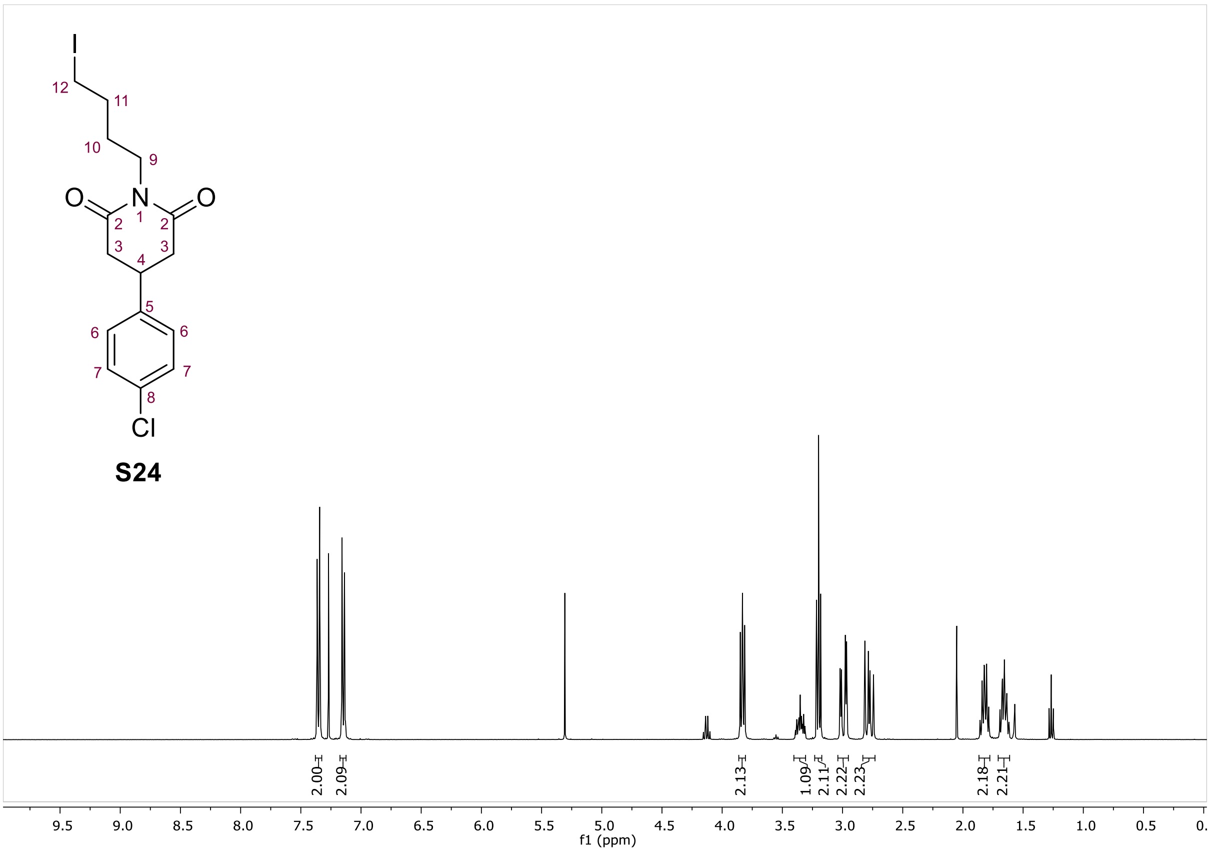
S24
I
Cl
N
O
O
2.00
2.09
2.13
1.09
2.11
2.22
2.23
2.18
2.21
f1 (ppm)

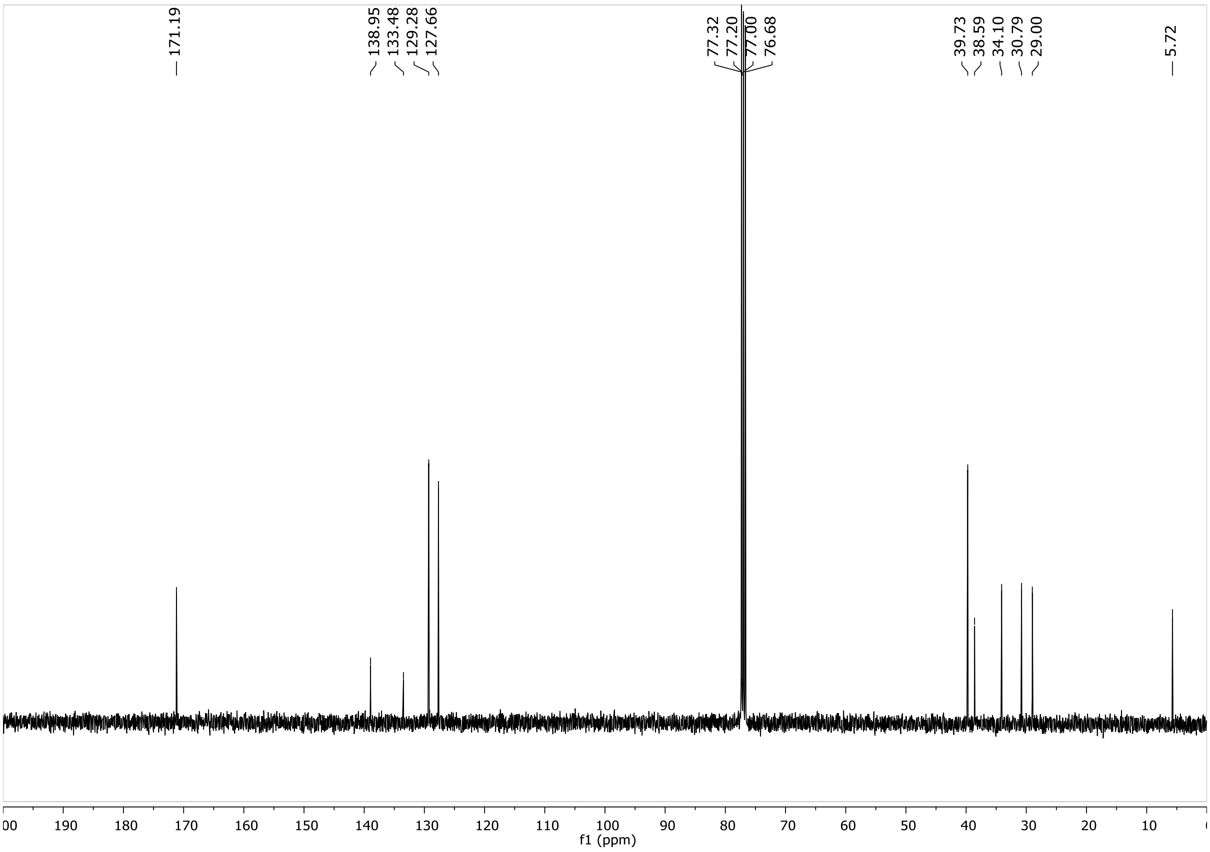
171.19
138.95
133.48
129.28
127.66
77.32
77.20
77.00
76.68
39.73
38.59
34.10
30.79
29.00
5.72
f1 (ppm)

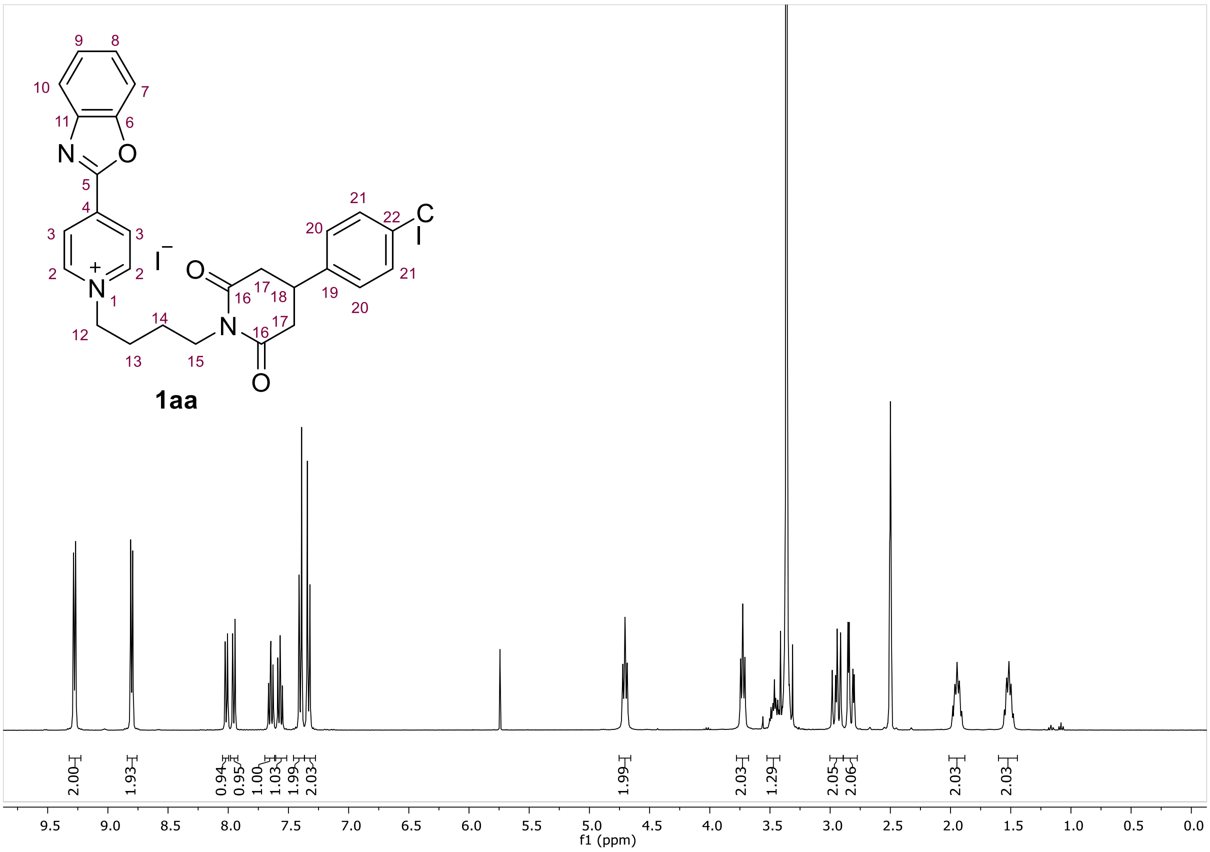
1aa
2.00
1.93
0.94
0.95
1.00
1.03
1.99
2.03
1.99
2.03
1.29
2.05
2.06
2.03
2.03
f1 (ppm)

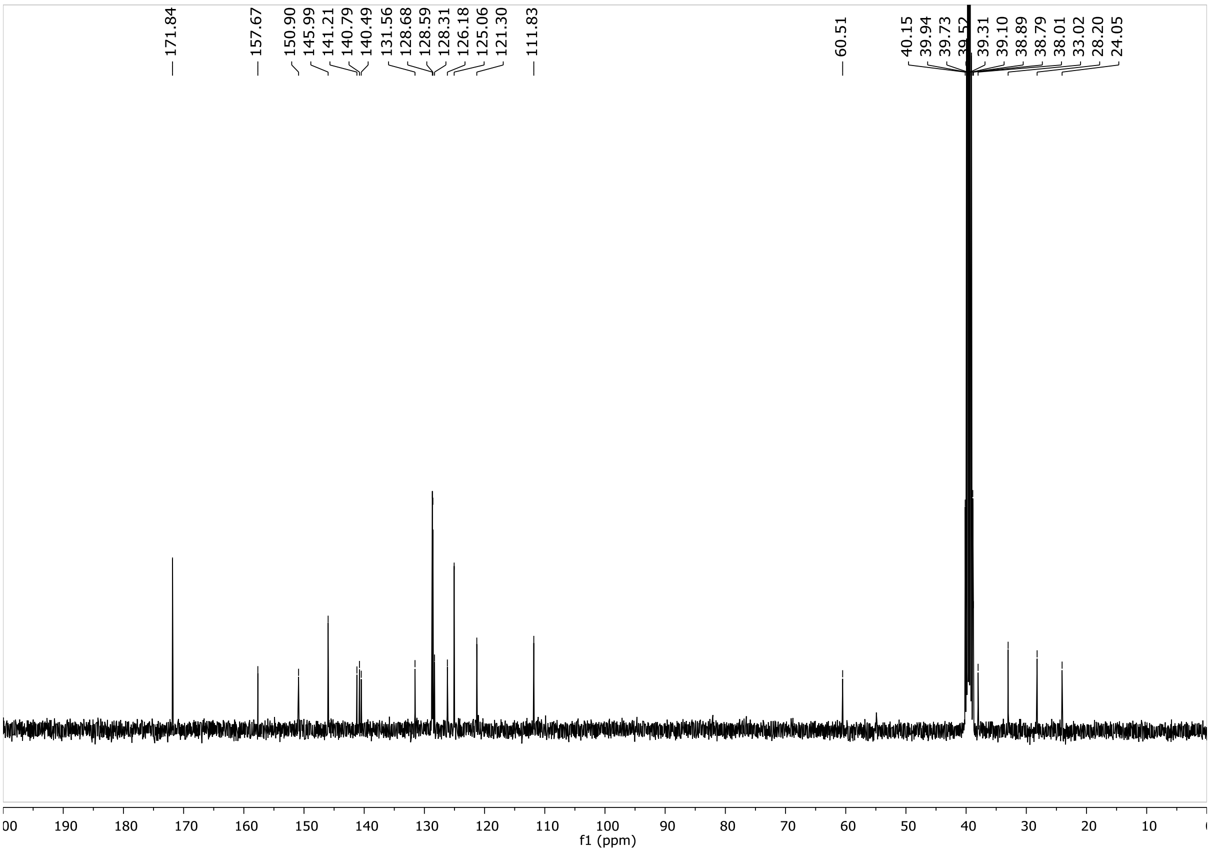
171.84
157.67
150.90
145.99
141.21
140.79
140.49
131.56
128.68
128.59
128.31
126.18
125.06
121.30
111.83
60.51
40.15
39.94
39.73
39.31
39.10
38.89
38.79
38.01
33.02
28.20
24.05
f1 (ppm)

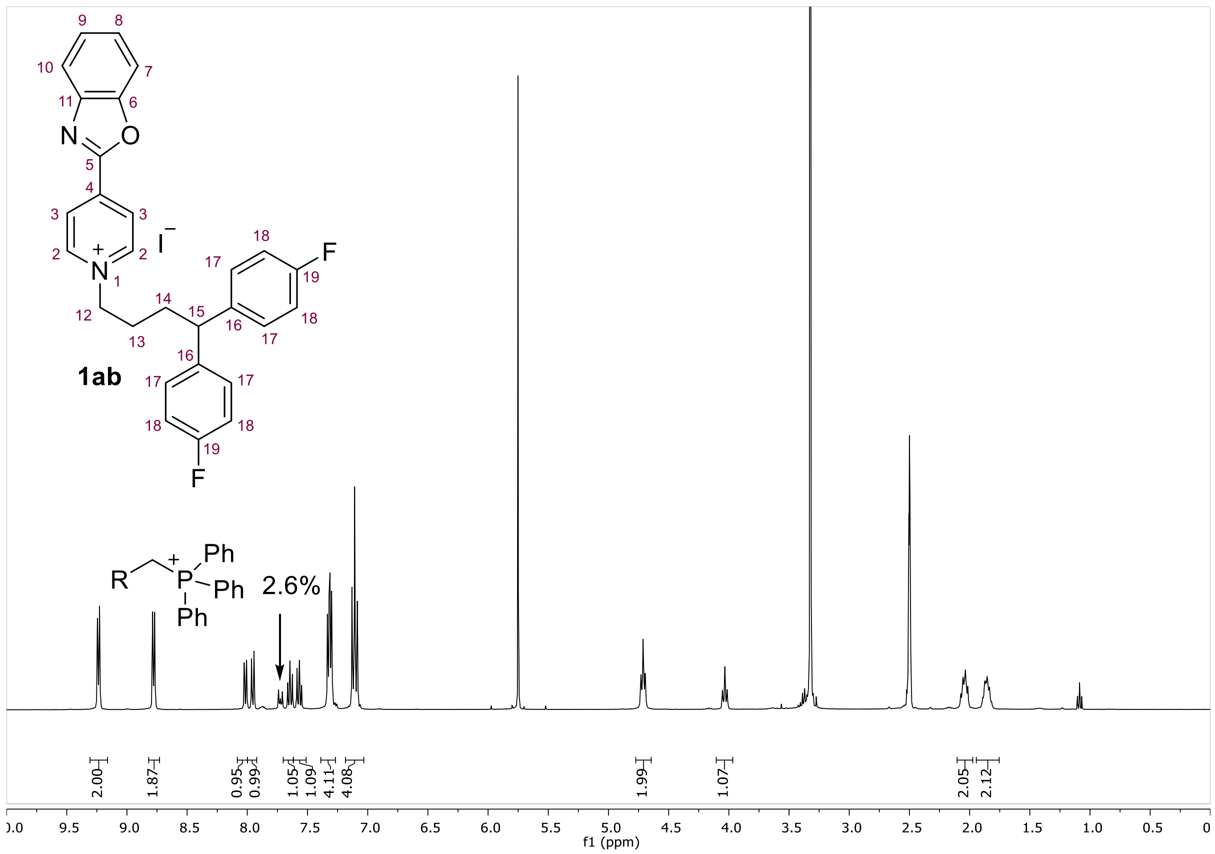

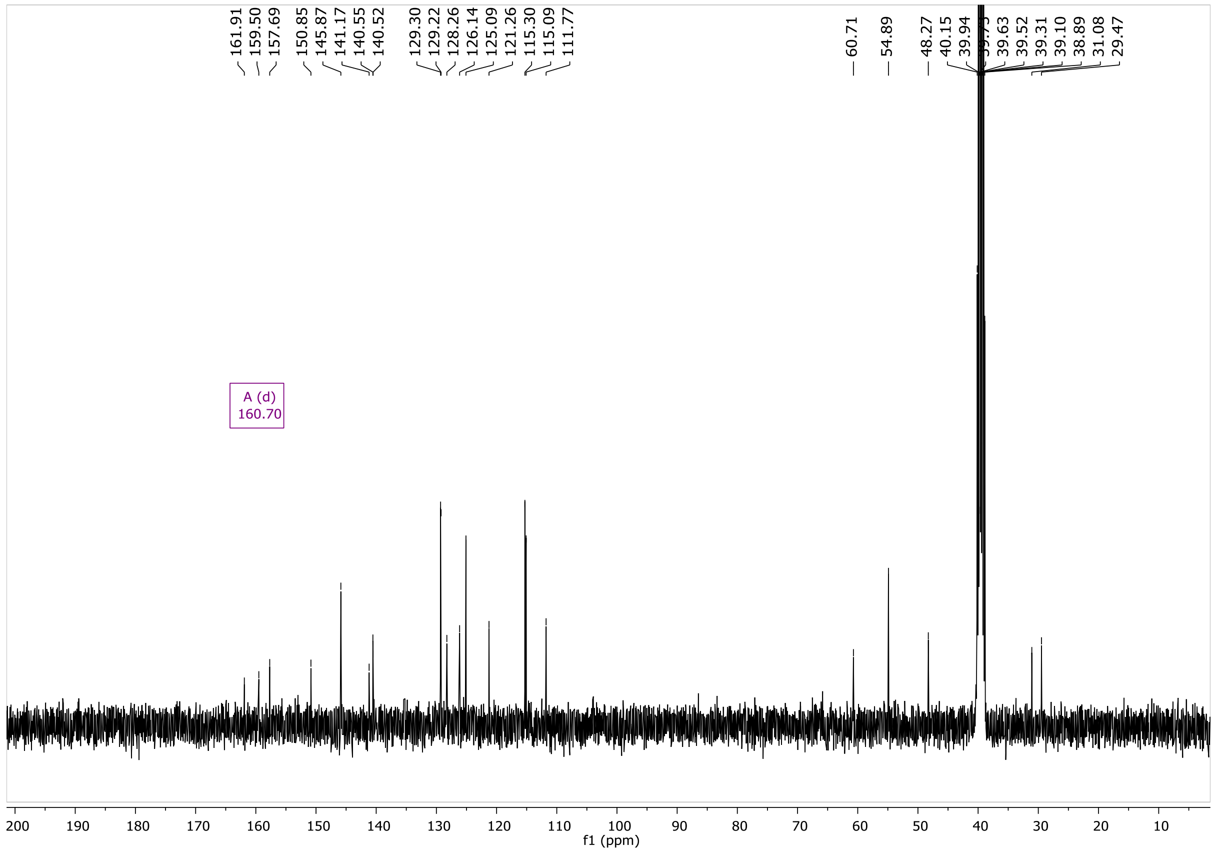

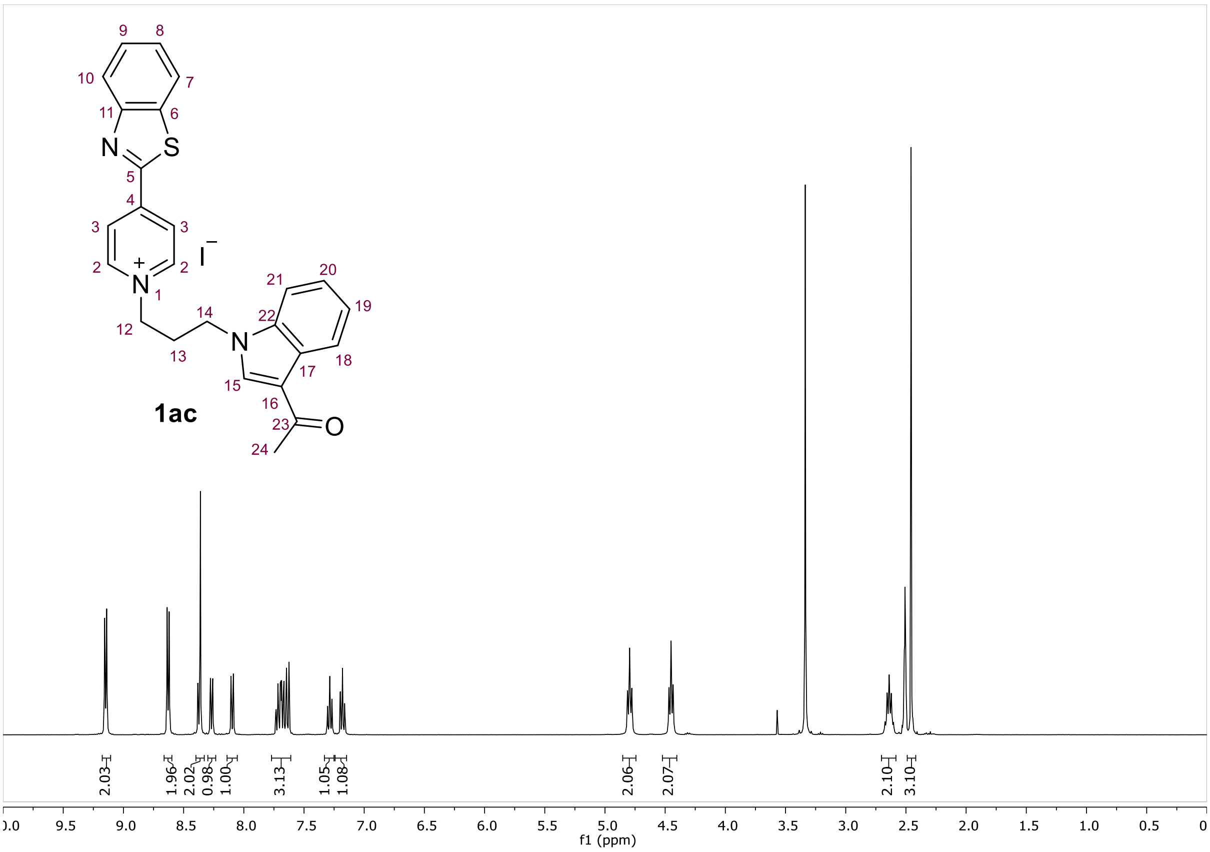

1ac
I⁻
2.03
1.96
2.02
0.98
1.00
3.13
1.05
1.08
2.06
2.07
2.10
3.10
f1 (ppm)

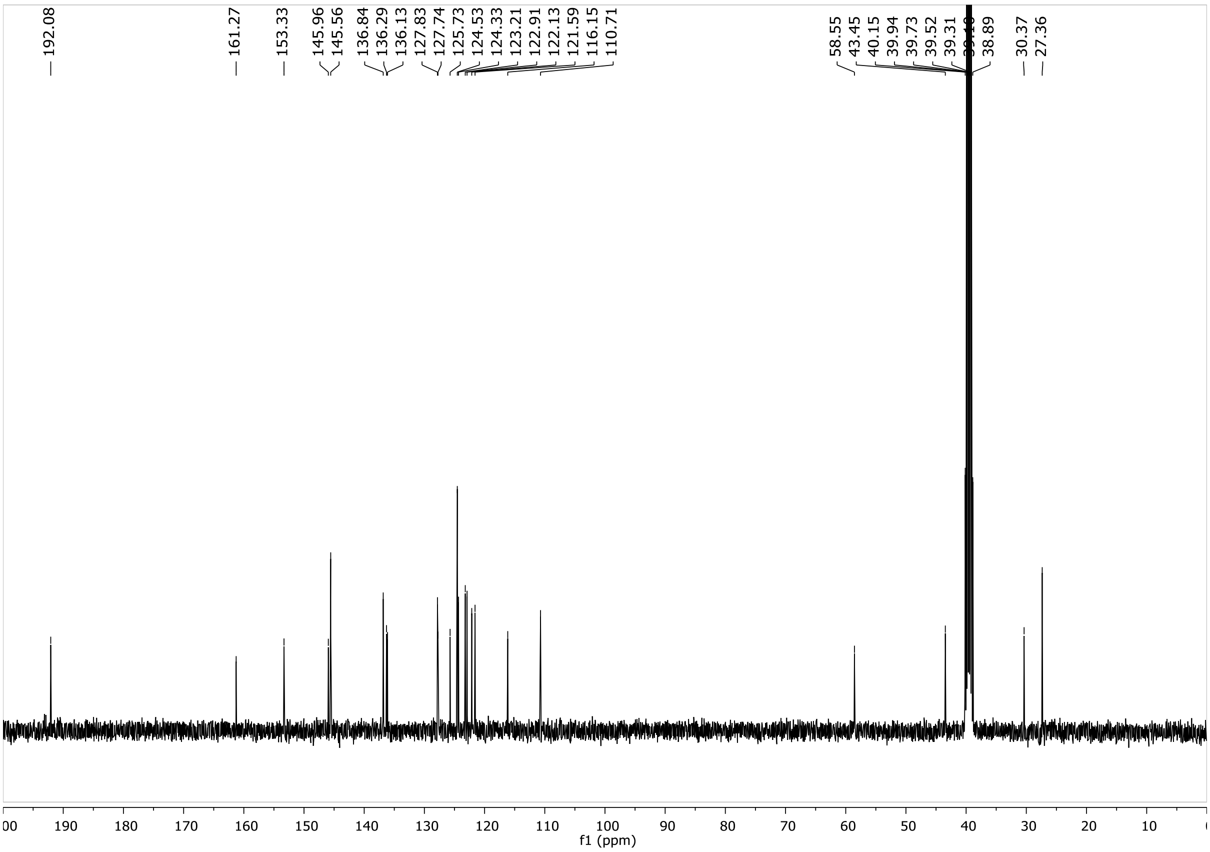

192.08
161.27
153.33
145.96
145.56
136.84
136.29
136.13
127.83
127.74
125.73
124.53
124.33
123.21
122.91
122.13
121.59
116.15
110.71
58.55
43.45
40.15
39.94
39.73
39.52
39.31
38.89
30.37
27.36
f1 (ppm)

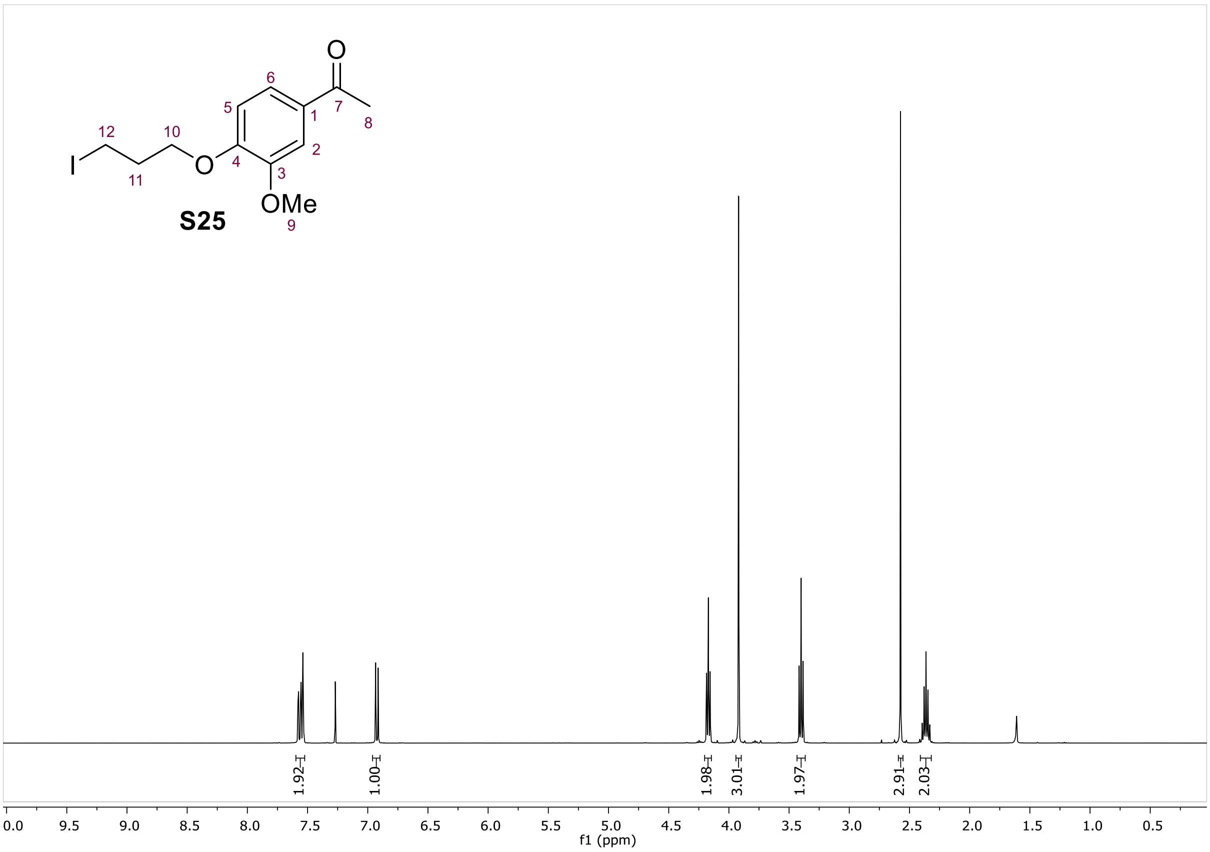
O
6
5
7
1
8
12
10
2
I
O
4
11
3
OMe
9
S25
1.92
1.00
1.98
3.01
1.97
2.91
2.03
0.0
9.5
9.0
8.5
8.0
7.5
7.0
6.5
6.0
5.5
5.0
4.5
4.0
3.5
3.0
2.5
2.0
1.5
1.0
0.5
f1 (ppm)

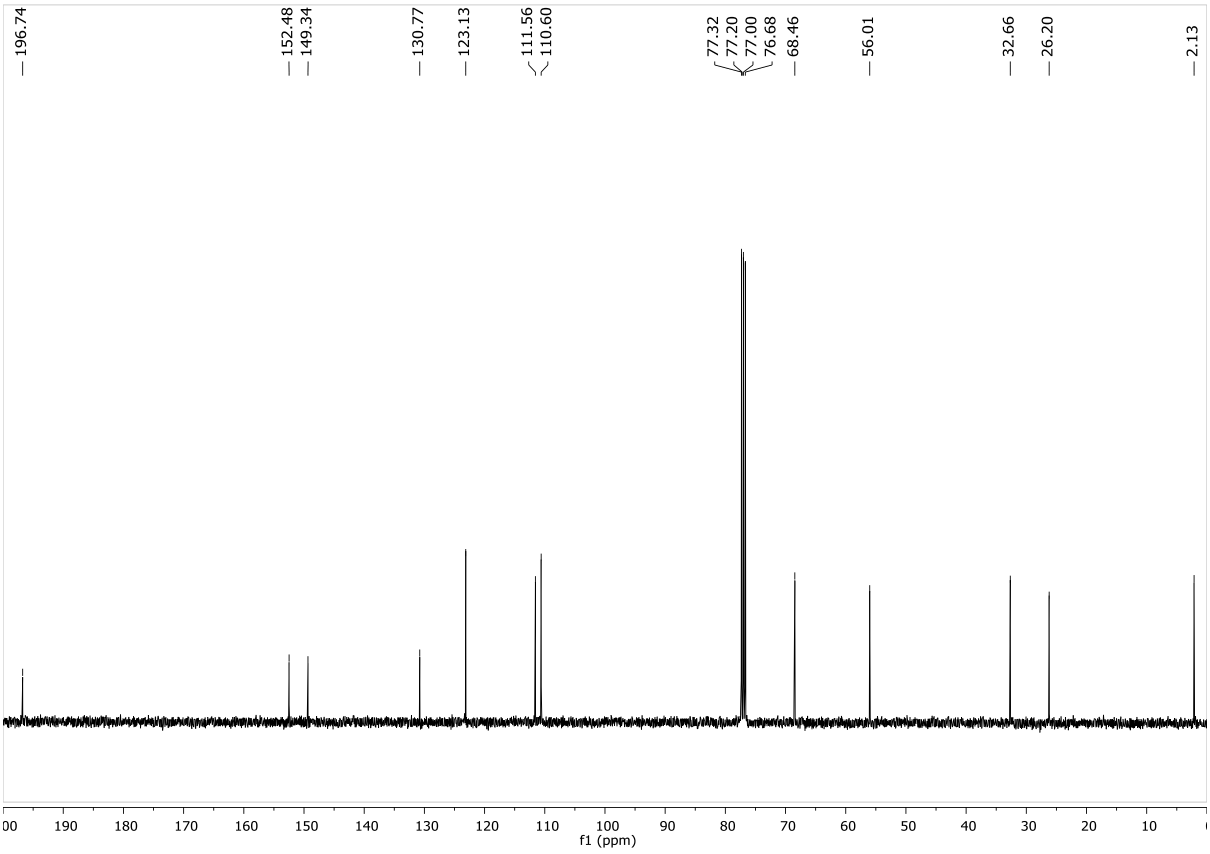
196.74
152.48
149.34
130.77
123.13
111.56
110.60
77.32
77.20
77.00
76.68
68.46
56.01
32.66
26.20
2.13
00
190
180
170
160
150
140
130
120
110
100
90
80
70
60
50
40
30
20
10
f1 (ppm)

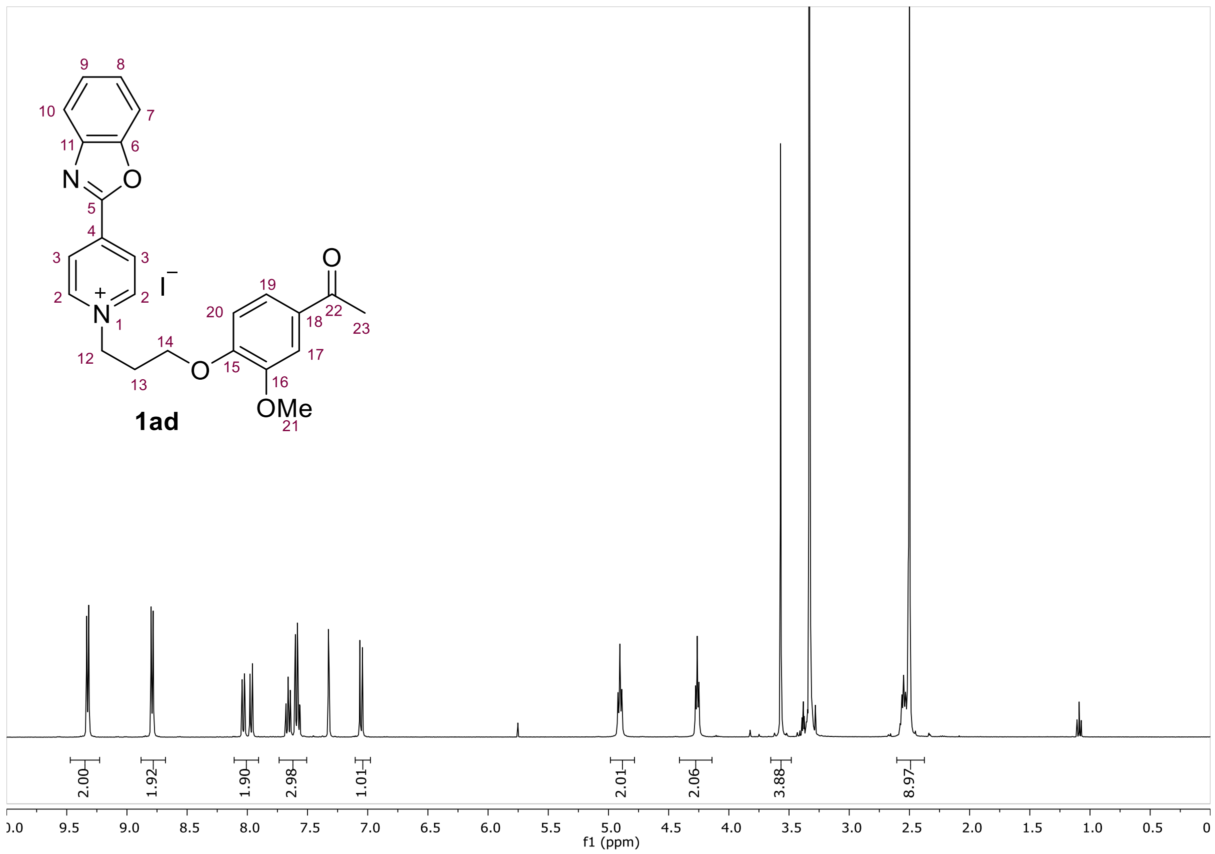
1ad
OMe
2.00
1.92
1.90
2.98
1.01
2.01
2.06
3.88
8.97
f1 (ppm)

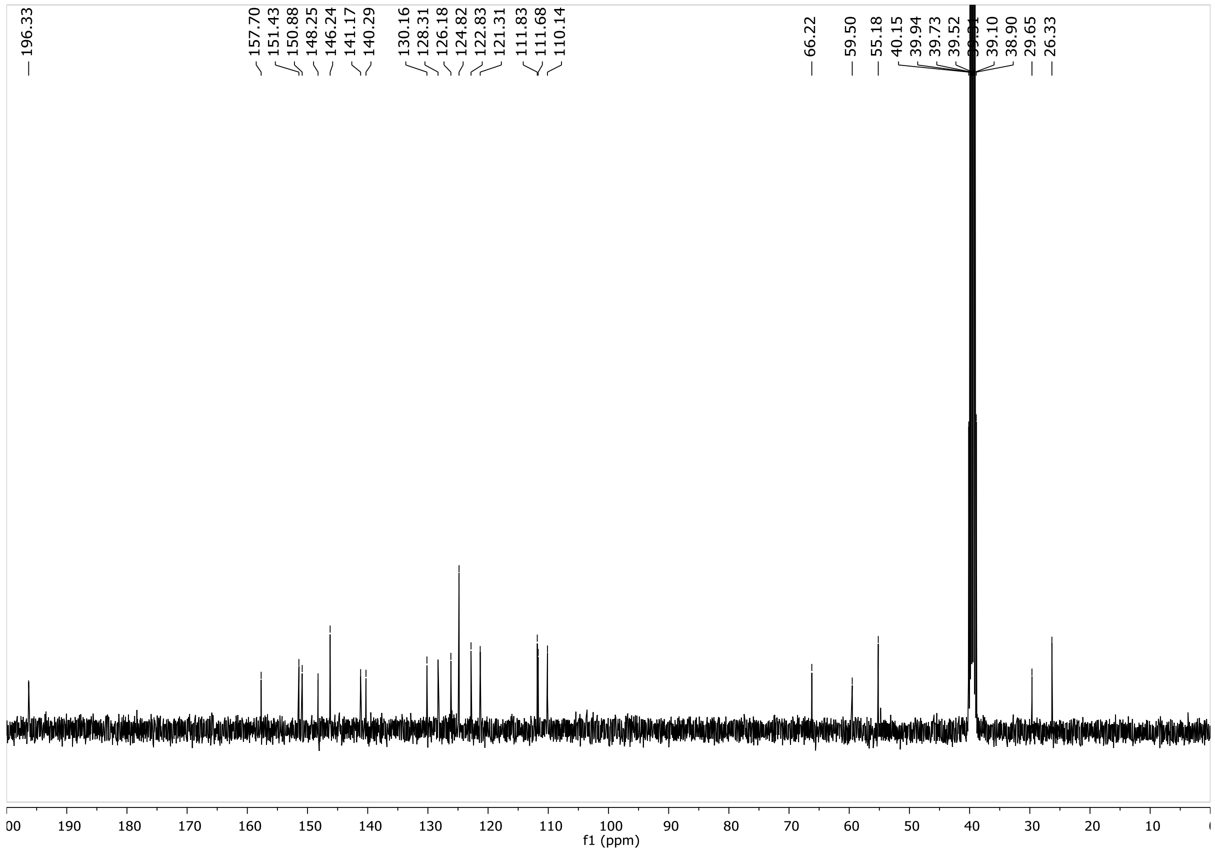
196.33
157.70
151.43
150.88
148.25
146.24
141.17
140.29
130.16
128.31
126.18
124.82
122.83
121.31
111.83
111.68
110.14
66.22
59.50
55.18
40.15
39.94
39.73
39.52
39.10
38.90
29.65
26.33
f1 (ppm)

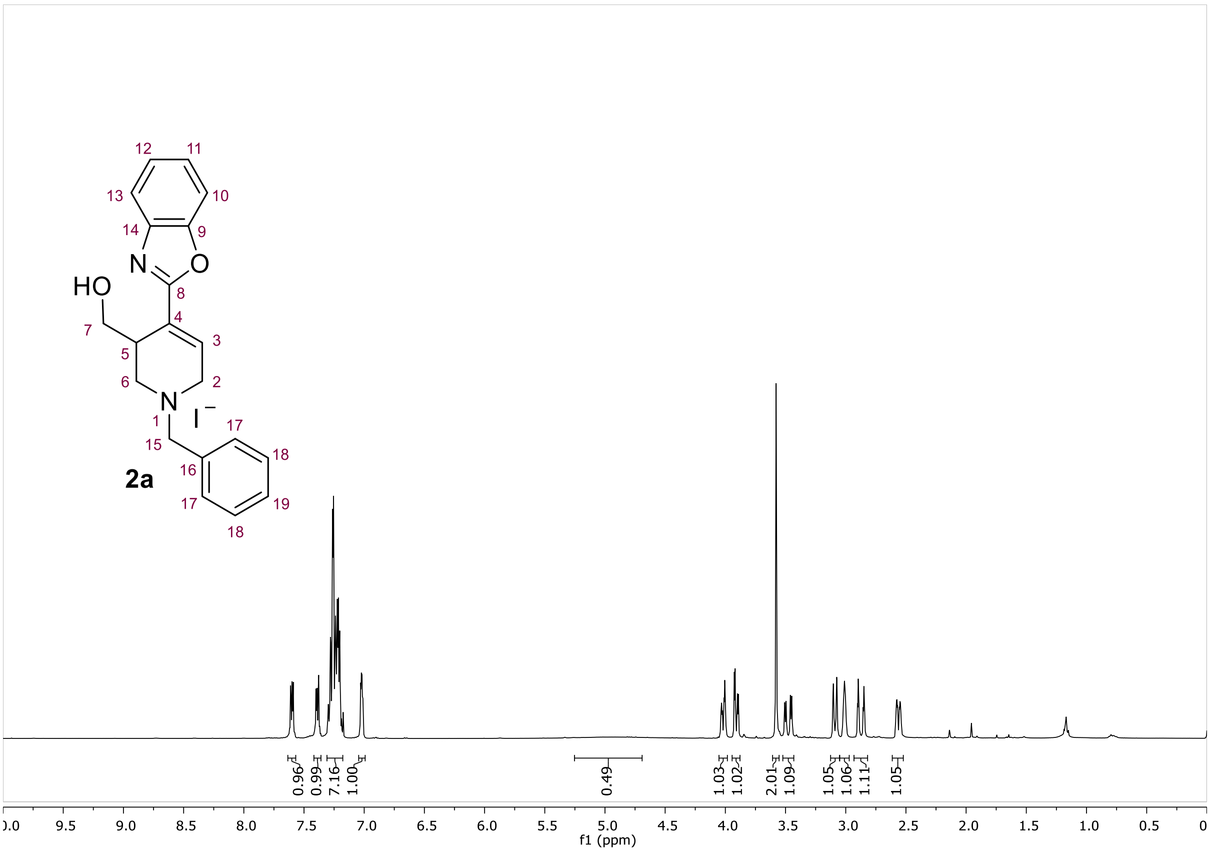
HO
N
O
N
I−
2a
0.96
0.99
7.16
1.00
0.49
1.03
1.02
2.01
1.09
1.05
1.06
1.11
1.05
f1 (ppm)

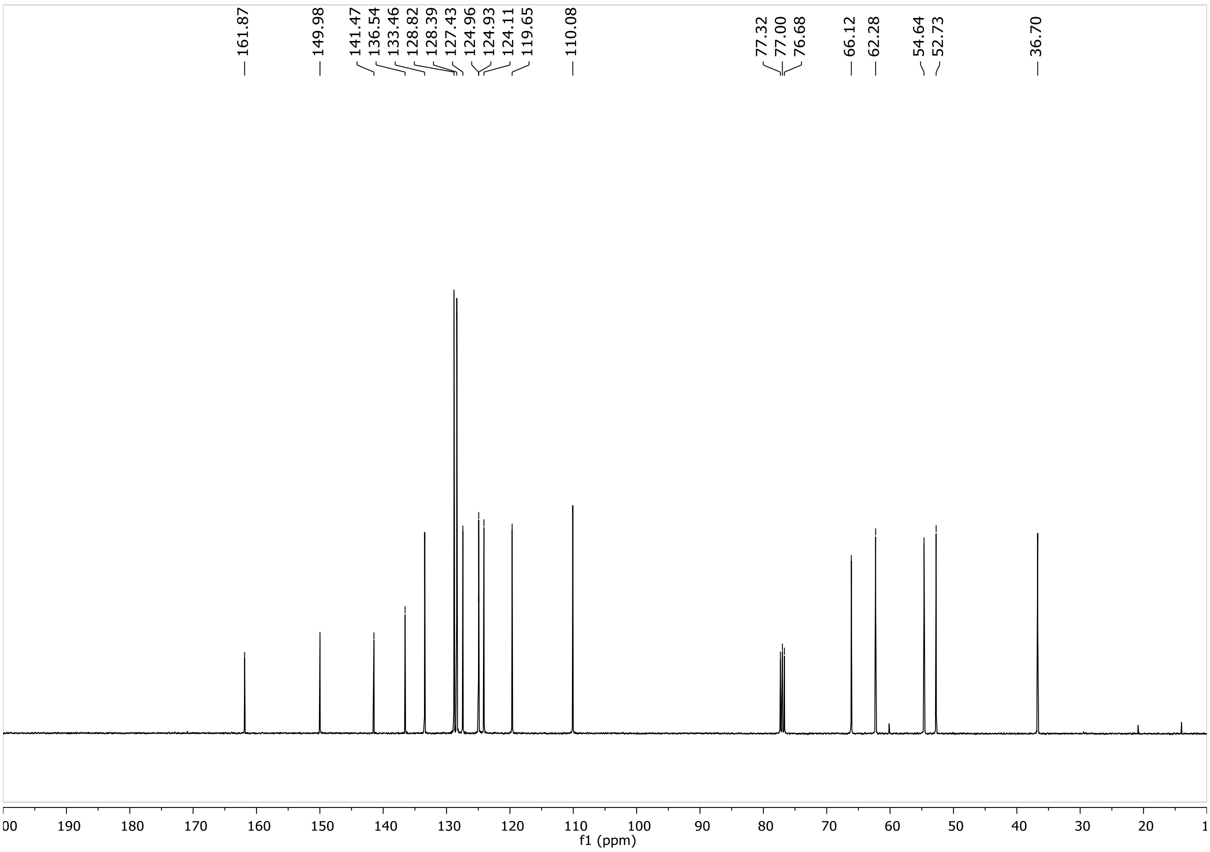
161.87
149.98
141.47
136.54
133.46
128.82
128.39
127.43
124.96
124.93
124.11
119.65
110.08
77.32
77.00
76.68
66.12
62.28
54.64
52.73
36.70
f1 (ppm)

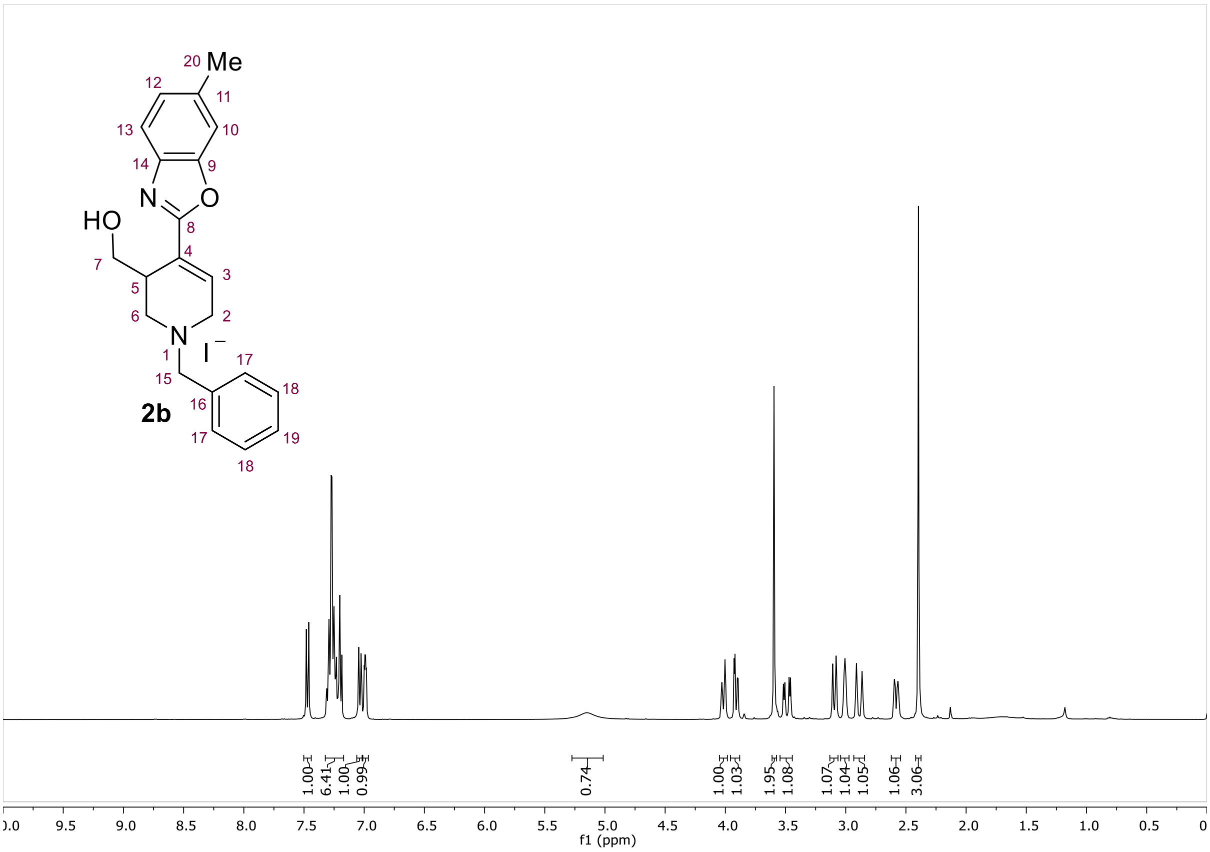
Me
HO
N
O
N
I⁻
2b
1.00
6.41
1.00
0.99
0.74
1.00
1.03
1.95
1.08
1.07
1.04
1.05
1.06
3.06
f1 (ppm)

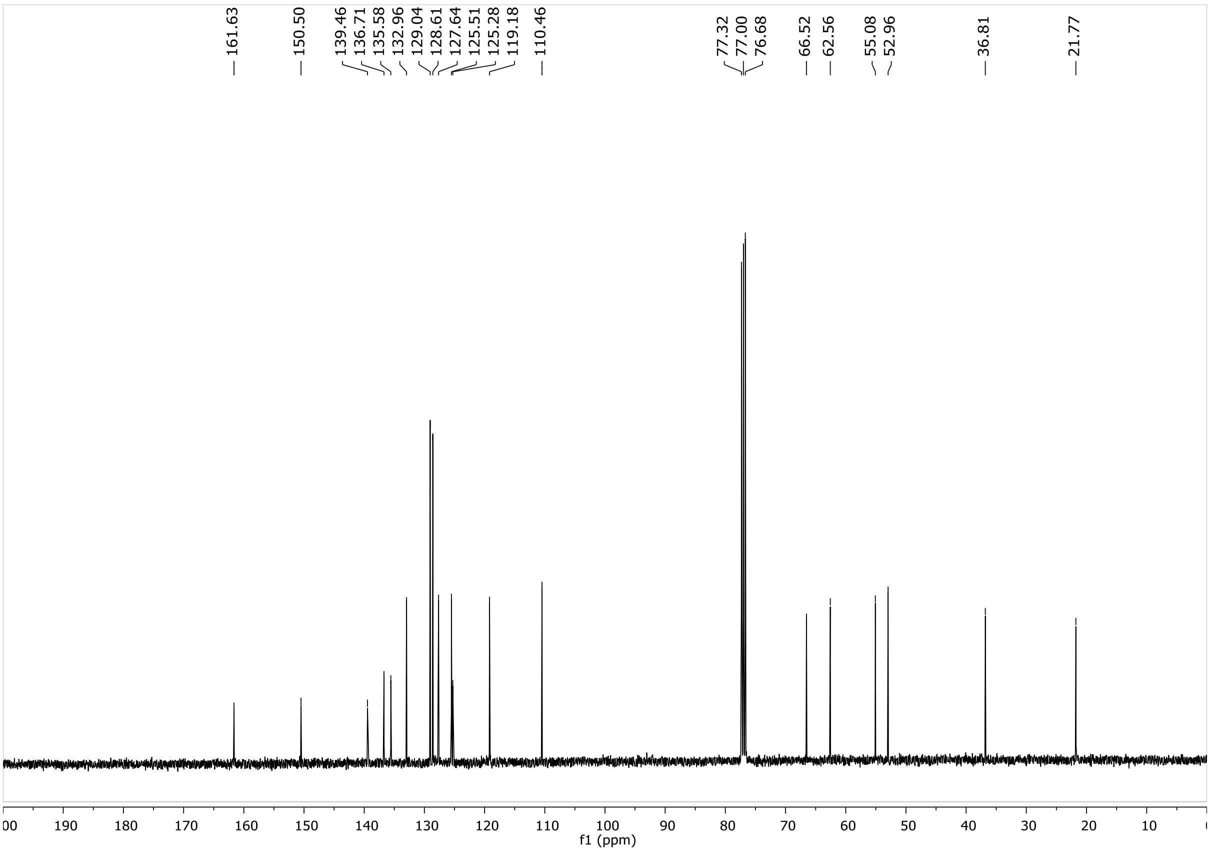
161.63
150.50
139.46
136.71
135.58
132.96
129.04
128.61
127.64
125.51
125.28
119.18
110.46
77.32
77.00
76.68
66.52
62.56
55.08
52.96
36.81
21.77
f1 (ppm)

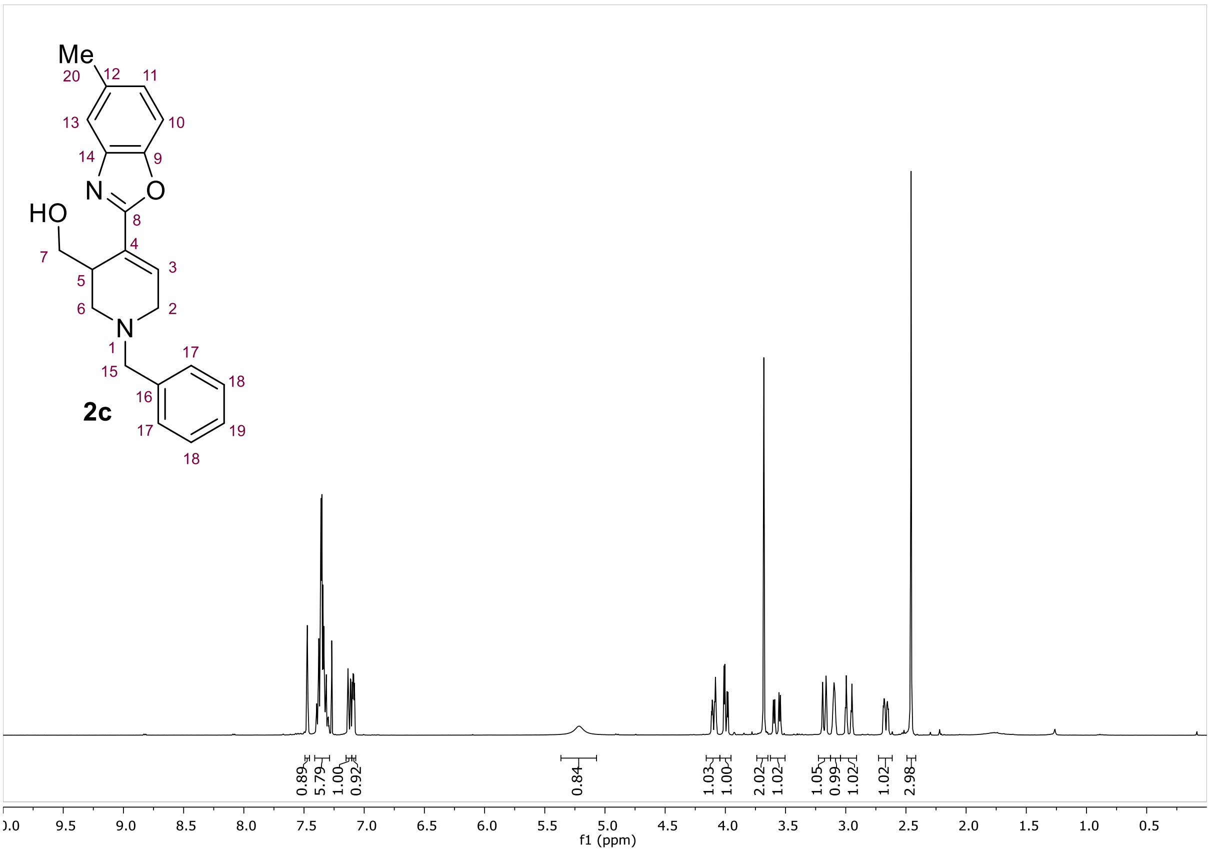
Me
HO
N
O
N
2c
0.89
5.79
1.00
0.92
0.84
1.03
1.00
2.02
1.02
1.05
0.99
1.02
1.02
2.98
f1 (ppm)

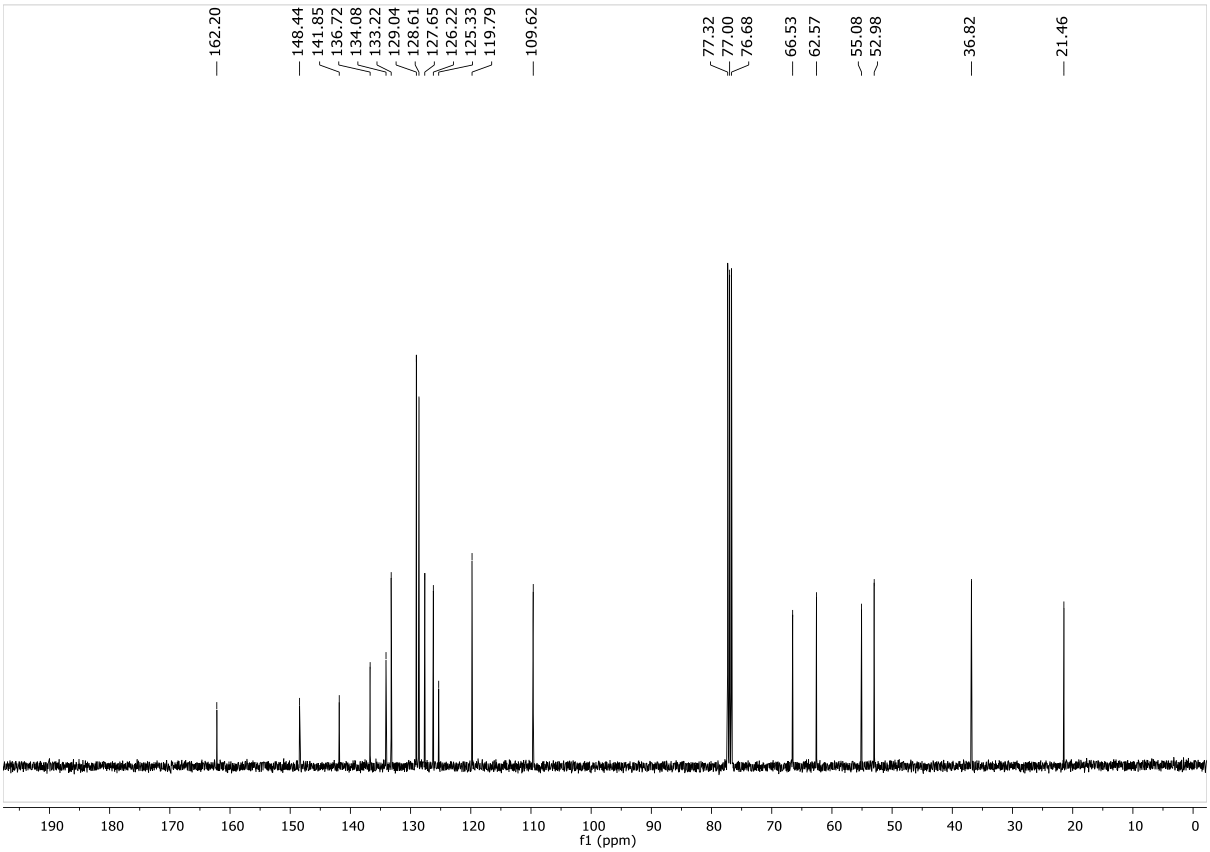
162.20
148.44
141.85
136.72
134.08
133.22
129.04
128.61
127.65
126.22
125.33
119.79
109.62
77.32
77.00
76.68
66.53
62.57
55.08
52.98
36.82
21.46
f1 (ppm)

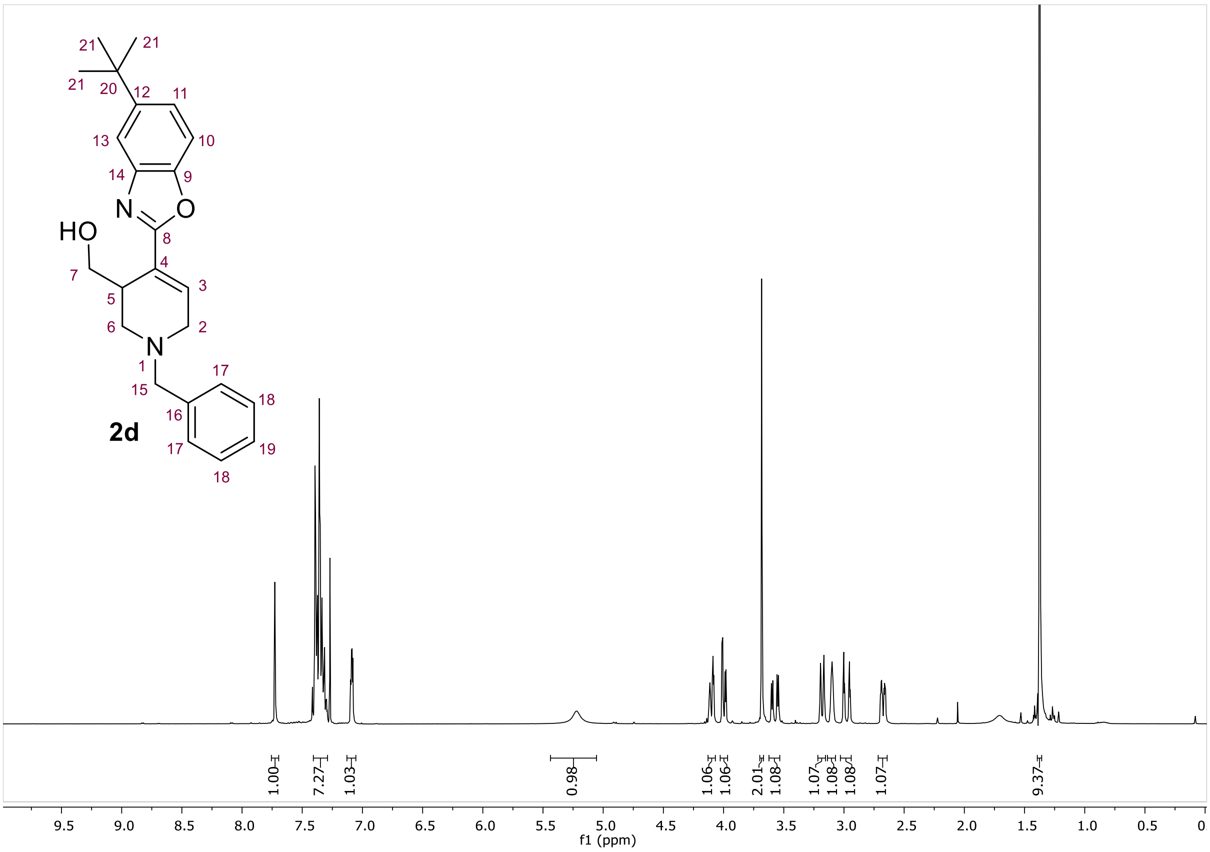

HO
N
O
N
2d
1.00
7.27
1.03
0.98
1.06
1.06
2.01
1.08
1.07
1.08
1.08
1.07
9.37
f1 (ppm)

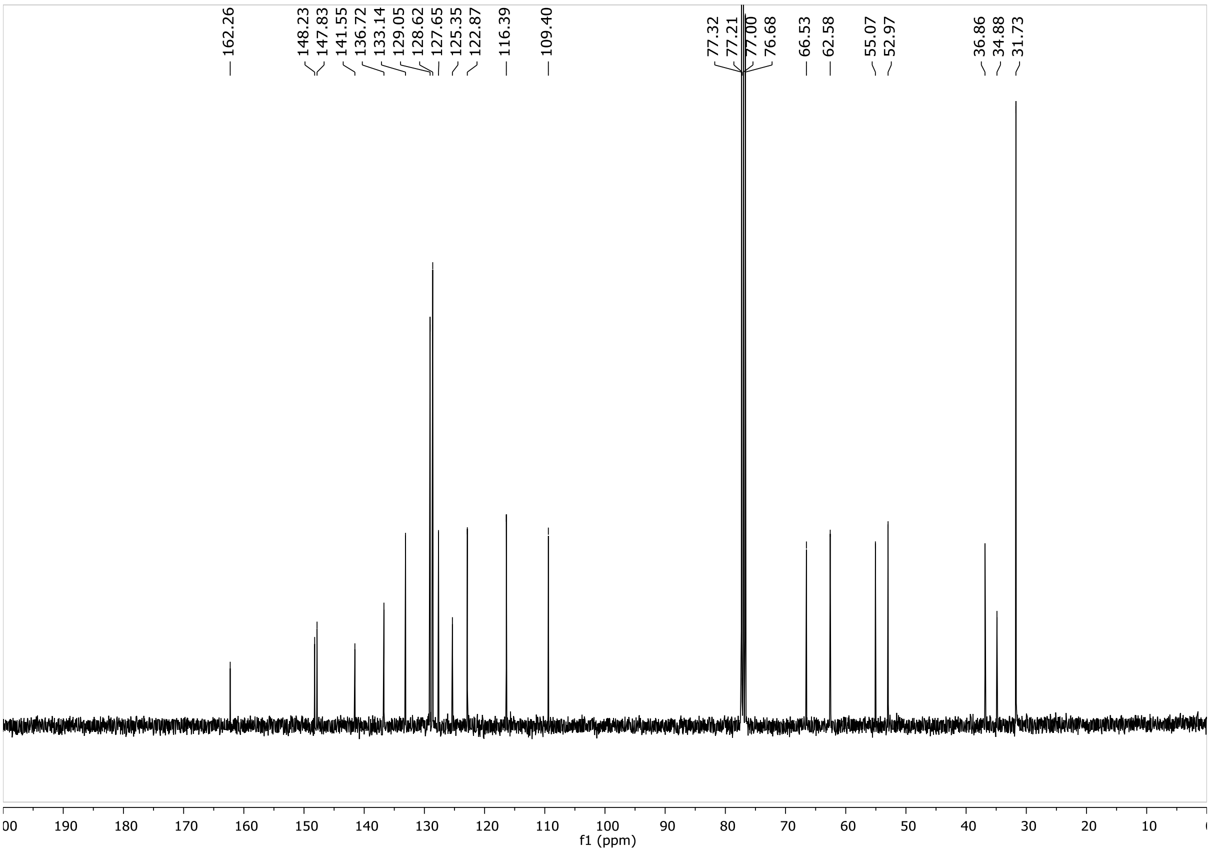

162.26
148.23
147.83
141.55
136.72
133.14
129.05
128.62
127.65
125.35
122.87
116.39
109.40
77.32
77.21
77.00
76.68
66.53
62.58
55.07
52.97
36.86
34.88
31.73
f1 (ppm)

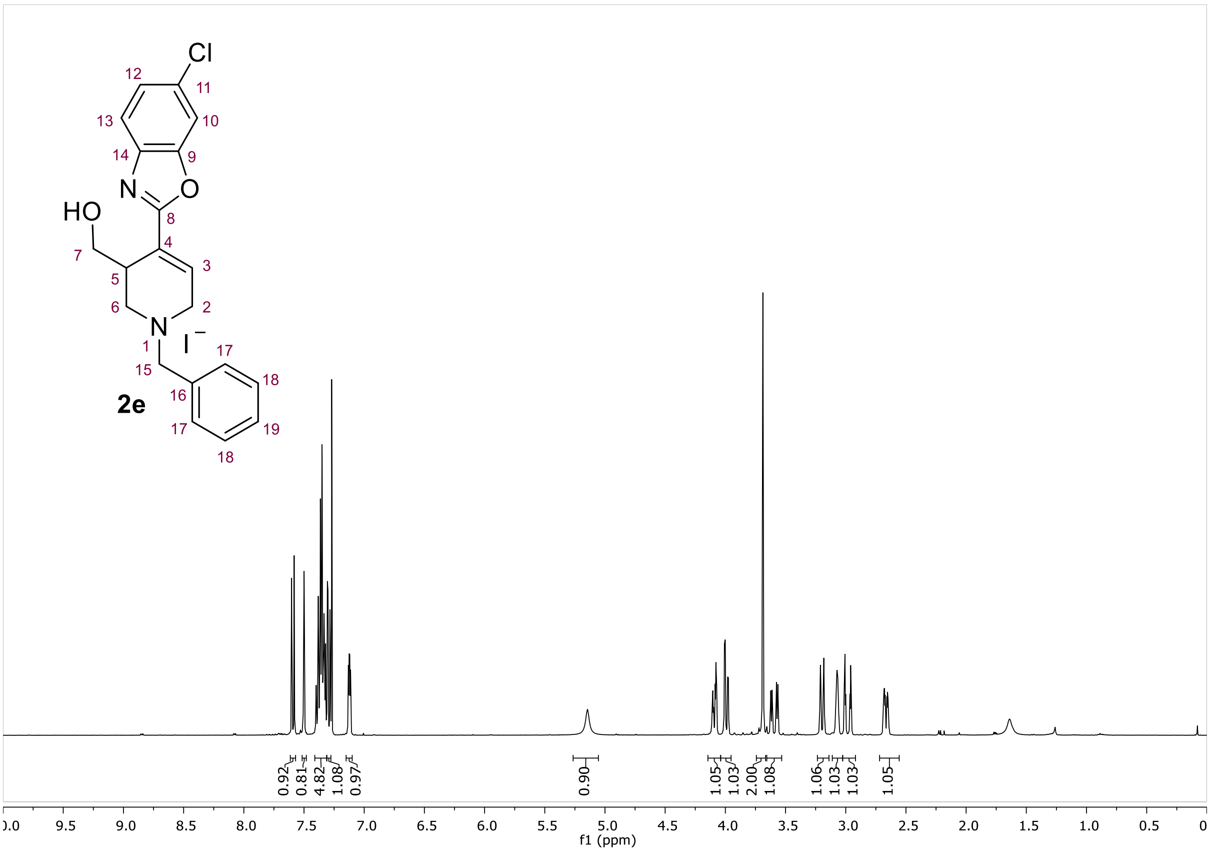
Cl
12
11
13
10
14
9
N
O
HO
8
4
7
3
5
6
2
N
I⁻
1
17
15
18
16
2e
17
19
18
0.92
0.81
4.82
1.08
0.97
0.90
1.05
1.03
2.00
1.08
1.06
1.03
1.03
1.05
f1 (ppm)

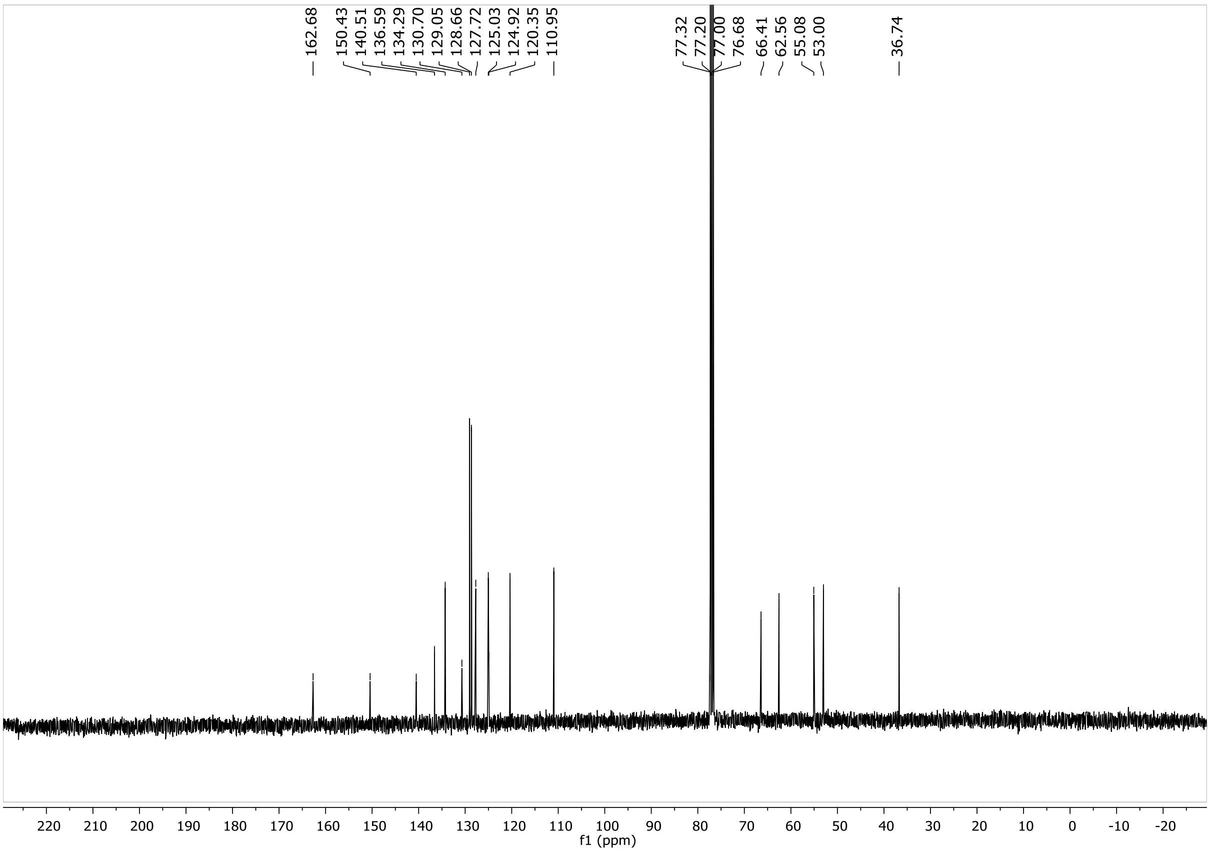
162.68
150.43
140.51
136.59
134.29
130.70
129.05
128.66
127.72
125.03
124.92
120.35
110.95
77.32
77.20
77.00
76.68
66.41
62.56
55.08
53.00
36.74
f1 (ppm)

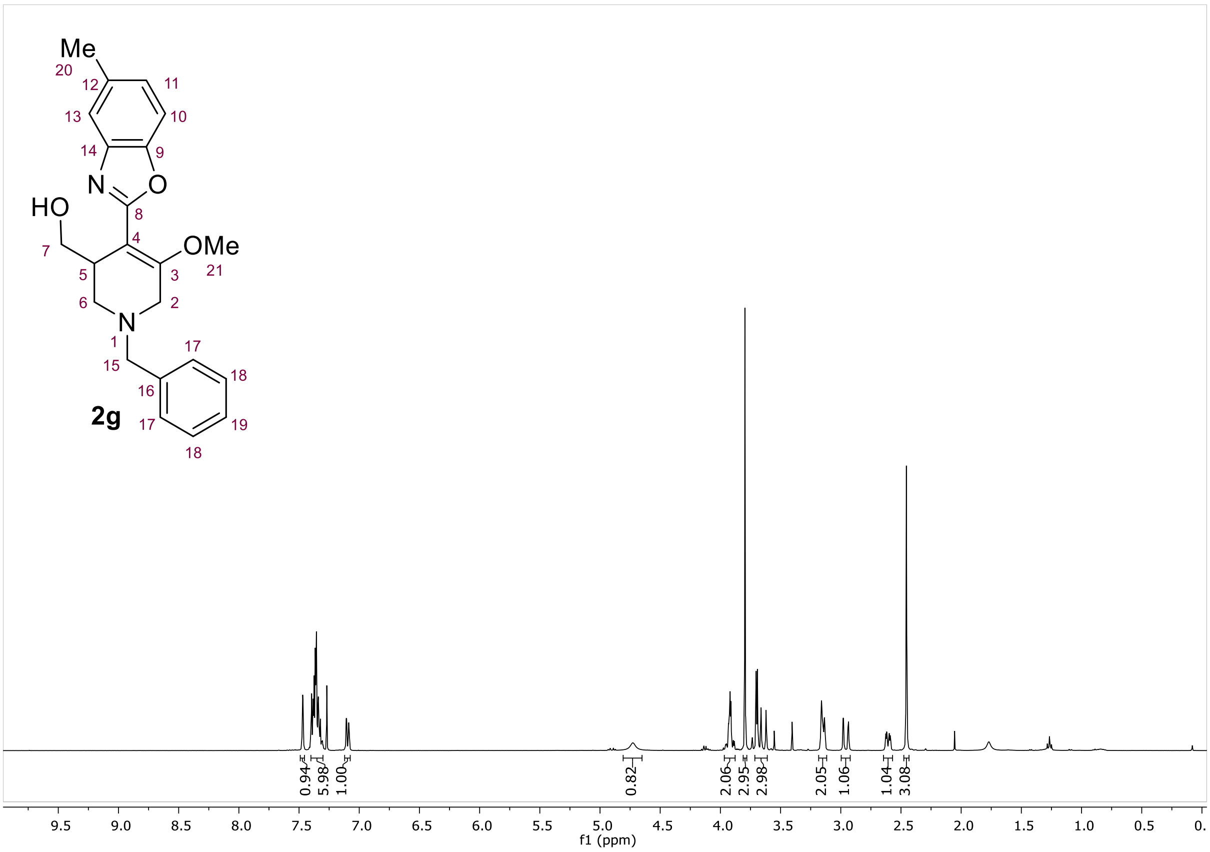
Me
HO
OMe
2g
0.94
5.98
1.00
0.82
2.06
2.95
2.98
2.05
1.06
1.04
3.08
f1 (ppm)

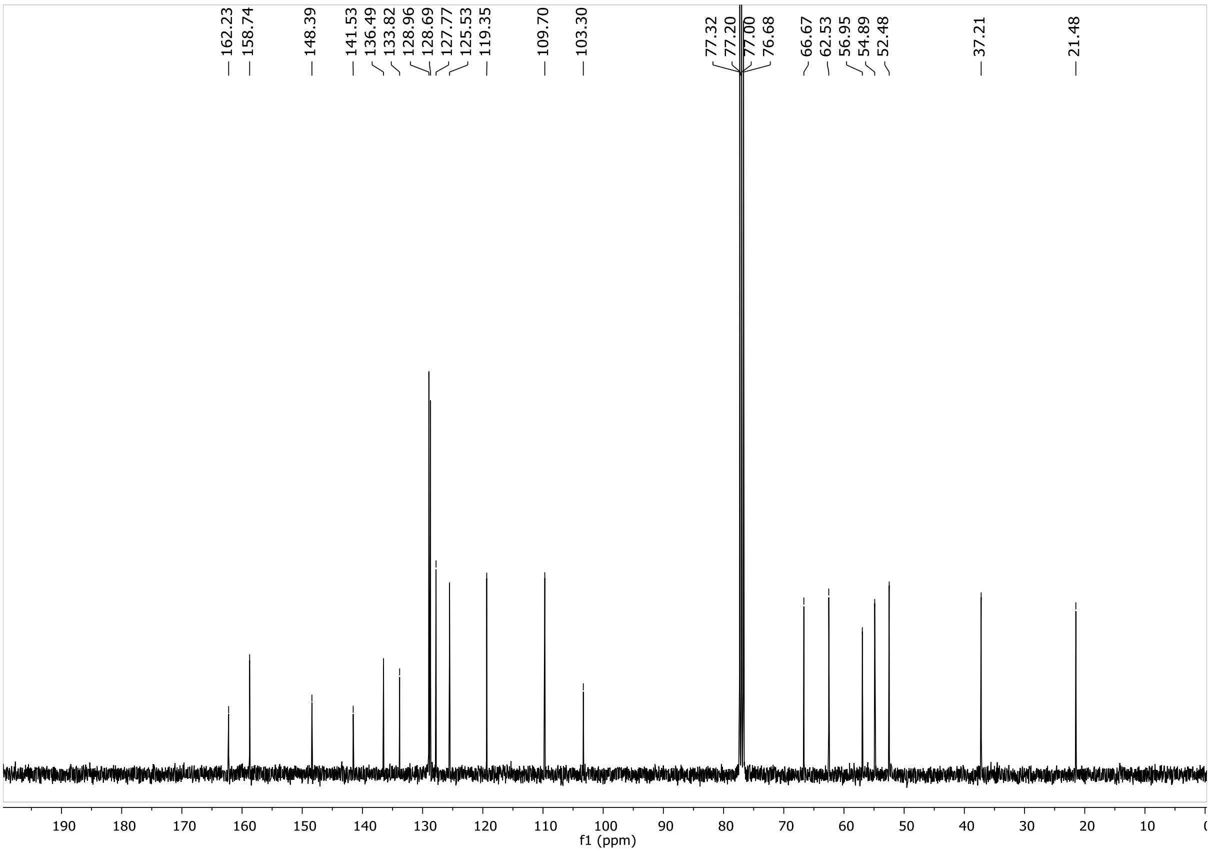
162.23
158.74
148.39
141.53
136.49
133.82
128.96
128.69
127.77
125.53
119.35
109.70
103.30
77.32
77.20
77.00
76.68
66.67
62.53
56.95
54.89
52.48
37.21
21.48
f1 (ppm)

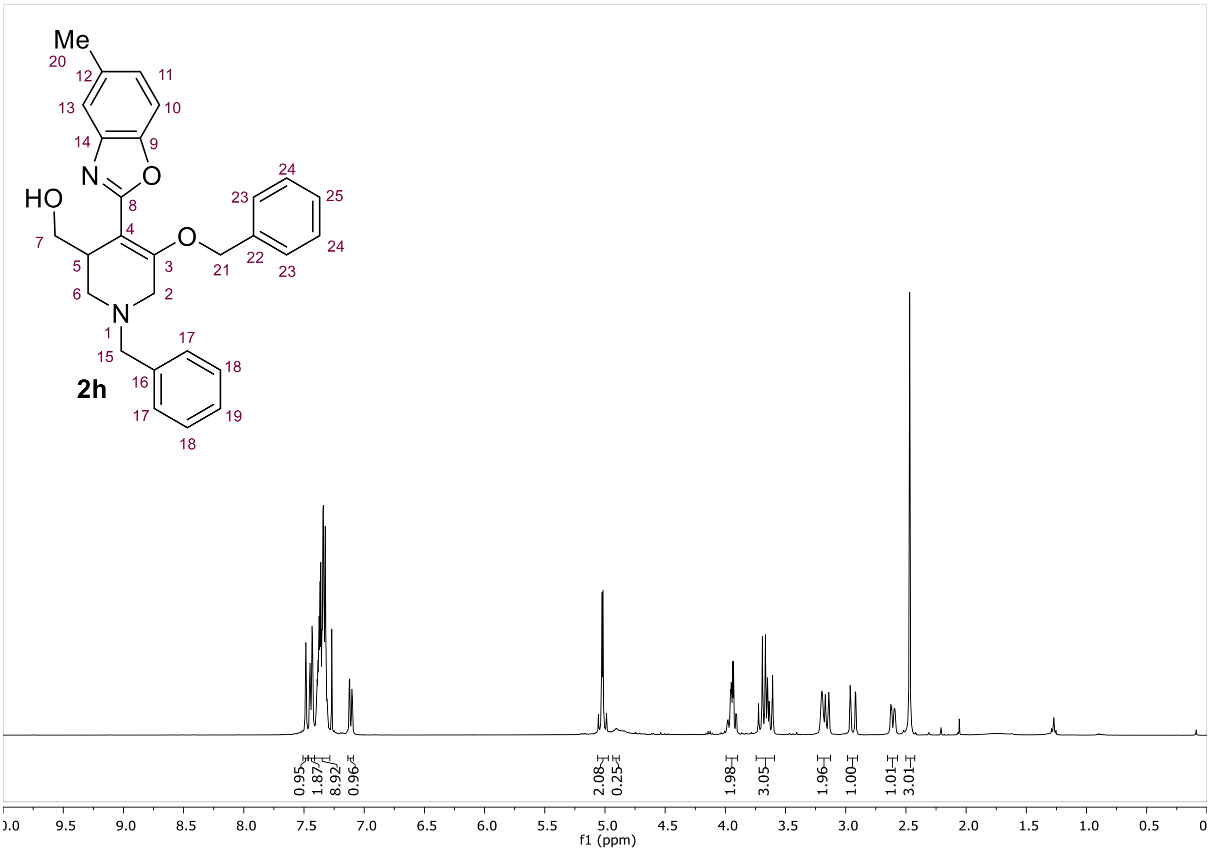
Me
2h
HO
N
O
0.95
1.87
8.92
0.96
2.08
0.25
1.98
3.05
1.96
1.00
1.01
3.01
f1 (ppm)

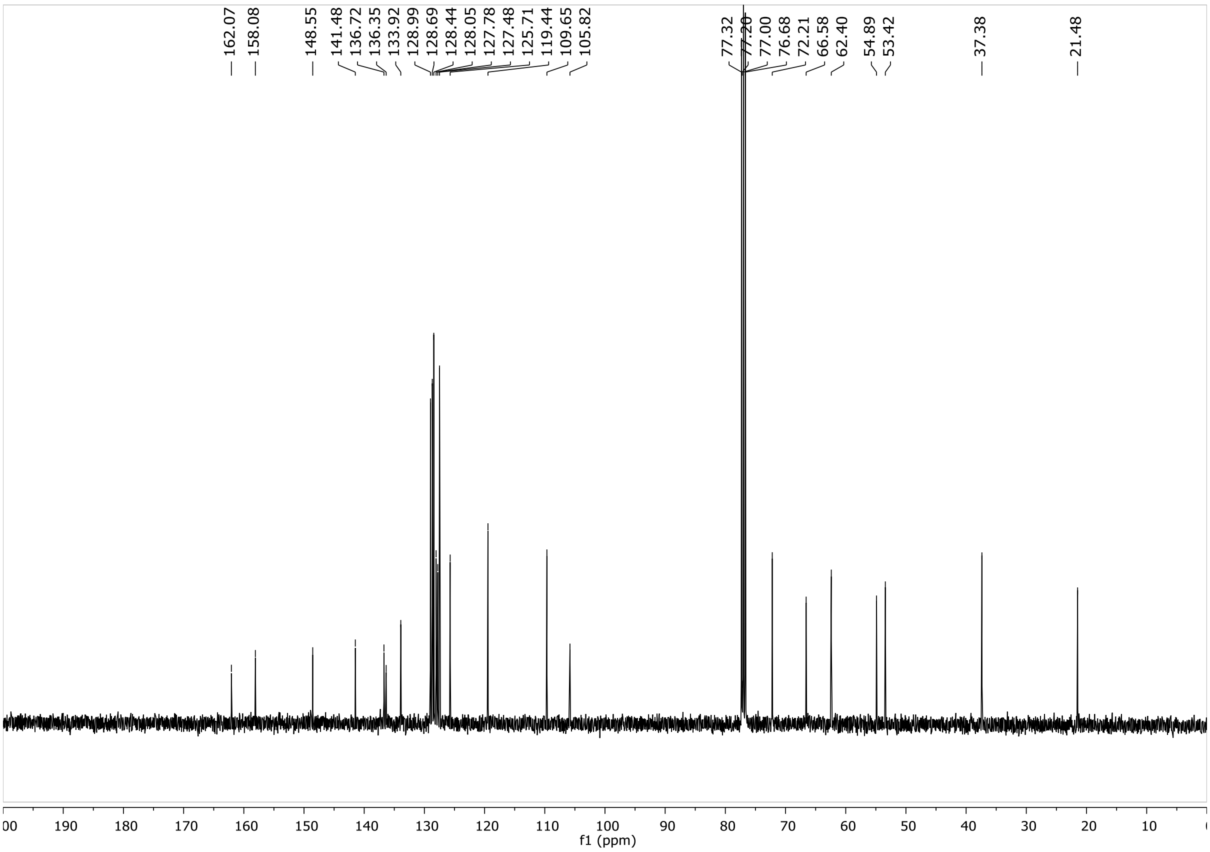
162.07
158.08
148.55
141.48
136.72
136.35
133.92
128.99
128.69
128.44
128.05
127.78
127.48
125.71
119.44
109.65
105.82
77.32
77.20
77.00
76.68
72.21
66.58
62.40
54.89
53.42
37.38
21.48
f1 (ppm)

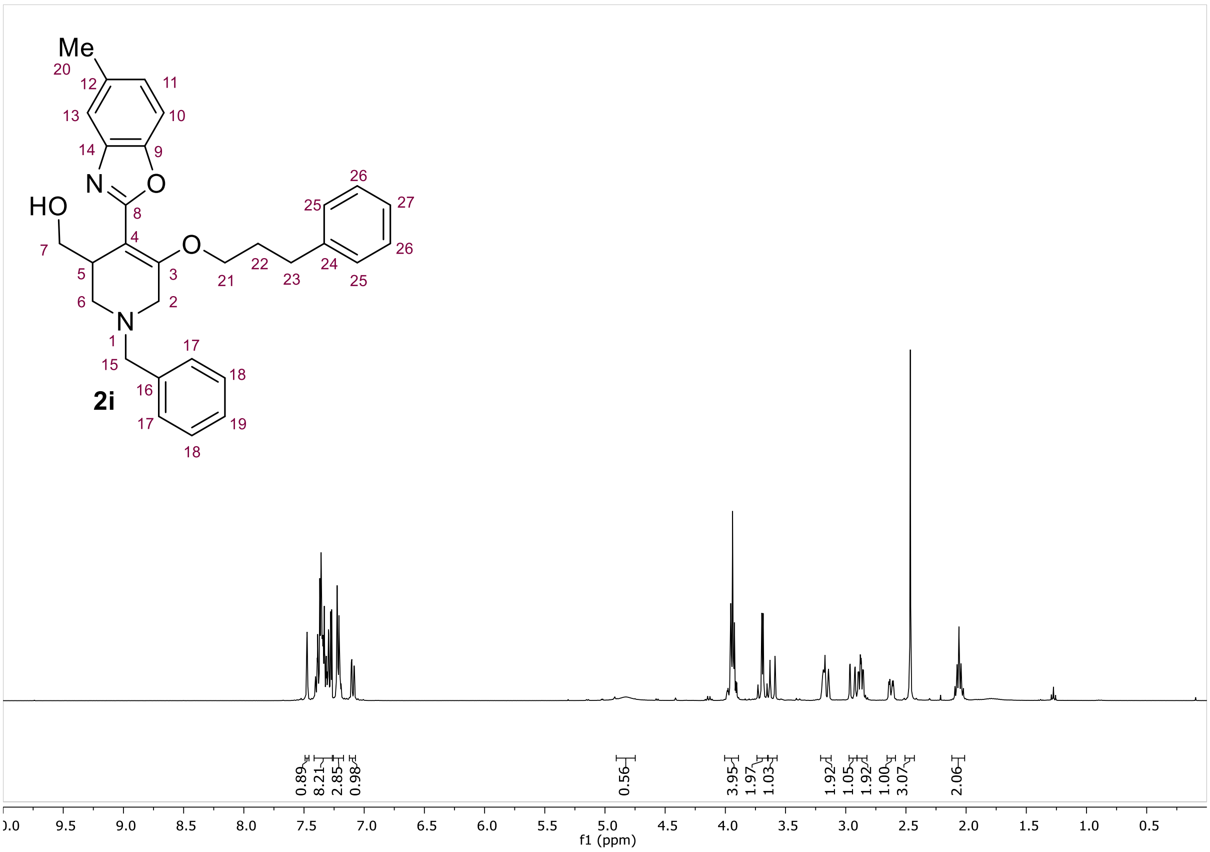
Me
HO
N
O
N
2i
0.89
8.21
2.85
0.98
0.56
3.95
1.97
1.03
1.92
1.05
1.92
1.00
3.07
2.06
f1 (ppm)

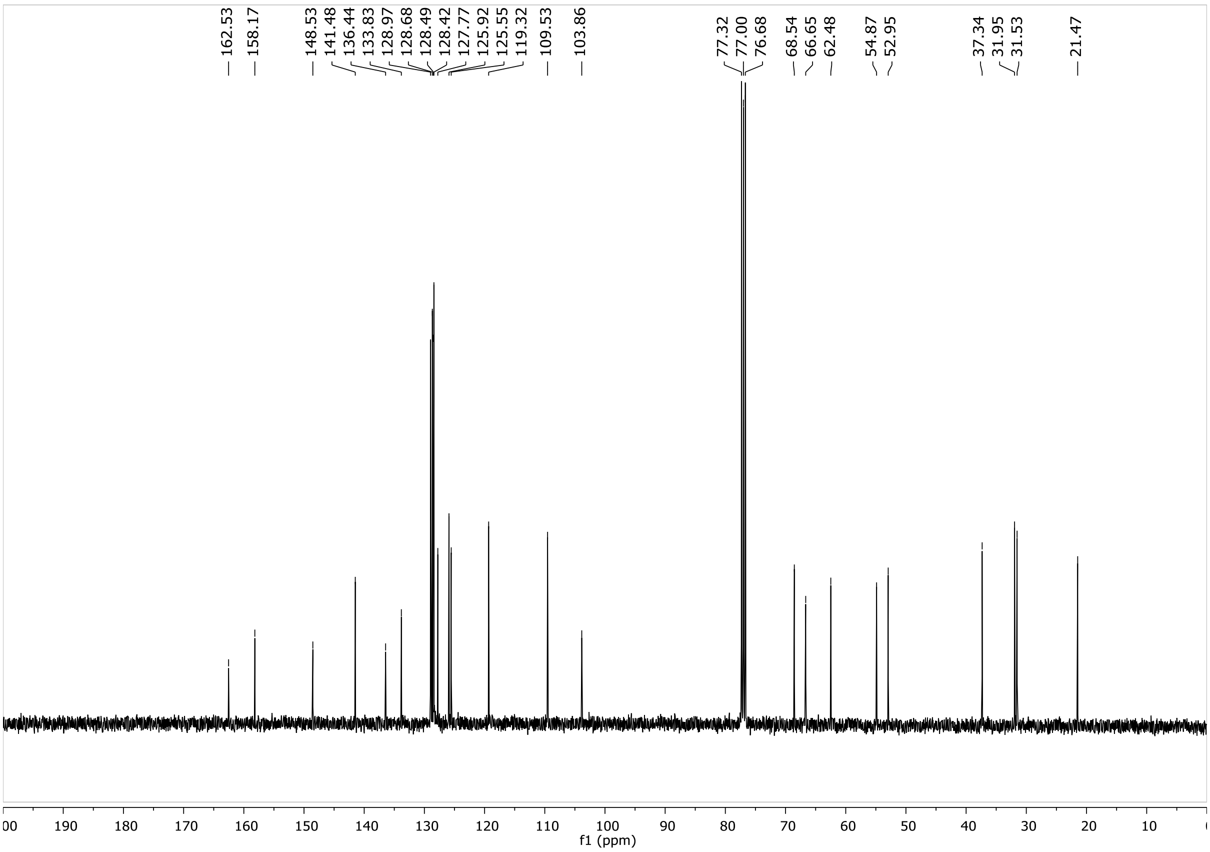
162.53
158.17
148.53
141.48
136.44
133.83
128.97
128.68
128.49
128.42
127.77
125.92
125.55
119.32
109.53
103.86
77.32
77.00
76.68
68.54
66.65
62.48
54.87
52.95
37.34
31.95
31.53
21.47
f1 (ppm)

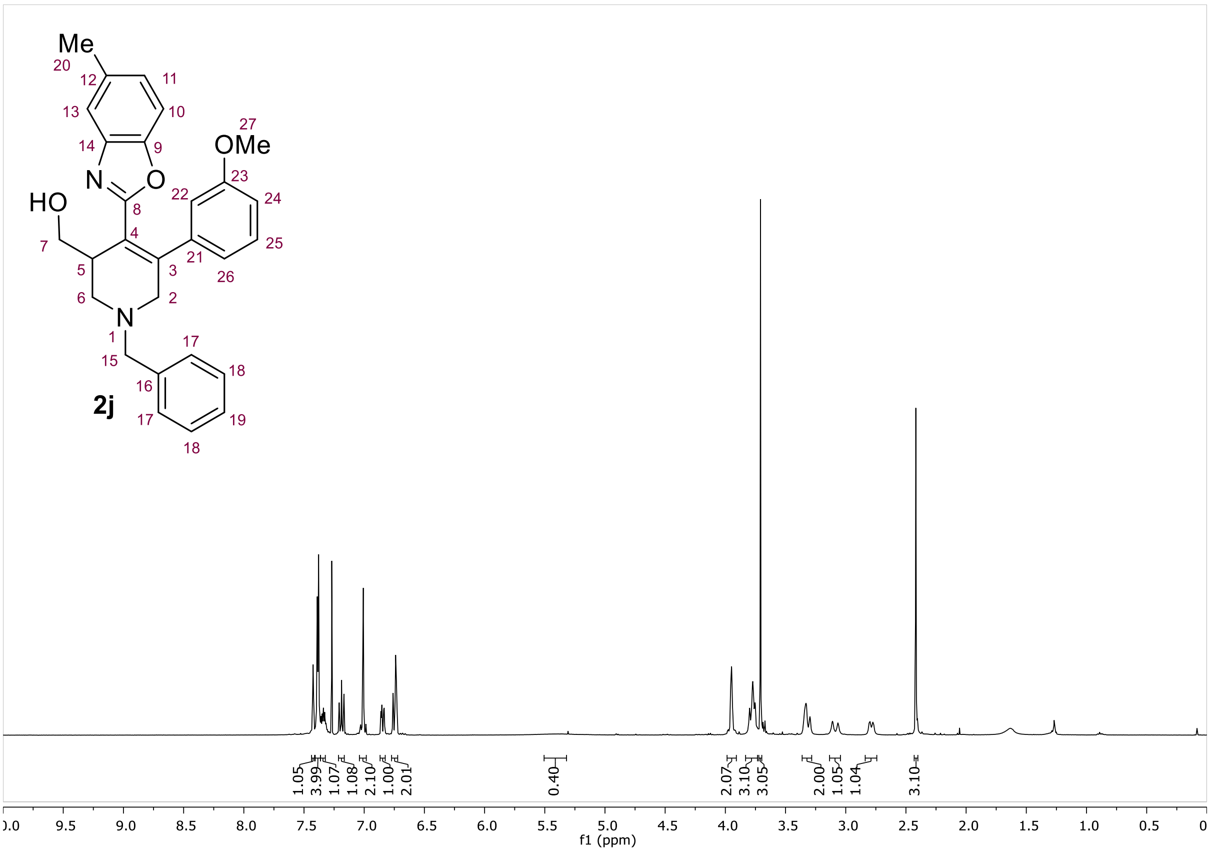
Me
OMe
HO
N
O
2j
1.05
3.99
1.07
1.08
2.10
1.00
2.01
0.40
2.07
3.10
3.05
2.00
1.05
1.04
3.10
f1 (ppm)

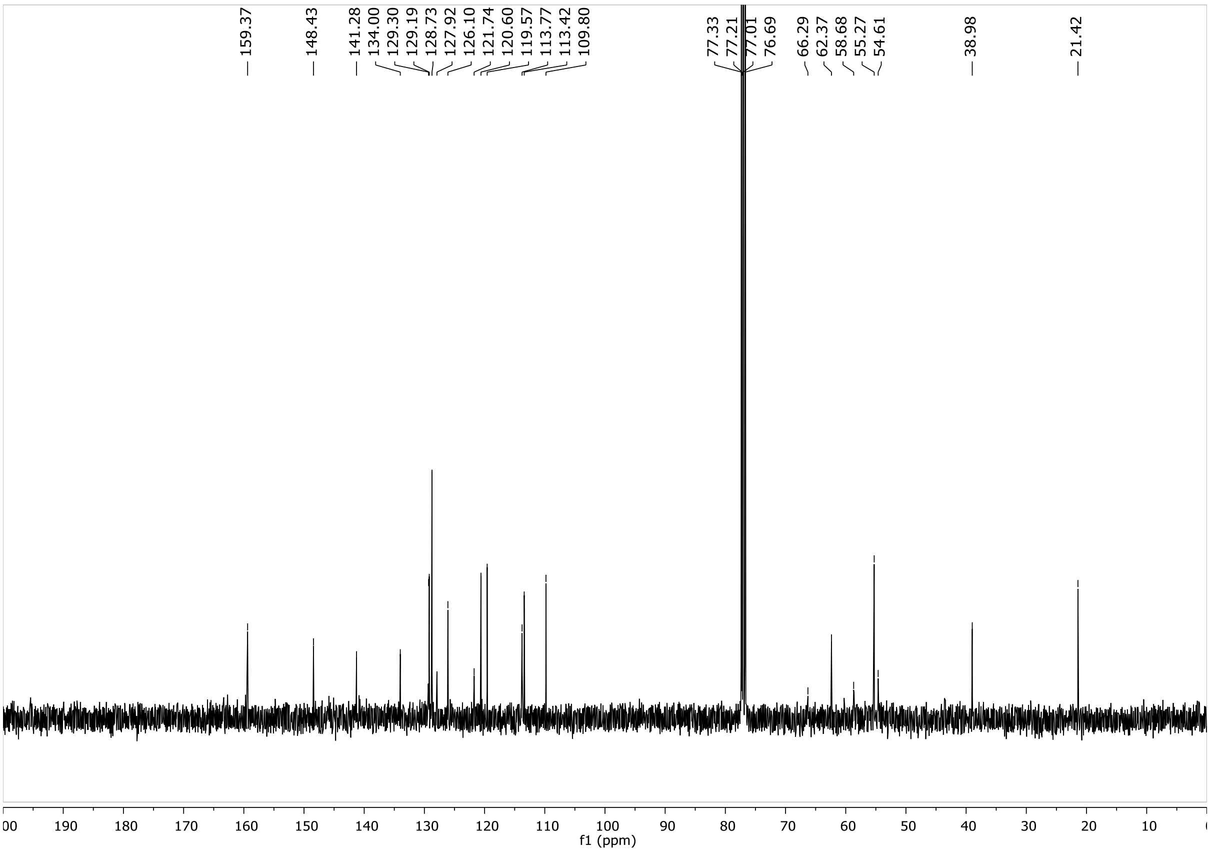
159.37
148.43
141.28
134.00
129.30
129.19
128.73
127.92
126.10
121.74
120.60
119.57
113.77
113.42
109.80
77.33
77.21
77.01
76.69
66.29
62.37
58.68
55.27
54.61
38.98
21.42
f1 (ppm)

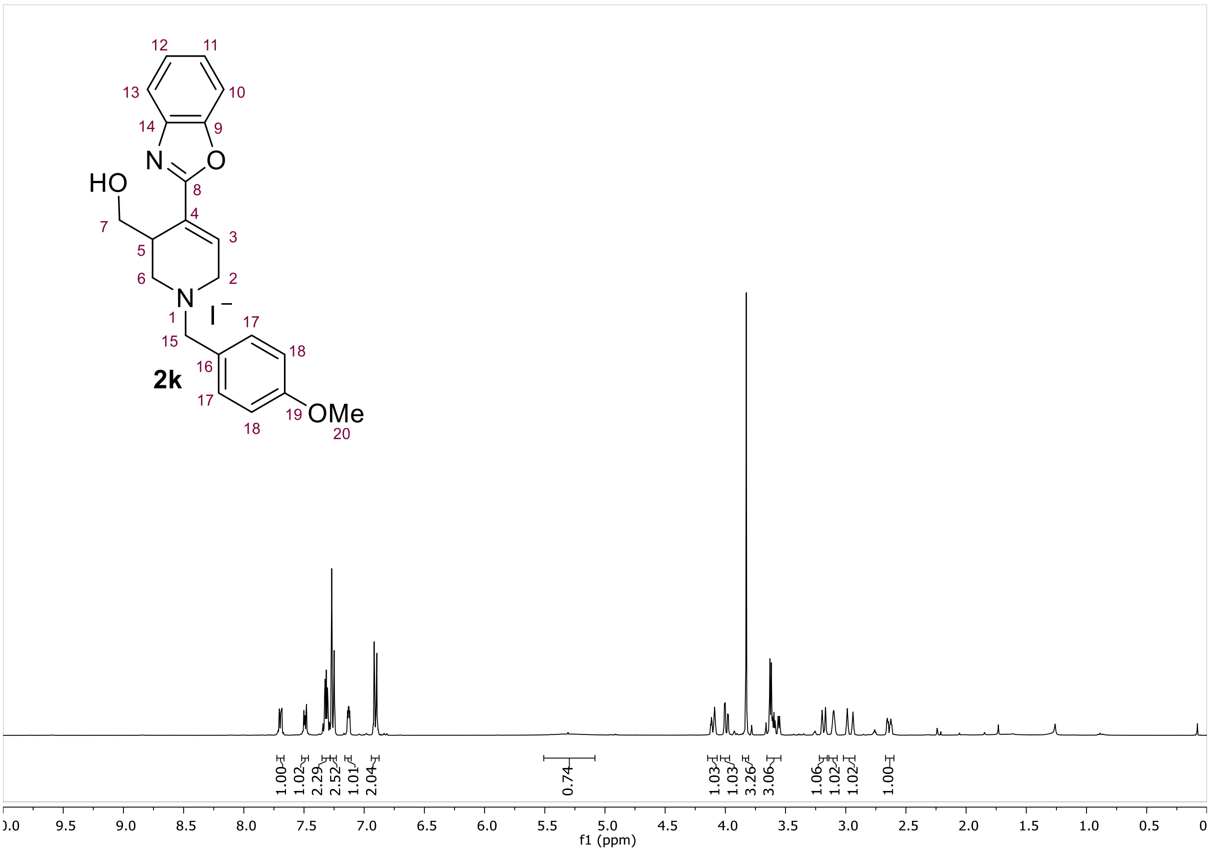
2k
HO
I⁻
OMe
1.00
1.02
2.29
2.52
1.01
2.04
0.74
1.03
1.03
3.26
3.06
1.06
1.02
1.02
1.00
f1 (ppm)

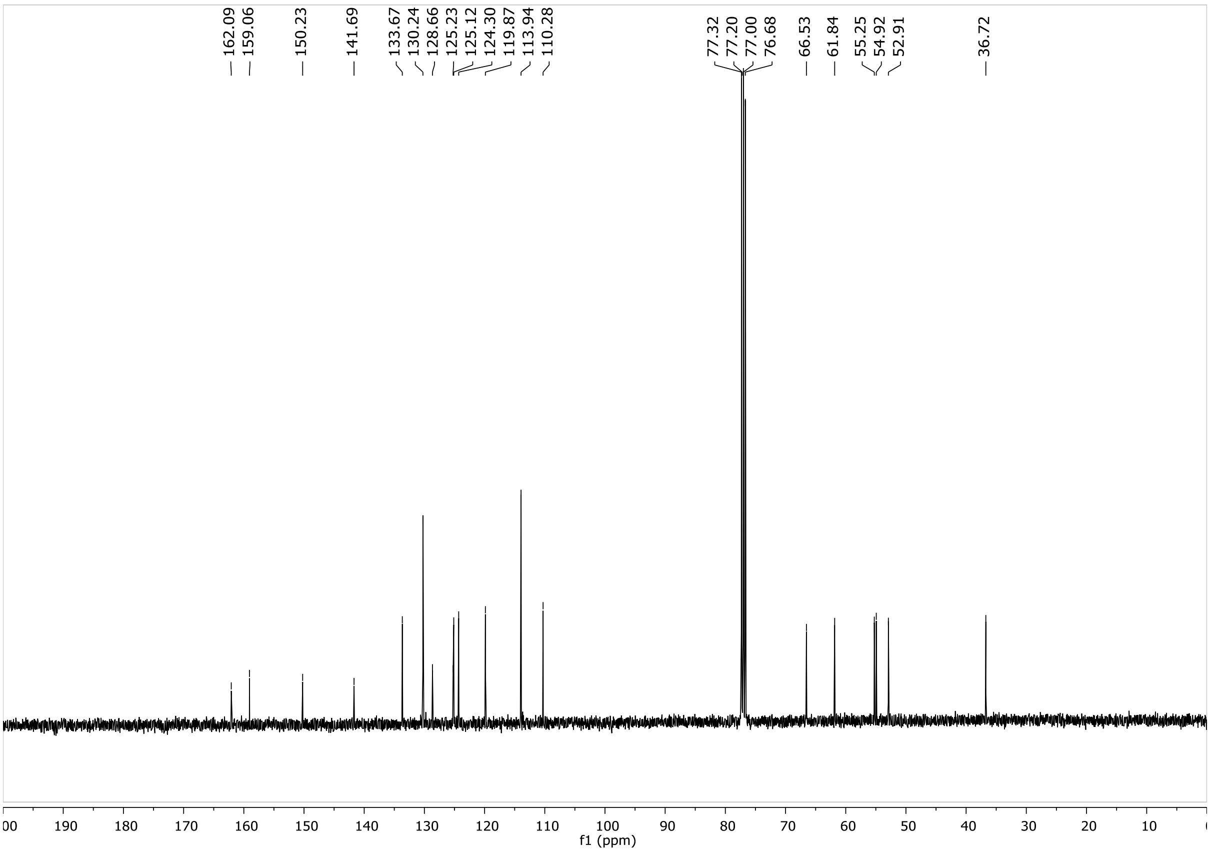
162.09
159.06
150.23
141.69
133.67
130.24
128.66
125.23
125.12
124.30
119.87
113.94
110.28
77.32
77.20
77.00
76.68
66.53
61.84
55.25
54.92
52.91
36.72
f1 (ppm)

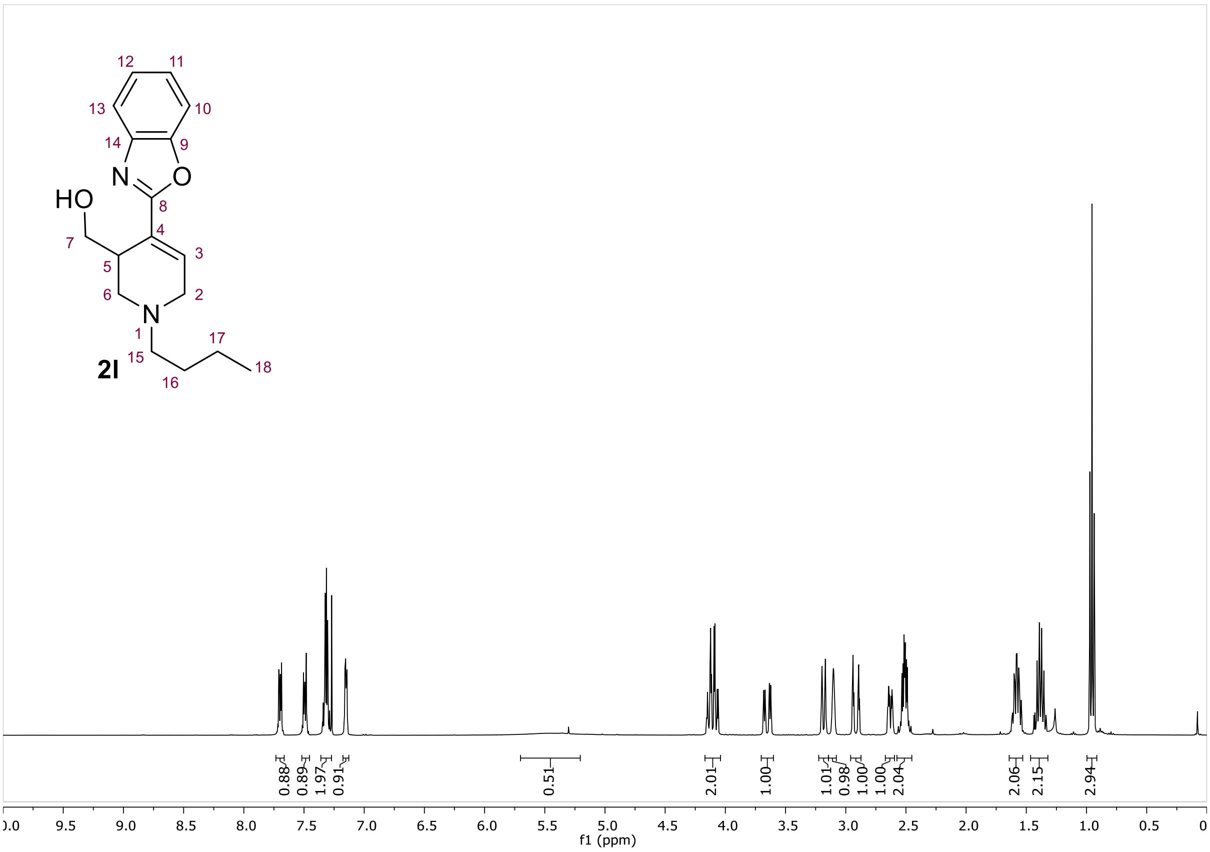

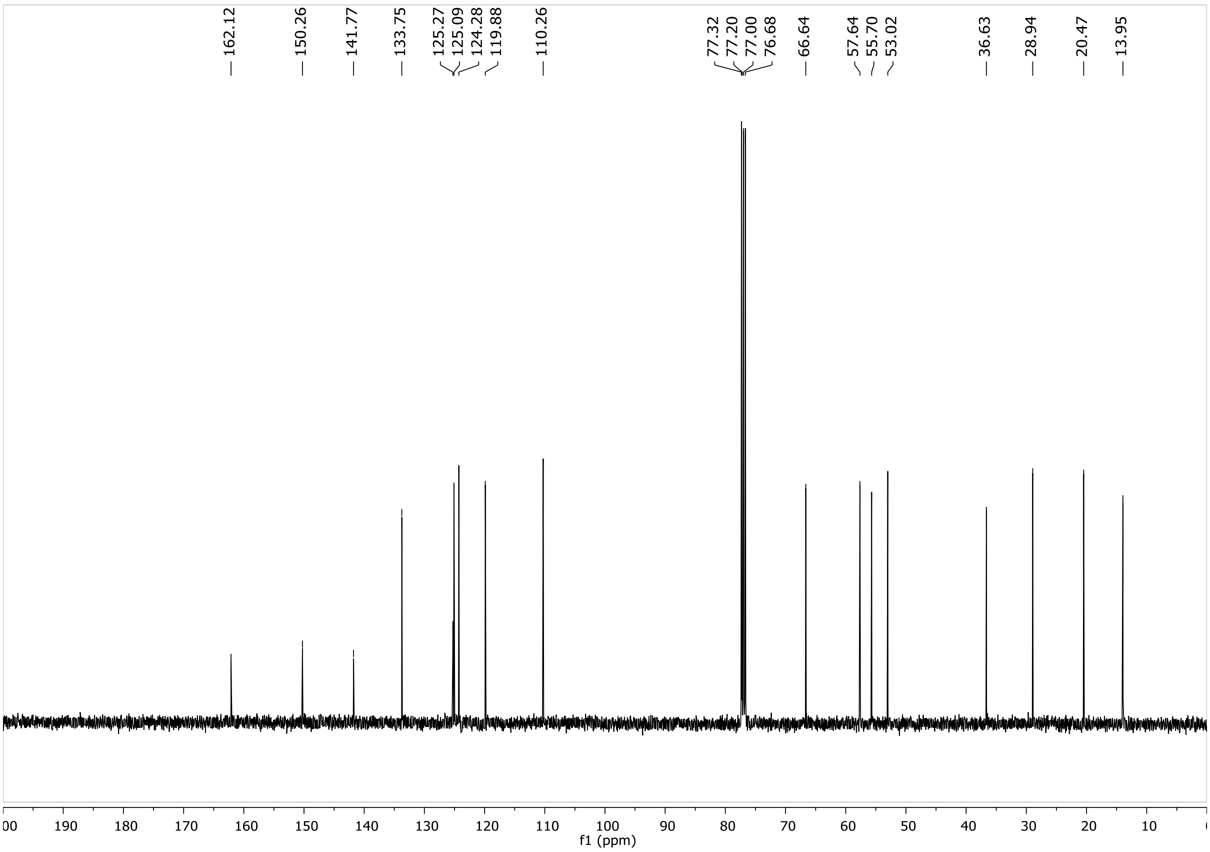

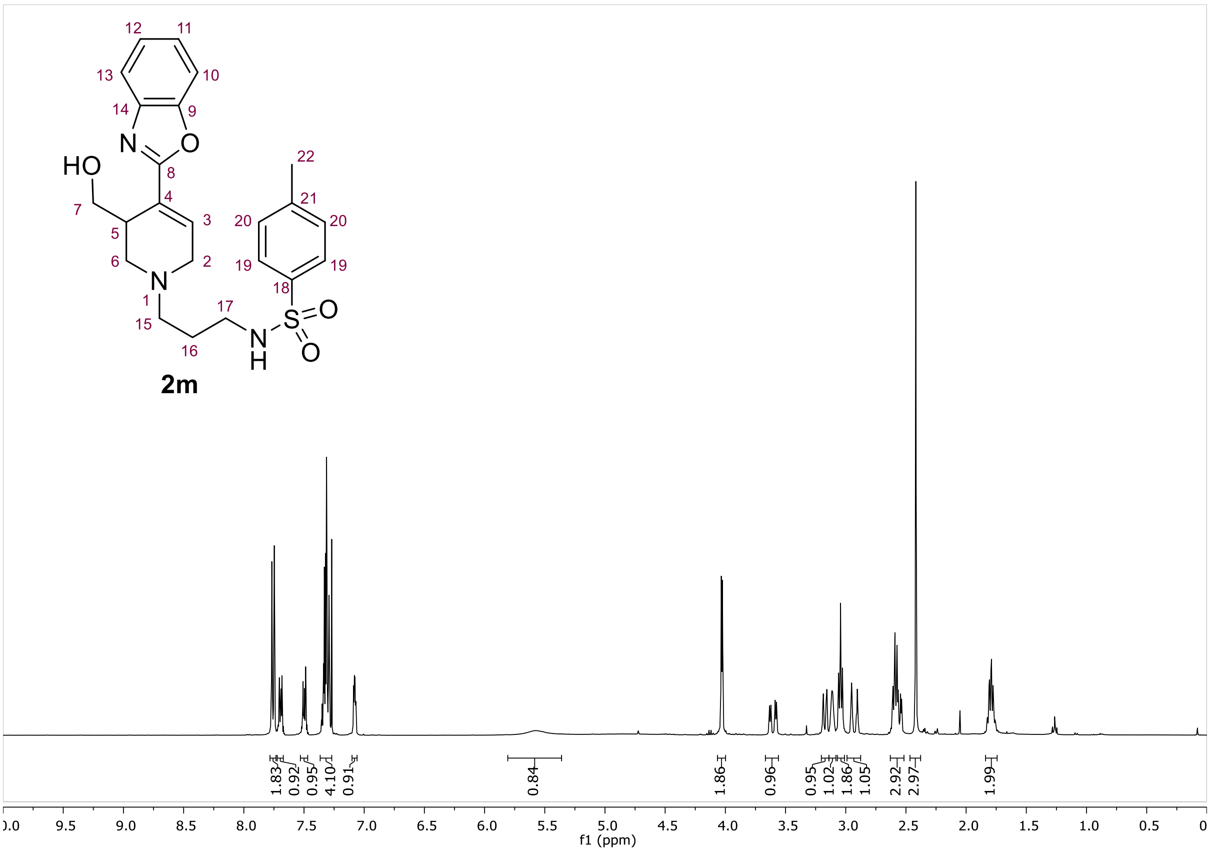
2m
1.83
0.92
0.95
4.10
0.91
0.84
1.86
0.96
0.95
1.02
1.86
1.05
2.92
2.97
1.99
f1 (ppm)

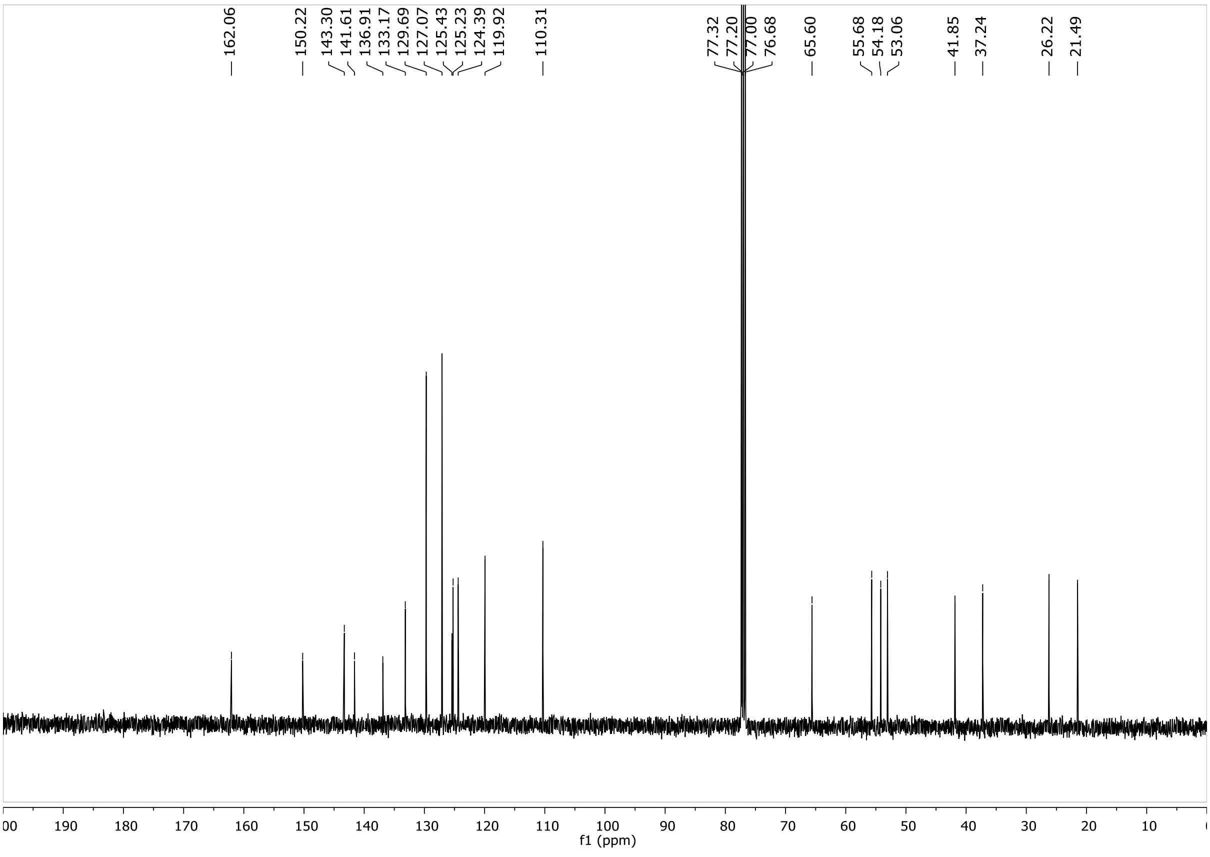
162.06
150.22
143.30
141.61
136.91
133.17
129.69
127.07
125.43
125.23
124.39
119.92
110.31
77.32
77.20
77.00
76.68
65.60
55.68
54.18
53.06
41.85
37.24
26.22
21.49
f1 (ppm)

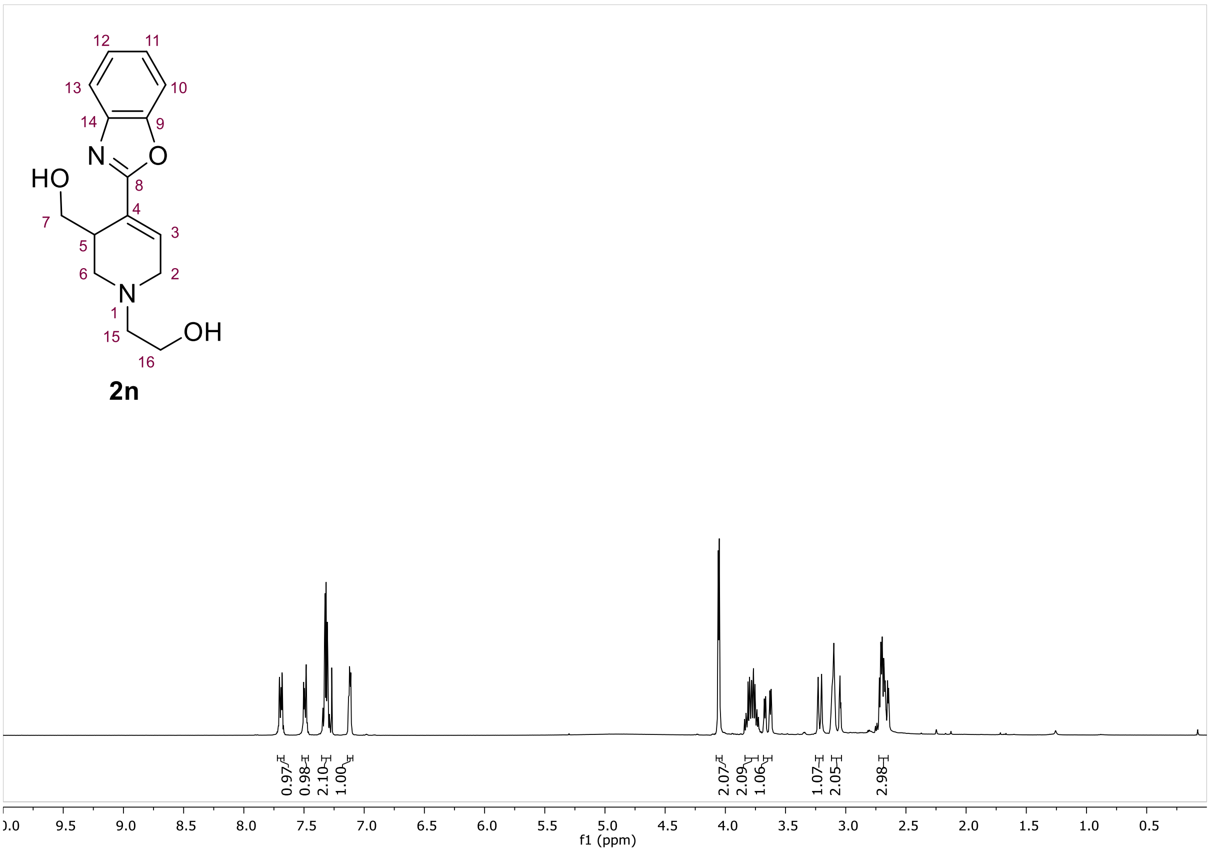
12
11
13
10
14
9
N
O
HO
8
7
4
3
5
6
2
N
1
15
OH
16
2n
0.97
0.98
2.10
1.00
2.07
2.09
1.06
1.07
2.05
2.98
9.5
9.0
8.5
8.0
7.5
7.0
6.5
6.0
5.5
5.0
4.5
4.0
3.5
3.0
2.5
2.0
1.5
1.0
0.5
f1 (ppm)

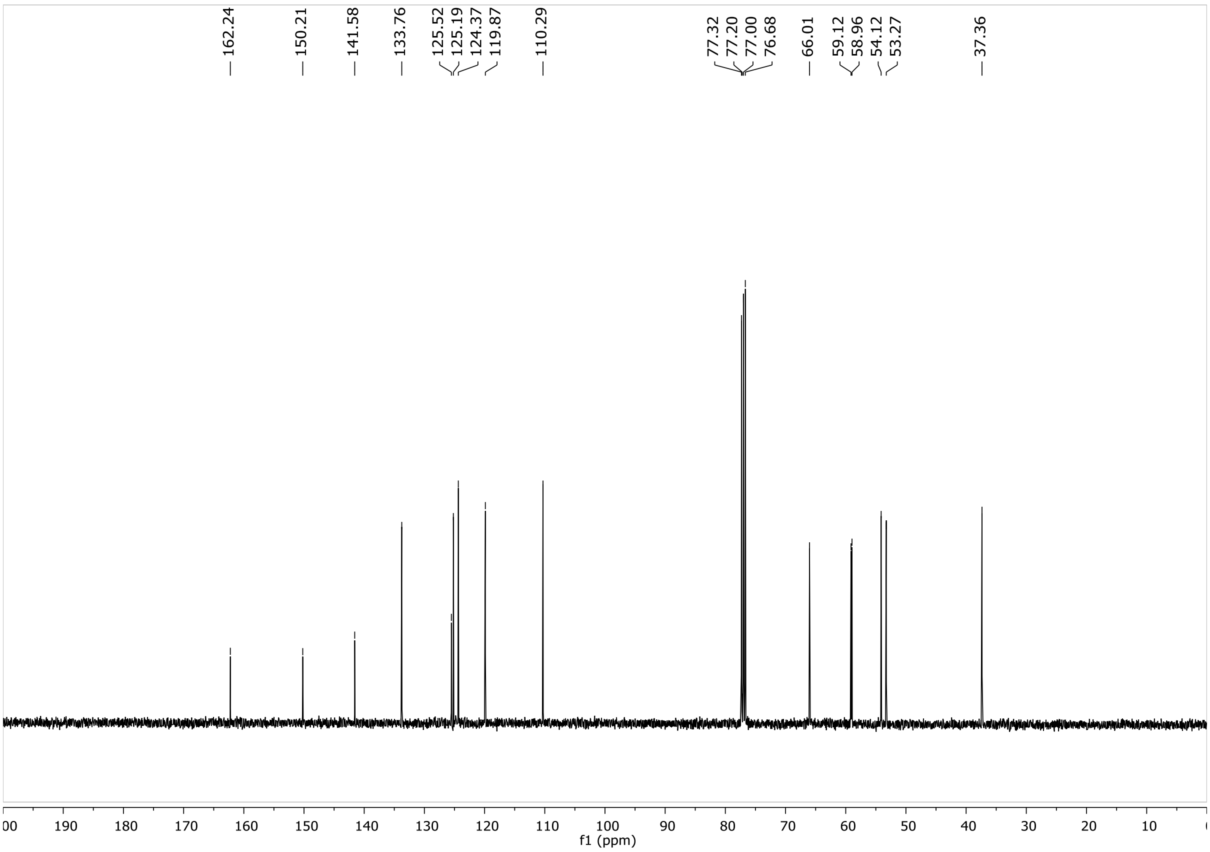
162.24
150.21
141.58
133.76
125.52
125.19
124.37
119.87
110.29
77.32
77.20
77.00
76.68
66.01
59.12
58.96
54.12
53.27
37.36
190
180
170
160
150
140
130
120
110
100
90
80
70
60
50
40
30
20
10
f1 (ppm)

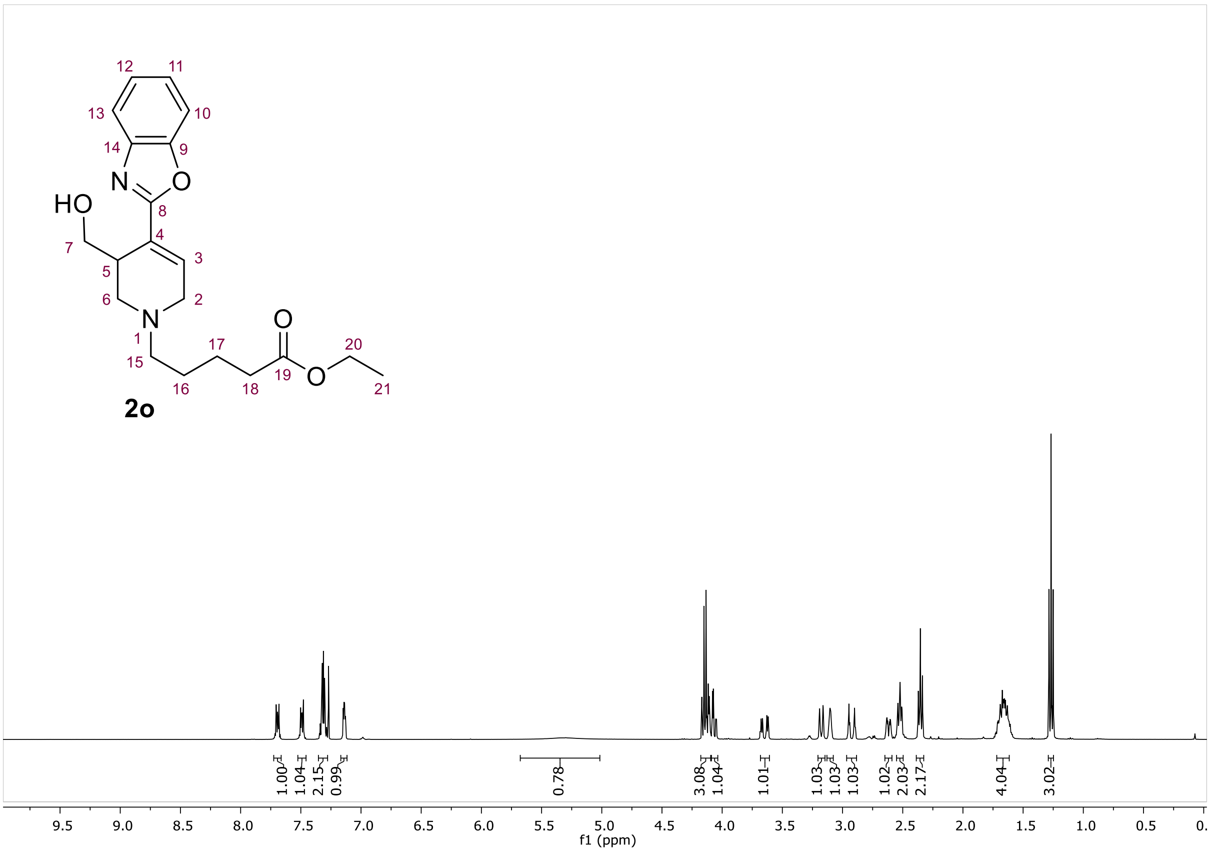

HO
N
O
O
O
N
1
2
3
4
5
6
7
8
9
10
11
12
13
14
15
16
17
18
19
20
21
2o
1.00
1.04
2.15
0.99
0.78
3.08
1.04
1.01
1.03
1.03
1.03
1.02
2.03
2.17
4.04
3.02
9.5 9.0 8.5 8.0 7.5 7.0 6.5 6.0 5.5 5.0 4.5 4.0 3.5 3.0 2.5 2.0 1.5 1.0 0.5
f1 (ppm)

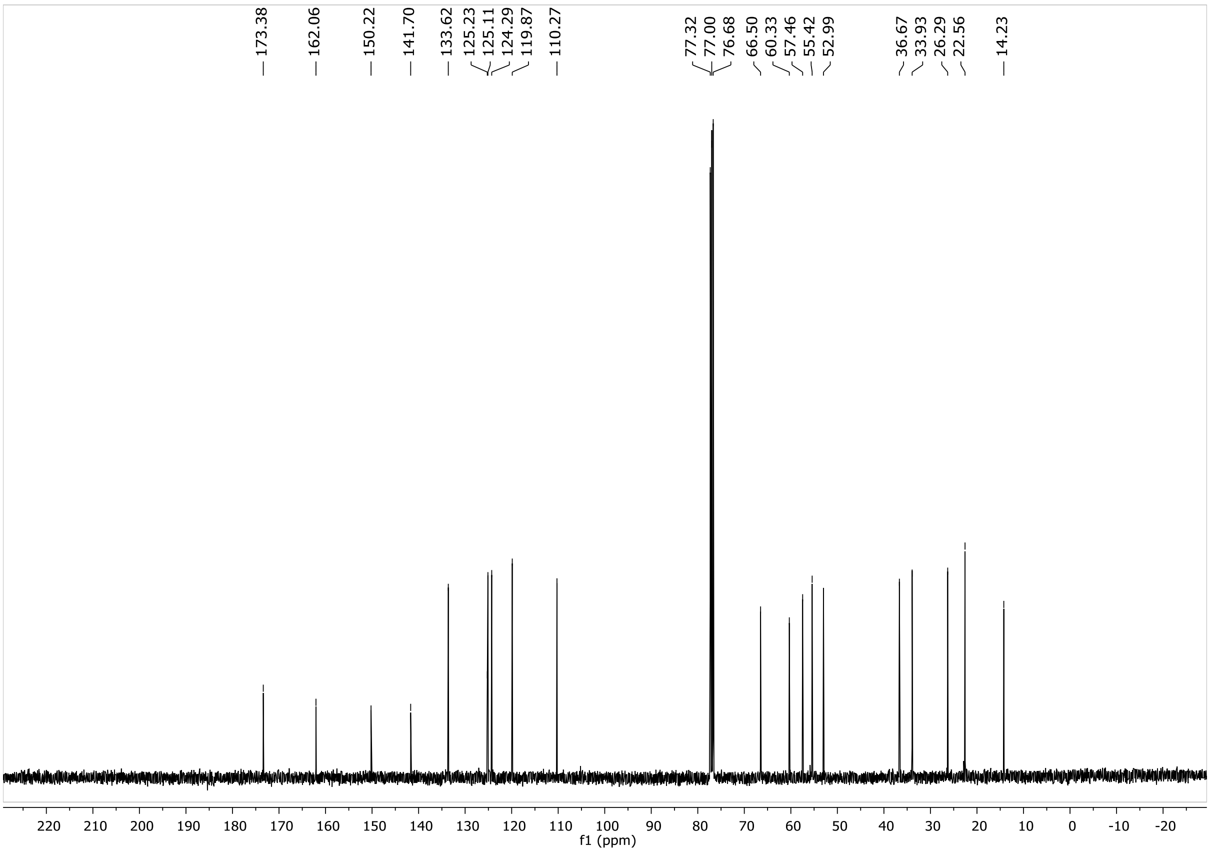

173.38
162.06
150.22
141.70
133.62
125.23
125.11
124.29
119.87
110.27
77.32
77.00
76.68
66.50
60.33
57.46
55.42
52.99
36.67
33.93
26.29
22.56
14.23
220 210 200 190 180 170 160 150 140 130 120 110 100 90 80 70 60 50 40 30 20 10 0 -10 -20
f1 (ppm)

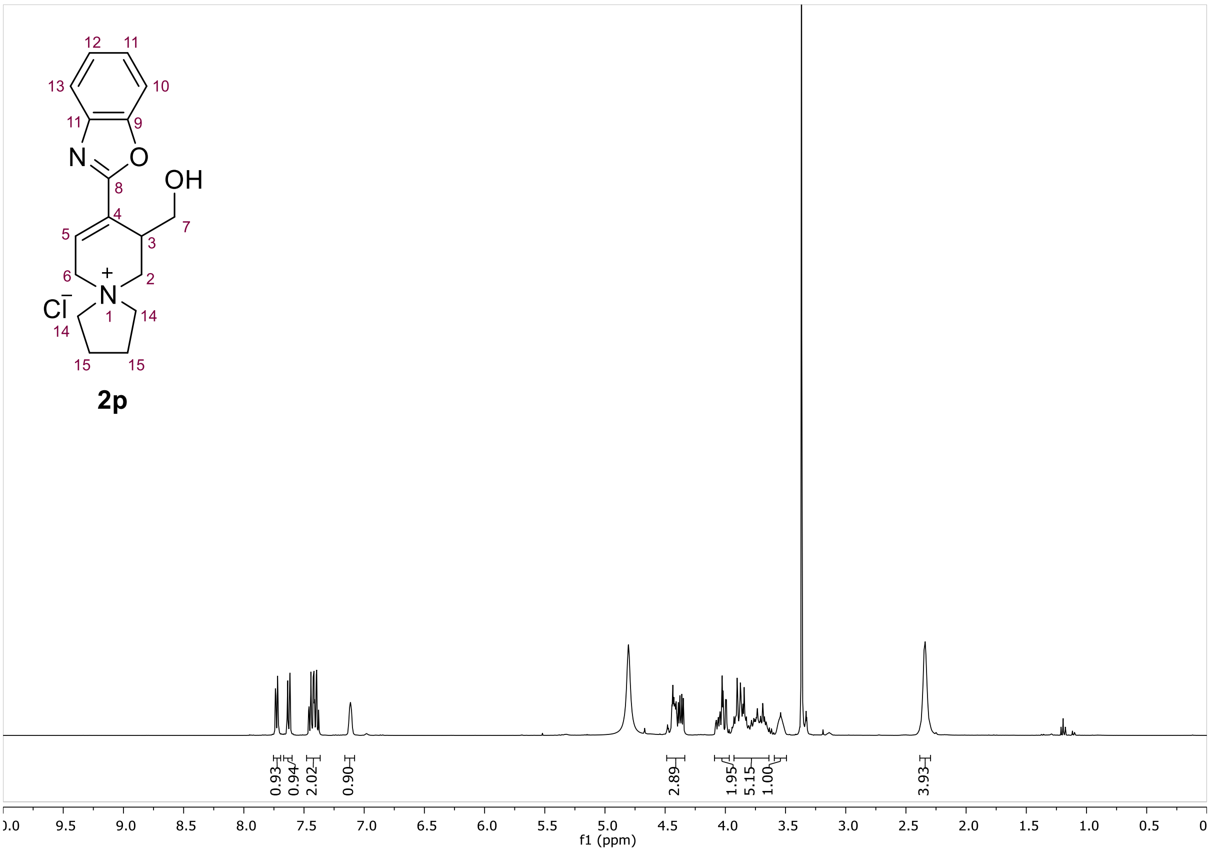
12
11
13
10
11
9
N
O
OH
8
4
5
3
7
6
2
N
+
Cl
1
14
14
15
15
2p
0.93
0.94
2.02
0.90
2.89
1.95
5.15
1.00
3.93
9.5
9.0
8.5
8.0
7.5
7.0
6.5
6.0
5.5
5.0
4.5
4.0
3.5
3.0
2.5
2.0
1.5
1.0
0.5
f1 (ppm)

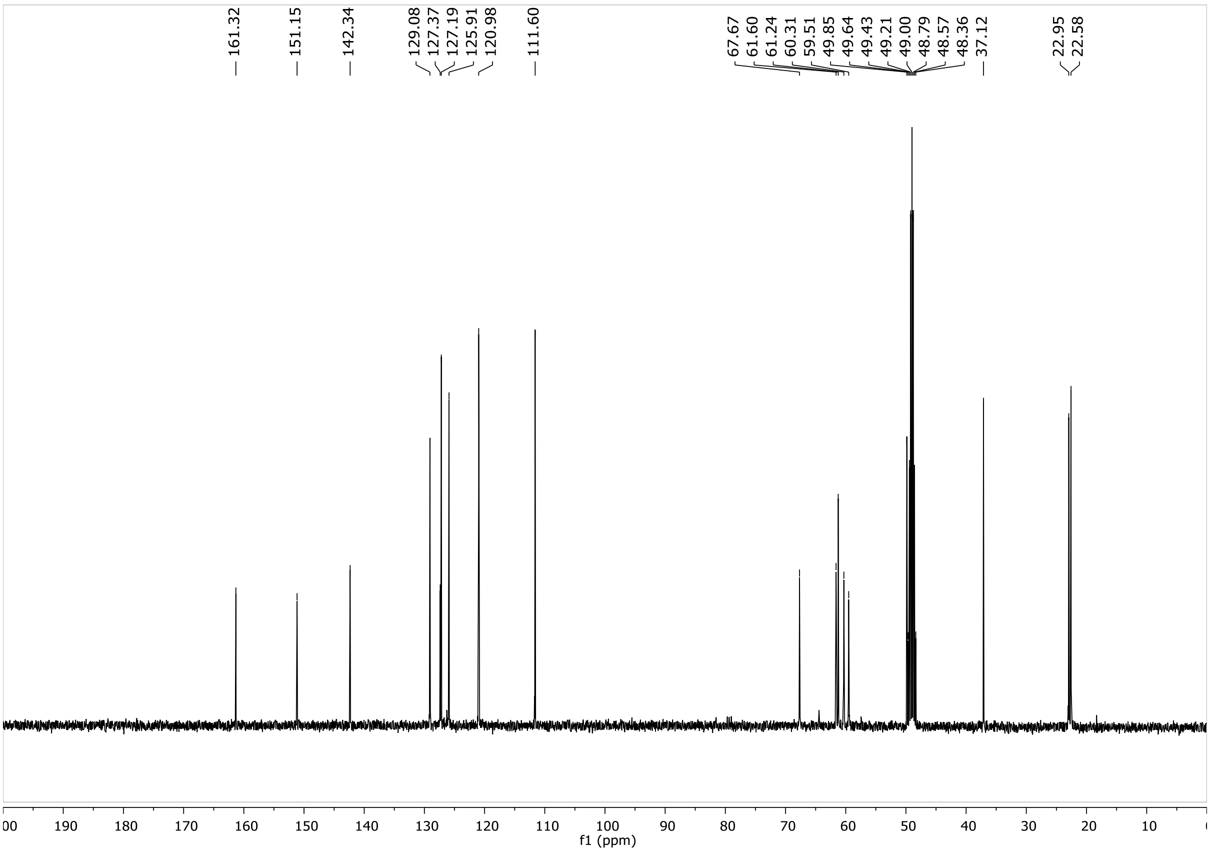
161.32
151.15
142.34
129.08
127.37
127.19
125.91
120.98
111.60
67.67
61.60
61.24
60.31
59.51
49.85
49.64
49.43
49.21
49.00
48.79
48.57
48.36
37.12
22.95
22.58
190
180
170
160
150
140
130
120
110
100
90
80
70
60
50
40
30
20
10
f1 (ppm)

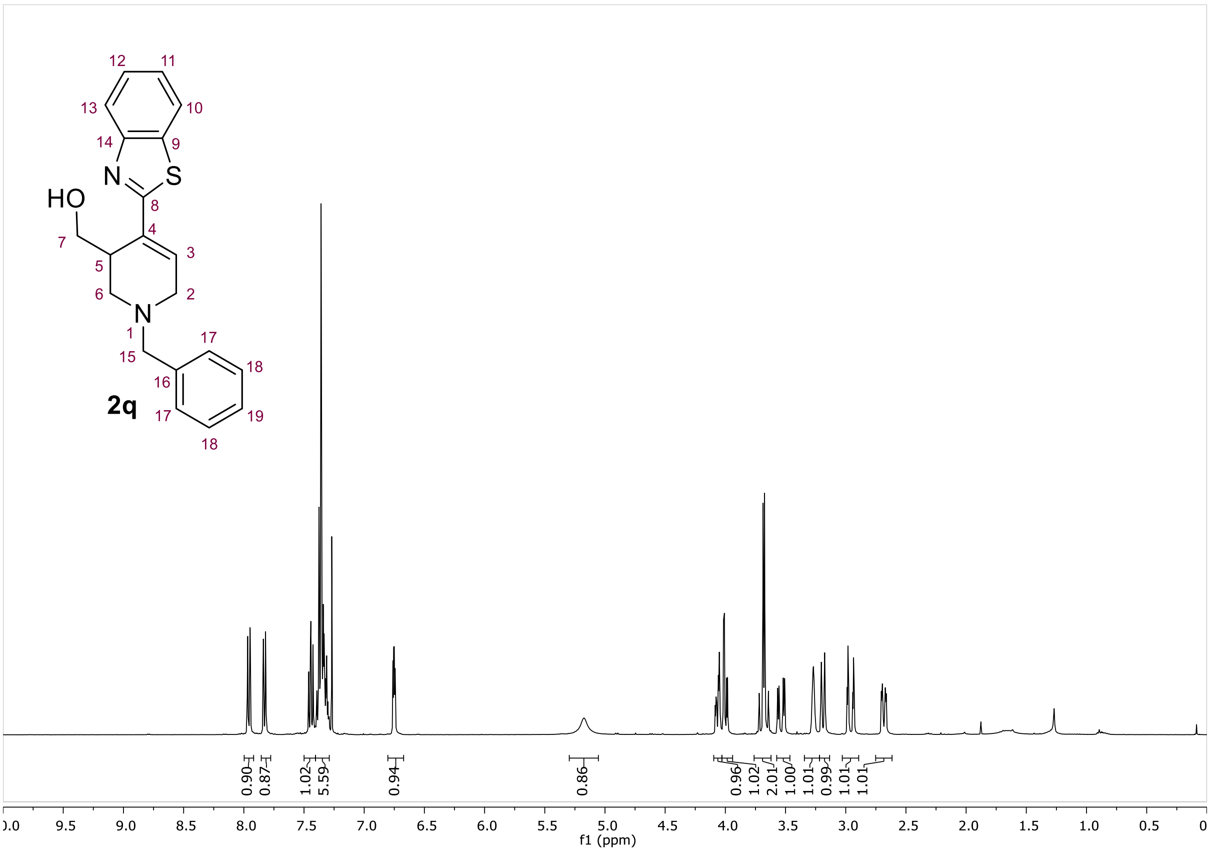
2q
HO
N
S
0.90
0.87
1.02
5.59
0.94
0.86
0.96
1.02
2.01
1.00
1.01
0.99
1.01
1.01
f1 (ppm)

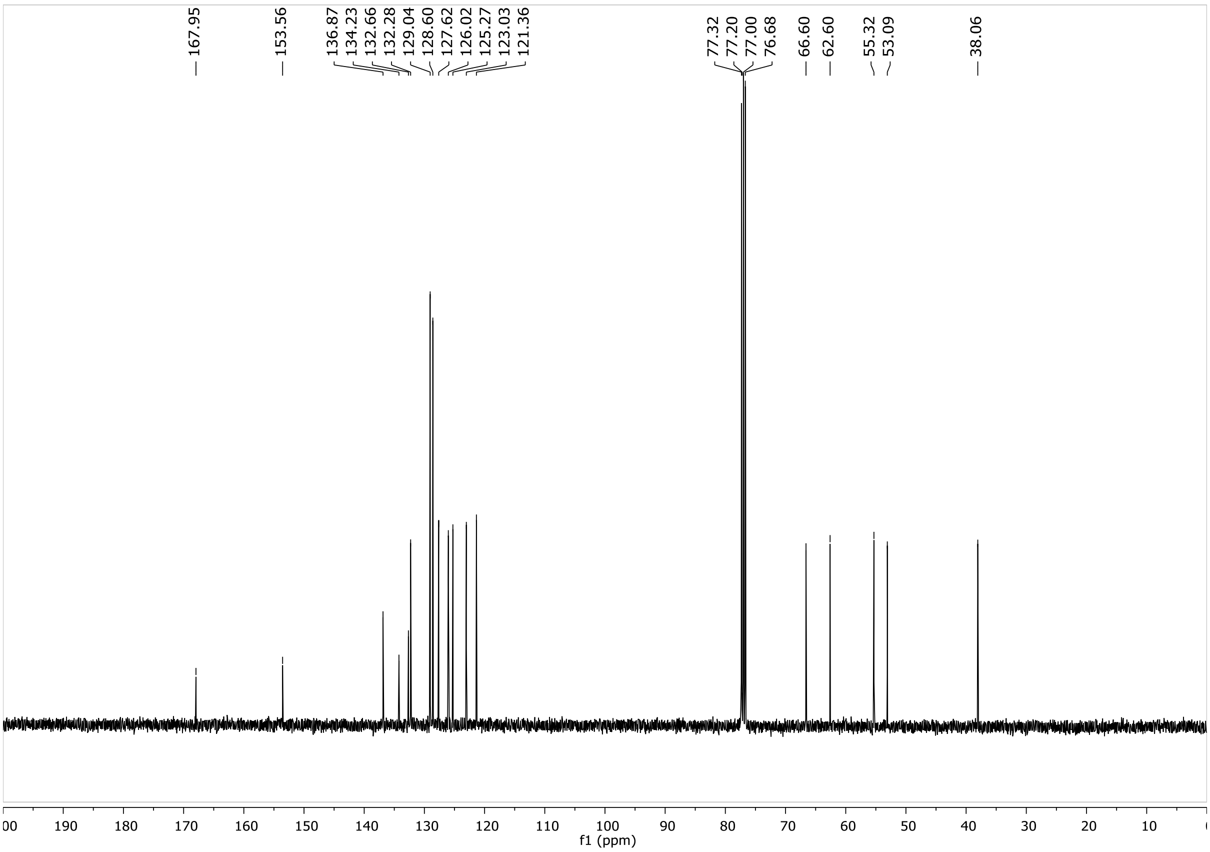
167.95
153.56
136.87
134.23
132.66
132.28
129.04
128.60
127.62
126.02
125.27
123.03
121.36
77.32
77.20
77.00
76.68
66.60
62.60
55.32
53.09
38.06
f1 (ppm)

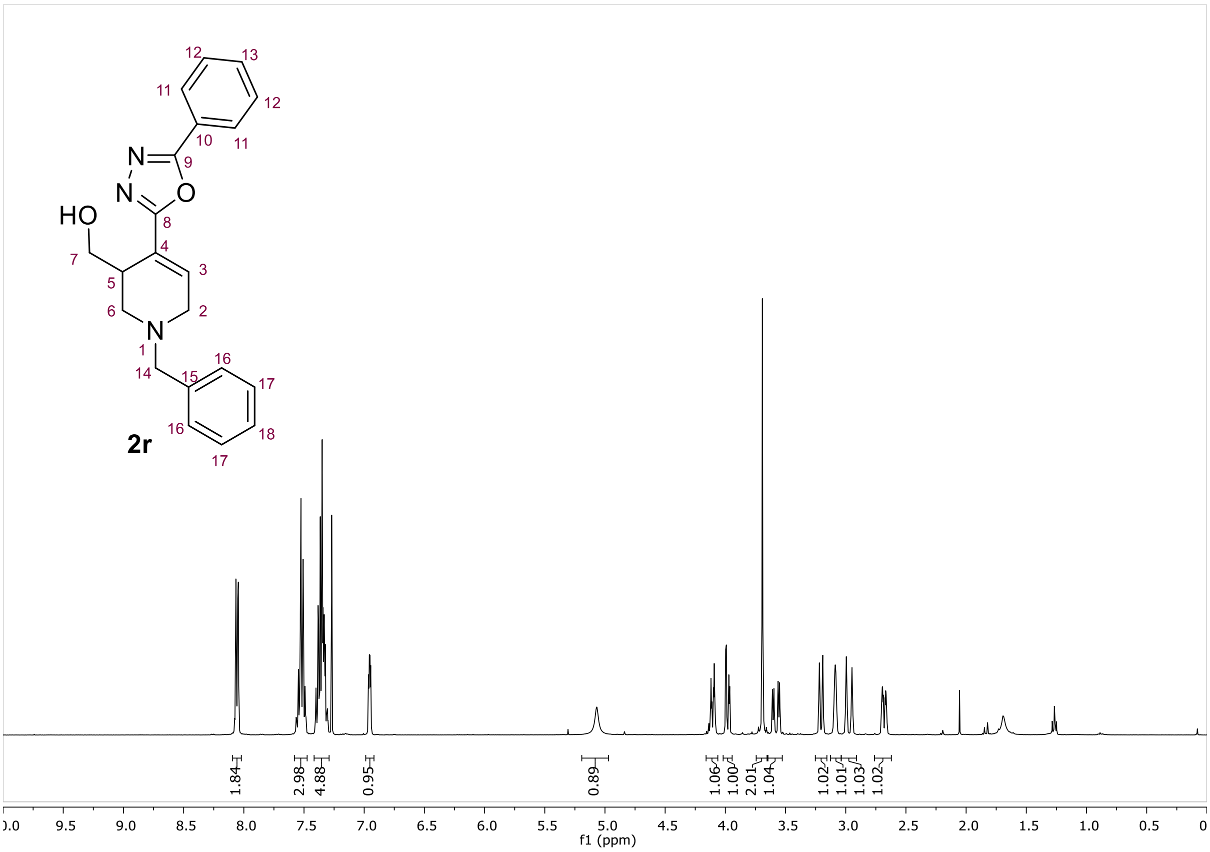
HO
N
N
O
2r
1.84
2.98
4.88
0.95
0.89
1.06
1.00
2.01
1.04
1.02
1.01
1.03
1.02
f1 (ppm)

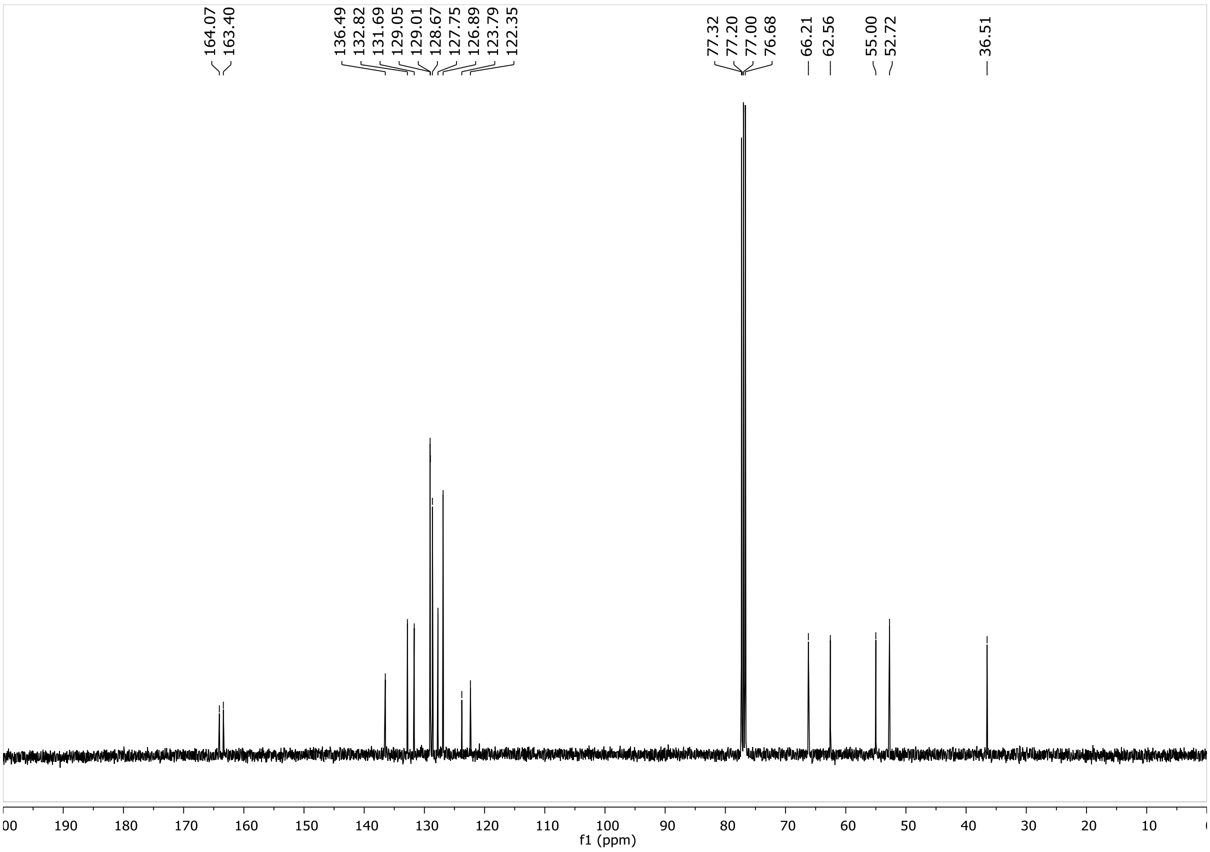
164.07
163.40
136.49
132.82
131.69
129.05
129.01
128.67
127.75
126.89
123.79
122.35
77.32
77.20
77.00
76.68
66.21
62.56
55.00
52.72
36.51
f1 (ppm)

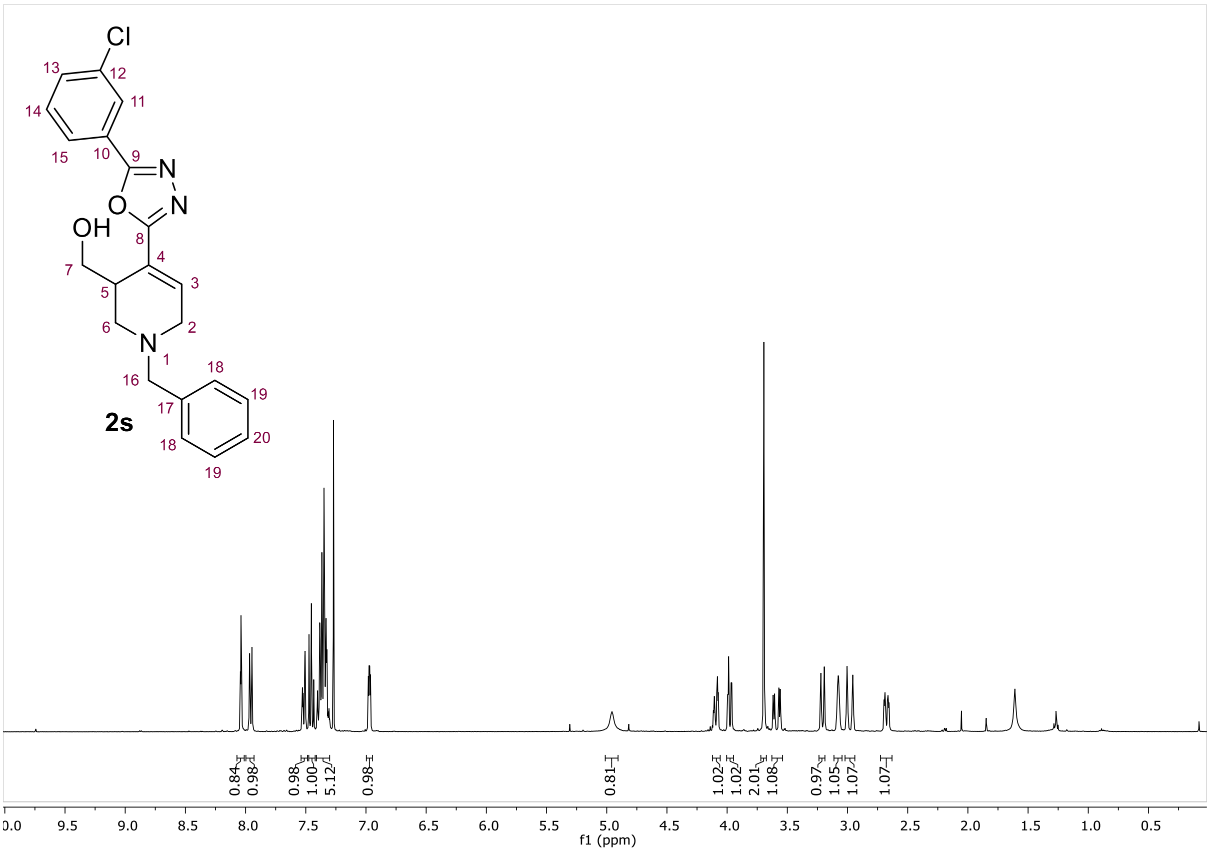

Cl
2s
0.84
0.98
0.98
1.00
5.12
0.98
0.81
1.02
1.02
2.01
1.08
0.97
1.05
1.07
1.07
f1 (ppm)

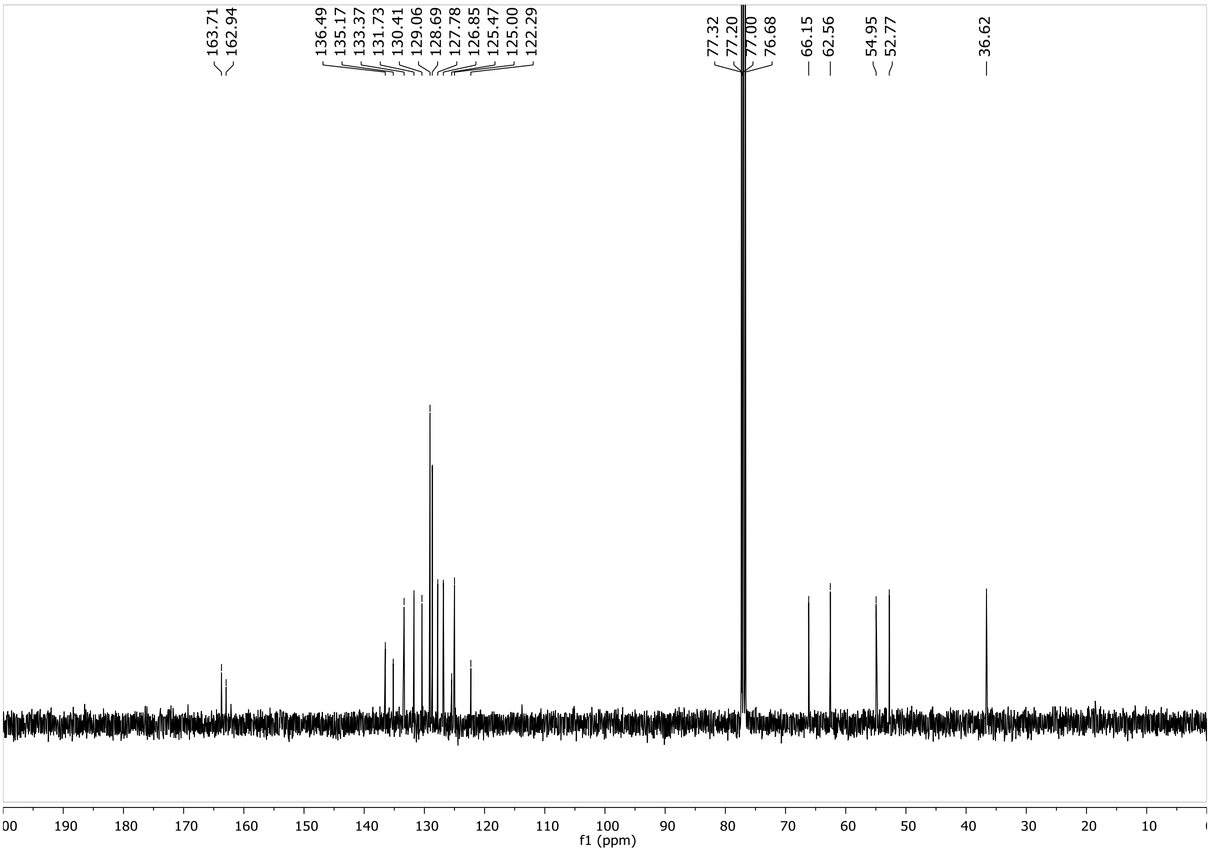

163.71
162.94
136.49
135.17
133.37
131.73
130.41
129.06
128.69
127.78
126.85
125.47
125.00
122.29
77.32
77.20
77.00
76.68
66.15
62.56
54.95
52.77
36.62
f1 (ppm)

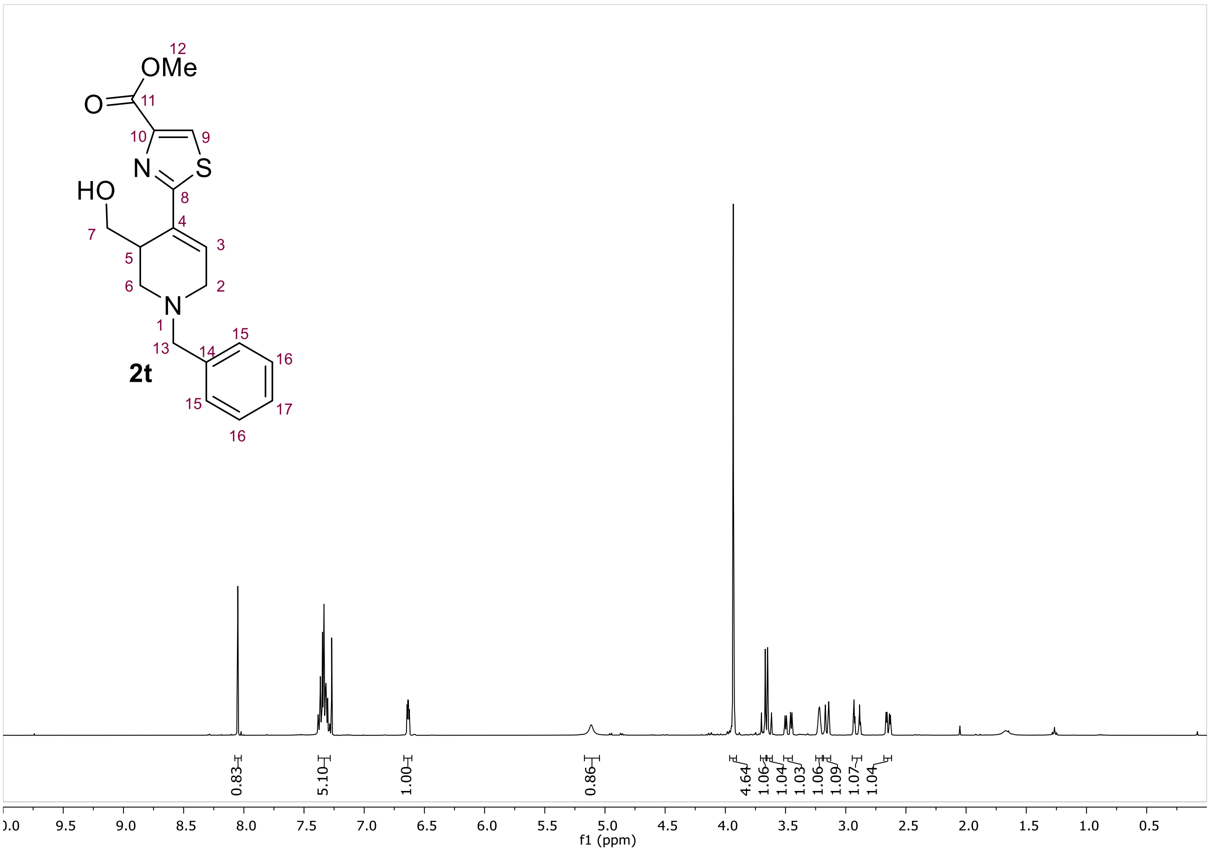

12
OMe
O
11
10
9
N
S
8
HO
4
7
3
5
6
2
N
1
13
14
15
16
2t
15
17
16
0.83
5.10
1.00
0.86
4.64
1.06
1.04
1.03
1.06
1.09
1.07
1.04
9.5
9.0
8.5
8.0
7.5
7.0
6.5
6.0
5.5
5.0
4.5
4.0
3.5
3.0
2.5
2.0
1.5
1.0
0.5
f1 (ppm)

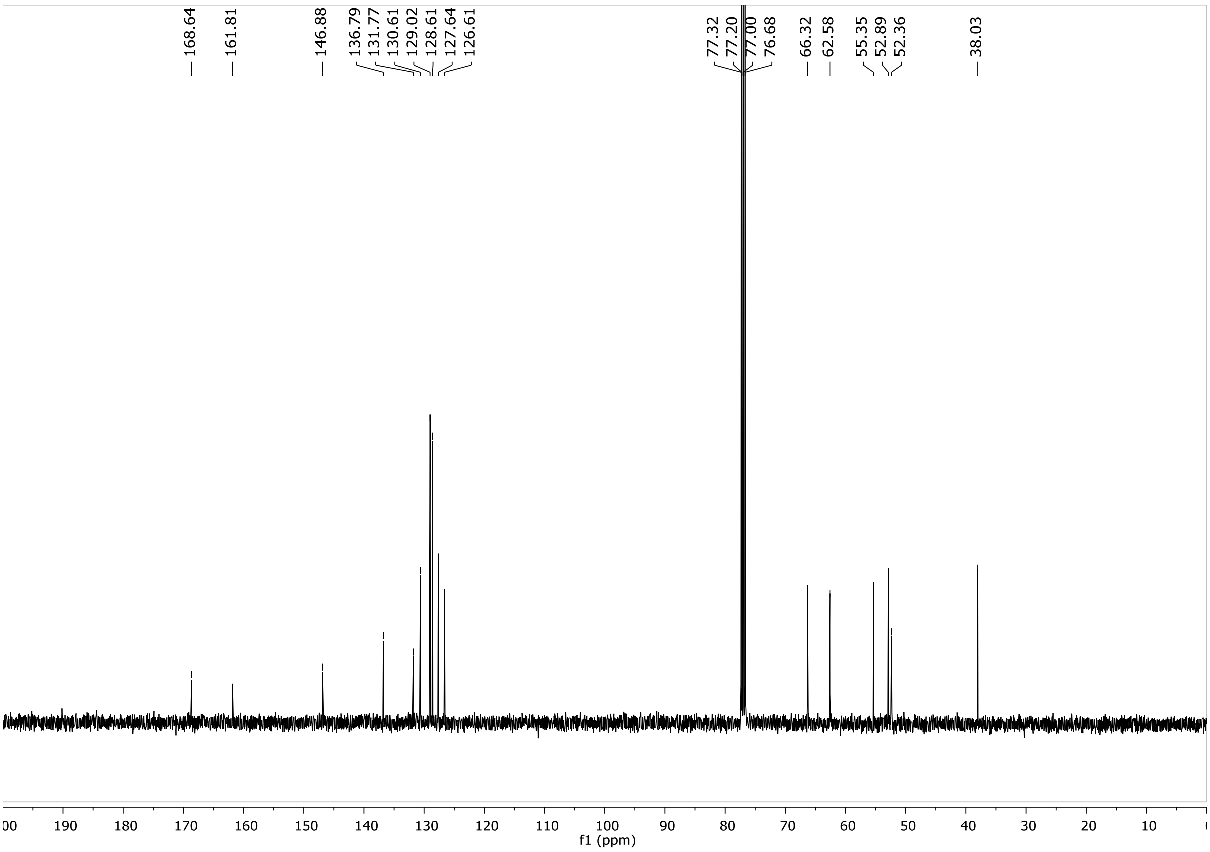

168.64
161.81
146.88
136.79
131.77
130.61
129.02
128.61
127.64
126.61
77.32
77.20
77.00
76.68
66.32
62.58
55.35
52.89
52.36
38.03
190
180
170
160
150
140
130
120
110
100
90
80
70
60
50
40
30
20
10
f1 (ppm)

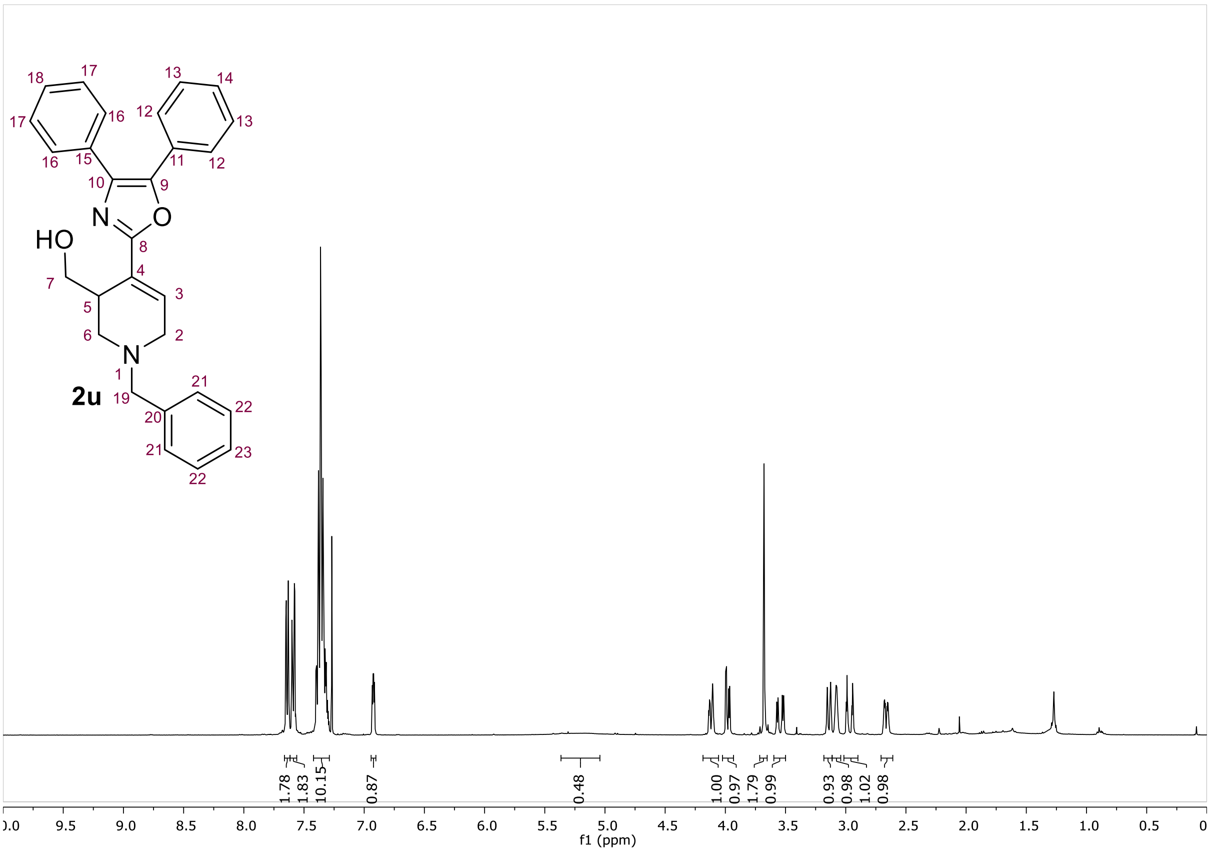
2u
HO
N
O
1.78
1.83
10.15
0.87
0.48
1.00
0.97
1.79
0.99
0.93
0.98
1.02
0.98
f1 (ppm)

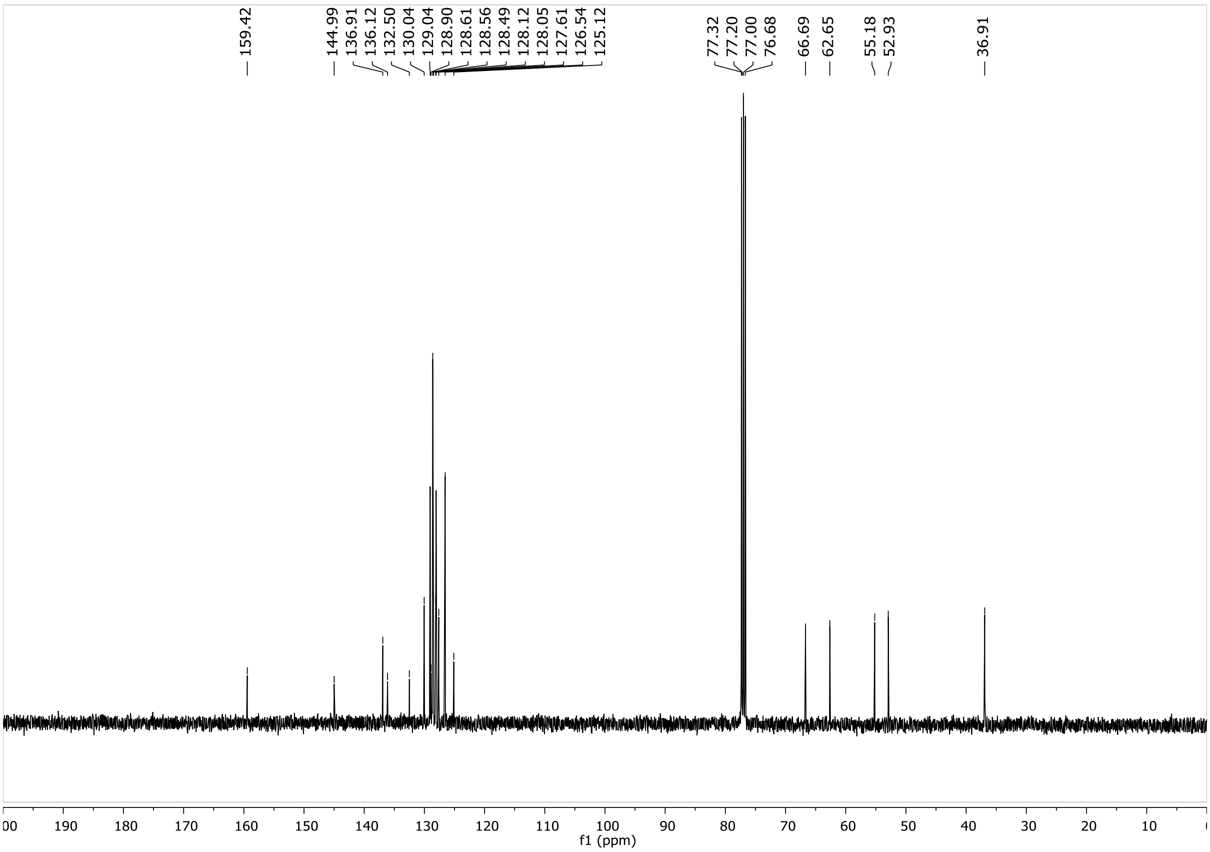
159.42
144.99
136.91
136.12
132.50
130.04
129.04
128.90
128.61
128.56
128.49
128.12
128.05
127.61
126.54
125.12
77.32
77.20
77.00
76.68
66.69
62.65
55.18
52.93
36.91
f1 (ppm)

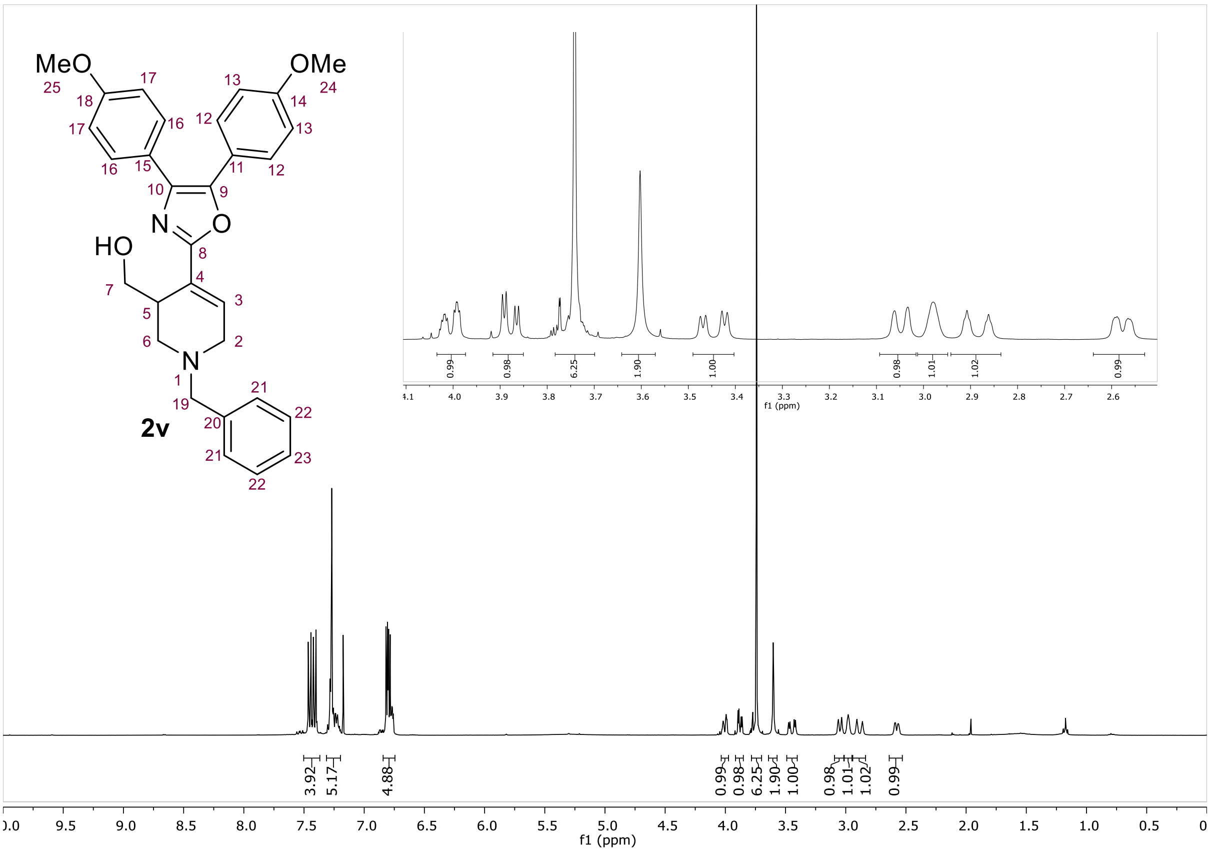
MeO
OMe
HO
N
O
2v
0.99 0.98 6.25 1.90 1.00 0.98 1.01 1.02 0.99
f1 (ppm)
3.92 5.17 4.88 0.99 0.98 6.25 1.90 1.00 0.98 1.01 1.02 0.99
f1 (ppm)

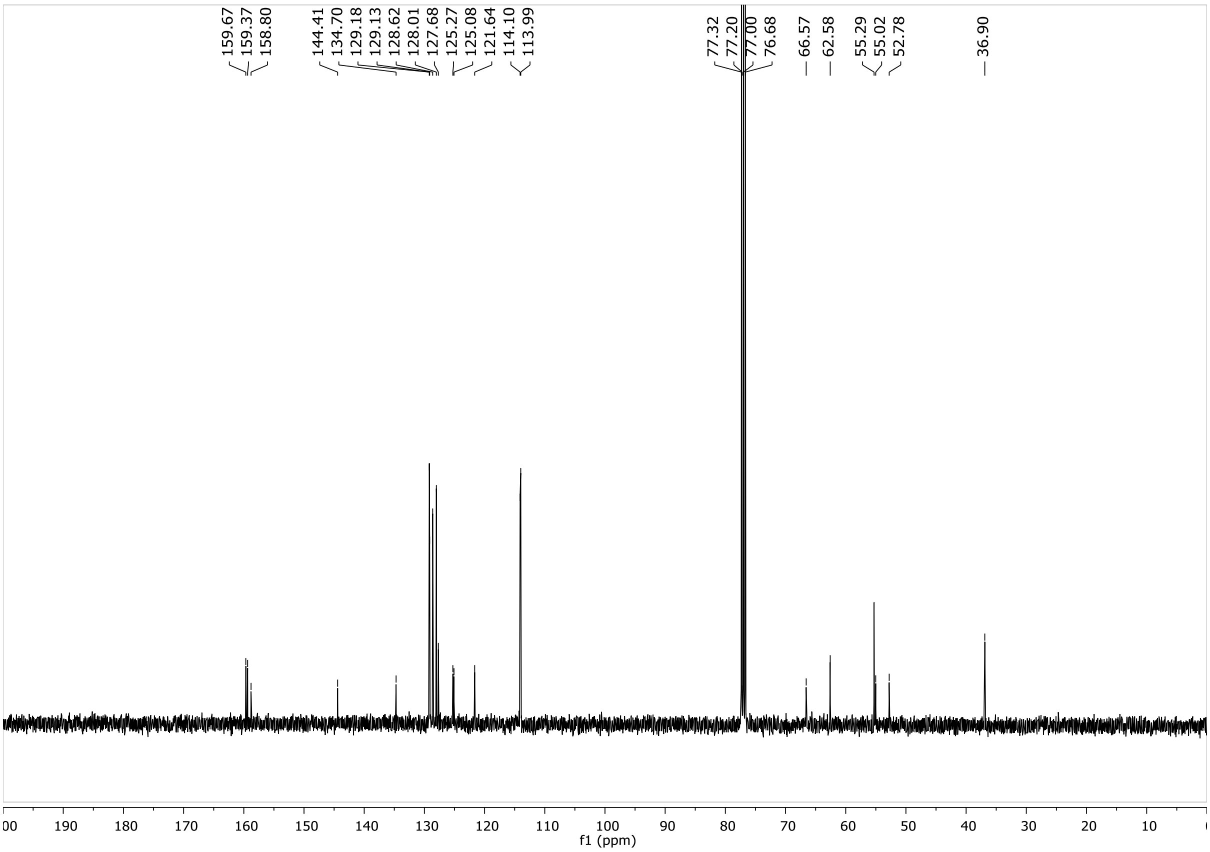
159.67 159.37 158.80 144.41 134.70 129.18 129.13 128.62 128.01 127.68 125.27 125.08 121.64 114.10 113.99
77.32 77.20 77.00 76.68 66.57 62.58 55.29 55.02 52.78 36.90
f1 (ppm)

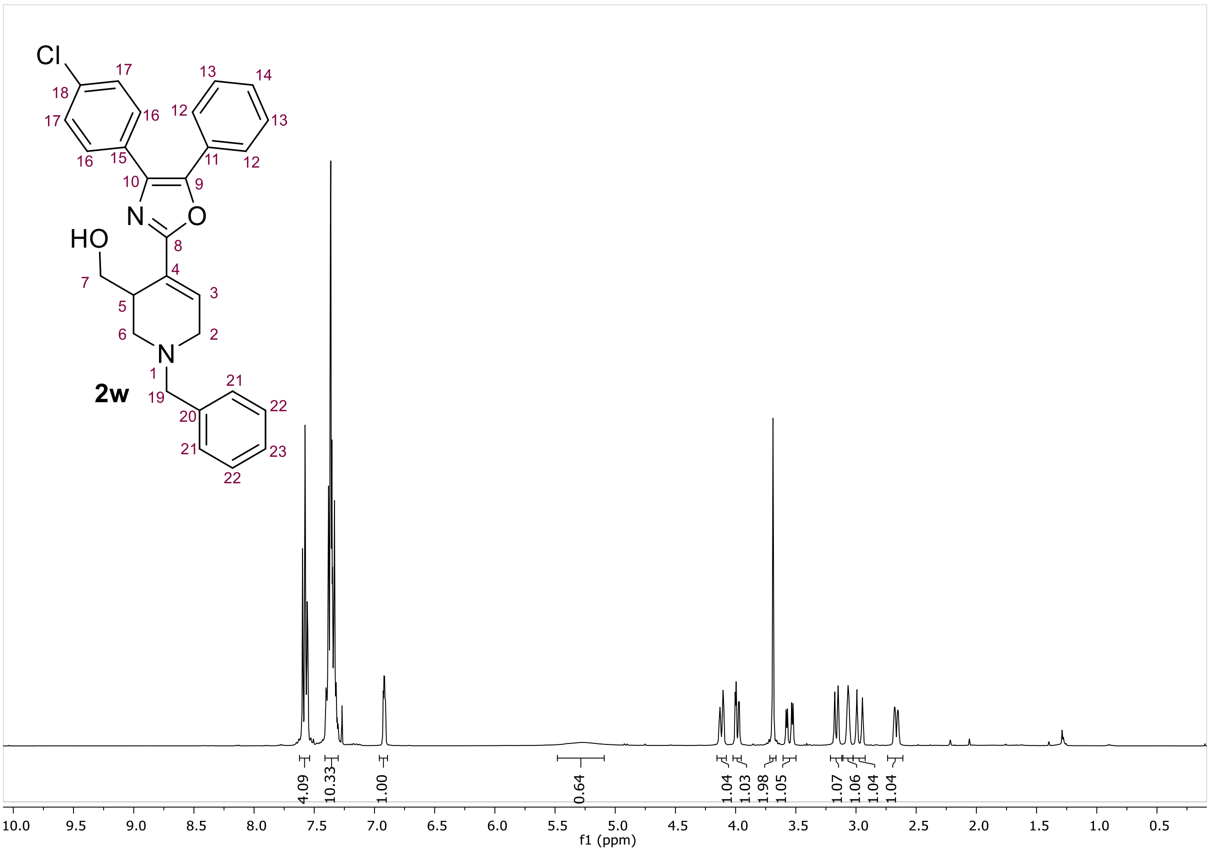
Cl
HO
N
O
N
2w
4.09
10.33
1.00
0.64
1.04
1.03
1.98
1.05
1.07
1.06
1.04
1.04
f1 (ppm)

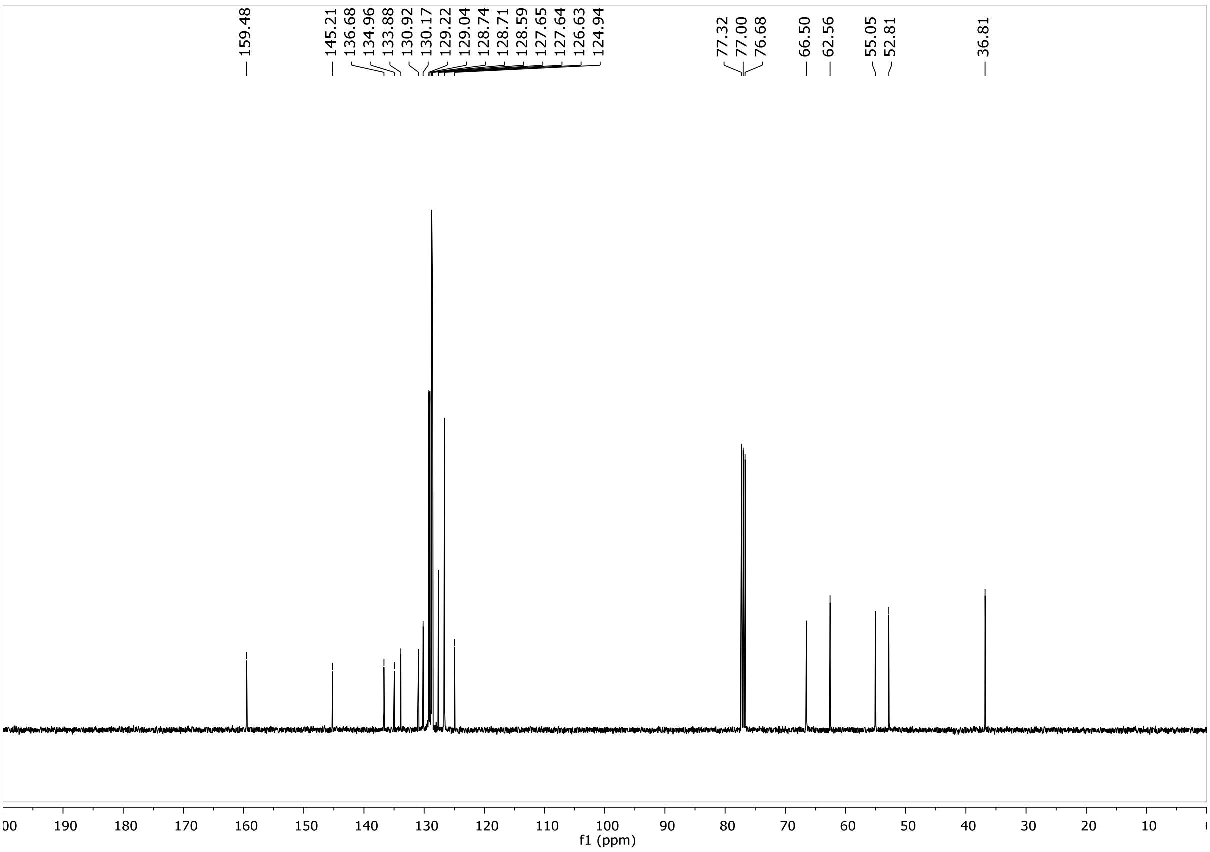
159.48
145.21
136.68
134.96
133.88
130.92
130.17
129.22
129.04
128.74
128.71
128.59
127.65
127.64
126.63
124.94
77.32
77.00
76.68
66.50
62.56
55.05
52.81
36.81
f1 (ppm)

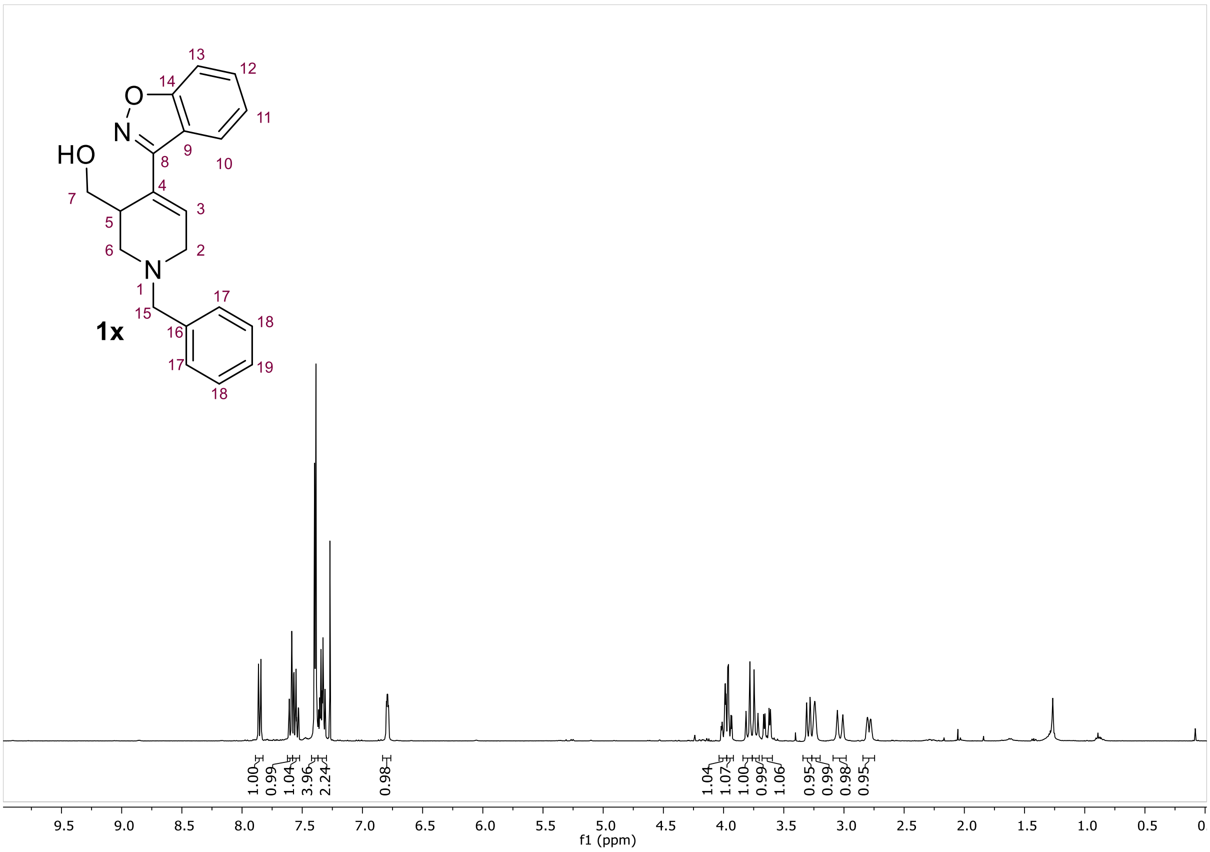
1x
f1 (ppm)

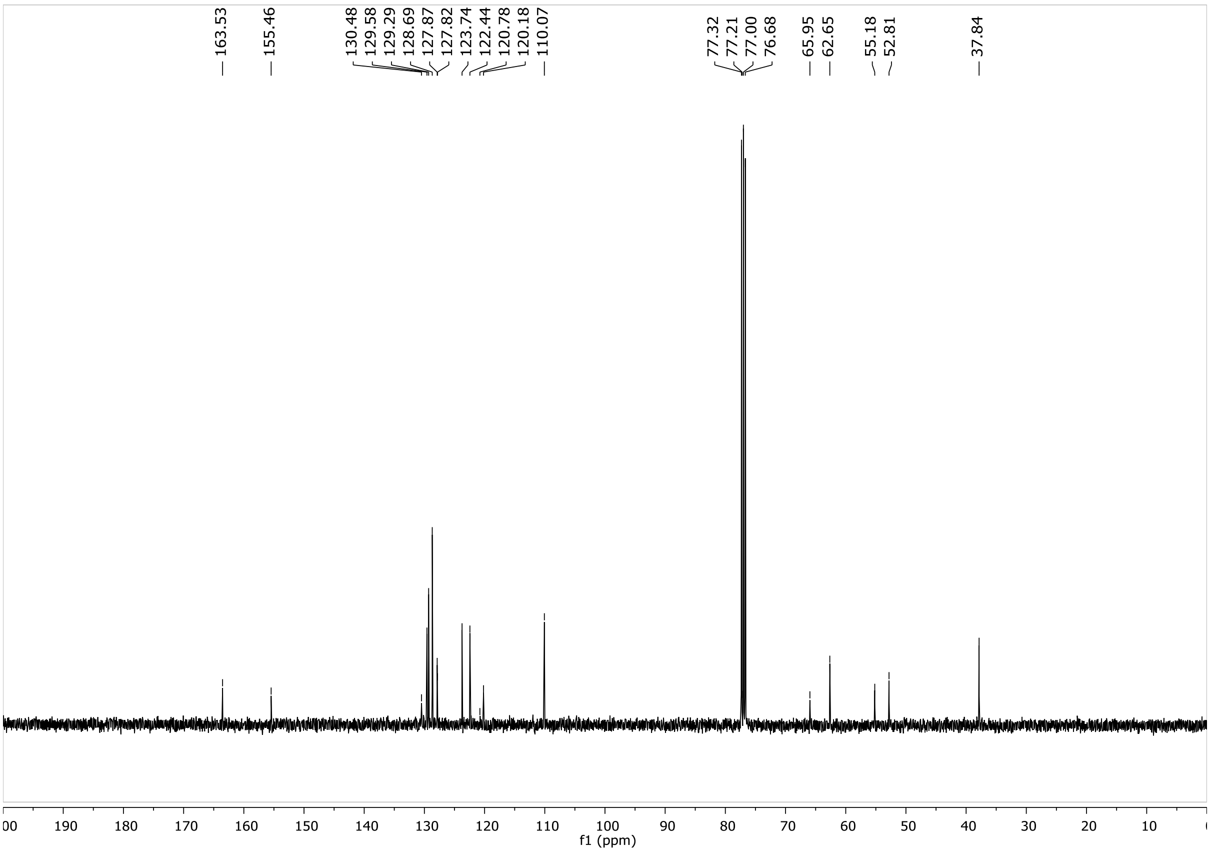
163.53
155.46
130.48
129.58
129.29
128.69
127.87
127.82
123.74
122.44
120.78
120.18
110.07
77.32
77.21
77.00
76.68
65.95
62.65
55.18
52.81
37.84
f1 (ppm)

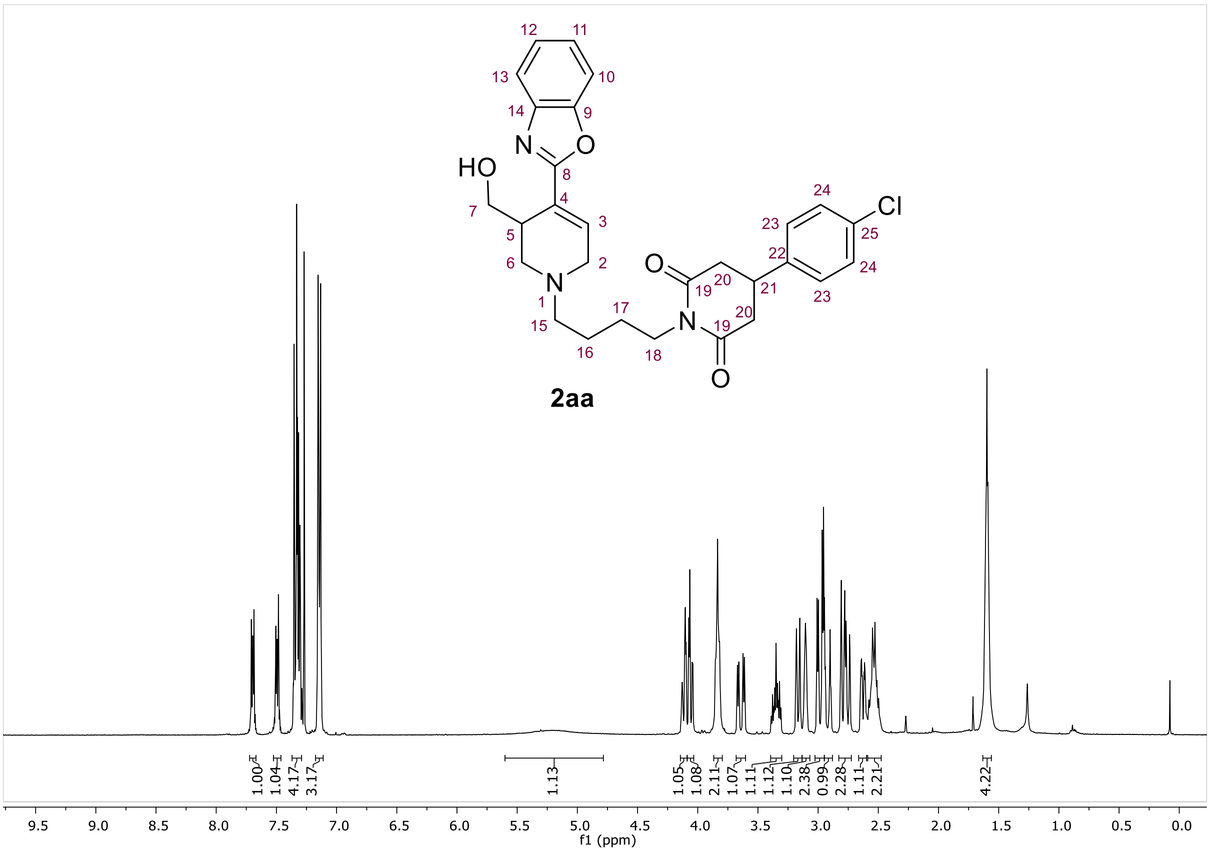

HO
N
O
Cl
O
O
2aa
1.00
1.04
4.17
3.17
1.13
1.05
1.08
2.11
1.07
1.11
1.12
1.10
2.38
0.99
2.28
1.11
2.21
4.22
f1 (ppm)

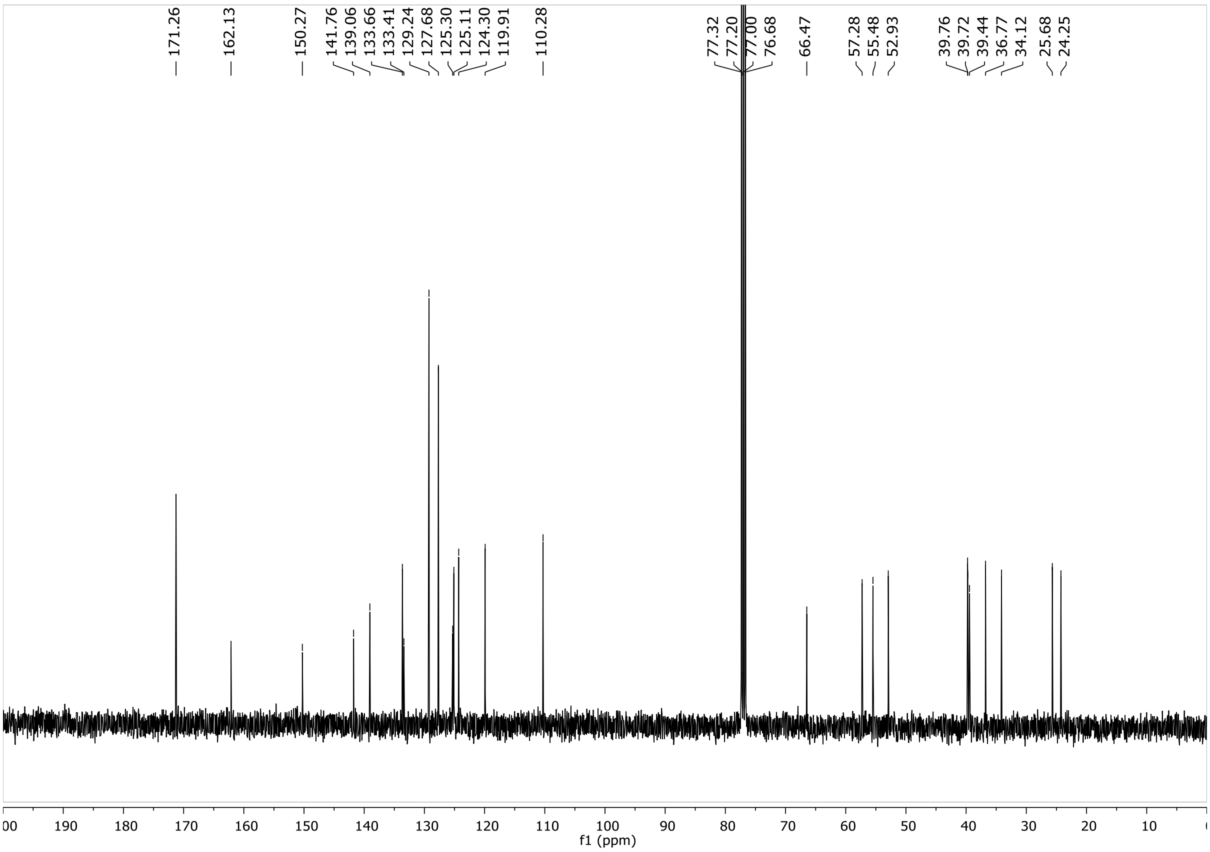

171.26
162.13
150.27
141.76
139.06
133.66
133.41
129.24
127.68
125.30
125.11
124.30
119.91
110.28
77.32
77.20
77.00
76.68
66.47
57.28
55.48
52.93
39.76
39.72
39.44
36.77
34.12
25.68
24.25
f1 (ppm)

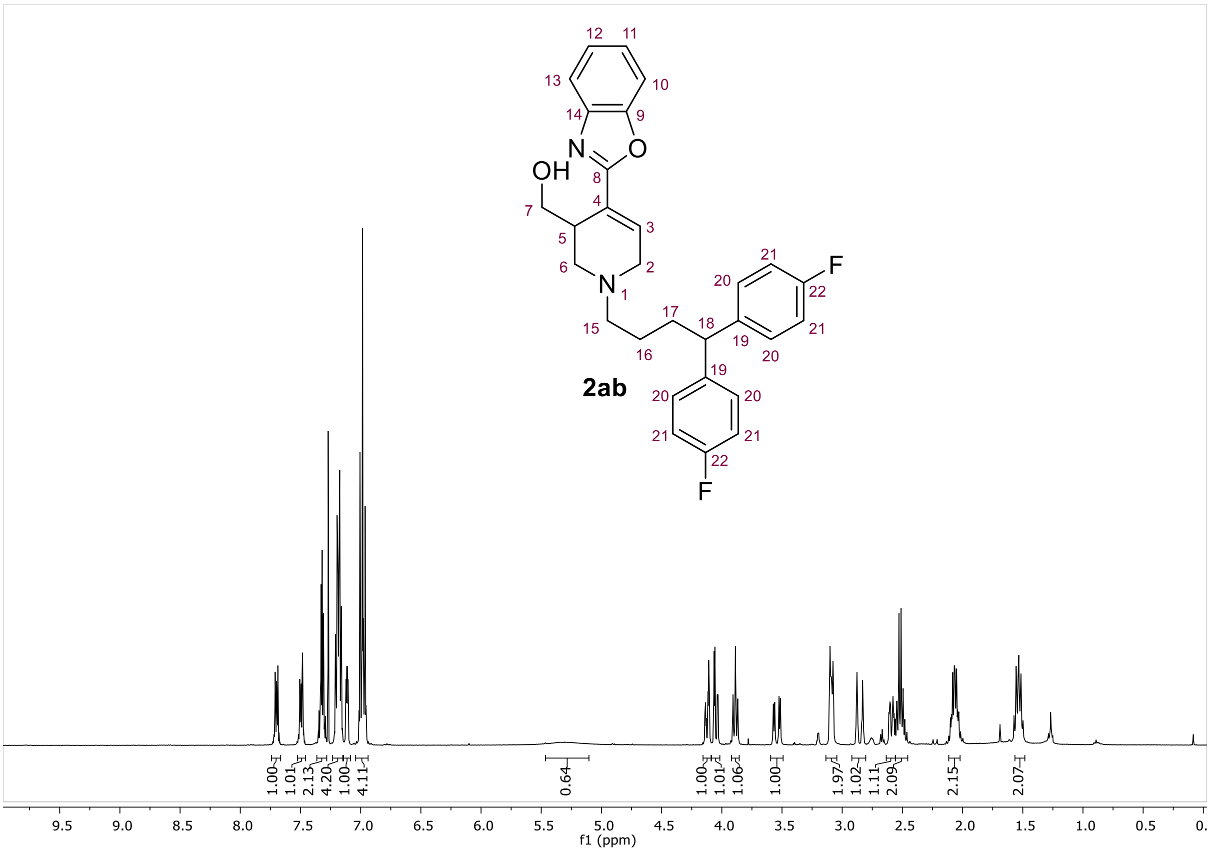
12
11
13
10
14
9
N
O
OH
8
7
4
3
5
6
2
N
1
21
F
20
22
17
18
15
16
19
21
20
2ab
19
20
20
21
21
22
F
1.00
1.01
2.13
4.20
1.00
4.11
0.64
1.00
1.01
1.06
1.00
1.97
1.02
1.11
2.09
2.15
2.07
9.5
9.0
8.5
8.0
7.5
7.0
6.5
6.0
5.5
5.0
4.5
4.0
3.5
3.0
2.5
2.0
1.5
1.0
0.5
f1 (ppm)

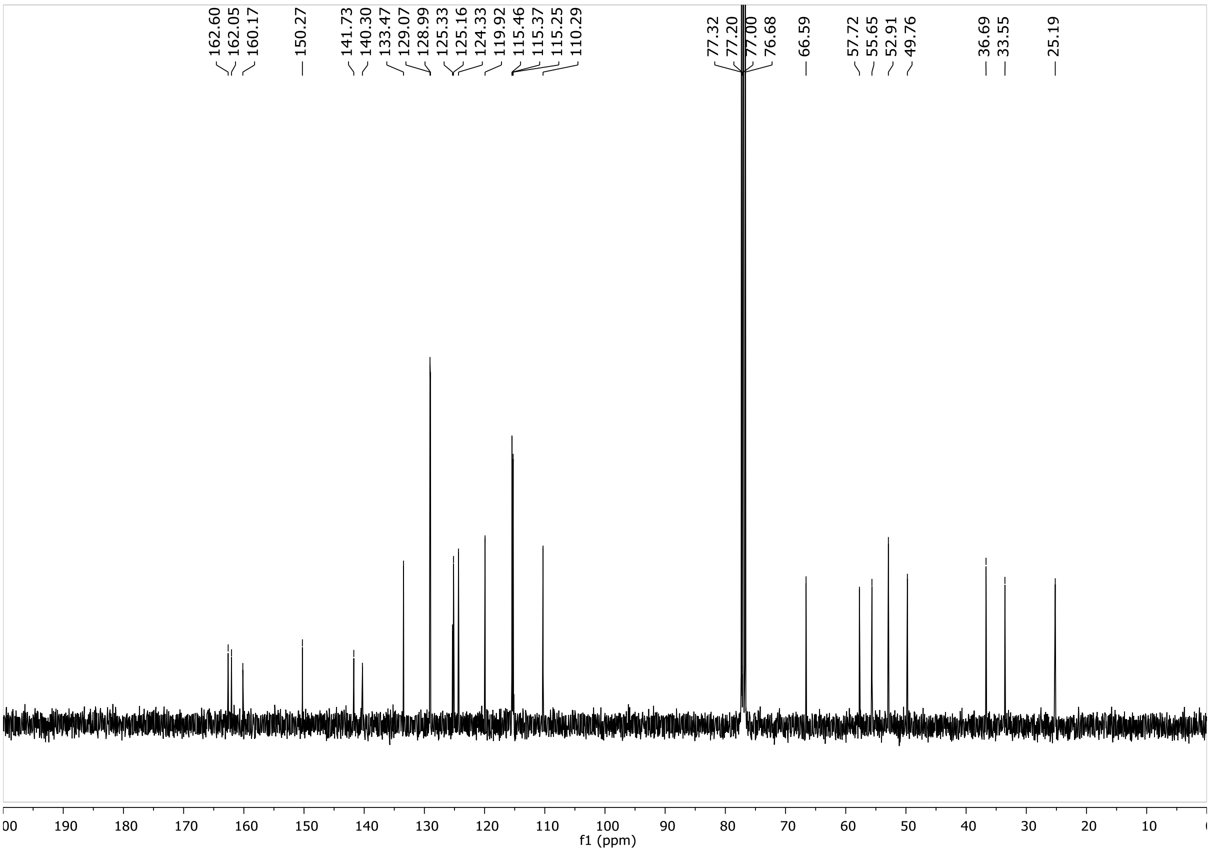
162.60
162.05
160.17
150.27
141.73
140.30
133.47
129.07
128.99
125.33
125.16
124.33
119.92
115.46
115.37
115.25
110.29
77.32
77.20
77.00
76.68
66.59
57.72
55.65
52.91
49.76
36.69
33.55
25.19
190
180
170
160
150
140
130
120
110
100
90
80
70
60
50
40
30
20
10
f1 (ppm)

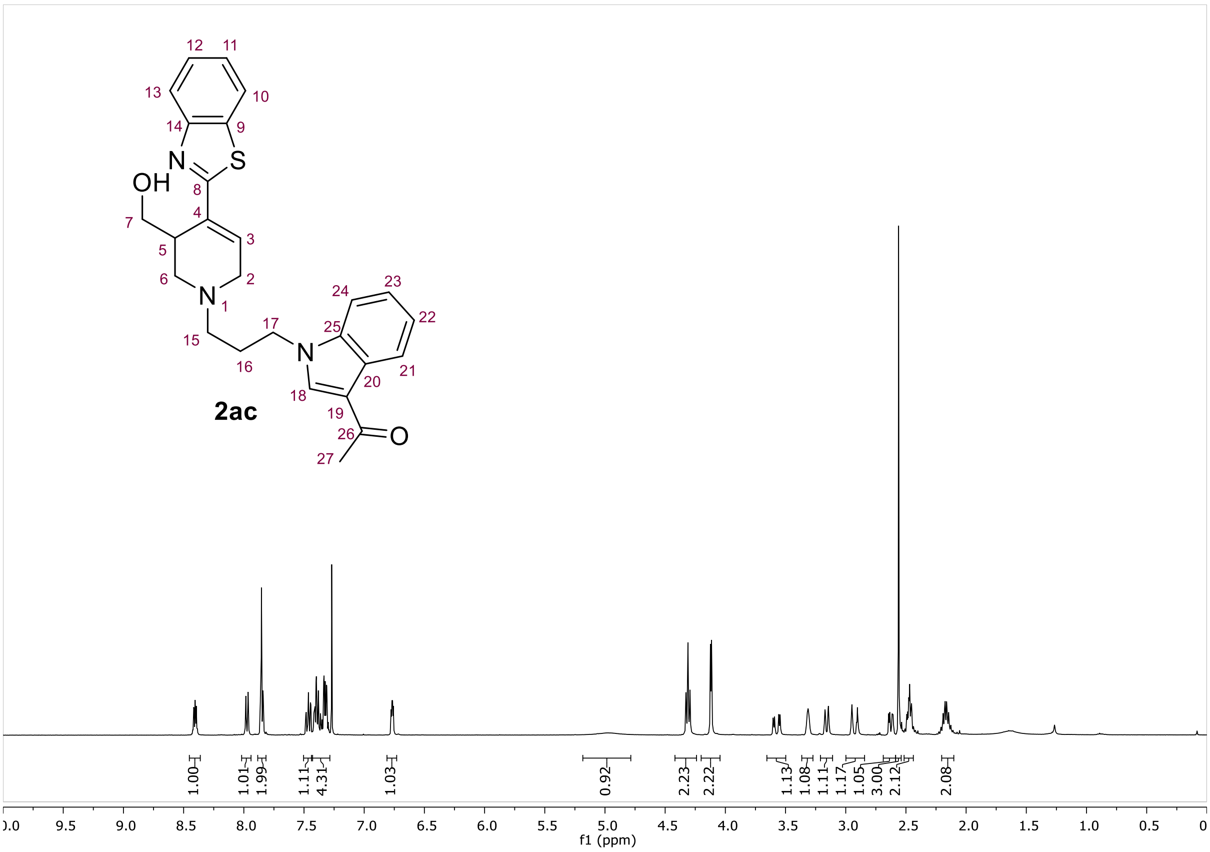
2ac
OH
N
S
N
N
O
12 11 10 13 14 9 8 7 4 5 3 6 2 1 15 16 17 24 23 22 25 21 20 18 19 26 27
1.00
1.01
1.99
1.11
4.31
1.03
0.92
2.23
2.22
1.13
1.08
1.11
1.17
1.05
3.00
2.12
2.08
f1 (ppm)

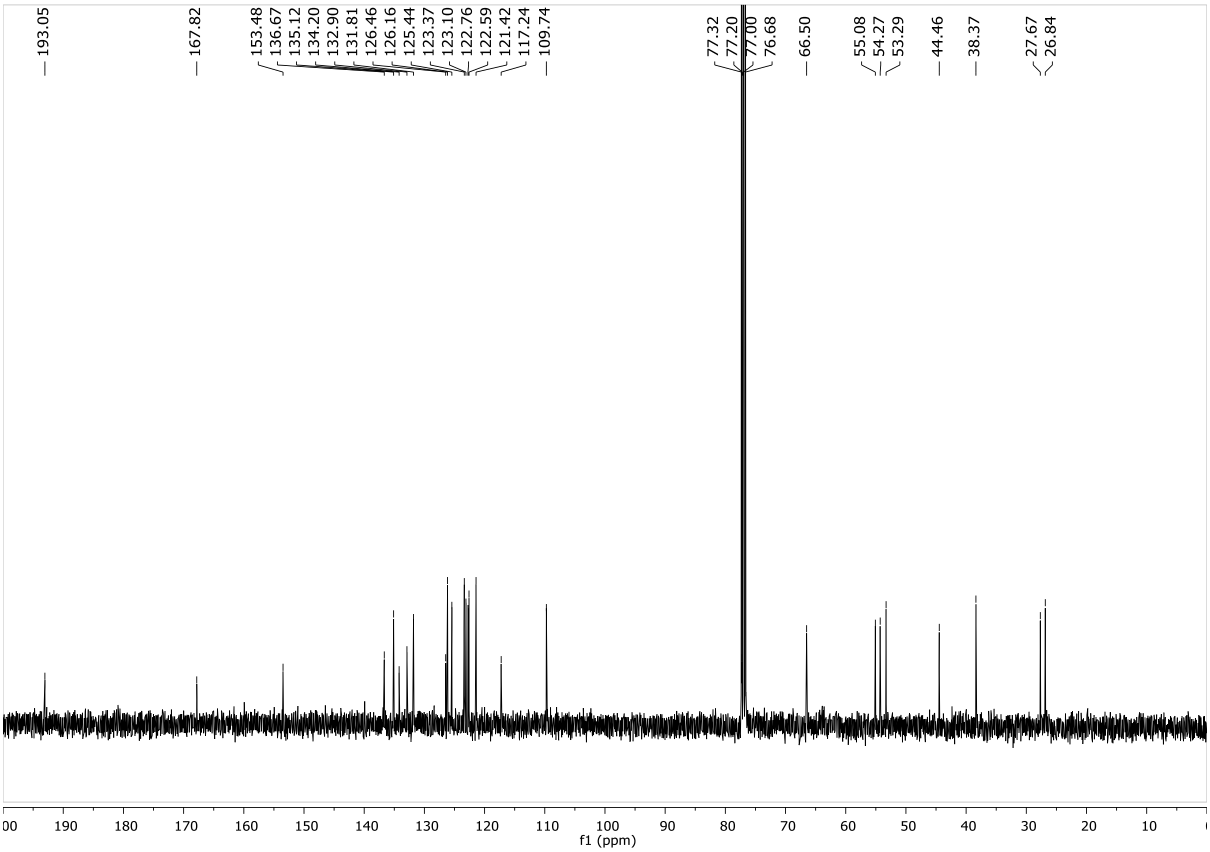
193.05
167.82
153.48
136.67
135.12
134.20
132.90
131.81
126.46
126.16
125.44
123.37
123.10
122.76
122.59
121.42
117.24
109.74
77.32
77.20
77.00
76.68
66.50
55.08
54.27
53.29
44.46
38.37
27.67
26.84
f1 (ppm)

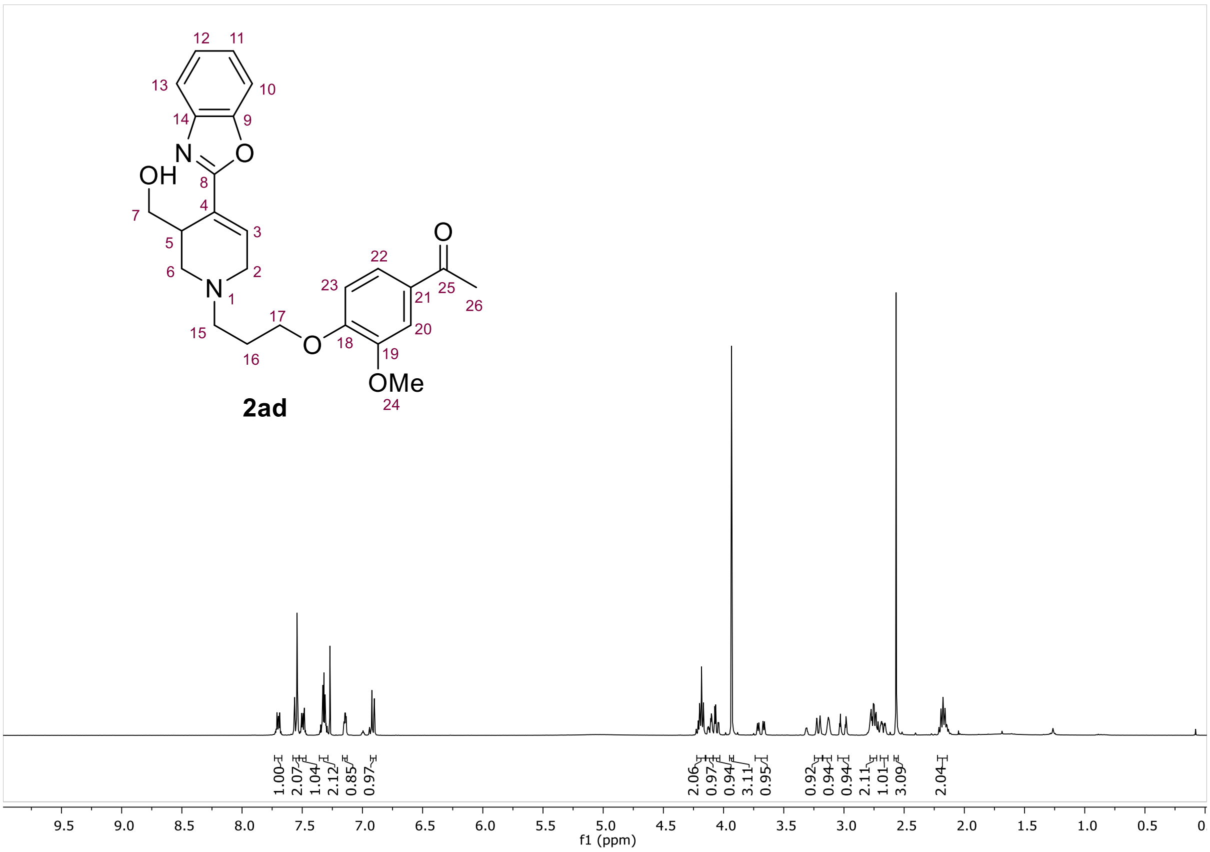
OH
N
O
OMe
O
2ad
f1 (ppm)

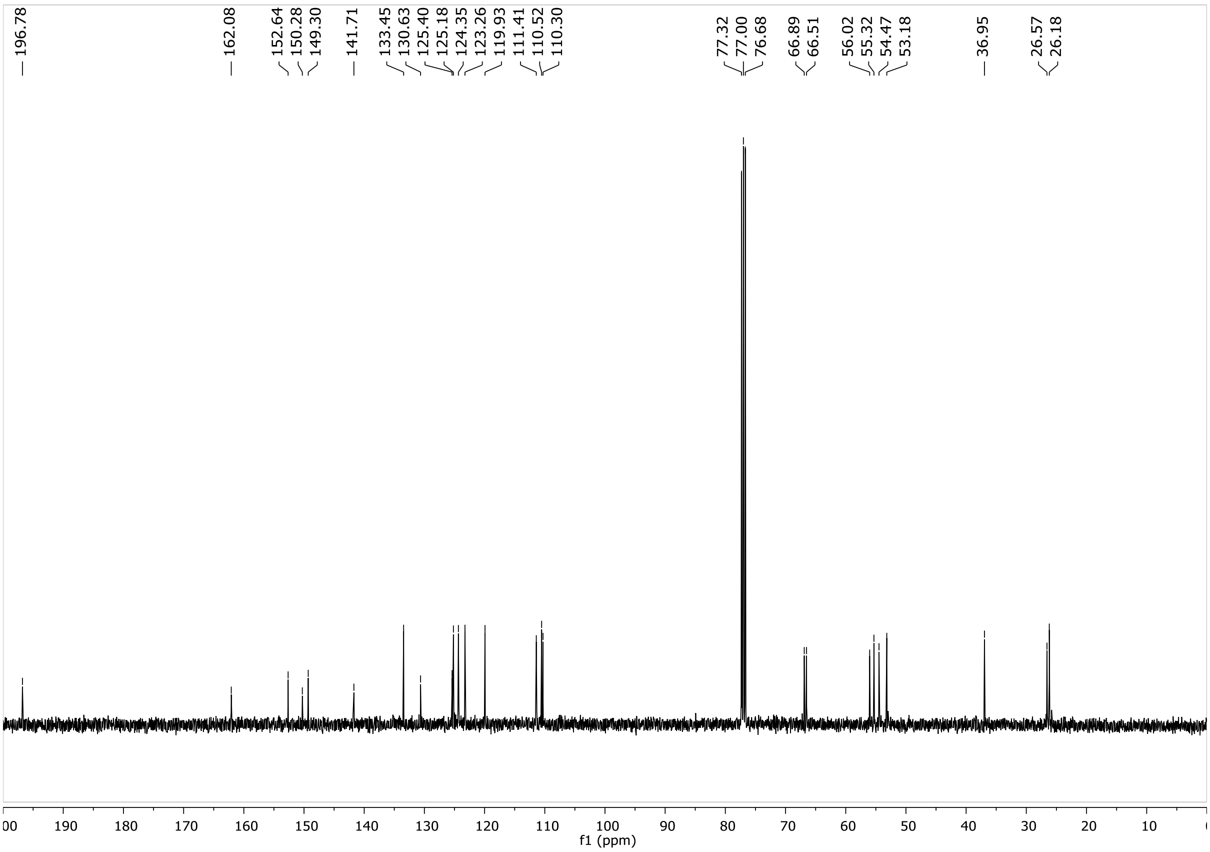
196.78
162.08
152.64
150.28
149.30
141.71
133.45
130.63
125.40
125.18
124.35
123.26
119.93
111.41
110.52
110.30
77.32
77.00
76.68
66.89
66.51
56.02
55.32
54.47
53.18
36.95
26.57
26.18
f1 (ppm)

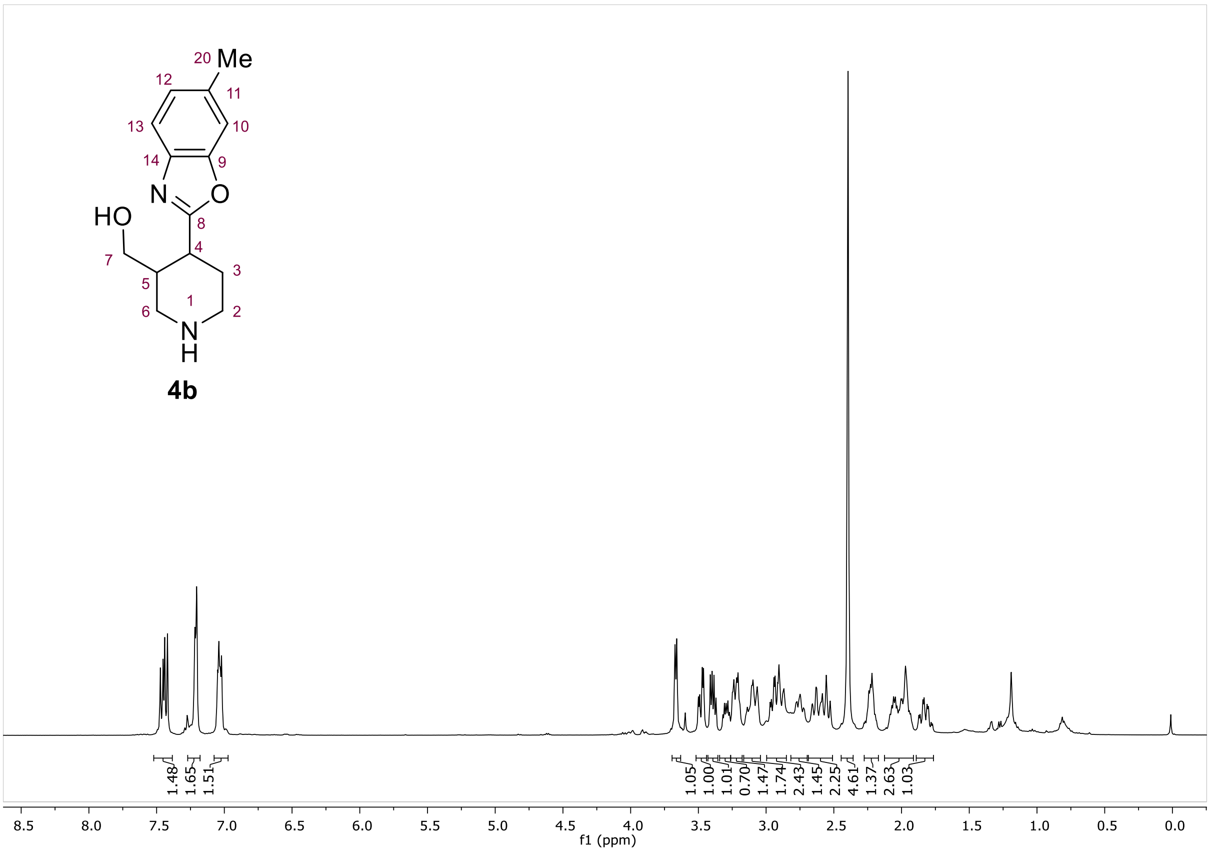
20 Me
12
11
13
10
14
9
N
O
8
HO
4
7
3
5
1
6
2
N
H
4b
1.48
1.65
1.51
1.05
1.00
1.01
0.70
1.47
1.74
2.43
1.45
2.25
4.61
1.37
2.63
1.03
f1 (ppm)

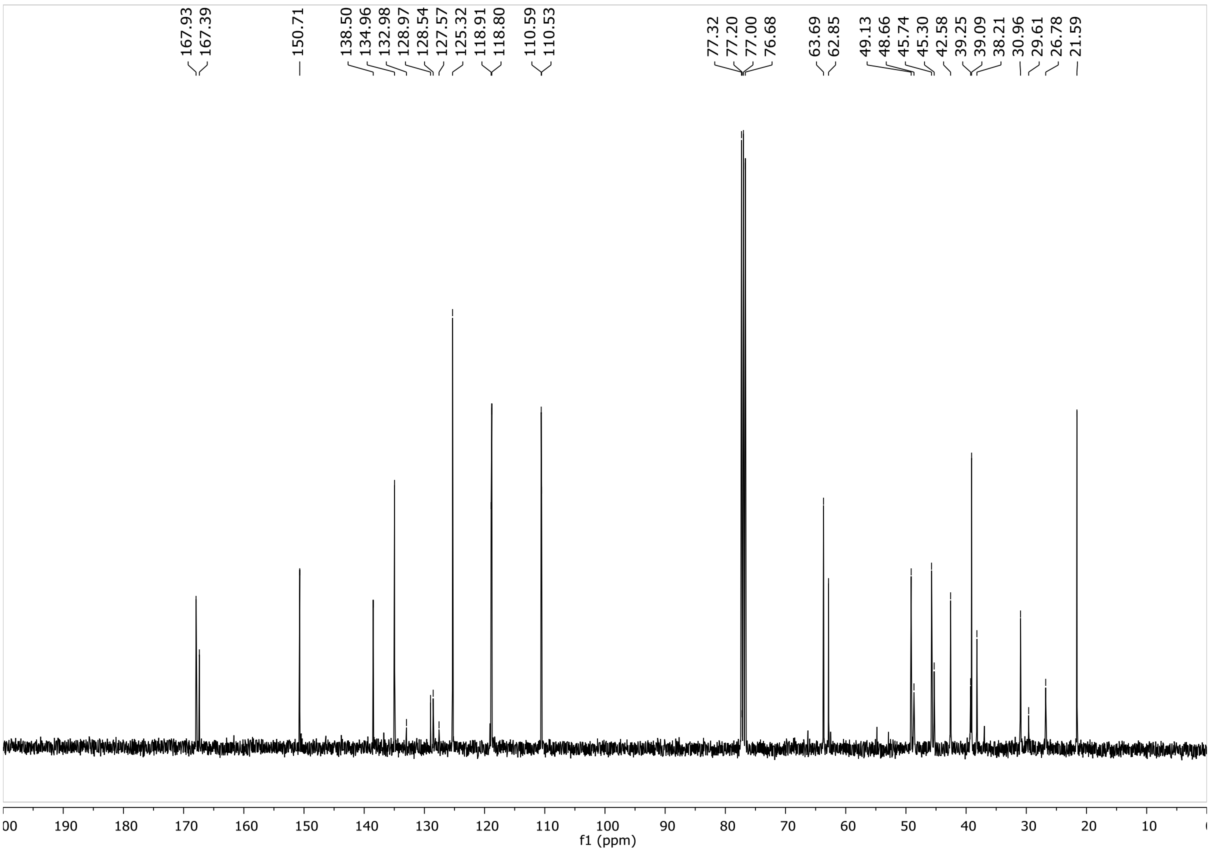
167.93
167.39
150.71
138.50
134.96
132.98
128.97
128.54
127.57
125.32
118.91
118.80
110.59
110.53
77.32
77.20
77.00
76.68
63.69
62.85
49.13
48.66
45.74
45.30
42.58
39.25
39.09
38.21
30.96
29.61
26.78
21.59
f1 (ppm)
